# Lysosomal and synaptic dysfunction markers in longitudinal cerebrospinal fluid of de novo Parkinson’s disease

**DOI:** 10.1038/s41531-024-00714-1

**Published:** 2024-05-17

**Authors:** Michael Bartl, Johanna Nilsson, Mohammed Dakna, Sandrina Weber, Sebastian Schade, Mary Xylaki, Bárbara Fernandes Gomes, Marielle Ernst, Maria-Lucia Muntean, Friederike Sixel-Döring, Claudia Trenkwalder, Henrik Zetterberg, Ann Brinkmalm, Brit Mollenhauer

**Affiliations:** 1 Department of Neurology, University Medical Center Goettingen, Goettingen, Germany; 2 Institute for Neuroimmunology and Multiple Sclerosis Research, University Medical Center Goettingen, Goettingen, Germany; 3 Institute of Neuroscience and Physiology, The Sahlgrenska Academy at the University of Gothenburg, Mölndal, Sweden; 4 Paracelsus-Elena-Klinik, Kassel, Germany; 5 Institute of Diagnostic and Interventional Neuroradiology, University Medical Center Goettingen, Goettingen, Germany; 6 Department of Neurology, Philipps-University, Marburg, Germany; 7 Department of Neurosurgery, University Medical Center Goettingen, Goettingen, Germany; 8 Clinical Neurochemistry Laboratory, Sahlgrenska University Hospital, Mölndal, Sweden; 9 UK Dementia Research Institute at UCL, London, UK; 10 Department of Neurodegenerative Disease, UCL Institute of Neurology, London, UK; 11 Hong Kong Center for Neurodegenerative Diseases, Hong Kong, China

**Keywords:** Parkinson's disease, Parkinson's disease

## Abstract

Lysosomal and synaptic dysfunctions are hallmarks in neurodegeneration and potentially relevant as biomarkers, but data on early Parkinson’s disease (PD) is lacking. We performed targeted mass spectrometry with an established protein panel, assessing autophagy and synaptic function in cerebrospinal fluid (CSF) of drug-naïve de novo PD, and sex-/age-matched healthy controls (HC) cross-sectionally (88 PD, 46 HC) and longitudinally (104 PD, 58 HC) over 10 years. Multiple markers of autophagy, synaptic plasticity, and secretory pathways were reduced in PD. We added samples from prodromal subjects (9 cross-sectional, 12 longitudinal) with isolated REM sleep behavior disorder, revealing secretogranin-2 already decreased compared to controls. Machine learning identified neuronal pentraxin receptor and neurosecretory protein VGF as most relevant for discriminating between groups. CSF levels of LAMP2, neuronal pentraxins, and syntaxins in PD correlated with clinical progression, showing predictive potential for motor- and non-motor symptoms as a valid basis for future drug trials.

## Introduction

Parkinson’s disease (PD) is an increasingly prevalent, progressive, and complex neurodegenerative disease that lacks a conclusive panel of biomarkers that assess the rate, trait, fate, and state^1–4^ of the disease. PD diagnosis is still clinically based and usually made when the majority of affected neurons have already degenerated. In this context, non-motor symptoms like hyposmia and isolated REM (rapid eye movement) sleep behavior disorder (iRBD) are very valuable in identifying those at risk of developing the disease. iRBD is very specific, and therefore, the most powerful prodromal entity of PD. It is characterized by dream-enacting behaviors during REM sleep and a high conversion rate to an α-synucleinopathy, including PD and dementia with Lewy bodies and multiple system atrophy (MSA)^5^.

PD, mostly but not exclusively, affects dopaminergic neurons, especially in the substantia nigra pars compacta. α-synuclein (αSyn) plays a key role in its pathology^6^ and is physiologically localized mainly in presynaptic terminals of neurons. In certain synucleinopathies, including PD and iRBD^7^, it accumulates within the neuronal soma, described as Lewy body inclusions^8^. Nevertheless, the utility of quantifying total levels of αSyn as a biomarker is limited. It is lower in the cerebrospinal fluid (CSF) and plasma of PD patients compared to healthy controls, but no longitudinal changes or predictive potential have been detected^9^. α-synuclein seeding aggregation assays (αSyn-SAAs) were recently established that diagnose α-synucleinopathy in PD with high sensitivity and specificity, therefore enabling the identification of molecular heterogeneity and increased PD risk among patients. SAAs have not yet been qualified as progression markers and they have not been sufficiently linked to the specific pathophysiology^10^. Further, they are binary assays, helpful for diagnostics but not for tracking changes over time. Therefore, many questions remain to be answered. Research for effective diagnostic and prognostic biomarkers in early and prodromal disease stages needs to be widened^11^.

Synaptic dysfunction is a hallmark of many neurodegenerative diseases, including PD. Multiple established risk factors and causative PD genes are known to influence synaptic functioning^12^. Lysosomal dysfunction is also implicated in PD: multiple variants in lysosomal storage disorder genes are associated with an increased risk of PD^13^ and previous studies suggest that there are alterations of the autophagic and endolysosomal system in PD^14^. Furthermore, accumulation of autophagic vacuoles is evident in PD patients’ brain cells, accompanied by a decrease in endolysosomal markers like LAMP2^15,16^.

Assessing lysosomal and synaptic functioning biomarkers in PD could potentially improve diagnostic and prognostic accuracy by focusing on the early disease phase and enabling treatment response monitoring in future drug trials. Previous attempts to assess synaptic functioning are based on research in synaptic proteins in CSF of patients with Alzheimer’s disease^17–19^. Some earlier studies, mainly focused on PD, either found no differences between PD and controls or had inconsistent results^20–25^.

Data on de novo PD and longitudinal analysis are generally lacking and no studies on prodromal PD stages are available to date.

We sought to fill this gap by investigating a combined panel of lysosomal and synaptic markers validated in different cohorts^26–28^. Over a 10-year follow-up, we used this approach to longitudinally assess differently expressed proteins in CSF between subjects with de novo PD and sex- and age-matched healthy controls (cross-sectional 88 PD, 46 HC; longitudinal 104 PD, 58 HC) from our ongoing, prospective, single-center de novo Parkinson’s disease (*DeNoPa*) cohort^29^. Further, we added samples from prodromal subjects with video-polysomnography diagnosed iRBD (cross-sectional 9, longitudinal 12) as an exploratory approach. We integrated our data into one^30,31^ of the two^31,32^ newly proposed neuronal αSyn-disease integrated staging systems and correlated the results with the available longitudinal clinical data. Linear mixed modelling was performed to analyze progression over time, and we evaluated the predictive and discriminative potential of these markers with machine learning algorithms.

## Results

### The DeNoPa cohort

We analyzed CSF samples from subjects of our longitudinal de novo PD (DeNoPa) cohort and included, from baseline, 88 PD patients (62 men, 70.5%) and 46 healthy controls (HC) (34 men, 73.9%) for whom CSF samples were available at all time points. The mean age was 65 years ( ± 9.8) for PD and 66 years ( ± 7.2) for HC. The mean Hoehn and Yahr Stage in the PD group was 2 ( ± 0.4), and the mean MDS-UPDRS part III score was 23 ( ± 11.4). MMSE (mini-mental state examination) did not significantly differ between PD and HC (28 in both groups), but the MoCA (Montreal cognitive assessment) score was lower in PD (PD: 24, healthy controls: 26). Further, we included an exploratory test set of nine subjects with iRBD (2 men, 2.2%) mean age 65 ( ± 9). The demographics and clinical characteristics of the cohort are shown in Table 1.

**Table 1:** Baseline demographics and some CSF biomarkers of the included subjects, SAA data displays positive and negative results of the available samples

Baseline	HC (*N* = 46)	PD (*N* = 88)	RBD (*N* = 9)	Adj. *p*-values
Sex				0.008*
- female	12 (26.1%)	26 (29.5%)	7 (77.8%)	
- male	34 (73.9%)	62 (70.5%)	2 (22.2%)	
Age				0.989
- Mean (SD)	65.6 (7.23)	65.136 (9.81)	64.78 (8.97)	
BMI				0.012*
- Mean (SD)	26.97 (4.63)	28.447 (4.71)	24.99 (3.68)	
MDS-UPDRS-III				< 0.001*
- Mean (SD)	0.63 (1.48)	22.966 (11.44)	3.11 (2.71)	
MDS-UPDRS total score				< 0.001*
- Mean (SD)	3.39 (3.63)	37.278 (16.76)	18.11 (10.17)	
Hoehn & Yahr				< 0.001*
- Mean (SD)	0.00 (0.00)	2.045 (0.74)	0.11 (0.33)	
MMSE total score				0.278
- Mean (SD)	28.64 (1.18)	28.207 (1.48)	28.56 (0.88)	
MoCA total score				0.013*
- Mean (SD)	26.05 (2.33)	24.257 (3.265)	25.44 (2.60)	
CSF albumin quotient				0.019*
- Mean (SD)	7.65 (3.74)	8.916 (4.507)	6.03 (1.88)	
CSF β-amyloid (Aβ)				0.154
- Mean (SD)	870.65 (211.07)	870.401 (210.363)	673.67 (65.68)	
CSF phospho-tau protein (pTau)				0.066
- Mean (SD)	44.97 (16.72)	42.192 (16.326)	27.77 (3.36)	
CSF Total tau protein (tTau)				0.099
- Mean (SD)	256.84 (122.39)	235.632 (115.464)	159.00 (16.82)	
CSF neurofilament light chains (NfL)				< 0.001*
- Mean (SD)	536.28 (203.38)	702.10 (405.19)	361.83 (85.92)	
α-synuclein seeding aggregation assay (SAA)	41 (−) out of 41	68/12 (+/−) out of 80	8/1 (+/−) out of 9	< 0.001*

PD subjects displayed a higher CSF albumin quotient, indicative of a blood-brain barrier dysfunction. Typical CSF Alzheimer’s disease markers (Aβ, t- and p-tau) showed no differences between the groups. As reported before, neurofilament light (NfL) was increased in PD samples. The *P*-value is adjusted for age and sex. *PD* Parkinson’s Disease, *HC* Healthy control, *iRBD* isolated REM sleep behavior disorder, *n* Number, *sd* Standard deviation, *CSF* Cerebrospinal fluid, *MDS-UPDRS* Movement Disorder Society – United Parkinson’s Disease Rating Scale, *MMSE* Mini-Mental State Examination, *MoCa* Montreal Cognitive Assessment Score.

*indicates statistically significant results.

For additional information on the differences between the subgroups see Supplementary Table 6.

We added our available α-synuclein–seed amplification assay (αSyn-SAA) data, based on high-throughput CSF αSyn-SAA, showing that 68 of 80 tested PD subjects were positive, as were 8 of the 9 iRBD subjects.

To increase the power of our longitudinal model, we also included additional samples from subjects with no available CSF samples at baseline. This resulted in a total of 12 iRBD (11 of 12 αSyn-SAA positive), 104 PD (87 of 100 αSyn-SAA positive), and 58 HC (all negative) subjects in our longitudinal model. Applying the neuronal αSyn-disease integrated staging system (NSD-ISS) led to four subjects categorized as stage NSD-2A (iRBD, S + D−), six in stage NSD-2B (iRBD, S + D + ) and 78 who met the criteria for stage 4 (PD, S + D + , moderate clinical impairment). See the methods section and Supplementary Table 4 and Supplementary Table 5 for details.

### Synaptic and lysosomal biomarker levels

The application of a targeted mass spectrometry biomarker panel (Table 2) showed nine markers that were differentially expressed between PD and healthy controls: neurosecretory protein VGF, amyloid-beta precursor protein (APP), the neuronal pentraxins and its corresponding receptor (NPTX1, NPTX2, NPTXR), secretogranin-2, neurogranin, syntaxin-7 and AP-2 complex subunit beta (AP2B1). The markers displayed significantly lower CSF levels in PD compared to healthy controls. All these markers also showed lower CSF levels in iRBD, with significant results for secretogranin-2 (*p* = 0.01). The analysis showed no changes in protein levels between PD and iRBD.

**Table 2:** Overview of the applied biomarker panel, the targeted peptide sequences, and the biological functions

Protein	Abbreviation	Accession	Sequence	Function
Neurosecretory protein VGF	VGF	O15240	AYQGVAAPFPK	Neurotransmitter secretion
			NSEPQDEGELFQGVDPR	
Neuronal pentraxin receptor	NPTXR	O95502	NNYMYAR	Glutamate receptor recruitment; synaptic plasticity
			LVEAFGGATK	
Neuronal pentraxin-1	NPTX1	Q15818	ETVLQQK	
			CESQSTLDPGAGEAR	
Neuronal pentraxin-2	NPTX2	P47972	VAELEDEK	
			ETVVQQK	
Amyloid-beta precursor protein	APP	P05067	VESLEQEAANER	Neuronal surface receptor
Secretogranin-2	SCG2	P13521	ALEYIENLR	Neuroendocrine protein, biogenesis of secretory granules
			VLEYLNQEK	
Neurogranin	NEUG	Q92686	KGPGPGGPGGAGVAR	Binds calmodulin, enhances synaptic transmission
Syntaxin-7	STX7	O15400	EFGSLPTTPSEQR	Vesicle trafficking
Syntaxin-1B	STX1B	P61266	QHSAILAAPNPDEK	
AP-2 complex subunit beta	AP2B1	P63010	AVWLPAVK	Mediating endocytosis
			IQPGNPNYTLSLK	
			QVFLATWK	
Chromogranin-A	CMGA	P10645	GLSAEPGWQAK	Aggregation and processing of secretory granules
			EDSLEAGLPLQVR	
GangliosideGM2 activator	SAP3	P17900	EVAGLWIK	Binding gangliosides, stimulating GM2 degradation
			IESVLSSSGK	
β-synuclein	SYUB	Q16143	EGVVQGVASVAEK	Presynaptic functioning
γ-synuclein	SYUG	O76070	ENVVQSVTSVAEK	
Complexin-2	CPLX2	Q6PUV4	AALEQPCEGSLTRPK	Vesicle trafficking
Rab GDP dissociation inhibitor alpha	GDIA	P31150	QLICDPSYIPDR	Vesicle trafficking
Phosphatidylethanolamine-binding protein 1	PEBP1	P30086	NRPTSISWDGLDSGK	Regulatory protein, presynaptic functioning
			LYEQLSGK	
Lysosome-associated membrane glycoprotein 2	LAMP2	P13473	IPLNDLFR	Chaperone-mediated autophagy
Cathepsin-F	CATF	Q9UBX1	TLLCSFQVLDELGR	Protein turnover, intracellular degradation
1433E	1433E	P62258	IISSIEQK	Adapter proteins, modulating general and specific pathways by activity regulation of the binding partners
1433 F	1433 F	Q04917	AVTELNEPLSNEDR	
1433 T	1433 T	P27348	AVTEQGAELSNEER	
1433Z	1433Z	P63104	VVSSIEQK	

The panel composition is based on untargeted and targeted proteomic approaches including PD subjects and own previous publications^33,40,64,65^.

**Fig. 1:**
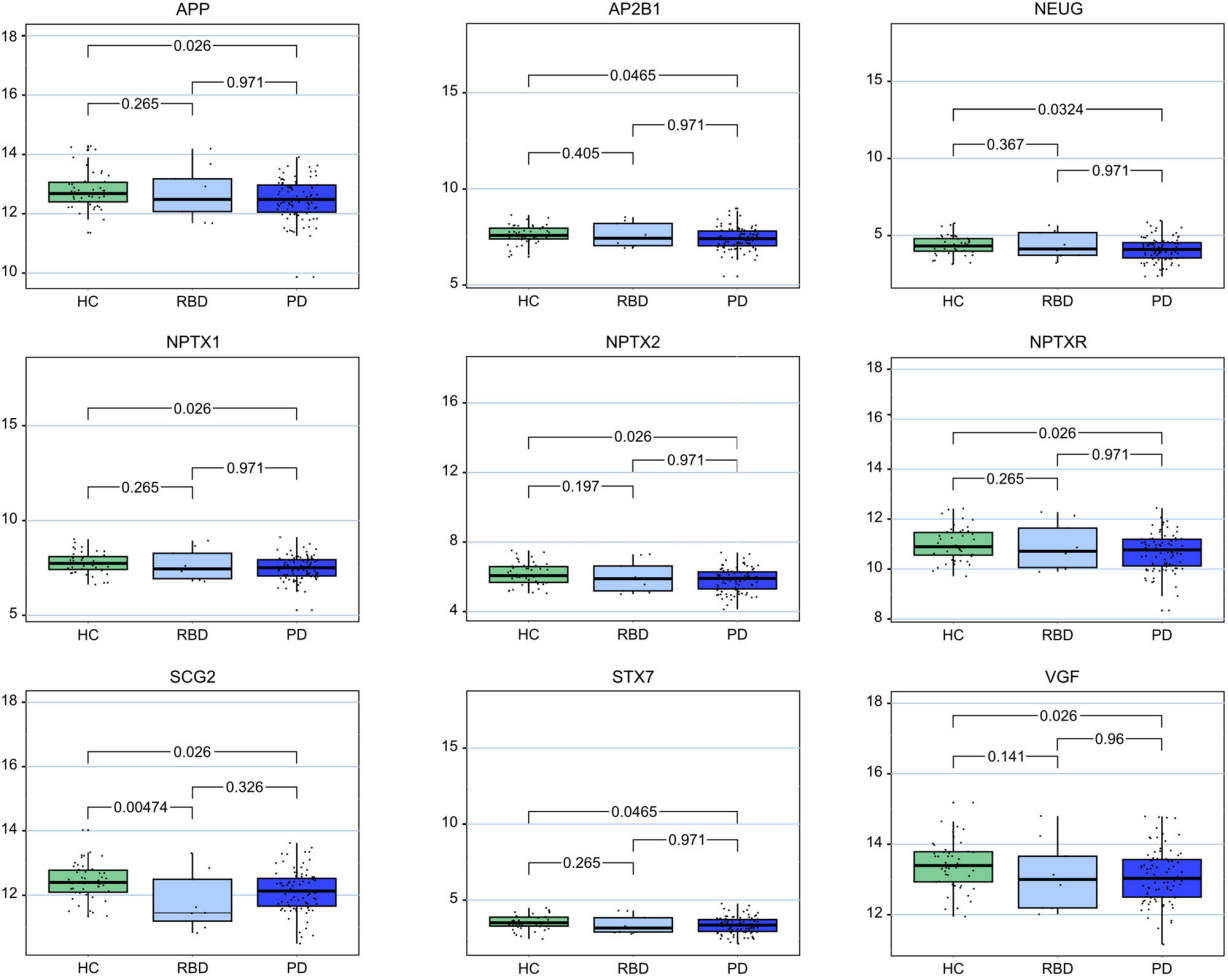
Box plots of the significantly differently expressed proteins. The center line displays the median, the box the interquartile range and the whiskers the minimum and maximum value of the data. HC Healthy controls, RBD isolated REM sleep behavior disorder, PD: Parkinson´s disease, APP Amyloid-beta precursor protein, AP2B1 AP-2 complex subunit beta, NEUG Neurogranin, NPTX1 Neuronal Pentraxin-1, NPTX2 Neuronal Pentraxin-2, NPTXR Neuronal Pentraxin receptor; SCG2: Secretogranin-2, STX7 Syntaxin-7; based on the protein measurements at baseline [sample size: HC (*N* = 46); PD (*N* = 88)].

Results are shown in Table 3 and box plots appear in Fig. 1.

**Table 3:** Results of the assessed proteins in CSF samples, significantly differently expressed values are marked with *, *P*-value is adjusted for age, and sex and Benjamini-Hochberg FDR adjusted for multiple testing

de novo PD				exploratory iRBD		
Protein	**HC**	**PD**	**HC-PD adj.** ***p*-val**.	**iRBD**	**HC-iRBD adj.** ***p*-val**.	**PD-iRBD adj.** ***p*-val**.
Secretogranin-2	12.42	12.12	0.03*	11.79	0.01*	0.43
VGF	13.42	13.09	0.03*	13.12	0.12	0.92
Neuronal Pentraxin receptor	10.99	10.67	0.03*	10.91	0.26	0.96
Amyloid-beta precursor protein	12.79	12.49	0.03*	12.71	0.24	0.96
Neuronal Pentraxin-2	6.16	5.83	0.03*	5.99	0.17	0.96
Neuronal Pentraxin-1	7.77	7.49	0.03*	7.65	0.24	0.96
Neurogranin	4.36	4.08	0.03*	4.36	0.39	0.96
Syntaxin-7	3.53	3.34	0.05*	3.37	0.26	0.96
AP-2 complex subunit beta	7.62	7.41	0.05*	7.57	0.44	0.96
β-synuclein	3.29	3.10	0.12	2.99	0.11	0.47
Syntaxin-1B	3.69	3.48	0.07	3.51	0.26	0.96
Chromogranin-A	12.12	11.86	0.07	12.14	0.48	0.96
Ganglioside GM2 activator	12.70	12.59	0.27	12.43	0.22	0.82
γ-synuclein	4.48	4.40	0.43	4.13	0.12	0.43
Complexin-2	5.86	5.71	0.16	5.75	0.37	0.96
Rab GDP dissociation inhibitor alpha	6.96	6.85	0.16	6.86	0.42	0.96
Lysosome-associated membrane glycoprotein 2	14.04	13.93	0.34	14.09	0.56	0.96
Phosphatidylethanolamine-binding protein 1	6.78	6.72	0.50	6.73	0.56	0.96
1433 F	2.98	2.92	0.81	2.93	0.49	0.96
1433E	3.75	3.77	0.92	3.94	0.70	0.96
1433 T	-0.34	-0.33	0.95	-0.74	0.21	0.43
1433Z	5.59	5.65	0.41	5.42	0.45	0.54
Cathepsin-F	9.60	9.59	0.90	9.35	0.39	0.84

*PD* Parkinson’s Disease, *HC* Healthy control, *iRBD* Isolated REM sleep behavior disorder; based on the protein measurements at baseline [sample size: HC (*N* = 46); PD (*N* = 88)].

Statistically significant results are displayed in bold.

### Linear mixed models and longitudinal changes

The study includes longitudinal CSF samples collected during the follow-up visits every two years of a period of 10 years. The established linear mixed models, adjusted for age and sex, revealed no significant changes in the assessed peptides over time in the healthy controls, PD, or iRBD groups. As the PD subjects were de novo at baseline and started drug treatment during the follow-up, we wanted to monitor possible treatment effects on the marker levels and, therefore, analyzed the influence of the LEDD (levodopa equivalent daily dosage) on the marker panel by including it as a fixed effect term in the models. This showed no significant relationship to the medication. Only age and sex contributed significantly to the model. For all the assessed proteins the term TIME and the interaction term COHORT: TIME (COHORT = HC; PD; iRBD) are non-significant, indicating no significant changes over time between the groups and a stable expression level in CSF. Results can be found in Supplementary Table 3 and Supplementary Figure 7.

### Correlation analysis

**Fig. 2:**
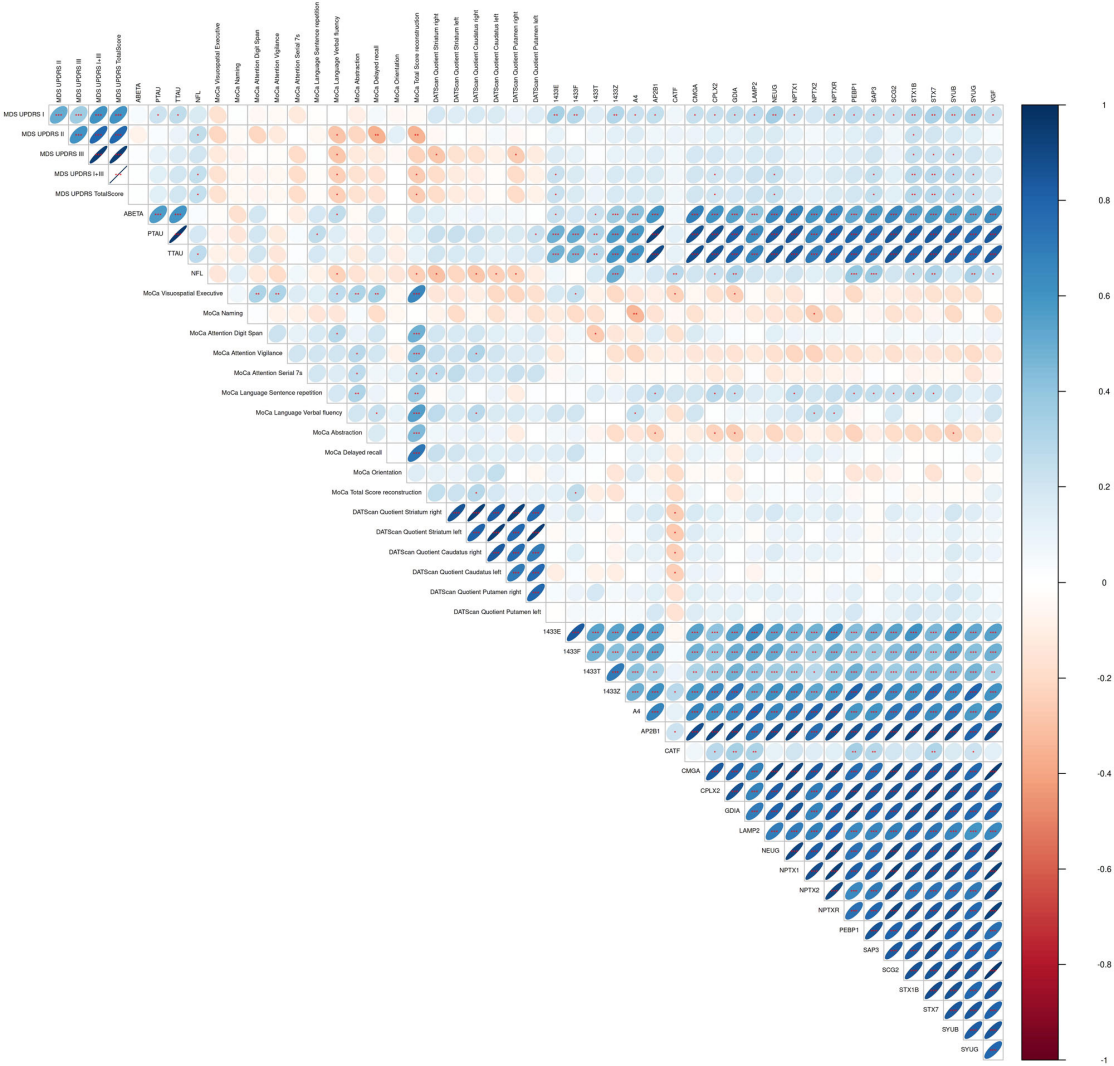
Correlation matrix of the measured panel and the available clinical data at baseline. The colours of the circles represent positive (blue) and negative (red) correlations. The circle sizes represent the size of the absolute correlation coefficients, larger signifying stronger correlations. *p < 0.05 **p < 0.01 ***p < 0.001, The *p*-values are Benjamini-Hochberg adjusted for multiple testing. MDS-UPDRS Movement Disorder Society – United Parkinson’s Disease Rating Scale, MoCa: Montreal Cognitive Assessment Score, SBR: specific binding ratio, based on the protein measurements at baseline [sample size: HC (*N* = 46); PD (*N* = 88)].

**Fig. 3:**
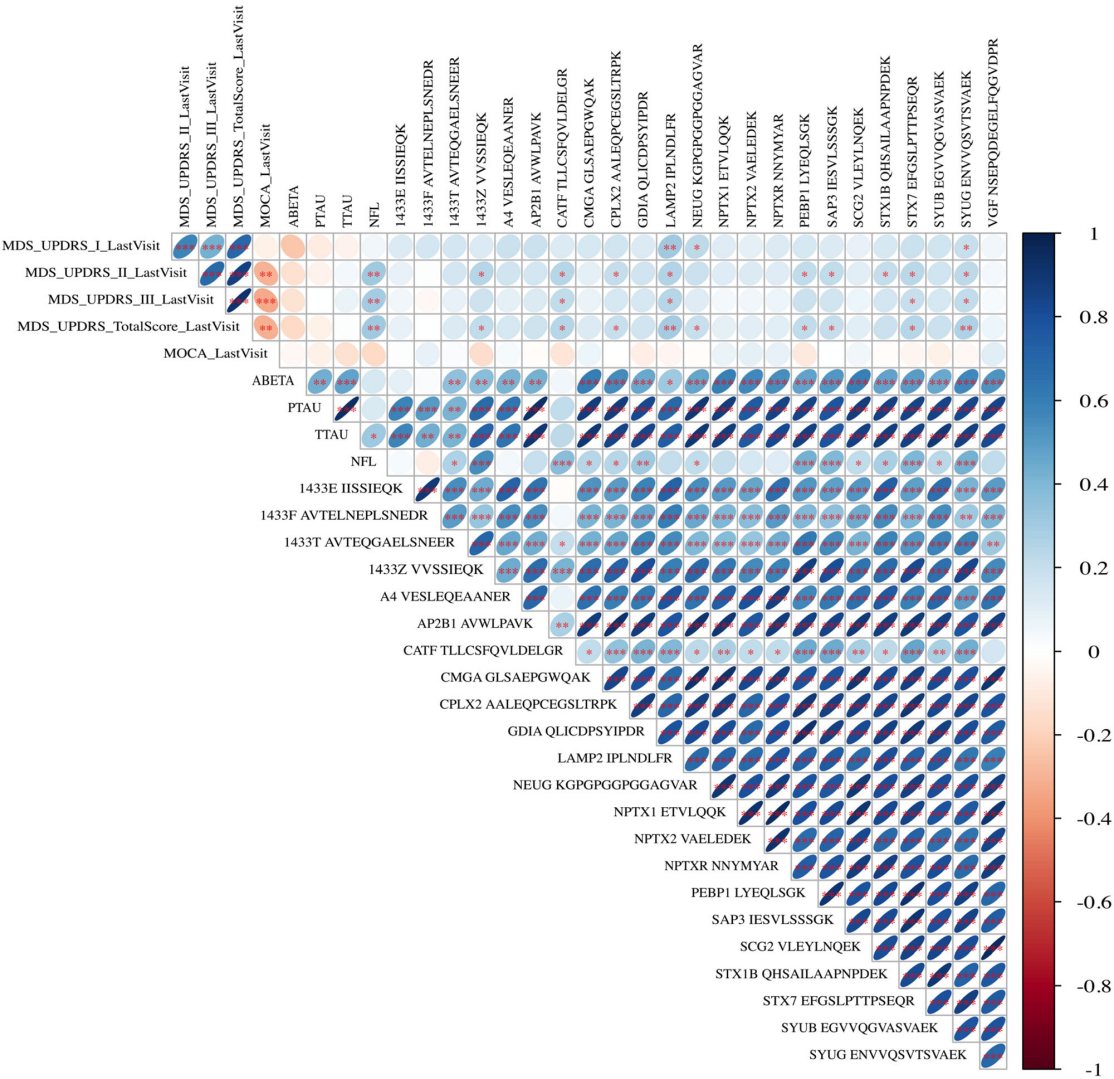
Predictive potential of the analyzed markers evaluated by the correlation of the baseline CSF levels with the available clinical data after 10 years of follow-up. The colours of the circles represent positive (blue) and negative (red) correlations. The circle sizes represent the size of the absolute correlation coefficients, larger signifying stronger correlations.*p < 0.05 **p < 0.01 ***p < 0.001, The *p*-values are Benjamini-Hochberg adjusted for multiple testing. Abbreviations: MDS-UPDRS = Movement Disorder Society – United Parkinson’s Disease Rating Scale, MoCa: Montreal Cognitive Assessment Score, based on the protein measurements at baseline [sample size: HC (*N* = 46); PD (*N* = 88)].

Multiple markers correlated with the available clinical and diagnostic data at baseline. This was especially the case for MDS-UPDRS part I: the rating of non-motor symptoms was strongly positively correlated with 20 markers, including VGF, NPTX1, NPTXR, neurogranin, γ-synuclein, β-synuclein, and AP2B1. Nine markers correlated positively with the MoCA domain “language sentence repetition”: SAP3, secretogranin-2, GDIA, PEBP1, AP2B1, syntaxin-7, syntaxin-1B, complexin-2, and NPTX1. Syntaxin-1B and 7 correlated positively with MDS-UPDRS parts I, II, III, and the total score, as well as with the MoCA domain “language sentence repetition”. This suggests a strong connection between the syntaxins and motor and non-motor impairment. The results from the dopamine-transporter–single-photon emission computed tomography (DAT-SPECT), namely the specific binding ratios of caudate nucleus and striatum on both sides, correlated negatively with Cathepsin-F. All correlations can be found in the correlation matrix (Figs. 2, 3).

### Predictive potential

The correlation of the baseline biomarker levels and the last available MDS-UPDRS scores after 10 years of follow-up revealed a predictive potential for several markers. Higher CSF levels at baseline predicted worse outcomes in MDS-UPDRS I for LAMP2, γ-synuclein, and Neurogranin. The markers Cathepsin-F, SAP3, LAMP2, PEBP1, Syntaxin-7, Syntaxin-1B, CPLX2, 1433Z, and γ-synuclein were predictive for MDS-UPDRS part II, Cathepsin-F, LAMP2, Syntaxin-7, and γ-synuclein for MDS-UPDRS part III, and Cathepsin-F, SAP3, LAMP2, PEBP1, Neurogranin, Syntaxin-7, CPLX2, 1433Z and γ-synuclein for the MDS-UPDRS total score. LAMP2 and γ-synuclein CSF baseline levels were predictive of the outcome in all MDS-UPDRS subscores. Cathepsin-F was predictive for all subscores of the MDS-UPDRS except for part I.

### Machine learning approach and discrimination analysis

**Fig. 4:**
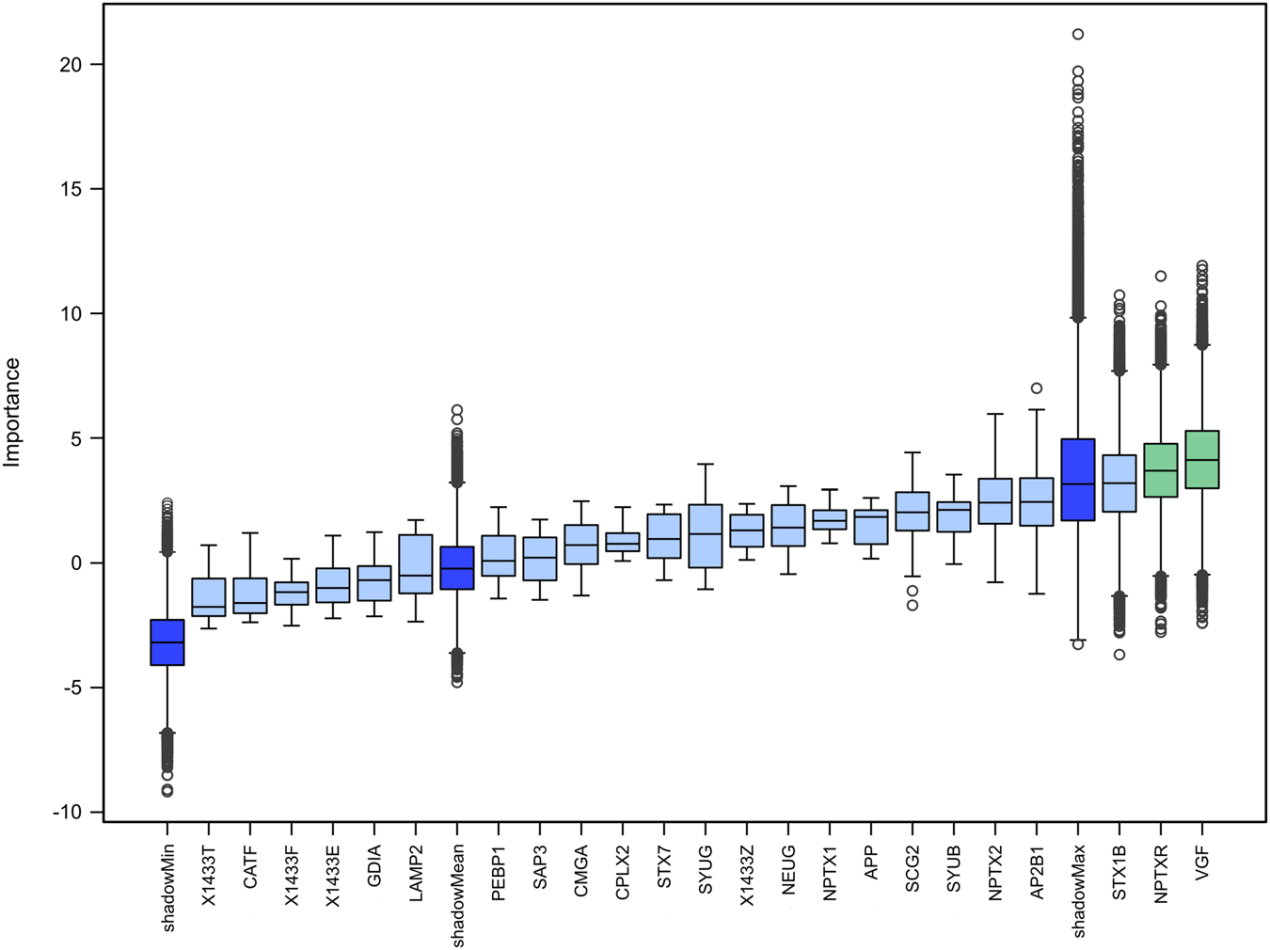
Results of the boruta algorithm. The analysis revealed Neurosecretory protein VGF and Neuronal pentraxin receptor with significant relevance in discriminating between Parkinson´s Disease and Healthy controls out of 100.000 iterations, based on the protein measurements at baseline [sample size: HC (*N* = 46); PD (*N* = 88); The center line displays the median, the box the interquartile range and the whiskers the minimum and maximum value of the data.

The Boruta analysis performed 100,000 iterations and the Boruta algorithm detected two proteins of high importance for the discrimination of the PD subjects from healthy controls, namely VGF and Neuronal pentraxin receptor (Fig. 4; based on the protein measurements at baseline (sample size: HC (*N* = 46); PD (*N* = 88)). Information on the classification accuracy is displayed in Supplementary Fig. 4a/b). Results of the discrimination analysis based on receiver operating characteristics (ROC) can be found in Supplementary Figure 7. The single markers showed Area Under the Curve (AUC) values around 0,6 between PD and HC.

## Discussion

In prodromal and early PD, we need effective biomarkers that reflect specific pathophysiological processes and are directly connected to disease progression and clinical phenotypes. This led us to apply a well-established biomarker panel in the CSF of prodromal and manifest PD patients as well as healthy subjects. Synaptic and lysosomal dysfunction has been shown to play an important role in neurodegenerative diseases and the proteins included in the analyzed panel have been implicated in Alzheimer’s disease, PD, and other Parkinson syndromes^26–28^ Nevertheless, longitudinal data and studies in early and prodromal PD disease stages are lacking. To fill this gap, we (i) analyzed the potential of lysosomal and synaptic function markers to detect or monitor endolysosomal dysfunction and synaptic degeneration in an established cohort of de novo, unmedicated PD and matched healthy controls at baseline and longitudinally. We extended our study by including subjects with polysomnography (PSG)-verified iRBD in an exploratory approach. We further analyzed (ii) correlations with clinical data to evaluate the potential of these evaluated markers in routine clinical diagnostics and for monitoring therapeutic effects. The data were further tested for their potential to (iii) predict progression of motor and non-motor symptoms. Linear mixed modeling was performed to assess longitudinal changes and progression. Finally, we applied machine-learning algorithms to evaluate how the different markers discriminate between groups.

PD is a progressive neurodegenerative disease with synaptic loss^28^. Markers of synaptic dysfunction are increased in the CSF^28^ of patients with Alzheimer’s disease, while the levels of many markers (e.g. AP2B1, LAMP2, Secretogranin-2) were decreased in a cross-sectional PD cohort^33^.

In our approach, longitudinal samples of prodromal and de novo PD (NSD-ISS Stage 4 at baseline) subjects were assessed, identifying nine markers that were significantly decreased in CSF samples of PD subjects compared to healthy controls (VGF, APP, NPTX1/2 and NPTXR, neurogranin, secretogranin-2, syntaxin-7 and AP2B1). Interestingly, all these nine markers were lower in the iRBD, but only secretogranin-2 was already significantly decreased in prodromal iRBD (NSD-ISS stage 2 A/B). As we discuss below, the role of secretogranin-2 in vesicle-mediated transport could be important in early pathophysiological steps when αSyn aggregation takes place.

The correlation analysis of all measured markers showed a strong relationship between 20 markers and the baseline MDS-UPDRS part I score (that is, a mix of non-motor symptoms, like dementia, but also other features like sleep and depression, etc.). These included NPTX1 and the corresponding receptor, neurogranin, secretogranin-2, β- and γ-synuclein, and syntaxin-1B and 7.

From the MoCA score (cognition only), the domain “language sentence repetition” correlated the most with nine markers, including neuronal pentraxins, secretogranin-2, and the syntaxins. Syntaxin-1B and 7 also correlated positively with MDS-UPDRS parts I-III and total score. Cathepsin-F was the only marker correlating with several DAT-SPECT parameters.

As the longitudinal DeNoPa cohort collects extensive data every two years we analyzed longitudinal CSF samples over 10 years. The CSF levels of proteins, that were significantly differentially expressed at BL, remained at a stable expression level over time and showed no further significant change. The linear mixed model estimated the influence of dopaminergic drug intake, but found no significant correlation between the LEDD and the marker levels. As previously shown in many different studies, age and sex significantly influenced the model^34^. LAMP2 and γ-synuclein were the markers with the highest predictive potential regarding the progression of the MDS-UPDRS total score as well as its subscores (I, II, and III). The additional machine-learning approaches via the Boruta algorithm showed that the proteins VGF and NPTXR were most powerful in discriminating between PD and healthy controls.

Below we discuss the markers according to their biological function:

The neuronal pentraxins (NPTX1, NPTX2, NPTXR) are widely expressed at excitatory synapses and are very important in synaptic plasticity and in the clustering of glutamate receptors^35^.

Lower levels of the secreted glycoproteins NPTX1 and NPTX2 are related to more severe non-motor PD symptoms and cognitive deficits, measured by MDS-UPDRS part I, supporting previous findings in PD^33,36,37^.

NPTX1/2 is upregulated in the substantia nigra of PD subjects while NPTXR is downregulated. NPTX2, similar to αSyn, is present in Lewy bodies in the substantia nigra^38^. Lower NPTX2 CSF levels in PD could be connected to the accumulation in Lewy bodies, similarly to αSyn. NPTXR contributed significantly to our machine-learning model.

Neurogranin is a post-synaptic protein, a neuronal injury marker connected to calmodulin regulation. Higher CSF levels of neurogranin have been reported in Alzheimer’s disease^19,21^. However, in contrast to Alzheimer’s disease, CSF levels in PD were lower in our study compared to healthy controls and positively correlated with MDS-UPDRS part I.

In this study, CSF levels of markers associated with synaptic plasticity and presynaptic functioning showed lower values in PD compared to healthy controls, and correlated mostly with non-motor symptoms. Synaptic dysfunction seems to be already present in prodromal and early disease stages. It reflects motor as well as non-motor symptoms, including cognitive decline, which is in line with previous studies in neurodegenerative disorders^28,39^.

Neurosecretory protein VGF is a member of the chromogranin/secretogranin family. It regulates secretory pathways.

VGF levels were lower in PD in our study and the protein was marked as important by the machine-learning model, revealing a discriminatory potential between early PD and healthy controls. Available data for VGF reports decreased levels in dementia with Lewy bodies, amyotrophic lateral sclerosis, Alzheimer’s disease, and brain tissue of PD subjects^33,40^. VGF is also decreased in patient plasma samples. In a 6-hydroxydopamine-(6OHD)-induced lesions rat model (a common experimental PD animal model with lesions in the medial forebrain bundle), brain tissue and plasma samples were reduced compared with controls. Interestingly, these were restored by levodopa treatment^41^.

Secretogranin-2 regulates the packing and sorting of peptide hormones and neuropeptides into secretory vesicles and is generally considered a marker for secretory granules, dense-core vesicles in neurons and neuroendocrine cells^42^. Secretogranin-2 CSF levels were not only significantly lower in PD but also in iRBD compared with healthy controls, with the PD levels lying in between. They were also positively correlated with MDS-UPDRS part I and MoCA domain “language sentence repetition”.

Recently, the SCG2 gene was reported as a signal integrator of glutamate and dopamine inputs^43^. Lower CSF levels have been shown in PD as well as PD with *GBA*-(lysosomal enzyme glucocerebrosidase) mutation, the most common genetic risk factor for PD^33^. In a 6OHD rat model, increased SCG2 mRNA levels were found in brain tissue samples and validated via immunohistochemical staining. In addition, these levels even increased during chronic levodopa treatment^43^. Nevertheless, in our linear mixed-model analysis LEDD did not significantly influence Secretogranin-2 or VGF CSF levels.

The validation of decreased Secretogranin-2 levels in iRBD points towards its role early in the disease course during the loss of synapses and preceding neuronal degeneration. Its function is strongly connected to vesicle-mediated transport and lysosomal dysfunction, leading to a potential relation to αSyn and its aggregation.

The proteins syntaxin-7 and syntaxin-1B are relevant for vesicle trafficking and higher CSF levels of the syntaxins have been reported in Alzheimer’s disease^44^. Here, PD subjects had lower CSF levels and the markers also correlated positively with MDS-UPDRS part I, II, III and total score and the MoCA domain “language sentence repetition” and were predictive of MDS-UPDRS II and STX-7, as well as MDS-UPDRS part III and total score. Therefore, secretion and vesicular trafficking seem to reflect parts of PD pathophysiology, especially regarding the correlation with the clinical picture^33^.

Endolysosomal function and chaperon-mediated autophagy are key components of PD pathology^33,45^. Several markers are involved in these pathways according to our panel. Previous data reported increased LAMP2 CSF levels in Alzheimer’s disease and lower in PD compared to controls^46^. Here, LAMP2 levels were not significantly different between the groups but seemed to be a strong predictor for the clinical outcome in PD. LAMP2 CSF levels at baseline were predictive of higher scores in MDS-UPDRS I-III and the total score over the ten years of follow-up.

SAP3 is a lysosomal protein that catalyzes the degradation of gangliosides and is involved in α-synuclein proteostasis^46^. SAP3 levels were associated with worse clinical performance in PD, indicated by the positive correlations with MDS-UPDRS part I and total score and the MoCA domain “language sentence repetition”. Previous data reported higher CSF levels in Alzheimer’s disease and dementia with Lewy bodies and lower levels in PD^33^.

Cathepsin-F was predictive for MDS-UPDRS parts II, III, and total score and correlated with the DAT-SPECT quotient of N. caudatus and striatum on both sides. It is a member of the papain-like cysteine protease family. It is involved in protein degradation after endocytosis and presentation of protein fragments via major histocompatibility complex (MHC) class II to T lymphocytes^47^. Therefore, proteins of αSyn proteostatis are already altered in the early disease stages and are linked to immunological presentation, which is congruent with the known activity of αSyn-specific T-cell populations both prior to and at the onset of motor symptoms^48^.

Besides the illustration of specific disease pathways, biomarkers have clinically relevant implications. We found several markers, whose baseline CSF levels were correlated with motor and non-motor clinical symptoms, that were associated with clinical outcomes. MDS-UPDRS part I correlated with 20 proteins. It rates non-motor symptoms in PD including cognitive impairment, hallucinations, affective symptoms like anxiety and depression, sleeping problems, and vegetative symptoms like urinary problems, which are known to significantly impair disease-related quality of life^49^. Synaptic dysfunction seems to affect pronounced non-motor aspects of the disease, which is in line with findings showing a connection between synaptic dysfunction and cognitive decline in Alzheimer’s disease^26^.

Regarding prediction, LAMP2 levels were strongly connected with the performance of all MDS-UPDRS subscores and the total score. Cathepsin-F was associated with the outcome of all but part I of the MDS-UPDRS and correlated with DAT-SPECT results. LAMP2 seems to be an ideal marker to predict disease progression over time, for motor and non-motor symptoms, independent of drug therapy. Previous data showed that αSyn preformed fibrils impair autophagy flux resulting in the degradation of LAMP2 in activated microglia, forming a direct connection between synaptic dysfunction, αSyn pathology and neuroinflammation^50^.

As the described synaptic markers are correlated with cognitive functioning and are prominently altered in Alzheimer’s disease, it is interesting, that many correlated with the MoCA score, especially the part “language sentence repetition”. Studies show that sentence repetition and generation are altered in PD before^51^. Multiple markers from our panel correlated with pTau. This can be due to overall neurodegeneration, but it could also be speculated, that pTau itself is involved in synaptic dysfunction. Our panel showed more correlations with clinical parameters like MDS-UPDRS as pTau did. As it is indicated by previous publications in AD^52^, a possible future development could be combined panels of new markers with established biomarkers like pTau and β-amyloid could be an effective tool in patients’ stratification.

A strength of our study is the validated and established protein panel as well as the established study cohort. We present, longitudinal, high quality and deeply phenotyped clinical data from prodromal and PD subjects staged with the new NSD-ISS. This has been previously recommended by researchers in the field^33^.

Limitations include the small number of iRBD subjects in the exploratory group, a point we will address with our ongoing iRBD cohort in the near future. Other possible limitations are the lack of validation in corresponding brain or blood samples, and the panel itself. This may not be fully representative of all relevant autolysosome and synaptic processes in PD.

We included 104 PD subjects in our analysis, 100 with an available α-synuclein SAA testing. 13 (87%) of these PD subjects showed a positive αSyn-SAA result.

Possible reasons for the negative SAA results include inaccuracy in the Parkinson’s diagnosis (around 50% of the initial PD diagnosis in untreated subjects are wrong^53^), the sensitivity (82.6%) and specificity (88.2%) of CSF α-synuclein SAA at the time point of the initial diagnosis in other cohorts^54,55^ and the possible presence of genetic variants such as *LRRK2*. *LRRK2* patients show less α-synuclein pathology and significantly fewer positive SAA results^56^. Our longitudinal cohort will be extensively genetically characterized in the future (thru GP2) and as part of our established brain donation program, we will receive some autopsy-conformed diagnoses in the future.

Autophagy, and lysosomal and synaptic dysfunction play a relevant role in PD pathology, but not all known synaptic markers showed the same importance in PD and prodromal subjects. LAMP2, Cathepsin-F and the Syntaxins were most predictive for the clinical outcome and progression over time. Neurosecretory protein VGF and the Neuronal pentraxin receptor were most able to discriminate the groups in our machine learning models and correlated with clinical measures. VGF showed already reduced levels in prodromal iRBD.

The validation of these markers in a larger cohort of prodromal PD subjects would be a promising way to assess new biomarker candidates for clinical trials and possible translation into clinical practice in the future.

## Methods

### The DeNoPa cohort

Recently diagnosed patients with PD and matched healthy controls were enrolled at the Paracelsus-Elena-Klinik, Kassel, Germany between 2009 and 2012. Participants had to be aged between 40 and 85 years old with newly diagnosed PD with at least two of the following criteria: resting tremor, bradykinesia, and rigidity according to UK Brain Bank Criteria; UKBBC)^57^. To be eligible for inclusion, participants had to meet the criteria for de novo PD: any exposure to L-dopa had to have been less than 2 weeks and not within the 4 weeks prior to study entry^29^. Reasons for exclusion were severe vascular encephalopathy, normal-pressure hydrocephalus (NPH) shown on magnetic resonance imaging (MRI) (when available at screening or when detected during imaging studies), evidence for multiple system atrophy (MSA) or progressive supranuclear palsy (PSP) as well as medication-induced PD.

Healthy individuals between 40 and 85 years, matched to the PD group by age, sex, and education level showing no pathological condition of the central nervous system and a negative family history of PD were included as controls. Biannual longitudinal clinical data were collected at baseline and at 2, 4, 6, 8, and 10 years of follow-up in 104 PD and 94 healthy controls (flow-chart see Supplementary Fig. 1a/b)^29^. Motor function in DeNoPa was assessed with the Movement Disorder Society-Unified Parkinson’s Disease Rating Scale (MDS-UPDRS) parts I, II, III, and total score. Cognitive decline was assessed using the Mini-Mental-State Examination (MMSE) and MoCA (Montreal cognitive assessment) in all patients at baseline, and 2, 4, 6, 8, and 10 years of follow-up.

Clinical diagnosis was reassessed at 24 month follow-up in the On-state for all patients at each follow-up by consensus of two teams of independent neurologists (CT/FS-D and BM/JE) and based on clinical examination and impression, reassessment of UPDRS, the effectiveness of dopaminergic medication to motor symptoms and the evaluation of cognitive decline, hallucinations, autonomic dysfunction and symptoms indicative for atypical PD^58^.

iRBD was diagnosed through video polysomnography by experienced raters (CT, FS-D, MLM) on two consecutive nights according to established criteria^59,60^. The LEDD (levodopa equivalent daily dosage) was calculated based on Tomlison et al.^61^.

The iRBD cohort is part of the DeNoPa cohort, designed as a single center, longitudinal, observational study, still ongoing and recruiting subjects in iRBD, but not in PD and HC. Follow-up visits take place every two years for each single subject, explaining the differing numbers between the follow-up visits in the flow chart. Supplementary Fig. 1b displays the current numbers at the time point of submission. Dropouts are subjects that decided not to continue in the study or did not have an RBD confirmed by video polysomnography (vPSG). Additional information on the iRBD subjects is shown in Supplementary Table 7, including the assessment of hyposmia/anosmia indicated by Sniffin sticks´ **T**(reshold)**D**(iscrimination)**I**(identification) score ( < 30 indicative for hyposmia), Becks Depression Inventory indecx (BDI; > 8 can indicate a depressive episode), Parkinson’s disease non-motor symptoms scale (PD-NMS; <10 describes mild non-motor symptoms), Mini-mental-state examination (MMSE; < 27 indicative for possible cognitive impairment).

Final diagnosis refers to the consensus diagnosis that was made up to 10 years of follow-up, including initial dopamine-transporter–single-photon emission computed tomography (DAT-SPECT), biannual clinical evaluations, levodopa challenge, lasting response to levodopa, and the emergence of advanced PD features such as motor fluctuations or levodopa–induced dyskinesias. Abnormal/pathological DAT-SPECT was determined by a specialist in nuclear medicine by visual inspection or quantification.

### Sample collection

This study includes CSF samples taken at baseline and at the follow-up visits every two years. CSF was collected in polypropylene tubes (Sarstedt, Nümbrecht, Germany) directly after the plasma collection by lumbar puncture in the sitting position. Tubes were centrifuged at 2500 g at room temperature (20 °C) for 10 min and aliquoted and frozen within 30 min after collection at −80 °C until analysis. Before centrifugation, white and red blood cell counts in CSF were determined manually^29,58^. CSF β-amyloid 1–42, total tau protein (t-tau), phosphorylated tau protein (p-tau181) and neurofilament light chains (NFL) concentrations were measured by board-certified laboratory technicians, who were blinded to clinical data, using commercially available INNOTEST ELISA kits for the tau and Aβ markers (Fujirebio Europe, Ghent, Belgium) and the UmanDiagnostics NF-light® assay (UmanDiagnostics, Umeå, Sweden) for NFL. Total protein and albumin levels were measured by nephelometry (Dade Behring/Siemens Healthcare Diagnostics)^58^.

For the α-synuclein seeding aggregation assay (αSyn-SAA) the CSF samples were blindly analyzed in triplicate (40 μL/well) in a reaction mixture (0.3 mg/mL recombinant α-Syn (Amprion [California, USA]; catalog number S2020), 100 mM piperazine-N,N′-bis(2-ethanesulfonic acid) (PIPES) pH 6.50, 500 mM sodium chloride, 10 μM thioflavin T, and one bovine serum albumin (BSA)–blocked 2.4 mm silicon nitride G3 bead (Tsubaki-Nakashima [Georgia, USA]). Beads were blocked in 1% BSA 100 mM PIPES pH 6.50 and washed with 100 mM PIPES pH 6.50. The assay was performed in 96-well plates (Costar [New York, USA], catalog number 3916) using a FLUOstar Omega fluorometer (BMG [Ortenberg, Germany]). Plates were orbitally shaken (800 rpm for 1 min every 29 min at 37 °C). Results from the triplicates were considered input for a three-output probabilistic algorithm with sample labeling as “positive,” “negative,” or “inconclusive”, based on the parameters: Maximum fluorescence (Fmax), time to reach 50% Fmax (T50), slope, and the coefficient of determination for the fitting were calculated for each replicate using a sigmoidal equation available in Mars data analysis software (BMG). The time to reach the 5000 relative fluorescence units (RFU) threshold (TTT) was calculated with a user-defined equation in Mars^62^.

Consent to collect CSF samples was not successful for all the subjects, see Table 1 and Supplementary Table 4 for details.

Sample processing and storing followed a very strict protocol^29^. CSF samples were frozen in a period of less than 30 min and stored at −80 C° in aliquots for single use only. An electrical monitoring system ensures and guarantees a constant temperature, continuously documented and checked. For analysis, single use aliquots were shipped on dry ice.

### Neuronal α-synuclein-disease integrated staging system (NSD-ISS)

Neuronal αSyn-disease is defined by the biological anchors S: presence of in vivo detected pathologic αSyn species, measured usually by αSyn seeding aggregation assays, (independent from clinical syndrome) and D: dopaminergic neuronal dysfunction, assessed by DAT-SPECT, leading to a proposed NSD Integrated Staging System (NSD-ISS) that includes clinical signs and symptoms. Stages 0−1 are defined by the presence of pathogenic variants in SNCA gene (Stage 0), S alone (Stage 1 A), or S and D (Stage 1B) without clinical signs/symptoms. The occurrence of clinical manifestations defines the transition to Stage 2 and higher. Stage 2 includes subtle signs/symptoms without functional impairment. Stages 2B-6 require S and D to be positive and for there to be stage-specific increases in functional impairment^31^. NSD-ISS was applied in all 88 participants where αSyn-SAA and DAT-SPECT were available (Supplementary Table 5).

### Statistical analysis

All analyses were performed with the statistical software R (version 4.0.5). Baseline continuous variables were expressed as mean (standard deviation), median, and the range as given by the minimum and maximum values. Group comparisons were performed using the nonparametric Mann–Whitney Kruskall-Wallis test because some of the parameters had nonnormal distribution. For the binary variable “sex”, the count in each category is provided, and the Fisher exact Chi-square tests were used for comparison. Differential expression was assessed using the empirical Bayes approach as implemented in the Bioconductor limma package. Multiple

hypothesis testing corrections were performed by using Benjamini and Hochberg’s (BH) false discovery rate at a = 5%. Linear mixed models were used for longitudinal data analysis that allowed fitting only for random intercept models. They were implemented using the function *lmer* from the cran package *lmerTes*t. The correlation between the assessed proteins and the clinical parameters was assessed via a nonparametric Spearman coefficient using the base R function *cor.test* from the *cran psych package*. Here again, the BH procedure was used to correct for multiplicity. To ensure a capture of the performance of every single marker in the longitudinal cohort in this first assessment of this panel in longitudinal de novo PD, we decided to not perform a dimension reduction.

For machine learning, the Boruta algorithm from the CRAN package *Boruta* was used, and algorithms were built around the random forest classification algorithm. It aims to capture all the relevant features in the dataset concerning the outcome variables of PD versus healthy controls. The algorithm adds randomness to the dataset by creating shuffled copies of all features (Shadow Features) and trains a random forest classifier on the extended dataset (original attributes plus shadow attributes), applying a feature importance measure (The Mean Decrease Accuracy), evaluating the importance of each feature. At every iteration, the Boruta algorithm checks whether a real feature is more important than others and removes features that are marked as highly unimportant. As a stopping rule, we used 100,000 iterations with a maximum of 500 random forests as indicated by the parameter maxRuns in the Boruta function. With actual CRAN implementation of Boruta, warm-up rounds are removed, and the multiple testing corrections are introduced, marking all features that are either strongly or weakly relevant to PD diagnosis.

Handling of missing data

The amount of missing data was small with *n* = 133 of overall *n* = 14421 measurements (0.92% of the missing cases) with less than 10% of the peptide show missing values. Most contributor was the protein LAMP2, especially on the last plate, thus it was excluded, concerning *n* = 35*2 peptides. The QCs can be found in Supplementary Table 1.

Supplementary Fig. 2 provide heat maps of the missing values across all time points and diagnosis.

*Limma* handles missing values like other linear model functions in R (missing at random (MAR)). For each peptide in the data matrix, the cases with missing values are removed from the data and the design matrix and the linear model is fitted to the non-missing values. If a particular regression coefficient cannot be estimated from the observed data for a particular peptide, then an *NA* value will be returned for that.

The Random forests (RF) use median imputation for missing non categorial variables. This has been proven as an effective imputation technique for MAR with a moderate amount of missing values^63^.

### Standard protocol approvals, registrations, and patient consent

Approval was received from the local ethical standards committee on human experimentation for all human participants in all cohorts (FF 89/2008, FF 130/2012, MC 310/2010). Written informed consent for research was obtained from all study participants. DeNoPa is registered in the German Register for Clinical trials (DRKS00000540),

### LC-MS/MS analysis

In summary, a mix of heavy standard peptides serving as an internal standard (JPT Peptide Technologies (Berlin, Germany; SpikeTides L). 25 µL (for concentration see Supplementary Table 1A) was added to 100 µL of CSF samples, which were then reduced, alkylated, digested, and desalted. The quantification was performed using liquid chromatography-tandem mass spectrometry (LC-MS/MS) with a micro-high-performance LC-MS/MS system (6495 Triple Quadrupole LC/MS system, Agilent Technologies) equipped with a Hypersil Gold reversed-phase column (100 × 2.1 mm, 1.9 µm particle size, Thermo Fisher Scientific). The spectra can be found in Supplementary Figure 5. For further details, see Supplementary Table 2, which describes the settings used. For detailed sample preparation, we refer to the following publications^26,28^.The method involved the measurement of a panel of 38 synaptic and lysosomal proteins as indicated in Table 2. The target selection and compilation of the panel is based on unbiased explorative quantitative proteomic approaches in AD and PD, that indicated the proteins as potential biomarkers for lysosomal and synaptic dysfunction in neurodegeneration. The panel was further refined after targeted application in different cohorts including PD^33,40,64,65^.

To monitor the assay’s performance, two different quality control (QC) samples comprising CSF pools were periodically injected, where one of them was used to adjust for potential plate differences (batches) and the second to evaluate the final analytical performance. Longitudinal samples from the same patients were run randomized on the same plates in order to minimize batch and run time variability. The analytical performance of the different proteins had a high precision within and between runs with a few exceptions (Supplementary Table 1A). Skyline 20.1 (MacCoss Lab Software) was utilized to analyze the mass spectrometric data. One peptide, the one with the best analytic performance, per target protein was selected for statistical analyses.

### Reporting summary

Further information on research design is available in the Nature Research Reporting Summary linked to this article.

### Supplementary information

Supplementary material

reporting summary

## Data Availability

All data generated or analysed during this study are included in this published article and its supplementary information files. Additional information can be provided on reasonable request. Patient samples can be provided to other researchers for certain projects after contact with and upon availability approval of the team in Kassel.
