# Author Correction: Lysosomal and synaptic dysfunction markers in longitudinal cerebrospinal fluid of de novo Parkinson’s disease

**DOI:** 10.1038/s41531-024-00726-x

**Published:** 2024-06-25

**Authors:** Michael Bartl, Johanna Nilsson, Mohammed Dakna, Sandrina Weber, Sebastian Schade, Mary Xylaki, Bárbara Fernandes Gomes, Marielle Ernst, Maria-Lucia Muntean, Friederike Sixel-Döring, Claudia Trenkwalder, Henrik Zetterberg, Ann Brinkmalm, Brit Mollenhauer

**Affiliations:** 1 Department of Neurology, University Medical Center Goettingen, Goettingen, Germany; 2 Institute for Neuroimmunology and Multiple Sclerosis Research, University Medical Center Goettingen, Goettingen, Germany; 3 Institute of Neuroscience and Physiology, The Sahlgrenska Academy at the University of Gothenburg, Mölndal, Sweden; 4 Paracelsus-Elena-Klinik, Kassel, Germany; 5 Institute of Diagnostic and Interventional Neuroradiology, University Medical Center Goettingen, Goettingen, Germany; 6 Department of Neurology, Philipps-University, Marburg, Germany; 7 Department of Neurosurgery, University Medical Center Goettingen, Goettingen, Germany; 8 Clinical Neurochemistry Laboratory, Sahlgrenska University Hospital, Mölndal, Sweden; 9 UK Dementia Research Institute at UCL, London, UK; 10 Department of Neurodegenerative Disease, UCL Institute of Neurology, London, UK; 11 Hong Kong Center for Neurodegenerative Diseases, Hong Kong, China

**Keywords:** Parkinson's disease, Parkinson's disease

Correction to: *npj Parkinson’s Disease* 10.1038/s41531-024-00714-1, published online 17 May 2024

In this article, the initials of author Johanna Nilsson were incorrectly written as ‘J.H.’ but should have been ‘J.N.’ in author contribution section. The original article has been corrected.

