# Supplementary material for: Lysosomal and synaptic dysfunction markers in longitudinal cerebrospinal fluid of de novo Parkinson’s disease
Source: NPJ Parkinsons Dis. 2024 May 17;10:102. doi: 10.1038/s41531-024-00714-1 (PMC11101466; doi:10.1038/s41531-024-00714-1)
Supplement: Supplementary file 1 — Supplementary material [file 41531_2024_714_MOESM1_ESM.pdf]

**Supplementary material for manuscript NPJPARKD-02485:**

# Lysosomal and synaptic dysfunction markers in longitudinal cerebrospinal fluid of de novo Parkinson's disease

Michael Bartl[1,2#], Johanna Nilsson[3] Mohammed Dakna[1], Sandrina Weber[1], Sebastian Schade[4], Mary Xylaki[1], Barbara Fernandes Gomes[3], Marielle Ernst[5], Maria-Lucia Muntean[4], Friederike Sixel-Döring[4,6], Claudia Trenkwalder[4,7], Henrik Zetterberg[,8,9,10,11], Ann Brinkmalm[8], Brit Mollenhauer[1,4]

[1] *Department of Neurology, University Medical Center Goettingen, Germany*
[2] *Institute for Neuroimmunology and Multiple Sclerosis Research, University Medical Center Goettingen, Germany*
[3] *Institute of Neuroscience and Physiology, The Sahlgrenska Academy at the University of Gothenburg, Mölndal, Sweden*
[4] *Paracelsus-Elena-Klinik, Kassel, Germany*
[5] *Institute of Diagnostic and Interventional Neuroradiology, University Medical Center Goettingen, Germany*
[6] *Department of Neurology, Philipps-University, Marburg, Germany*
[7] *Department of Neurosurgery, University Medical Center Goettingen, Robert-Koch Str. 40, 37075 Goettingen, Germany*
[8] *Clinical Neurochemistry Laboratory, Sahlgrenska University Hospital, Mölndal, Sweden*
[9] *UK Dementia Research Institute at UCL, London, United Kingdom*
[10] *Department of Neurodegenerative Disease, UCL Institute of Neurology, London, United Kingdom*
[11] *Hong Kong Center for Neurodegenerative Diseases, Hong Kong, China*

## Supplementary Figures

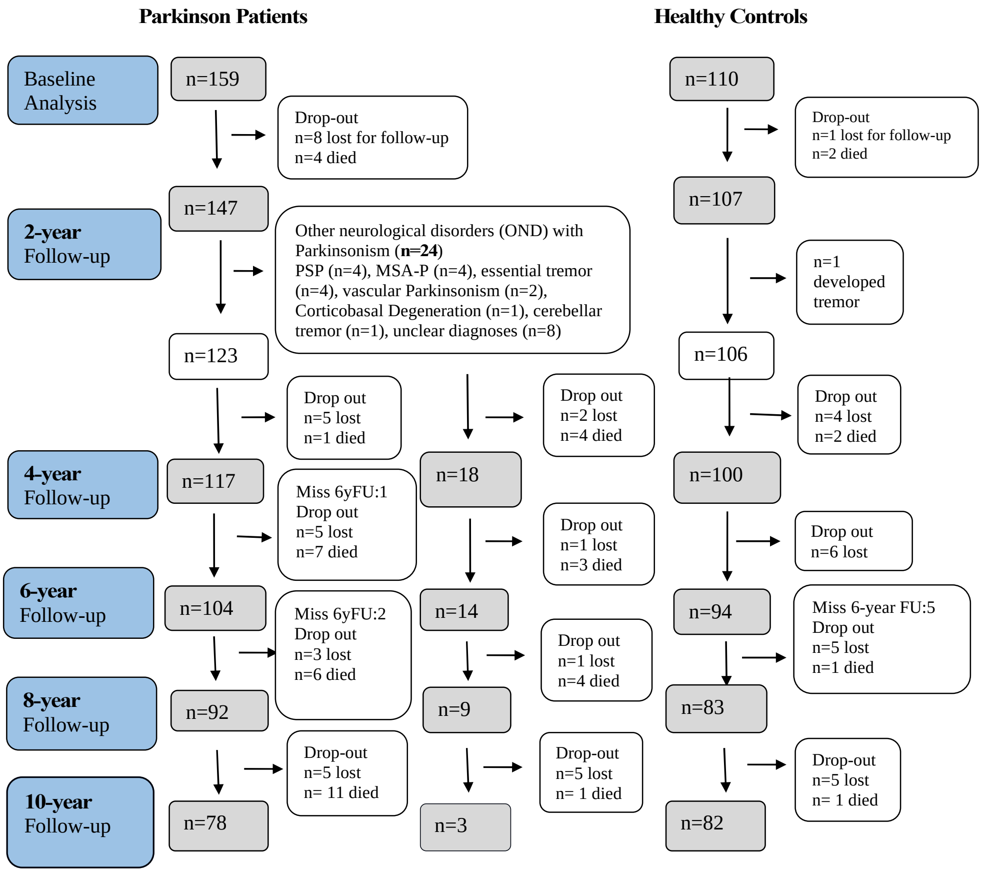


***Supplementary figure 1a**: Flow-chart of the DeNoPa cohort: Parkinson Patients and Healthy Controls*

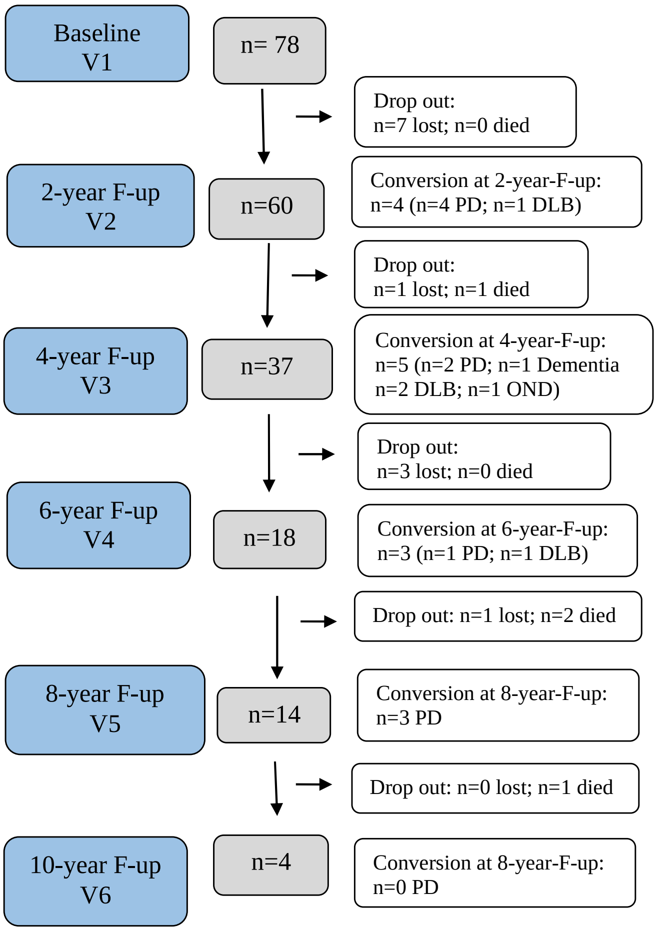


***Supplementary figure 1b:*** *Flow-chart of the iRBD cohort: isolated REM-sleep behavior disorder. The iRBD cohort is part of DeNoPa, a single center, longitudinal, observational study, still ongoing and recruiting iRBD subjects. Follow-up visits are conducted every two years for each single subject, explaining the differing numbers between the follow-up visits.*
*The figure displays the current numbers at the time point of publication. Dropouts are subjects that decided not to continue in the study*

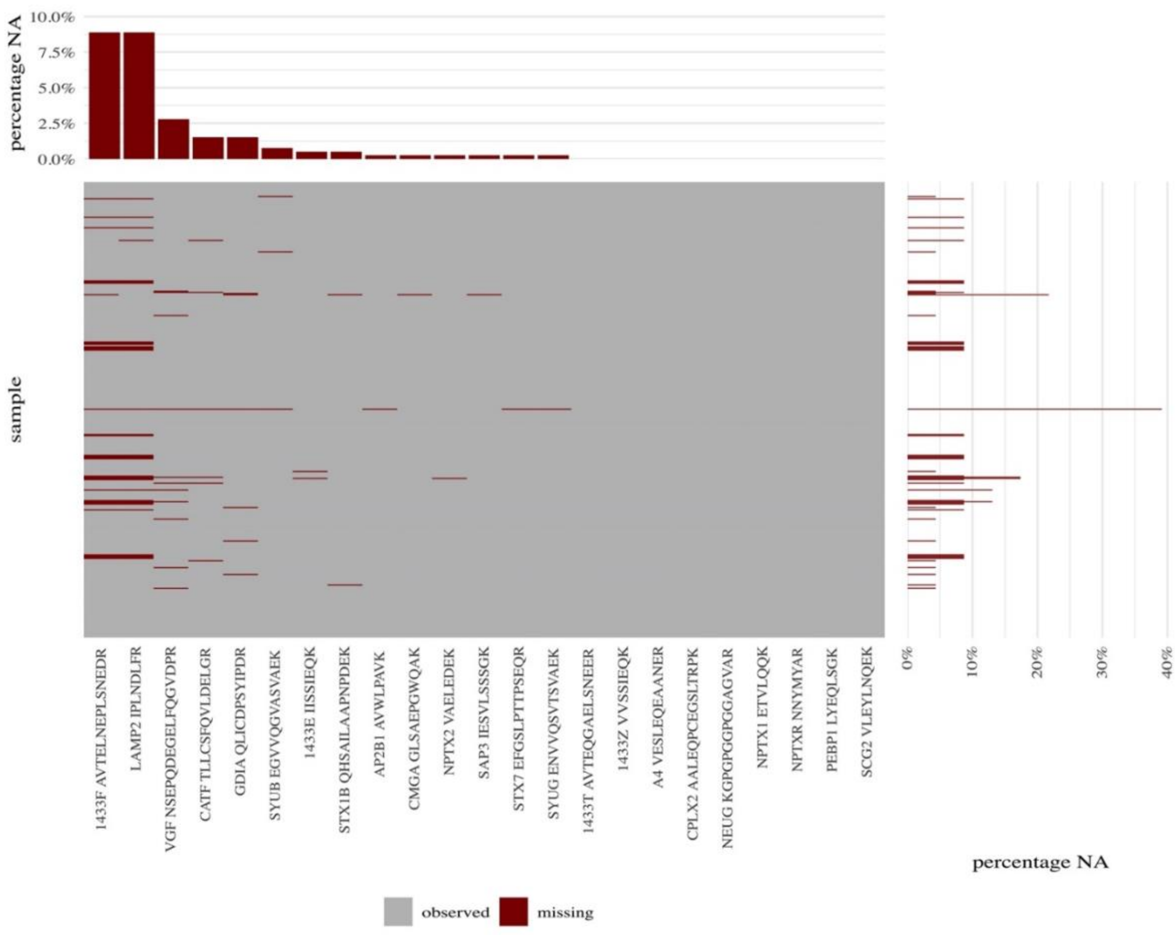


***Supplementary Figure 2:*** *Heat map displaying the percentage of missing data per peptide. Abbreviation: NA= not applicable*

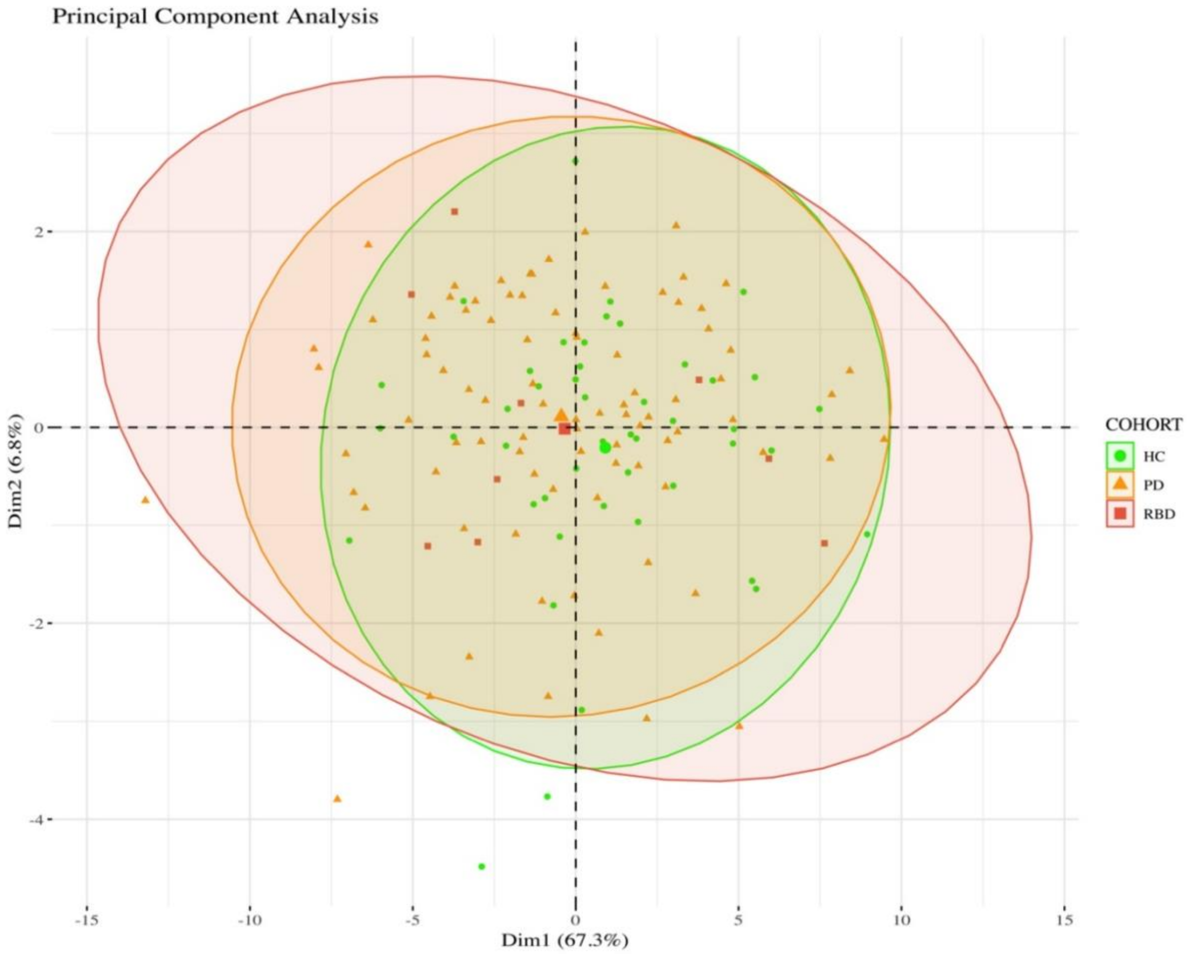


***Supplementary Figure 3:*** *Principal component analysis (PCA) showing, that the first component of the PCA explains 67% of the data variability, whereas the second component accounts for 7%.*

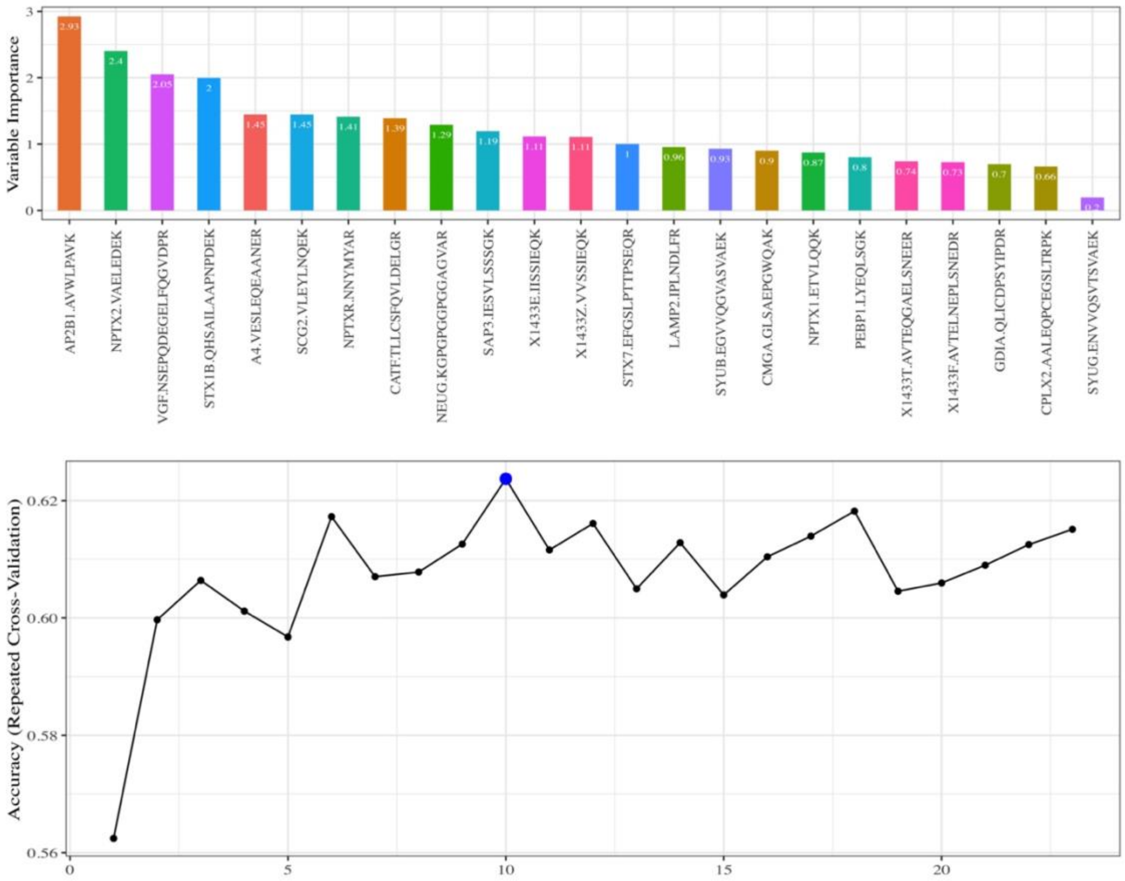


***Supplementary Figure 4a****: Results of Random forest classifier on the splitting of the baseline data set (88 PD/46 HC) based on 67% training data and 33% test data. Fivefold cross validation with 10 repetitions is used for building classifiers with 1 to 23 peptides. From the accuracy plot shows that a classifier with 10 peptides reaches the optimal accuracy of 0.622.*

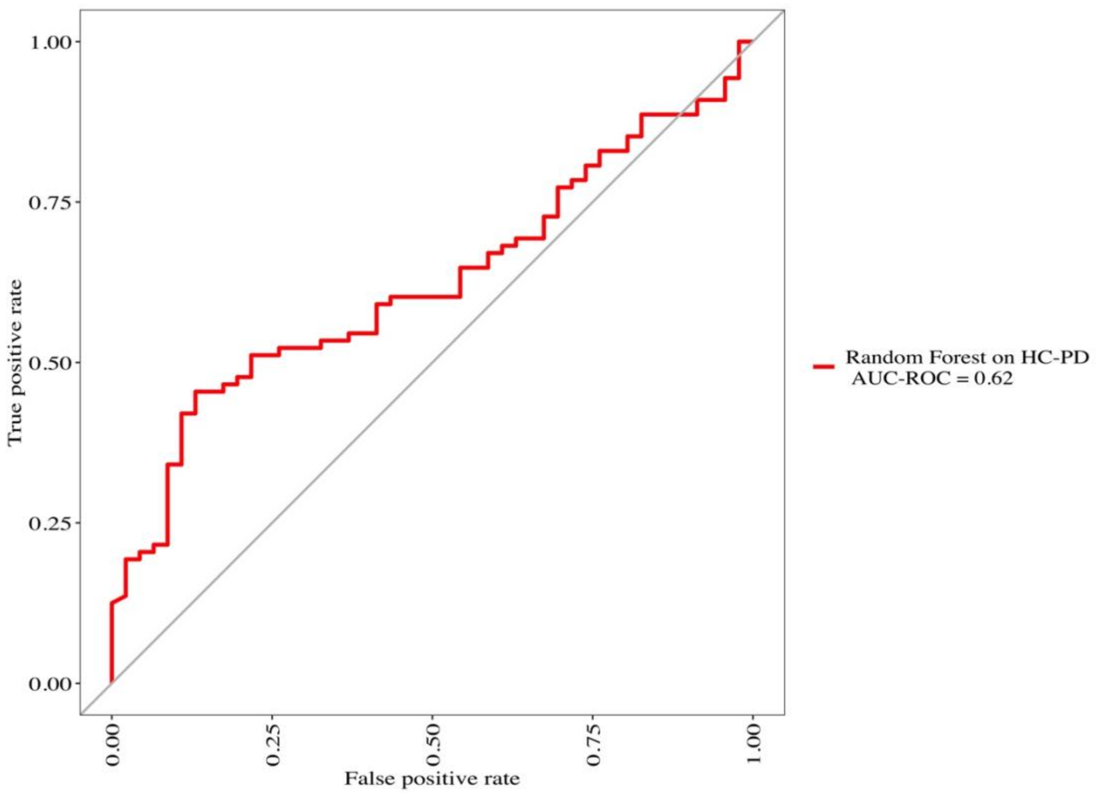


***Supplementary Figure 4b****: Results of Random forest classifier on the splitting of the baseline data set (88 PD/46 HC) based on 67% training data and 33% test data. Fivefold cross validation with 10 repetitions is used for building classifiers with 1 to 23 peptides. From the accuracy plot shows that a classifier with 10 peptides reaches the optimal accuracy of 0.622.*

*The Area Under the Curve of the model on the test set is of 0.62.*

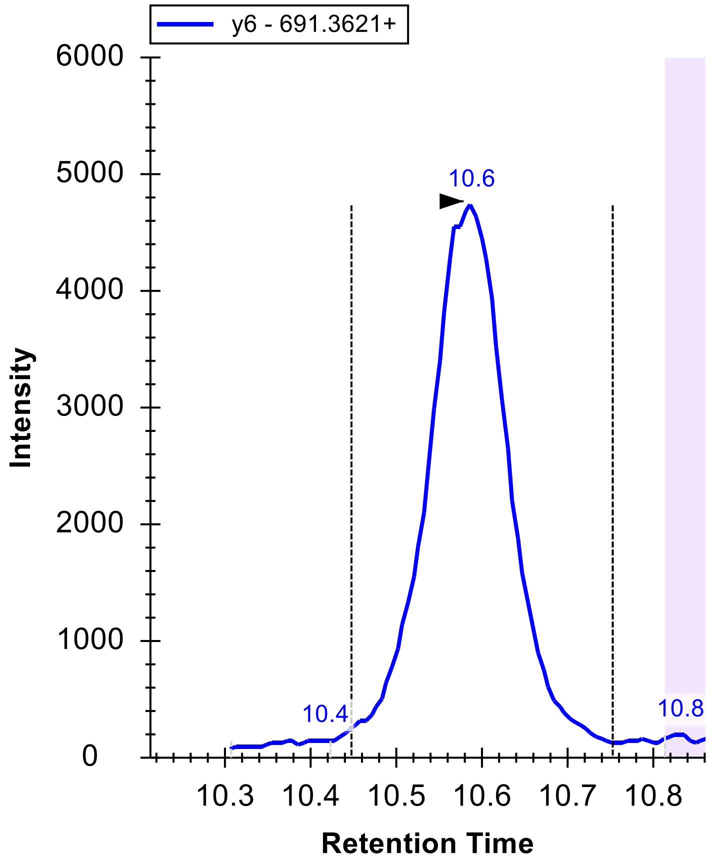


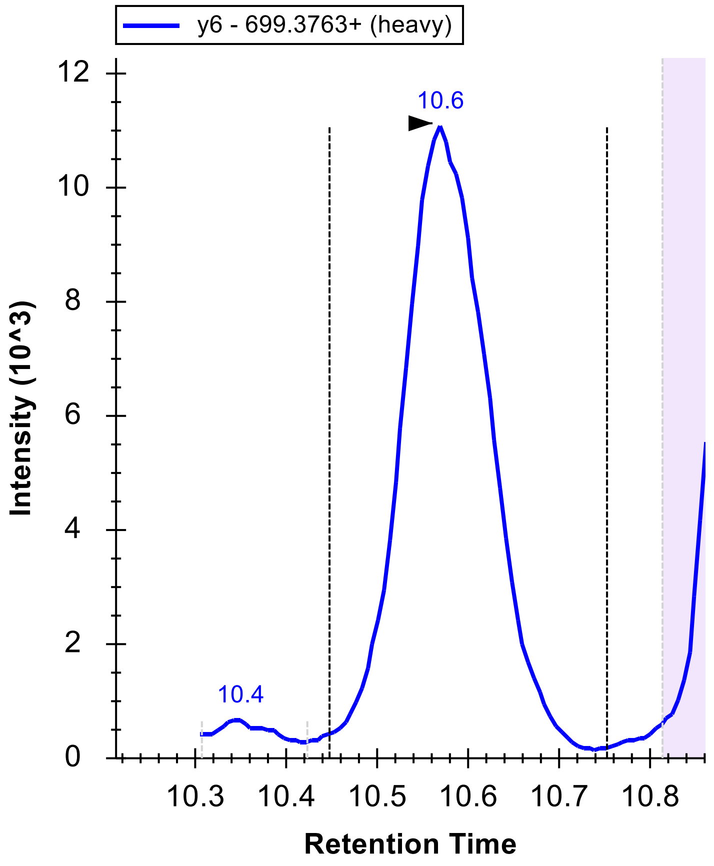


***Supplementary Figure 5a:*** *Spectrum 1433E*

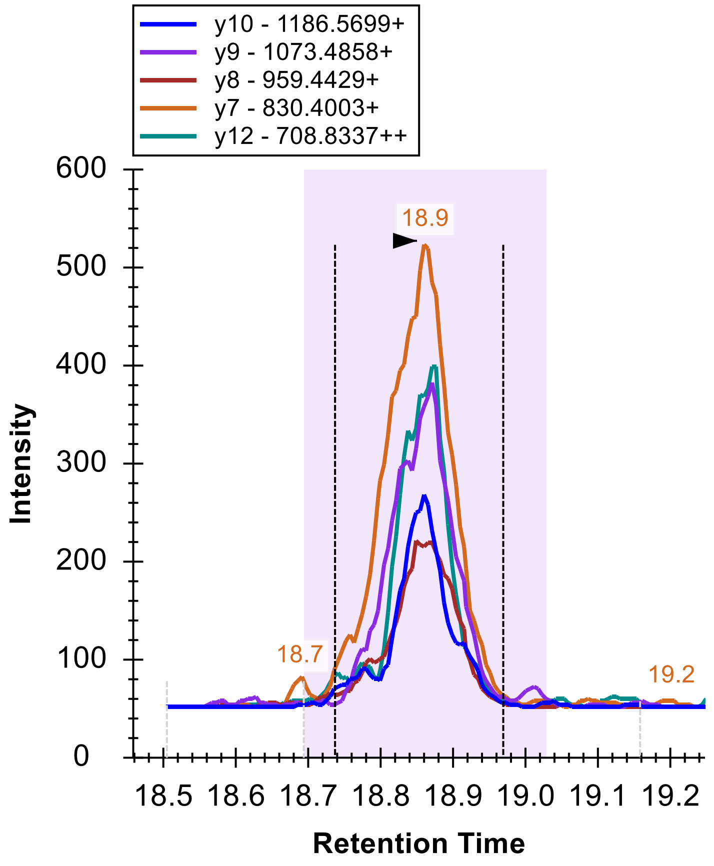


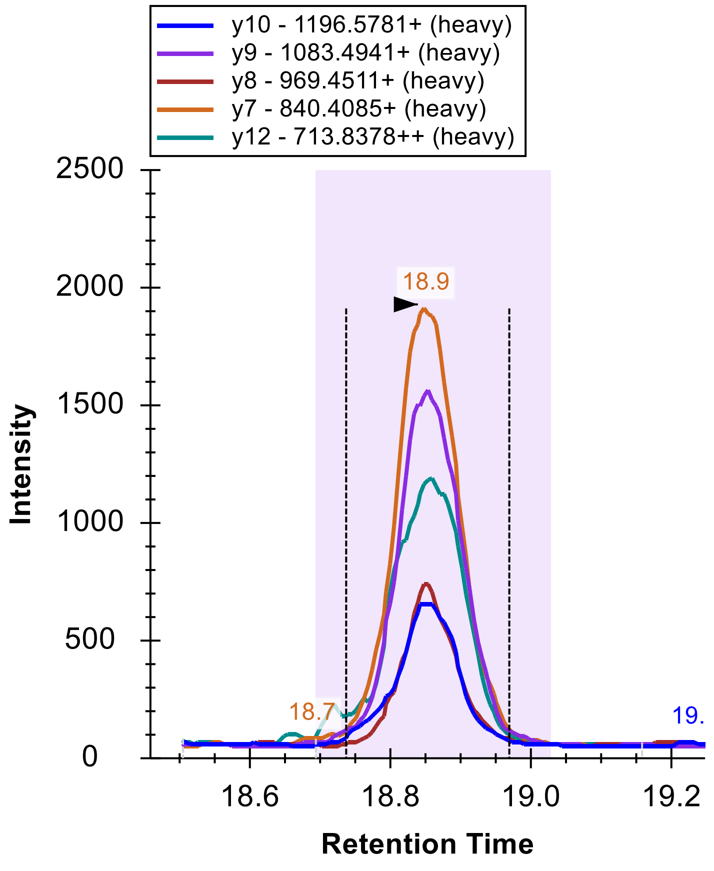


***Supplementary Figure 5b:*** *Spectrum 1433F*

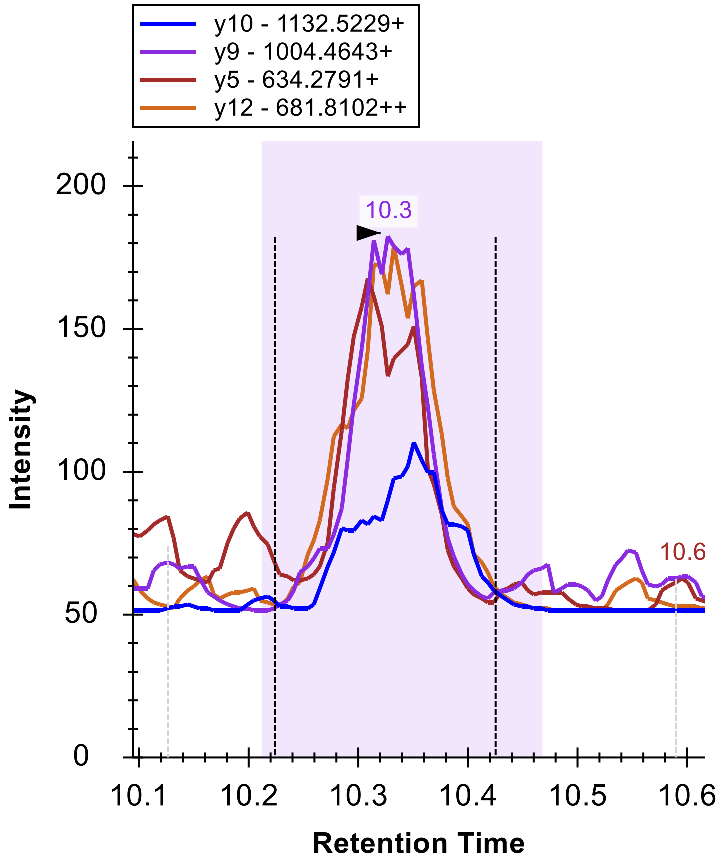


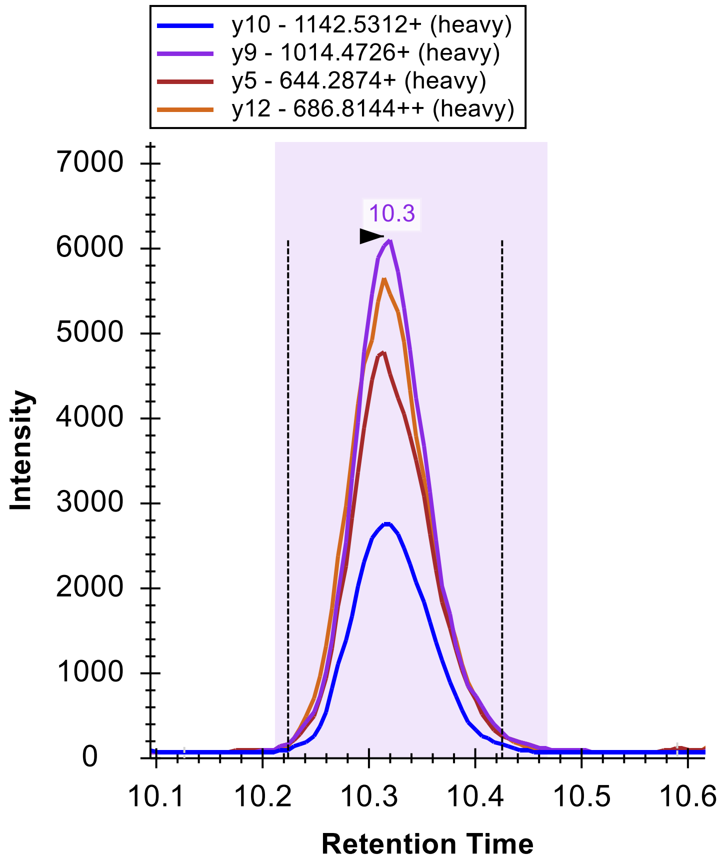


***Supplementary Figure 5c:*** *Spectrum 1433T*

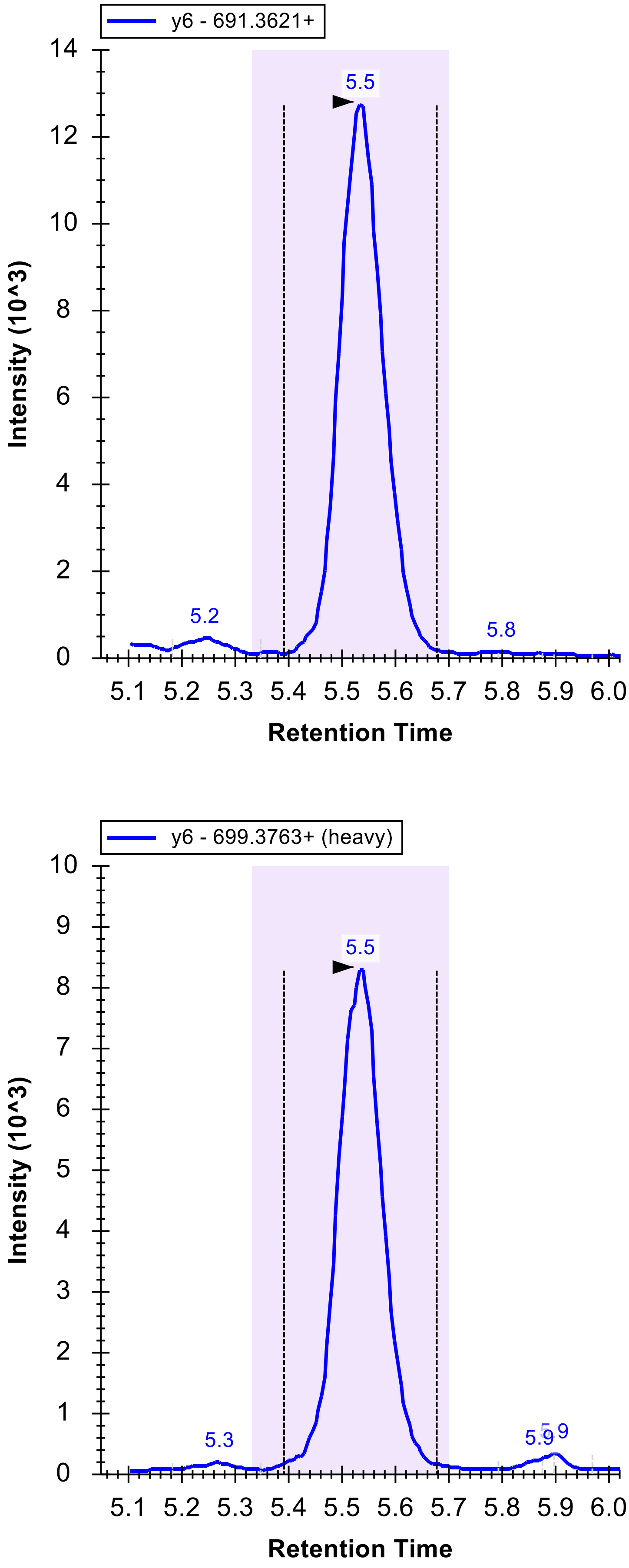


***Supplementary Figure 5d:*** *Spectrum 1433Z*

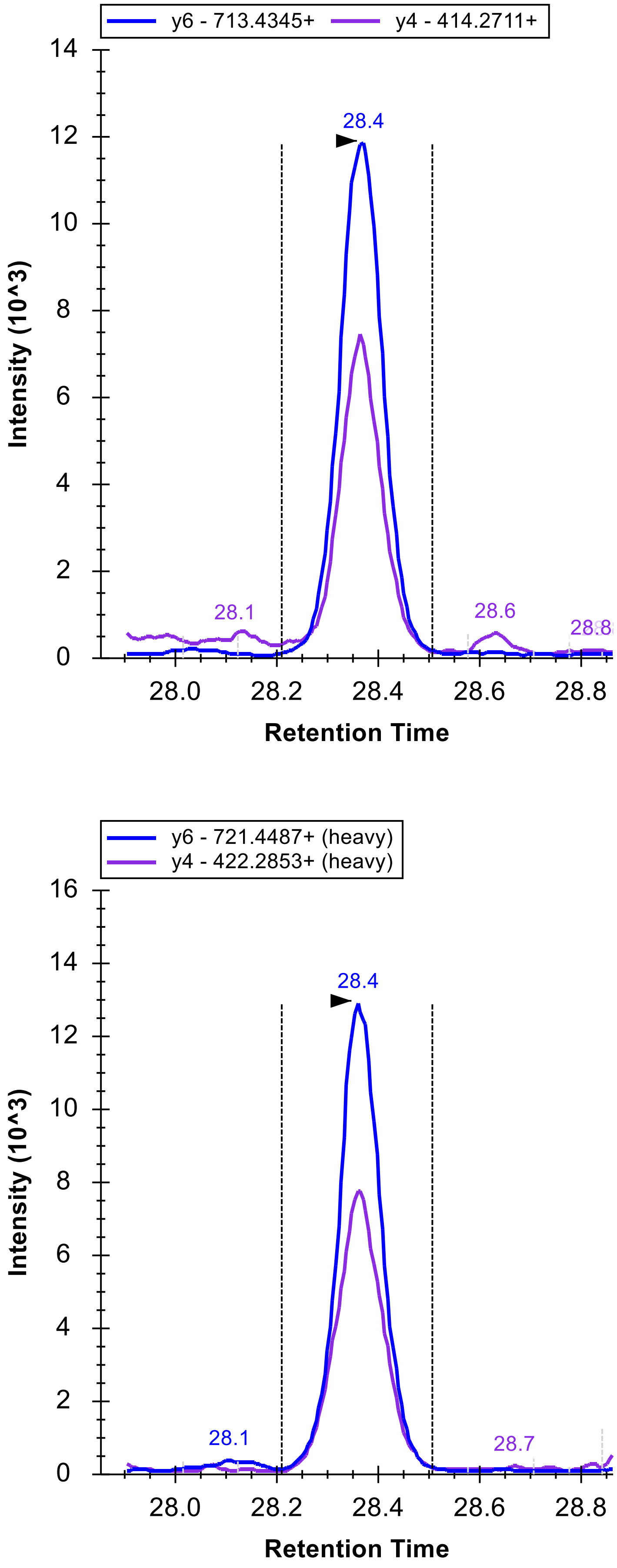


***Supplementary Figure 5d:*** *Spectrum AP2B1_AVW*

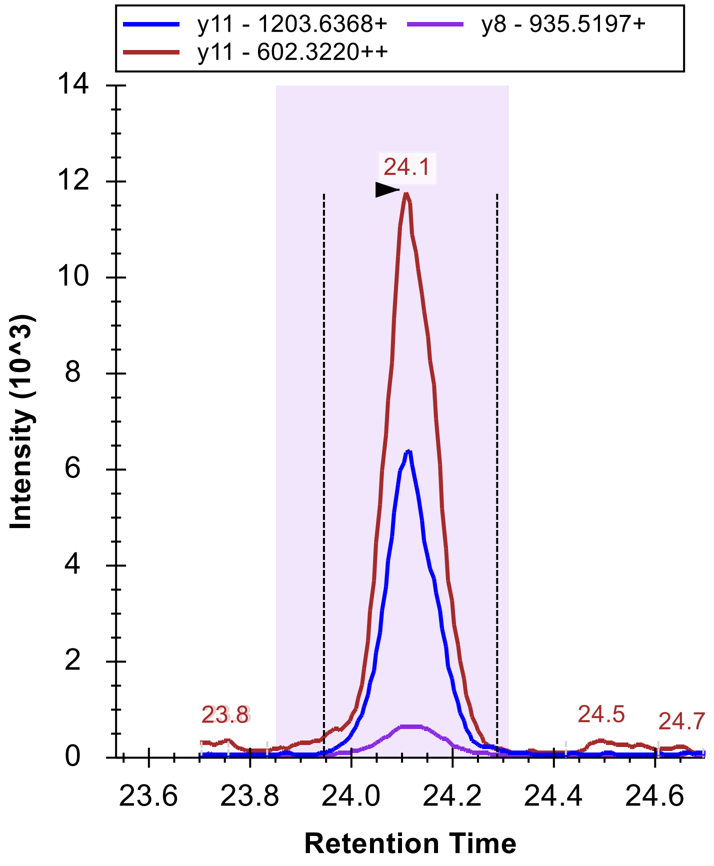


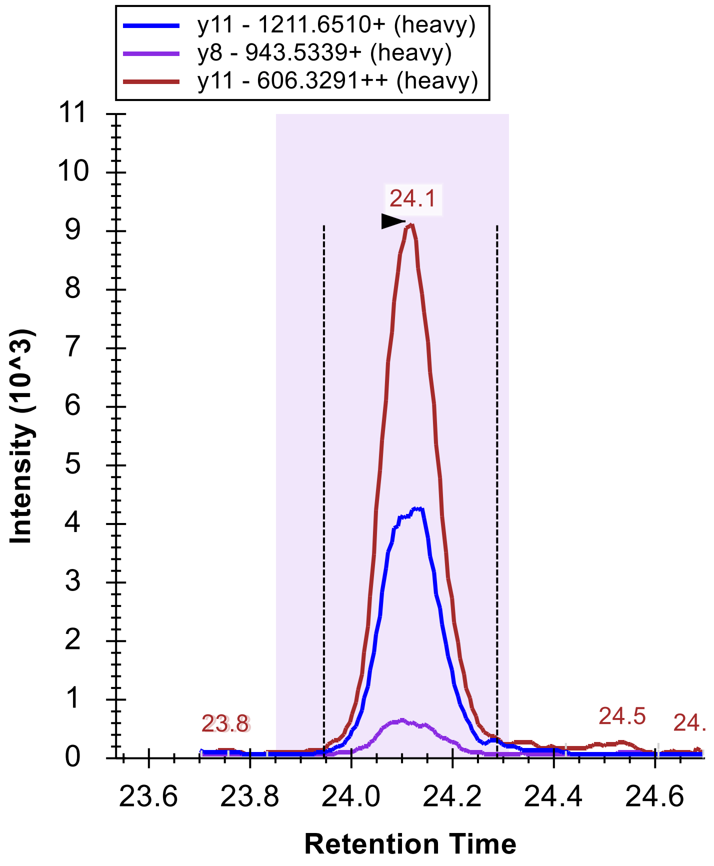


***Supplementary Figure 5e:*** *Spectrum AP2B1_IQPG*

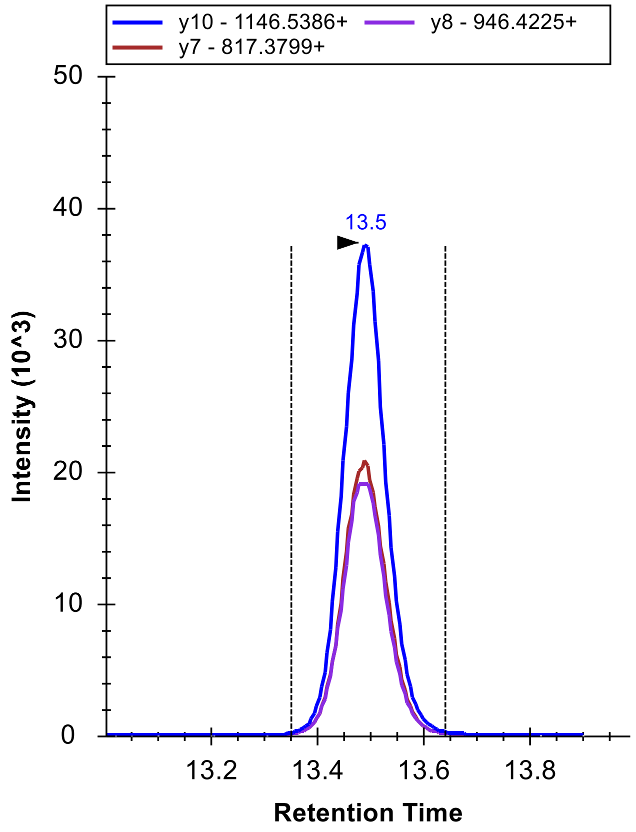


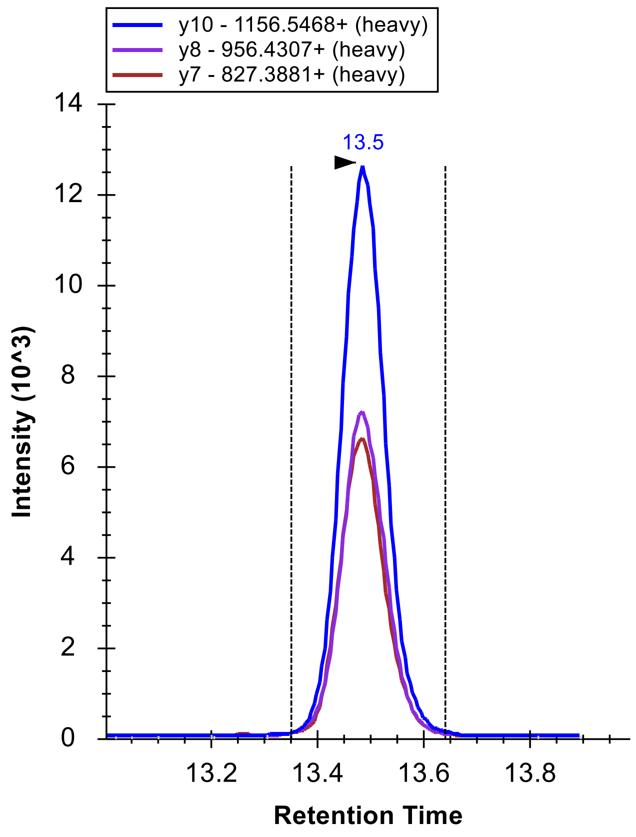


***Supplementary Figure 5f:*** *Spectrum APP*

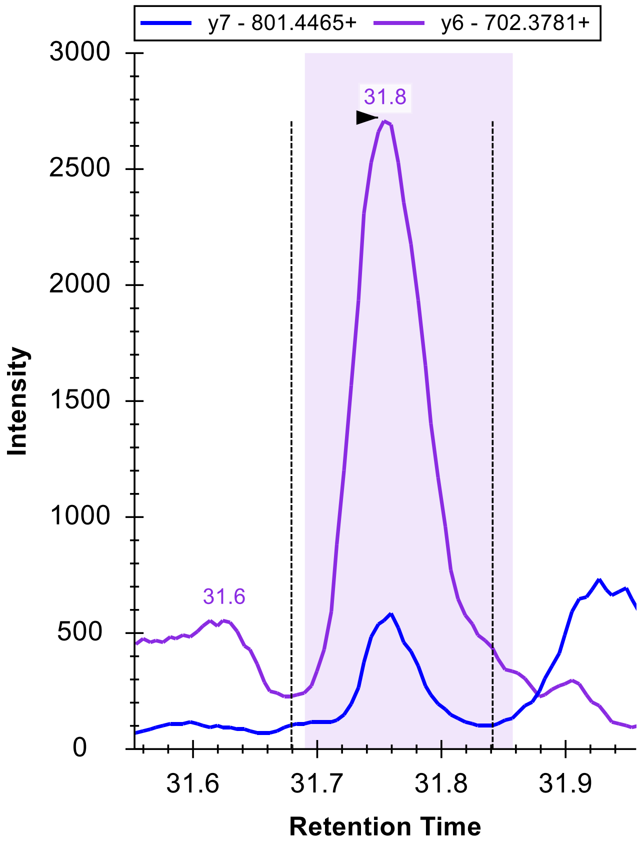


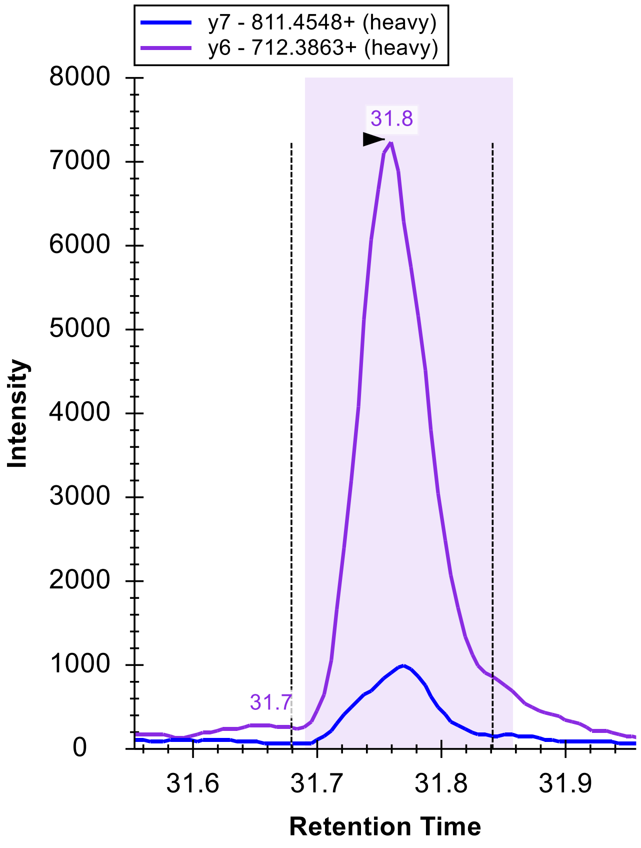


***Supplementary Figure 5g:*** *Spectrum CAT F*

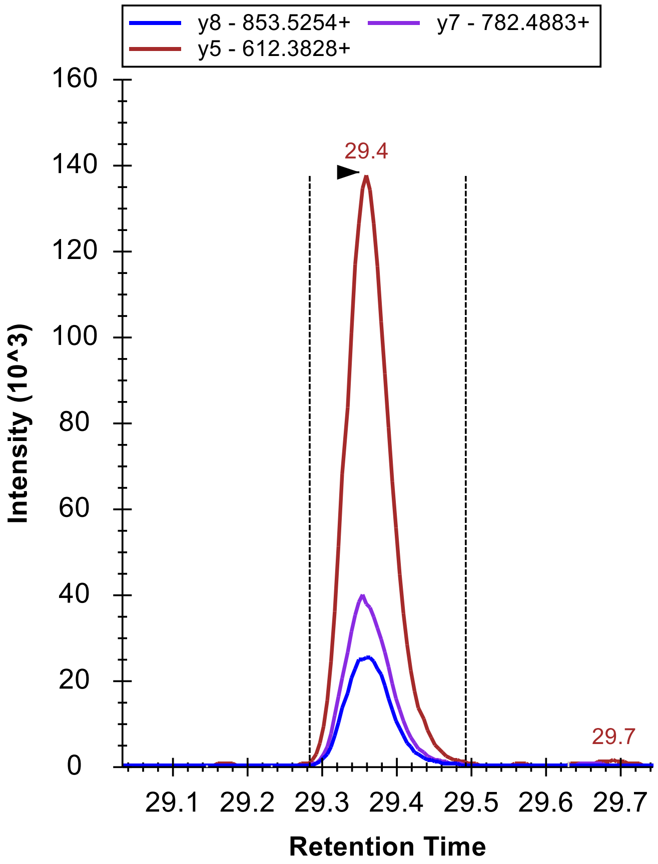


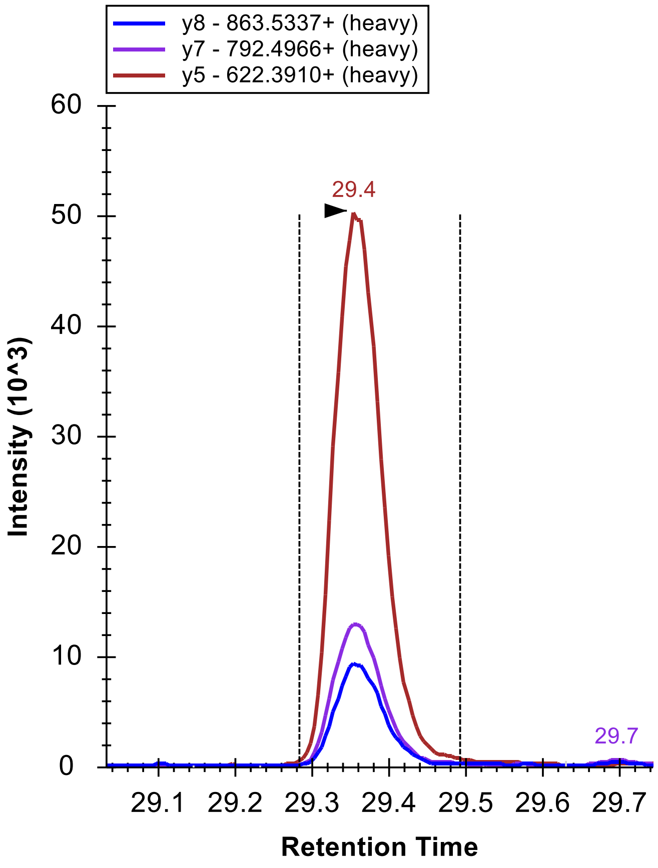


***Supplementary Figure 5h:*** *Spectrum CHMGA_EDSL*

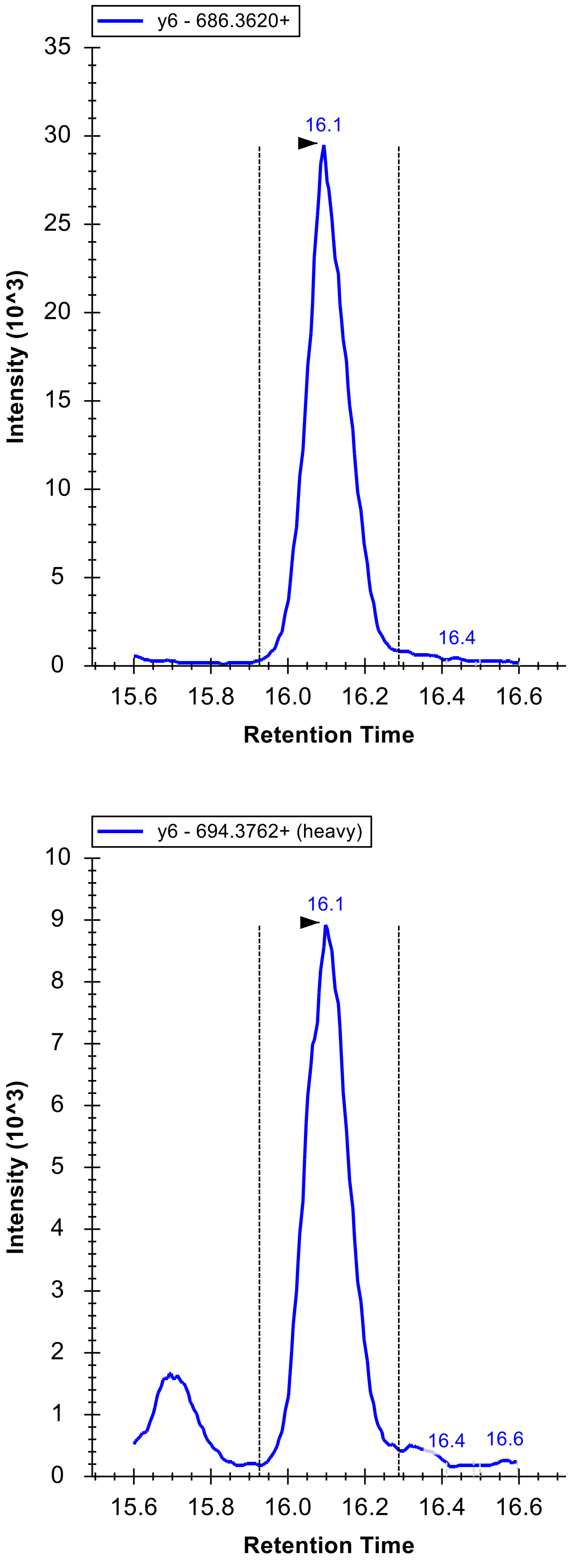


***Supplementary Figure 5i:*** *Spectrum CHMGA_GLAS*

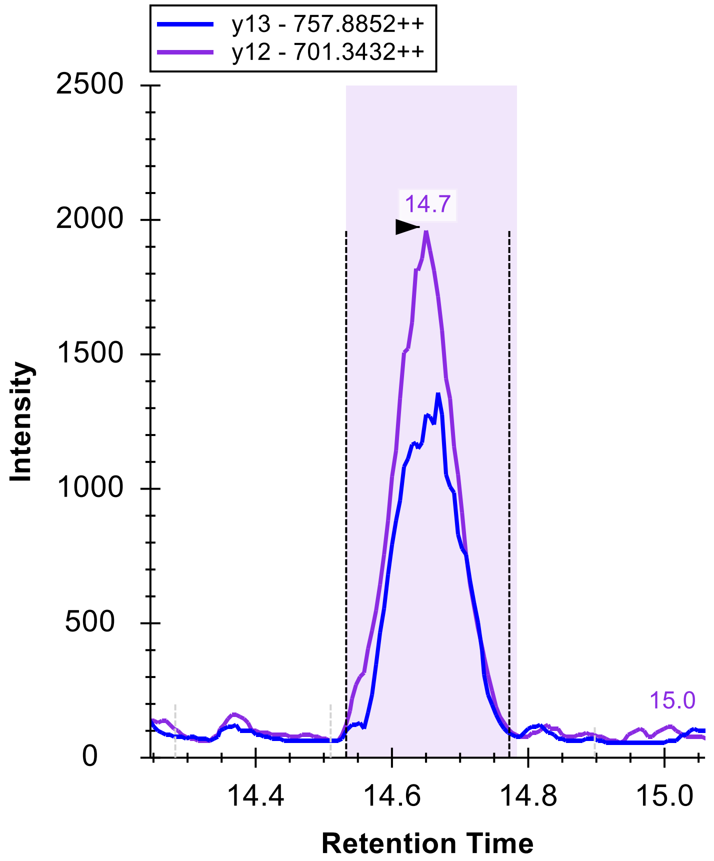


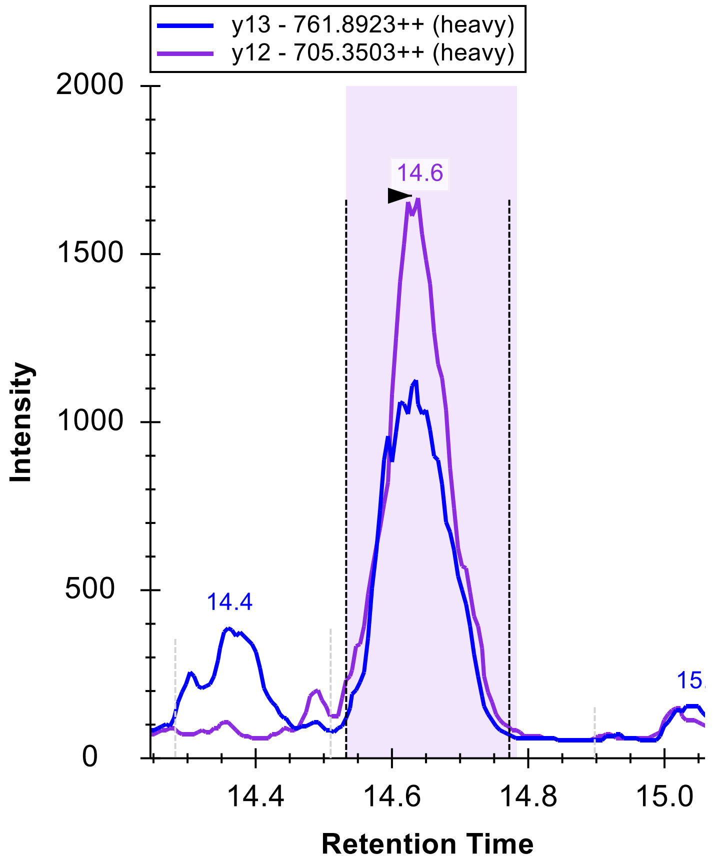


***Supplementary Figure 5j:*** *Spectrum CPLX2*

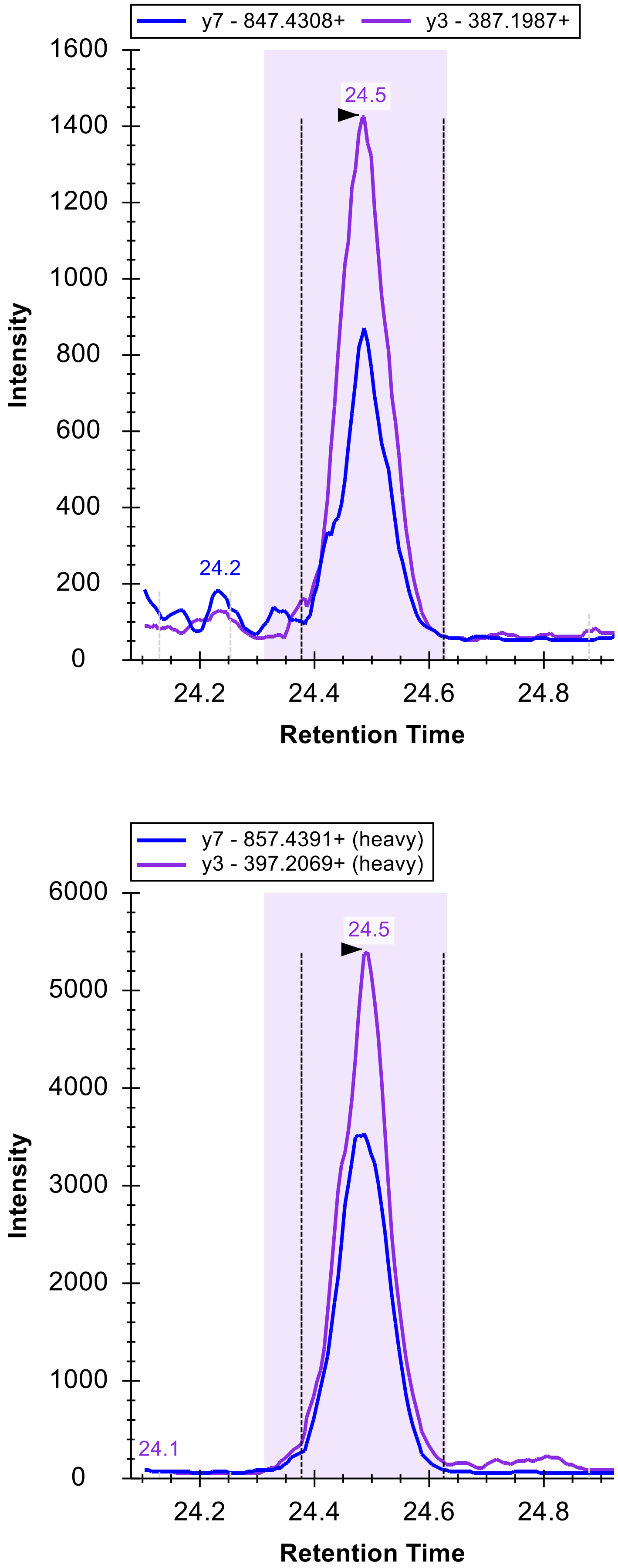


***Supplementary Figure 5k:*** *Spectrum GDIA*

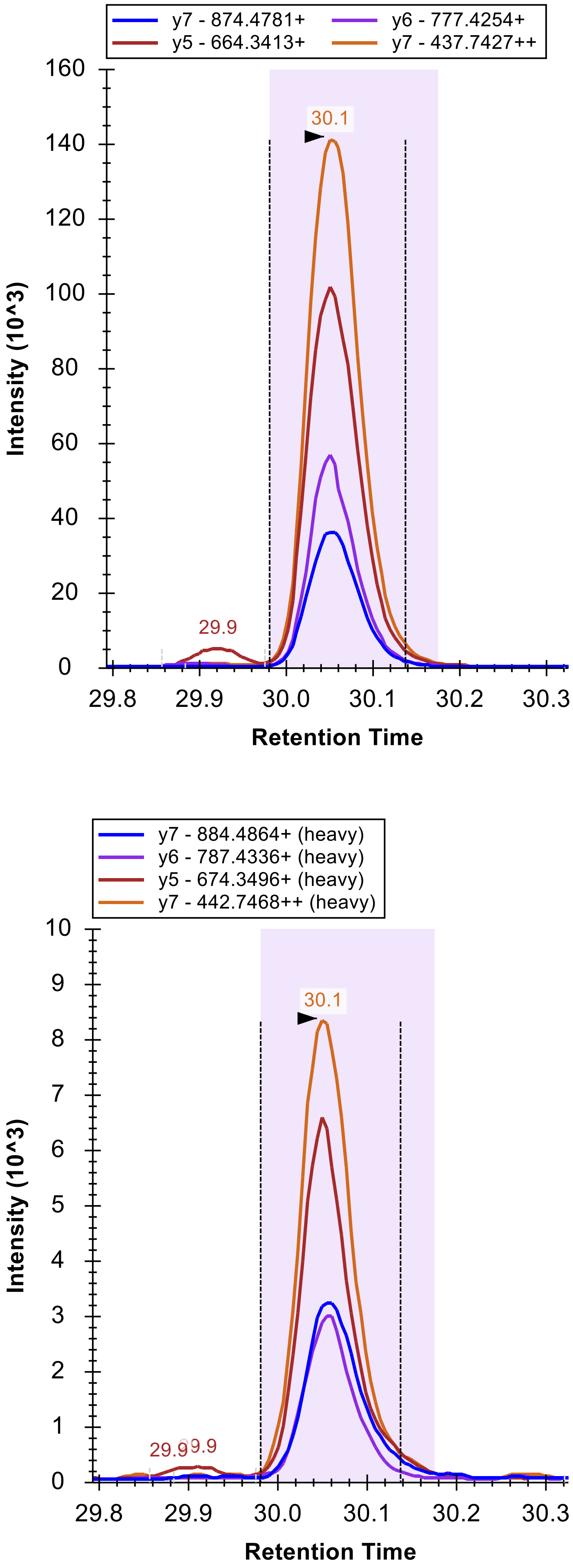


***Supplementary Figure 5I:*** *Spectrum LAMP2_IPL*

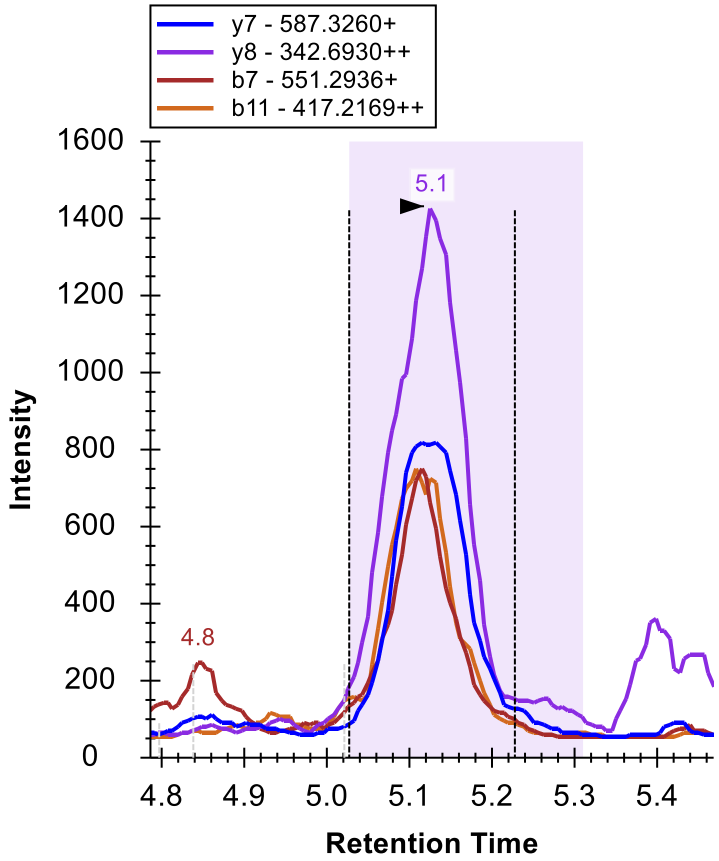


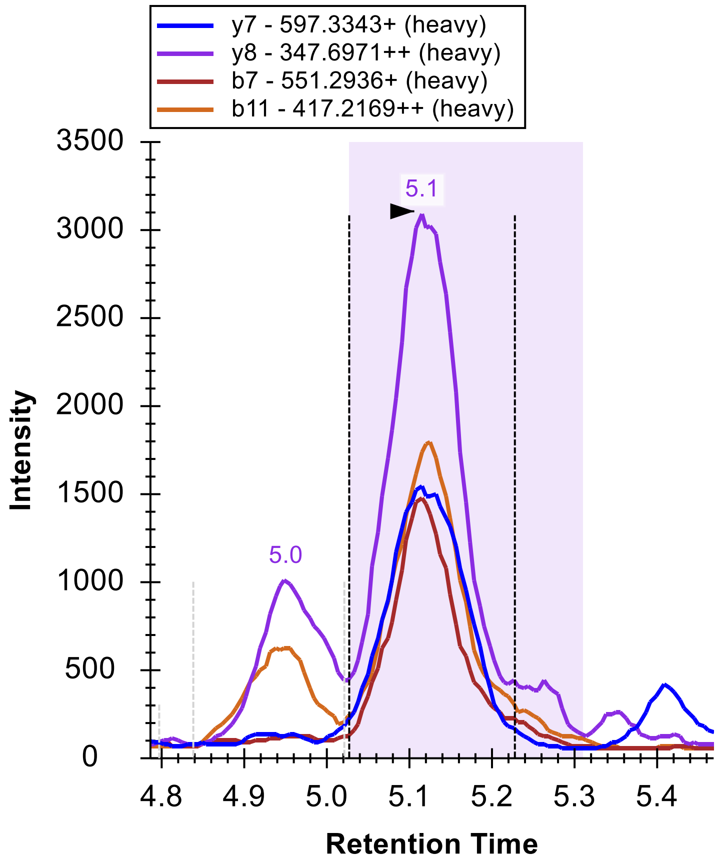


***Supplementary Figure 5m:*** *Spectrum NEUR*

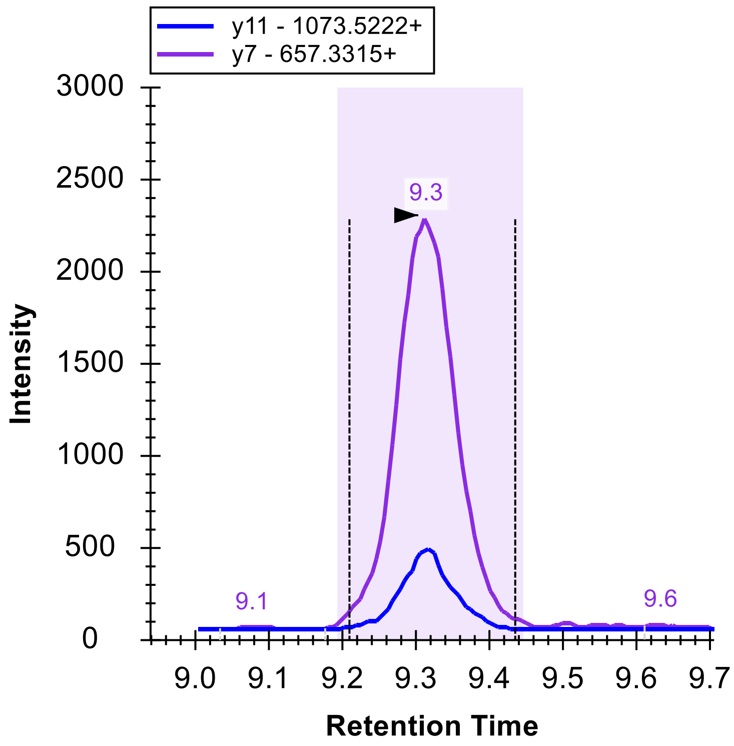


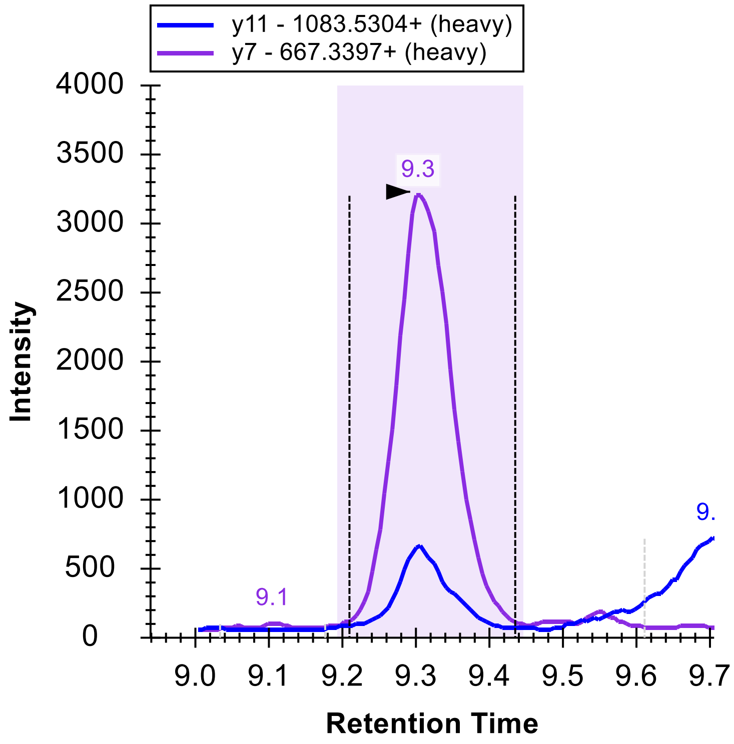


***Supplementary Figure 5n:*** *Spectrum NPTX1_CES*

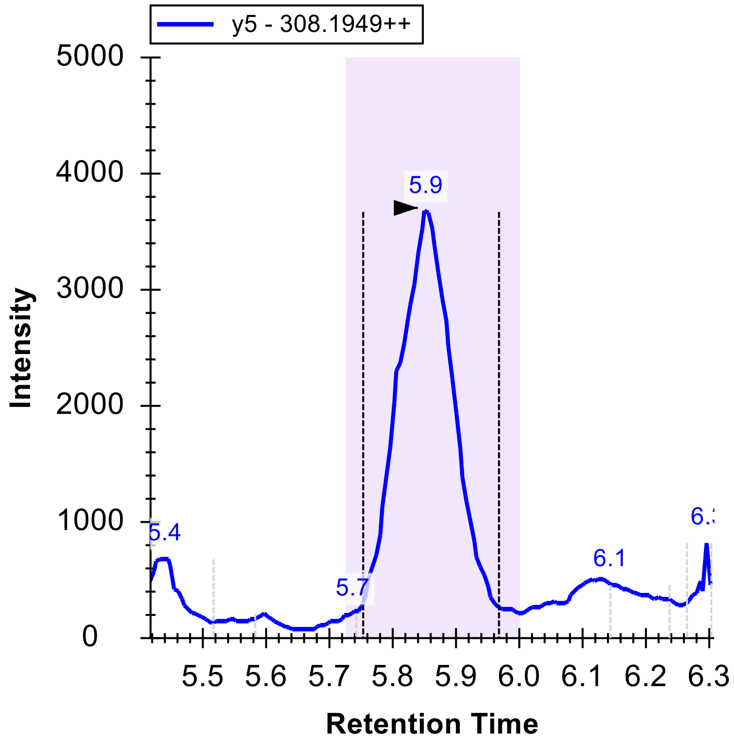


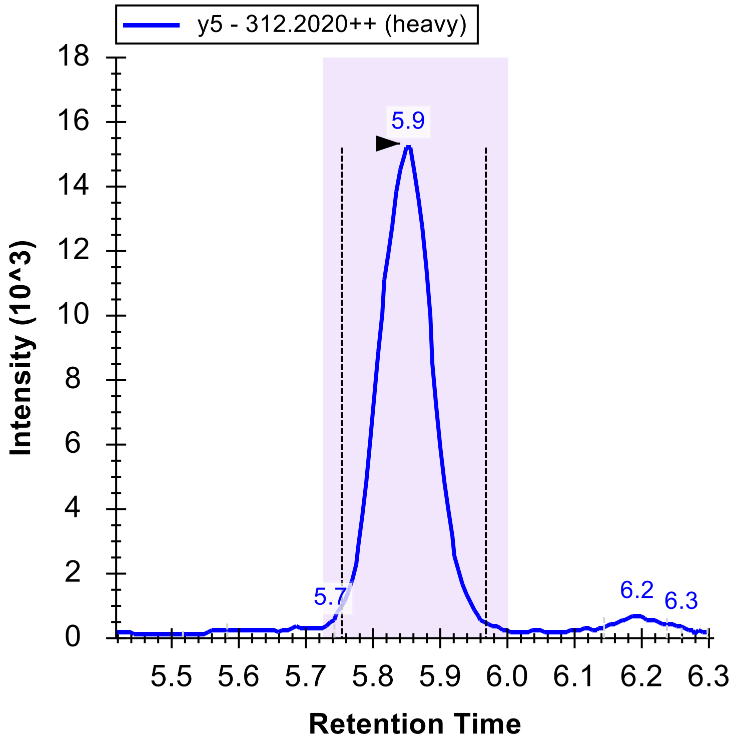


***Supplementary Figure 5o:*** *Spectrum NPTX1*

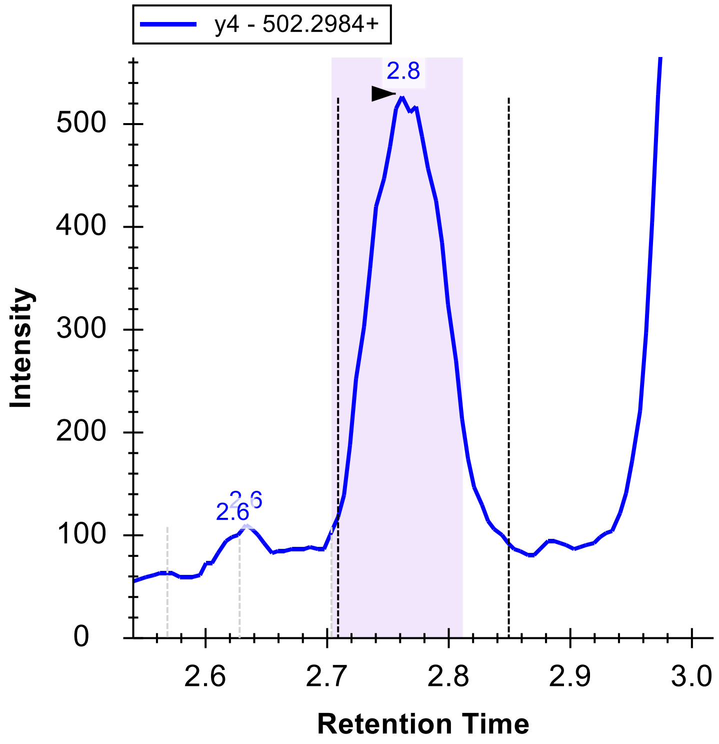


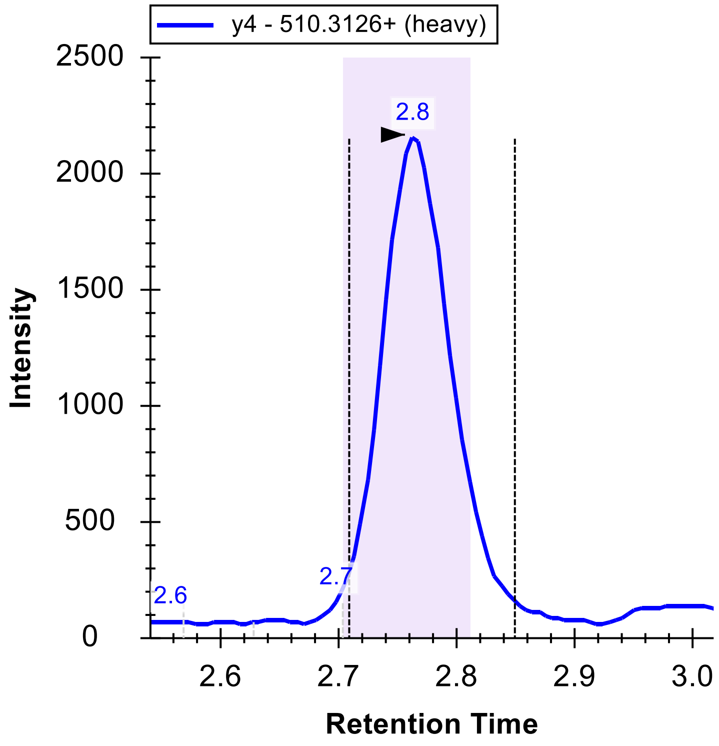


***Supplementary Figure 5p:*** *Spectrum NPTX2_ETVV*

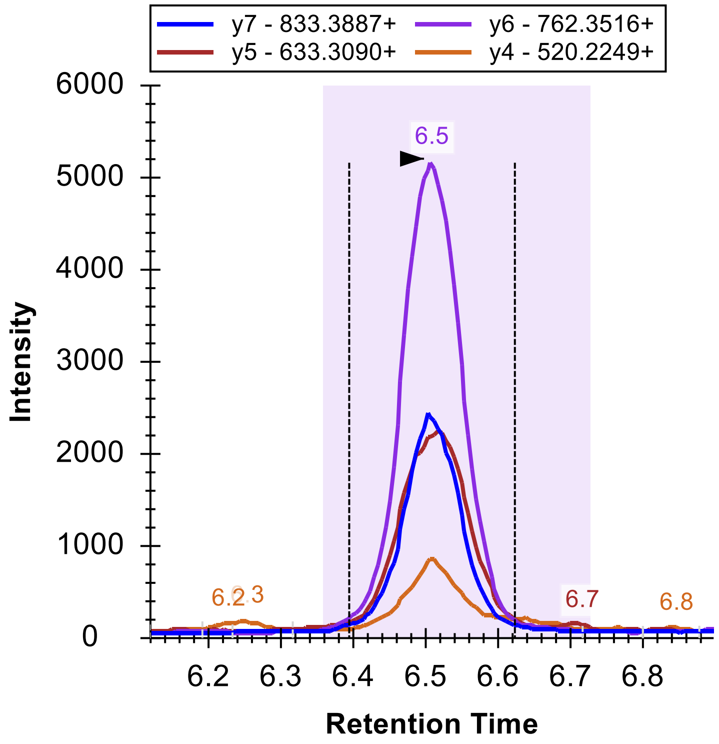


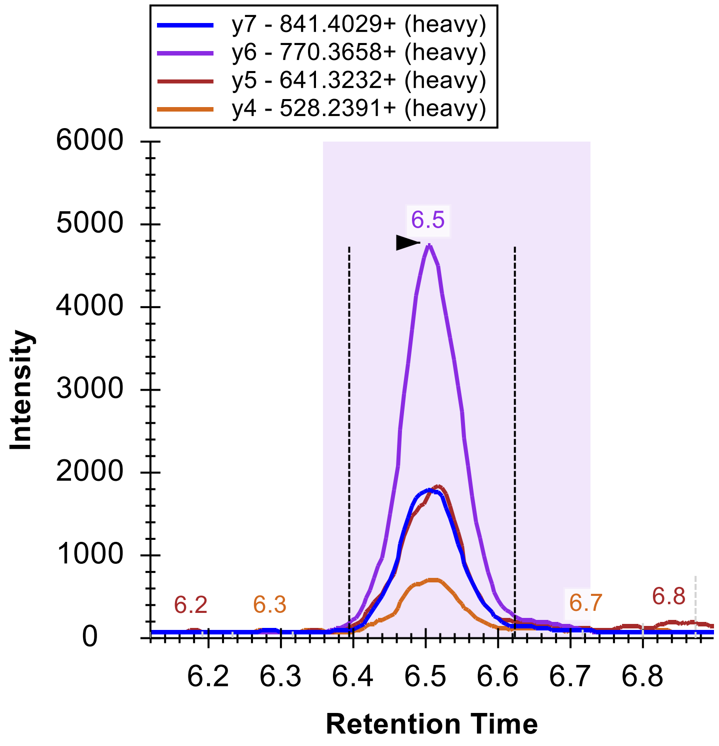


***Supplementary Figure 5q:*** *Spectrum NPTX2_VAEL*

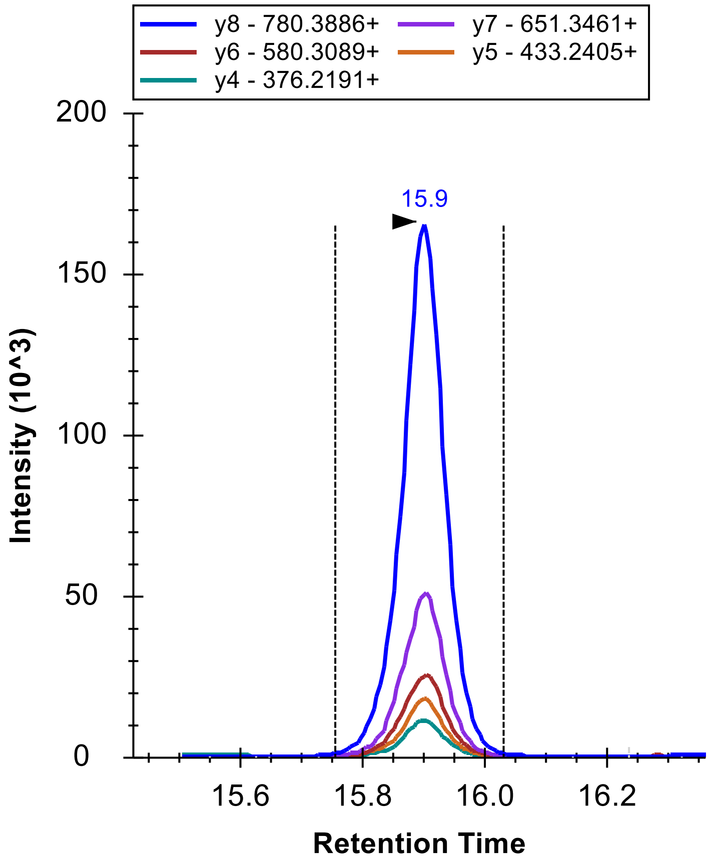


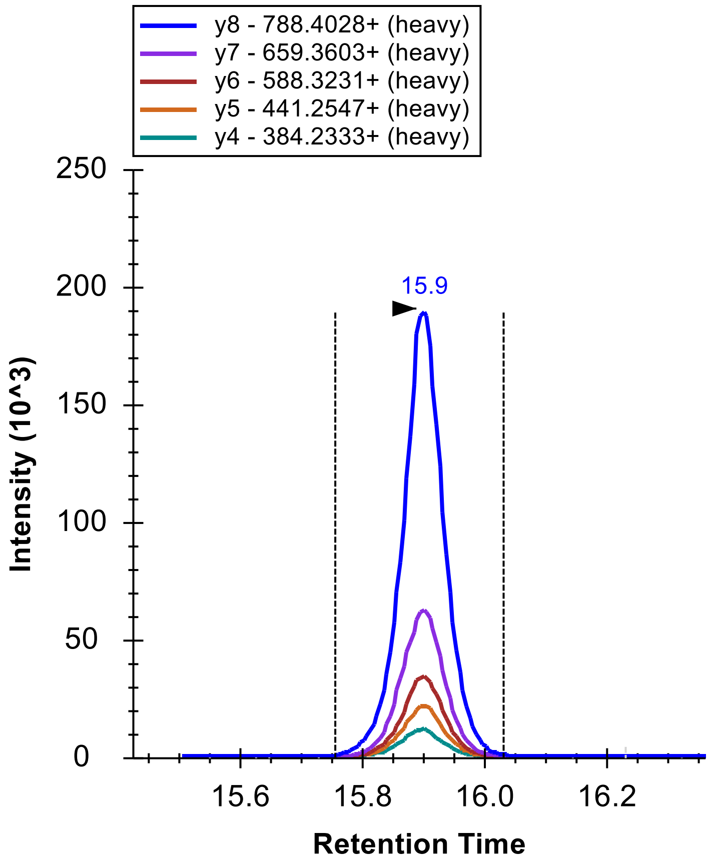


***Supplementary Figure 5r:*** *Spectrum NPTXR_LVEA*

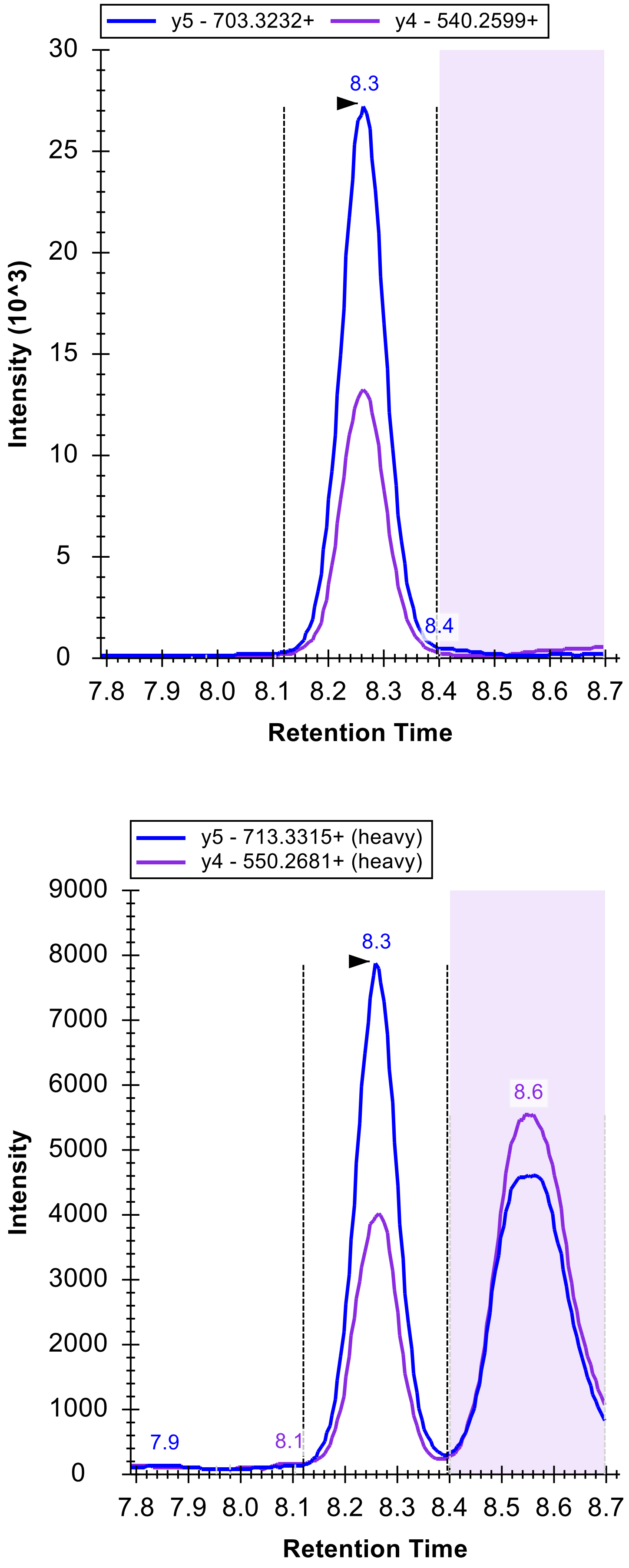


***Supplementary Figure 5s:*** *Spectrum NPTXR_NNY*

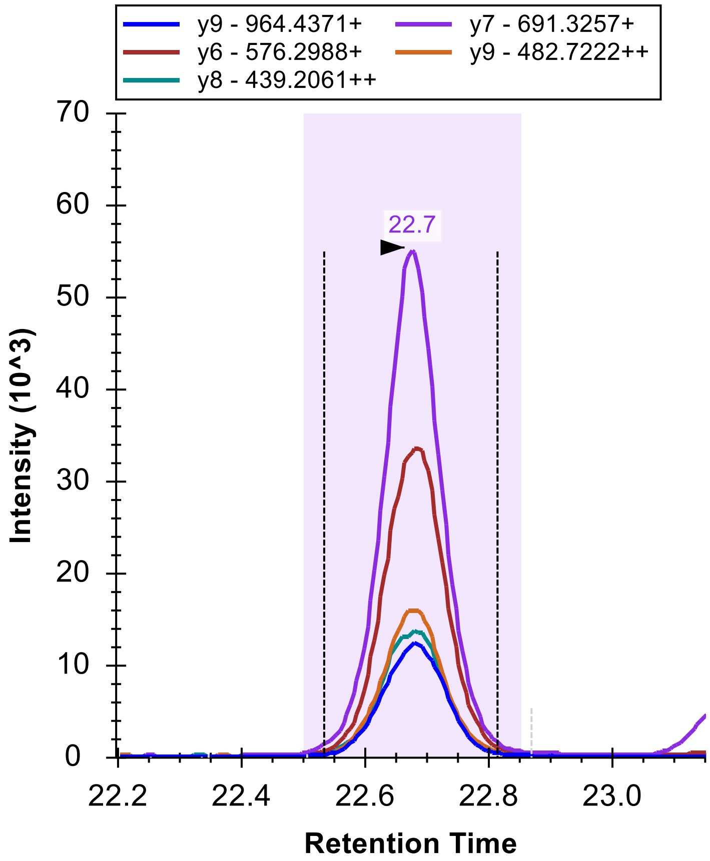


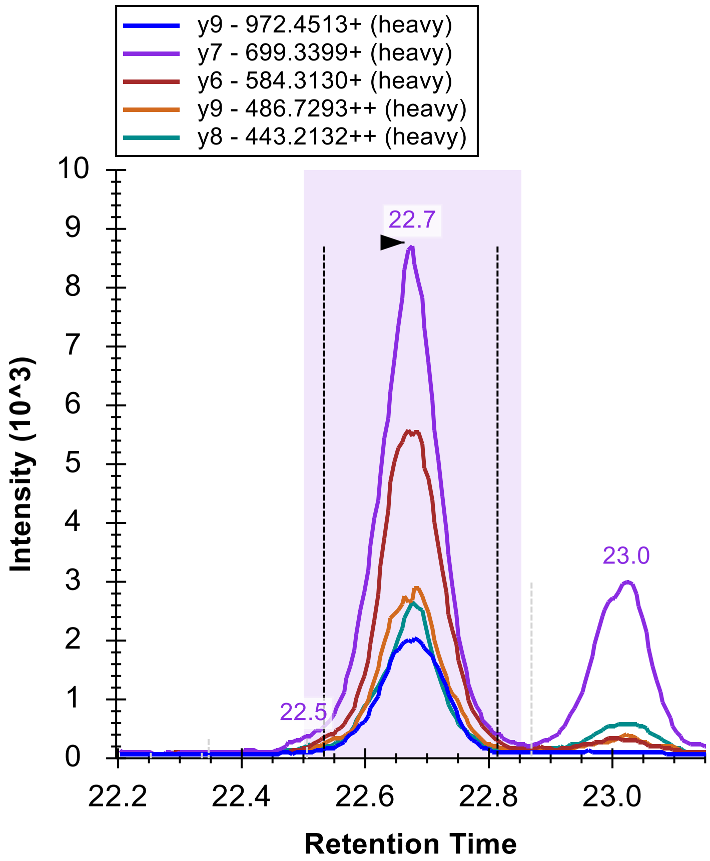


***Supplementary Figure 5t:*** *Spectrum PEBP1_LYE*

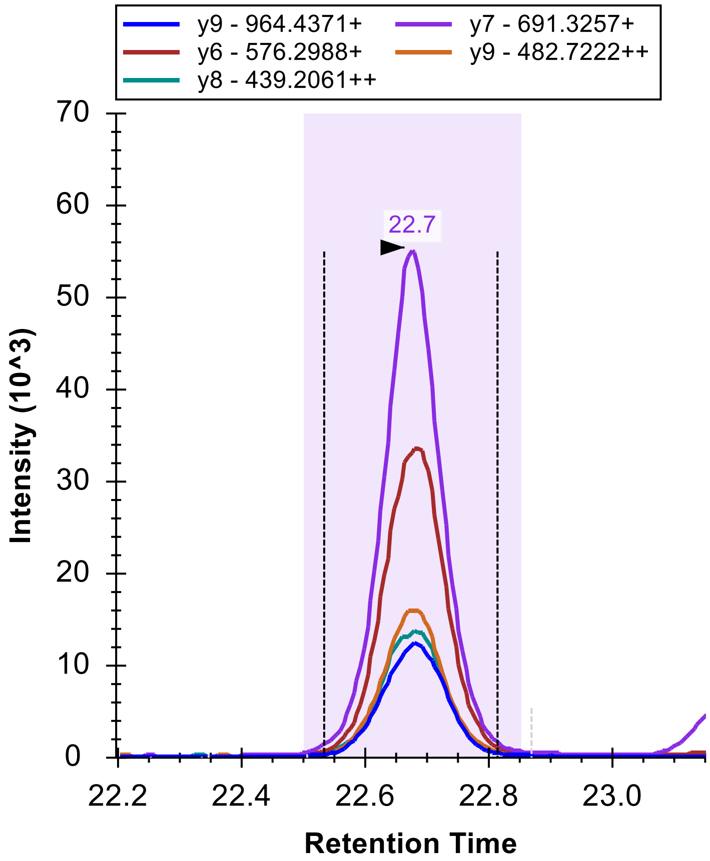


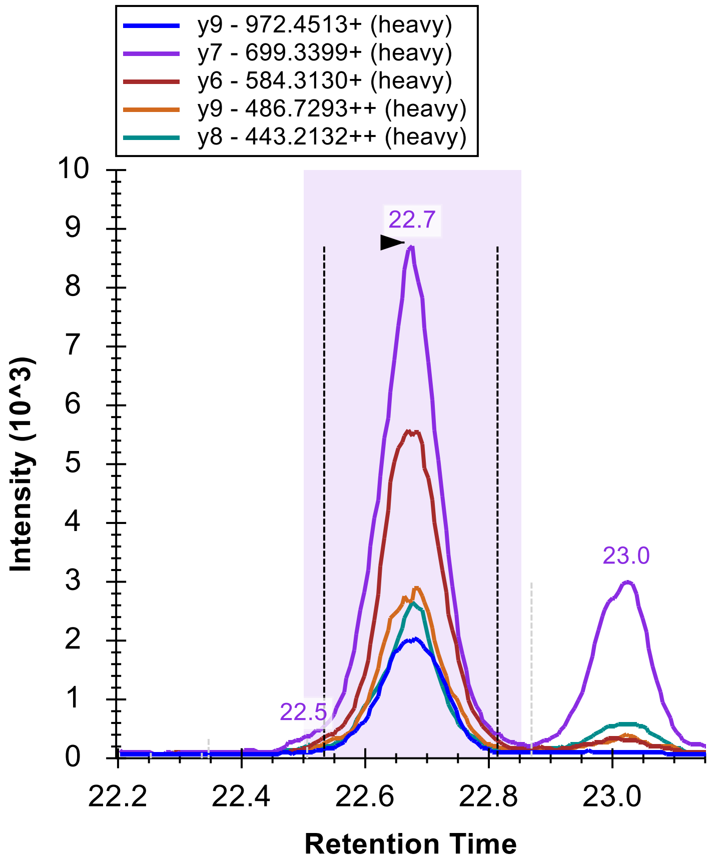


***Supplementary Figure 5u:*** *PEBP1_NRPT*

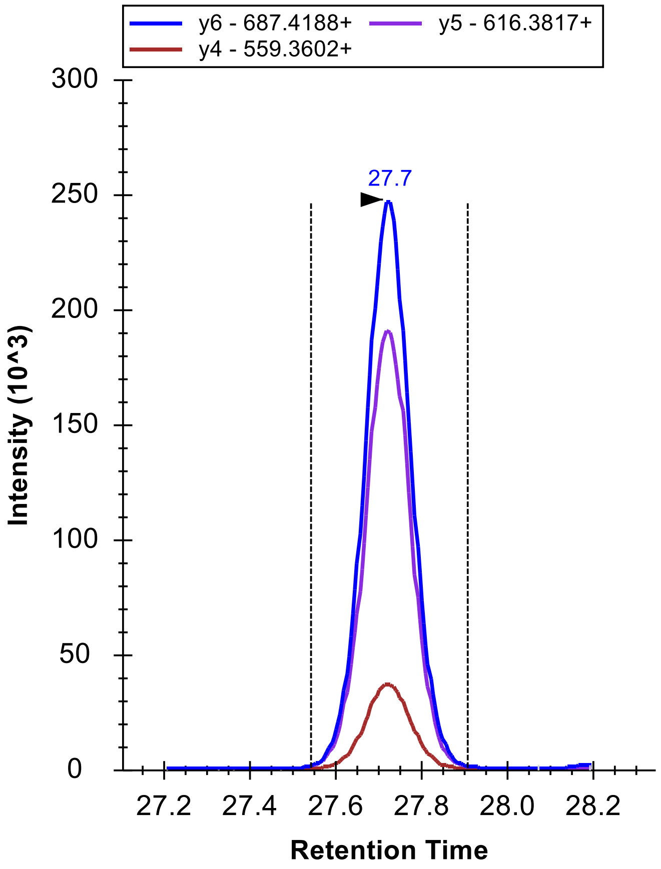


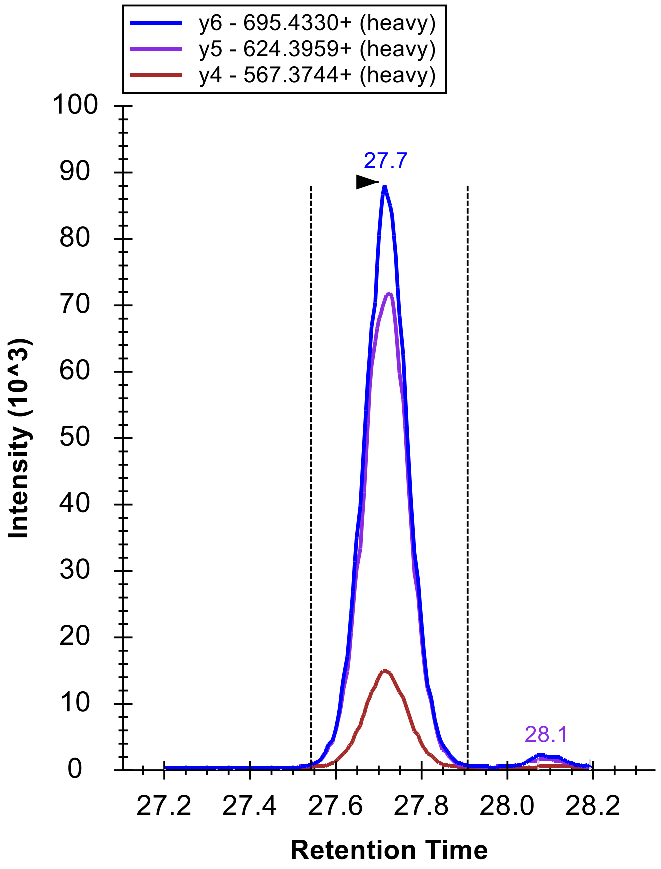


***Supplementary Figure 5v:*** *Spectrum SAP3_EVA*

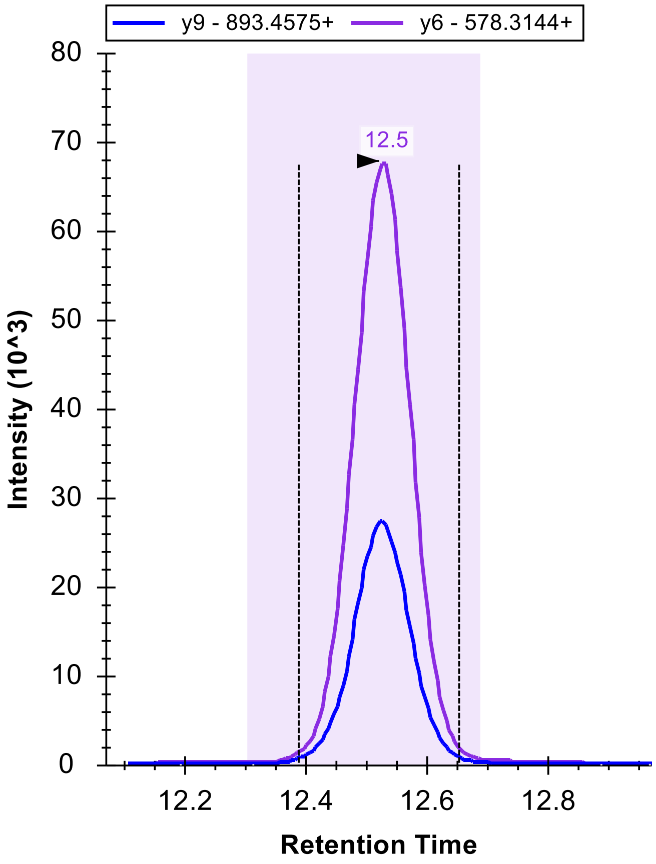


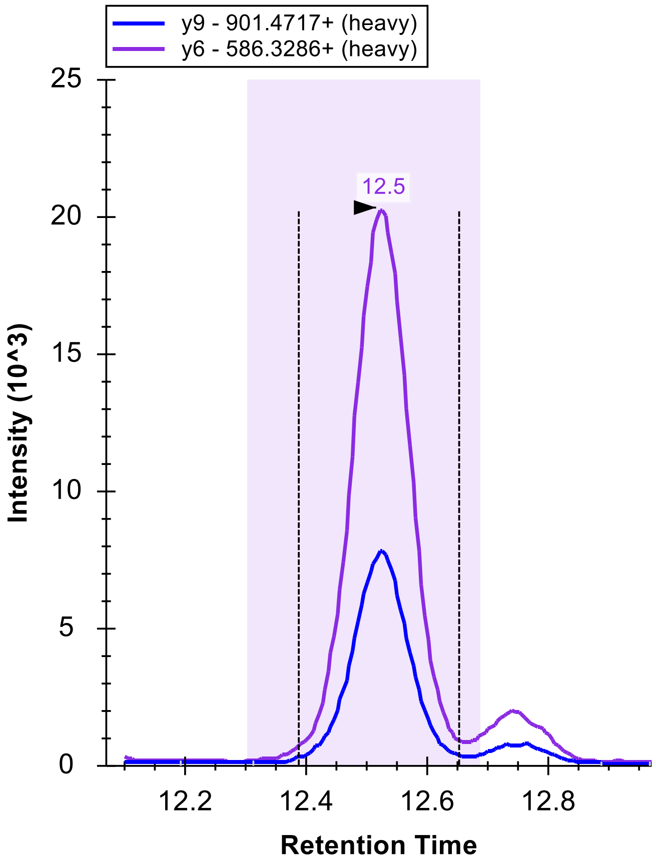


***Supplementary Figure 5w:*** *Spectrum SAP3_IES*

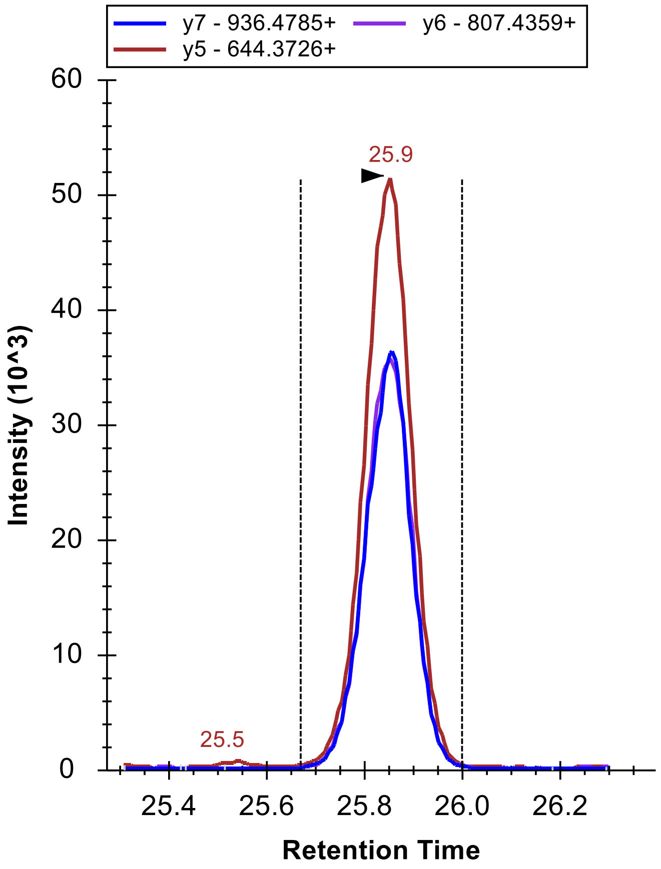


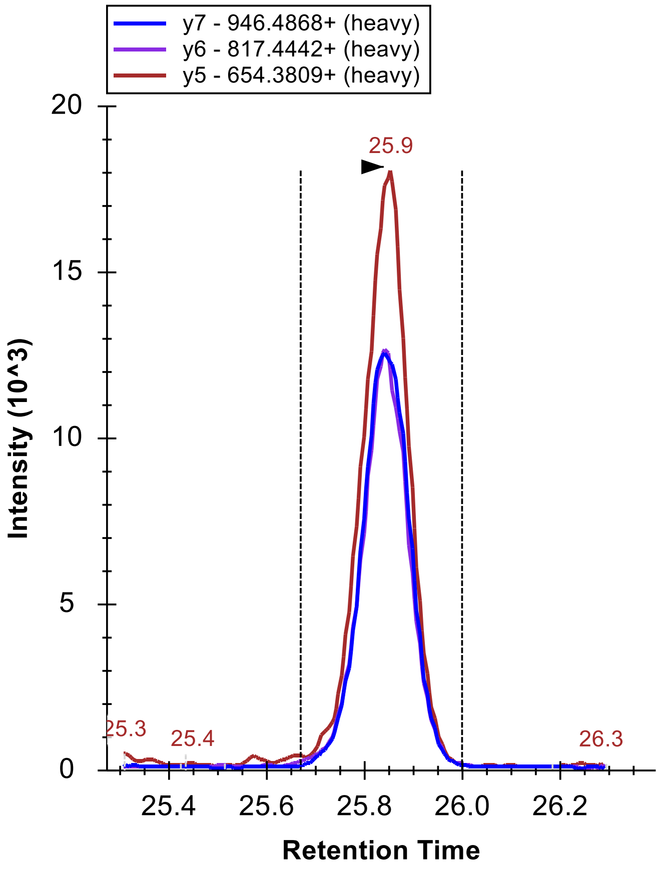


***Supplementary Figure 5x:*** *Spectrum SCG2_ALE*

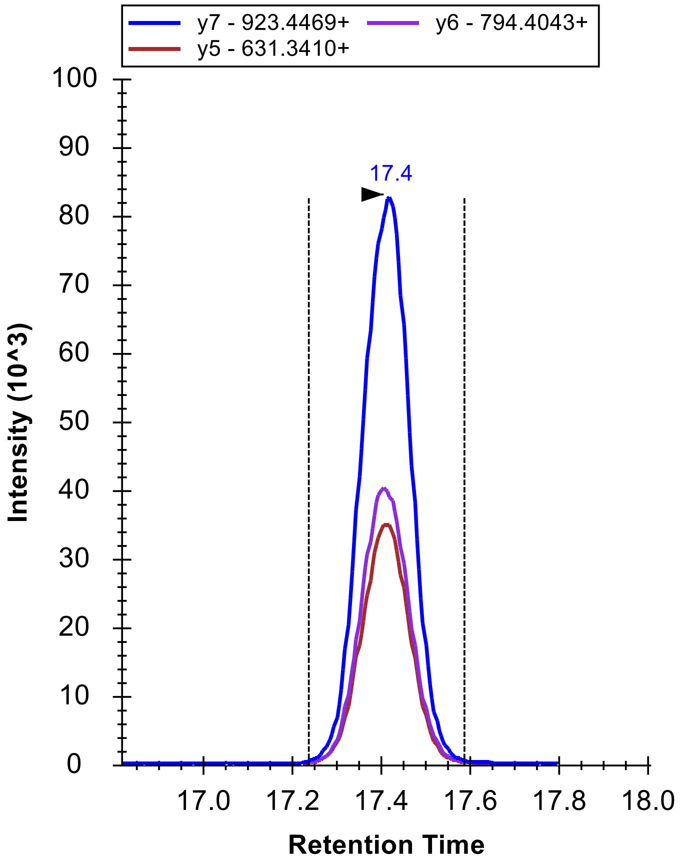


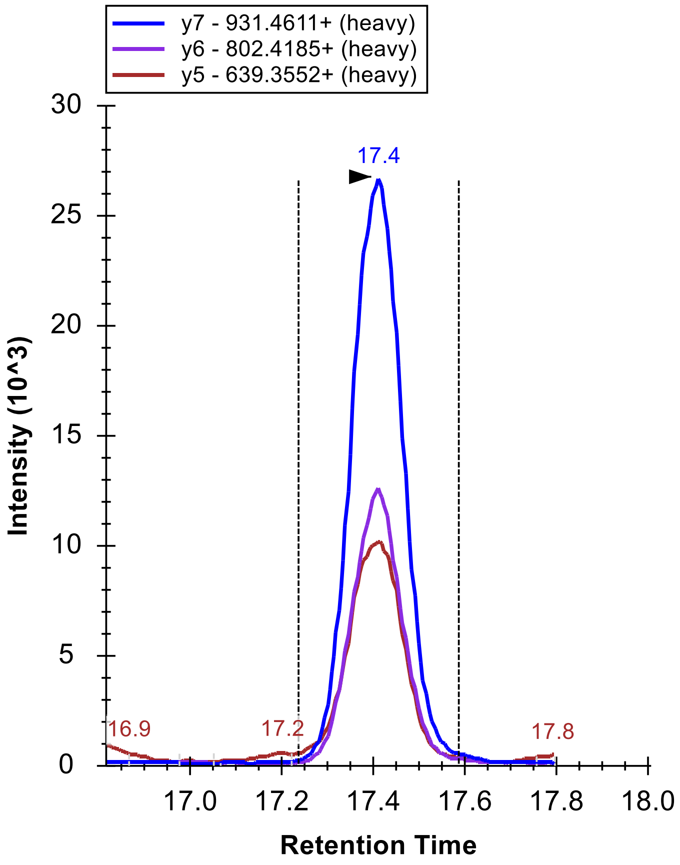


***Supplementary Figure 5y:*** *Spectrum SCG2_VLE*

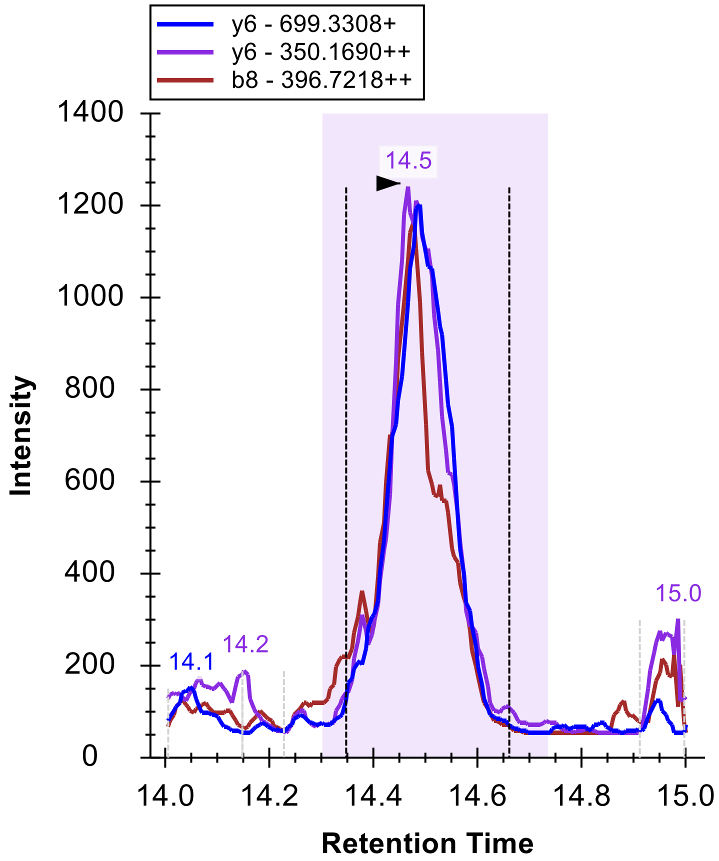


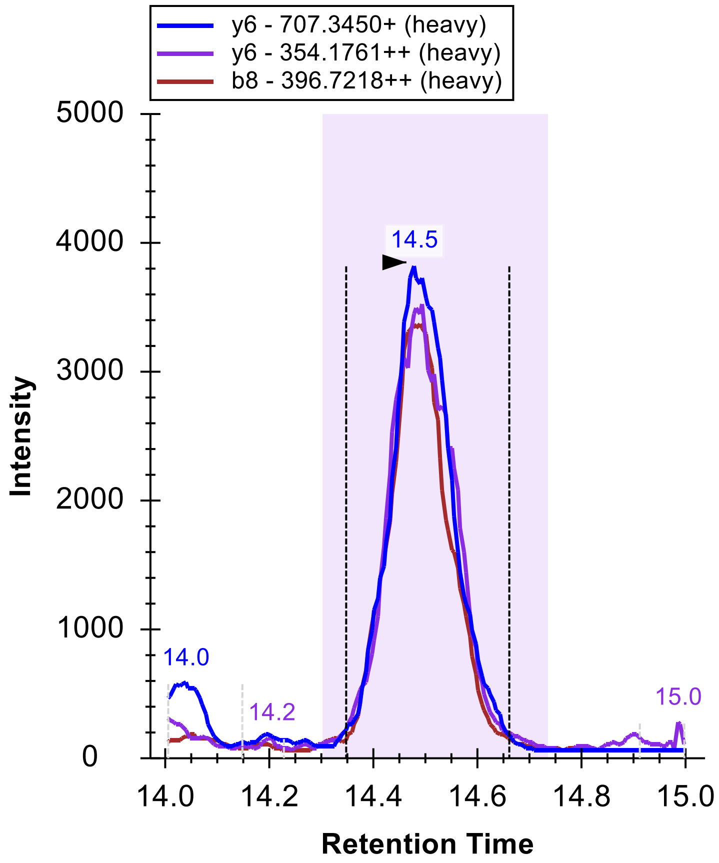


***Supplementary Figure 5z:*** *Spectrum STX1B*

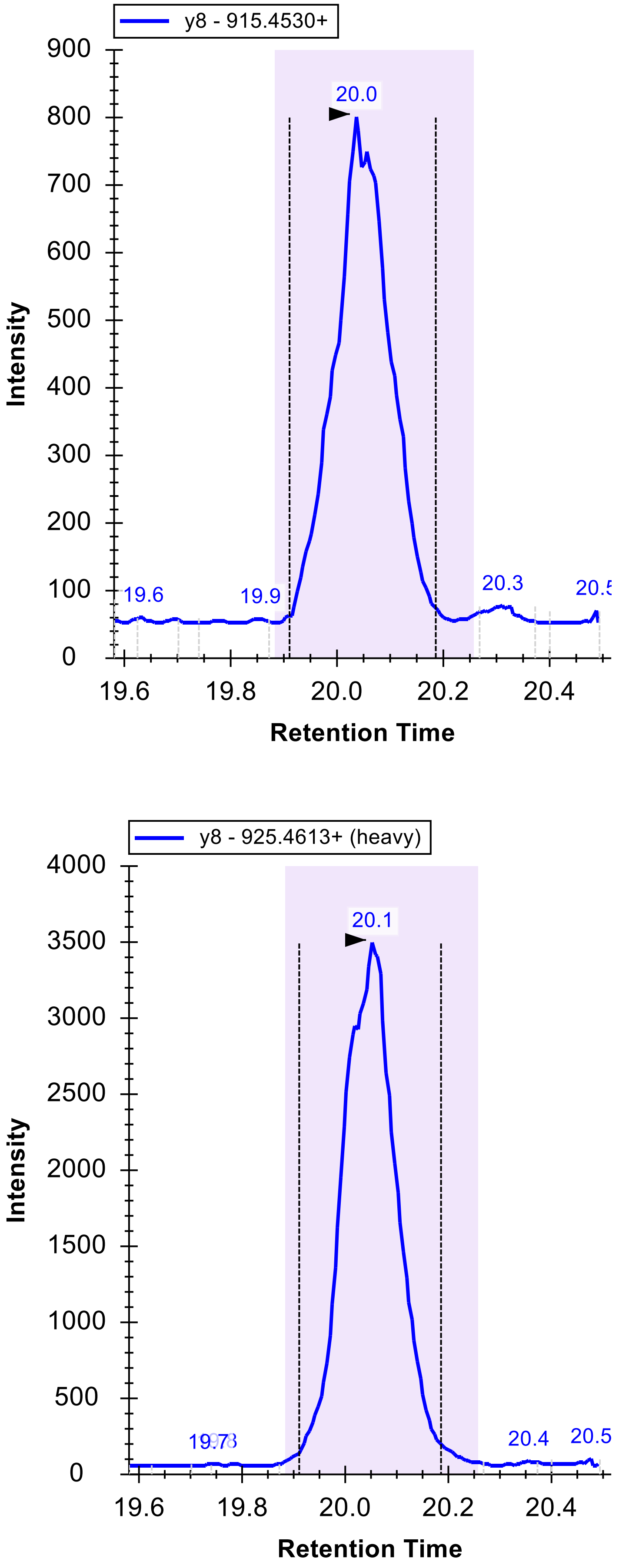


***Supplementary Figure 5a1:*** *Spectrum STX7*

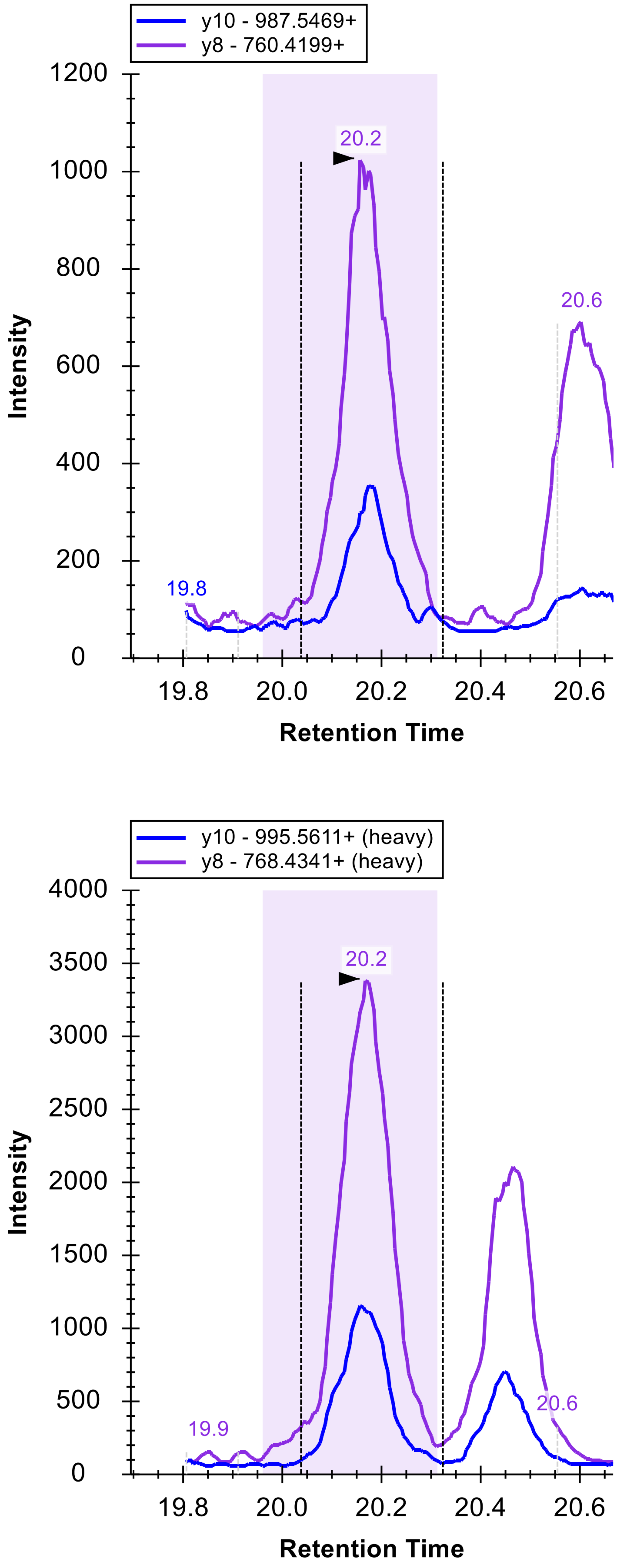


***Supplementary Figure 5b1:*** *Spectrum SYUB*

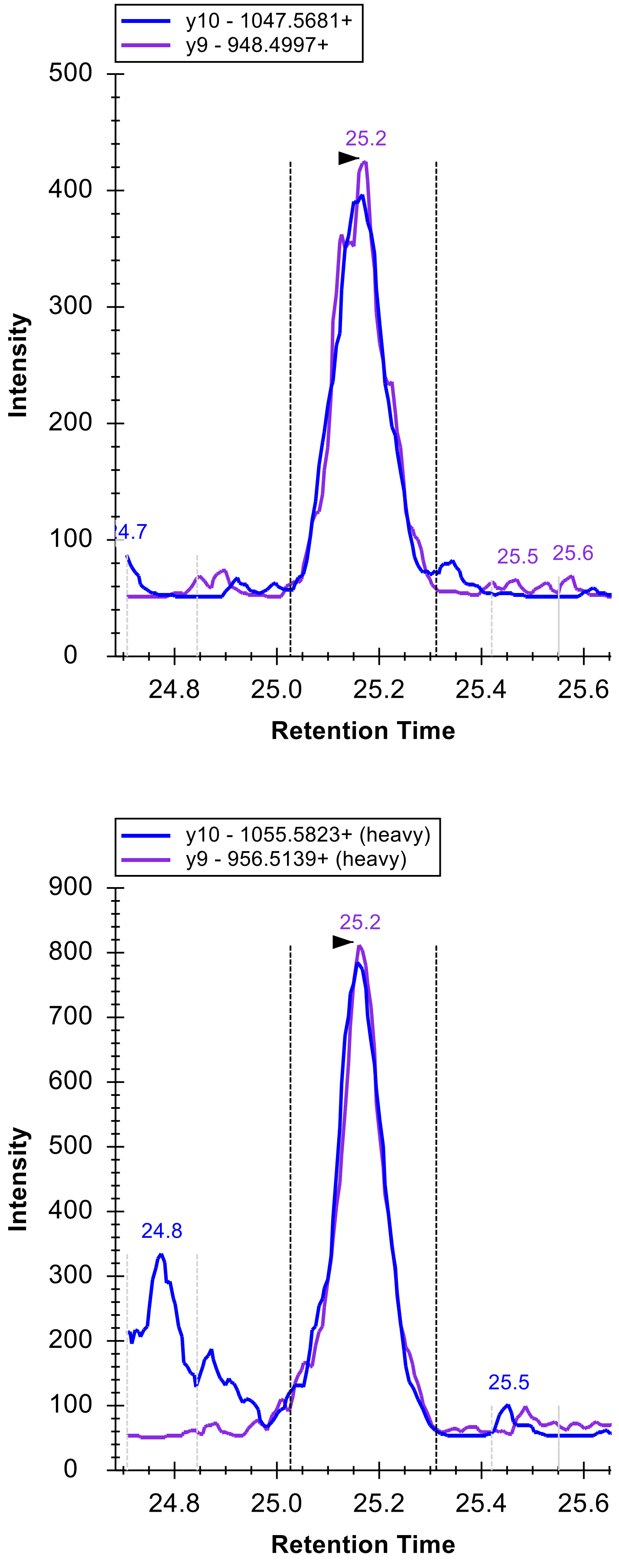


***Supplementary Figure 5b2:*** *Spectrum SYUG*

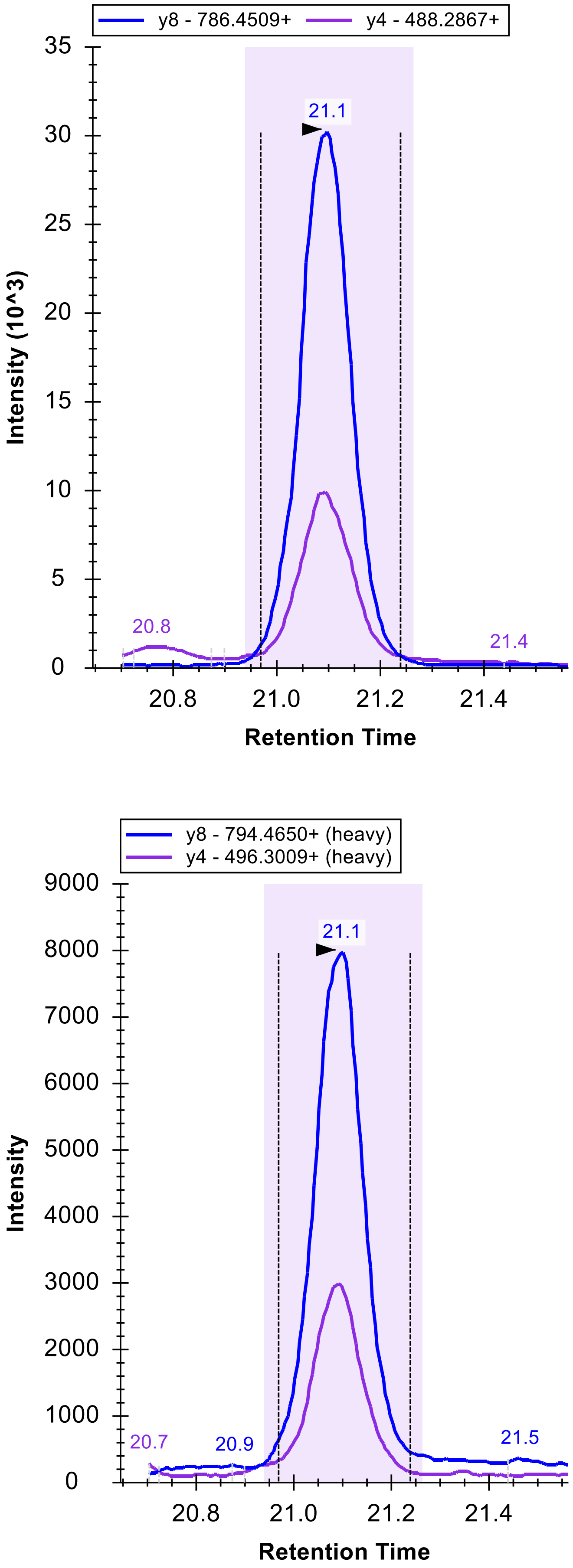


***Supplementary Figure 5b3:*** *Spectrum VGF_AYQ*

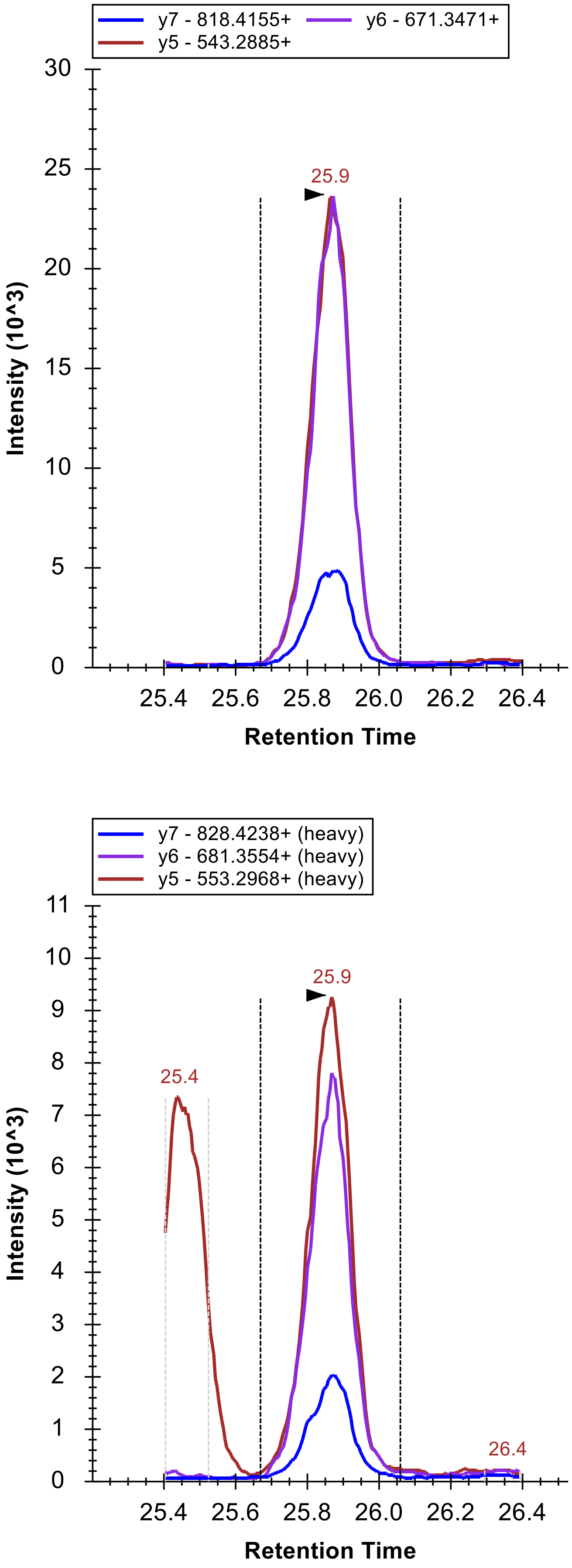


***Supplementary Figure 5b4:*** *Spectrum VGF_NSE*

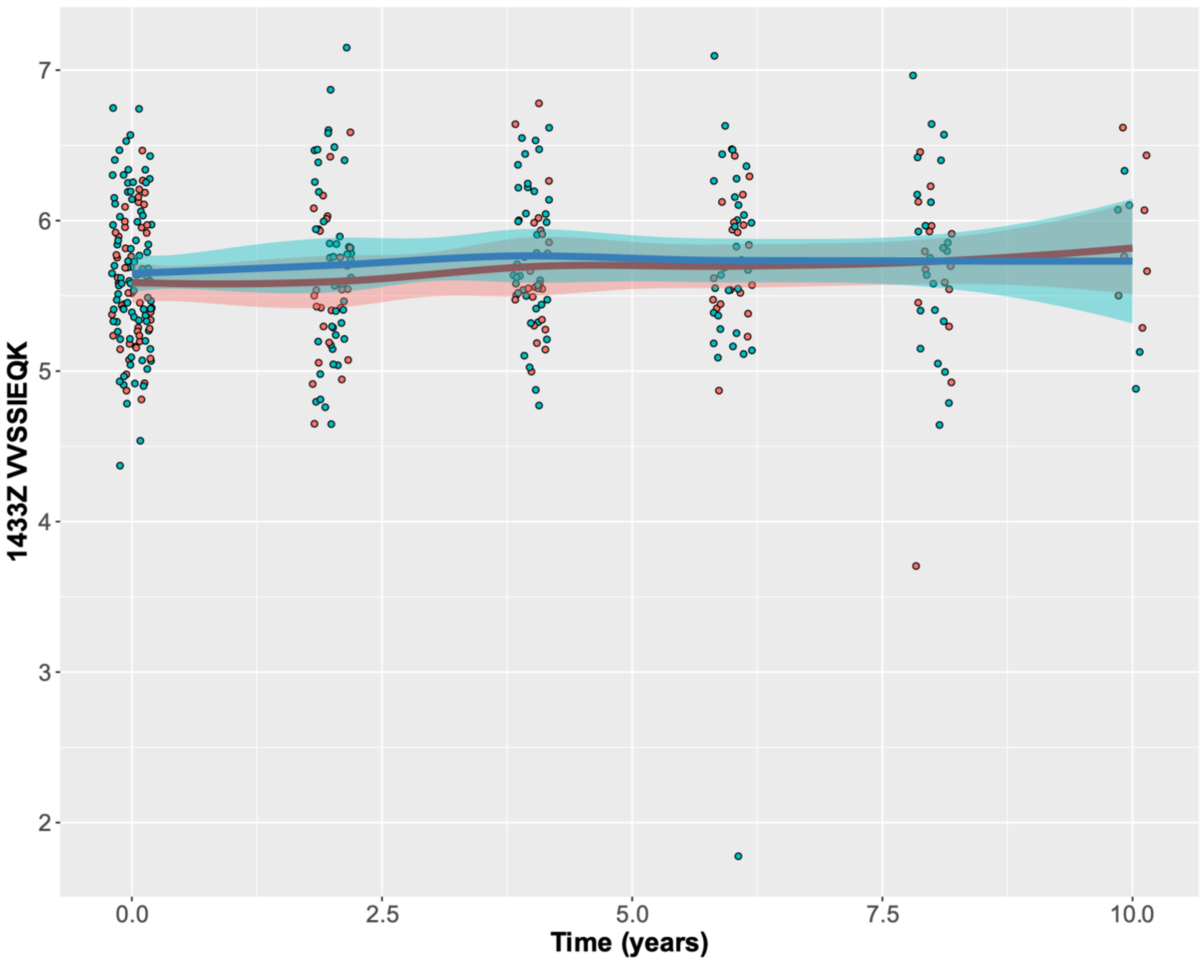


***Supplementary Figure 6a:*** *Longitudinal analysis of the cerebrospinal fluid levels over 10 years. The ribbon gives estimates of the standard error, the solid line gives a loose fit of the measured data points. Parkinson's disease: blue, healthy controls: red*

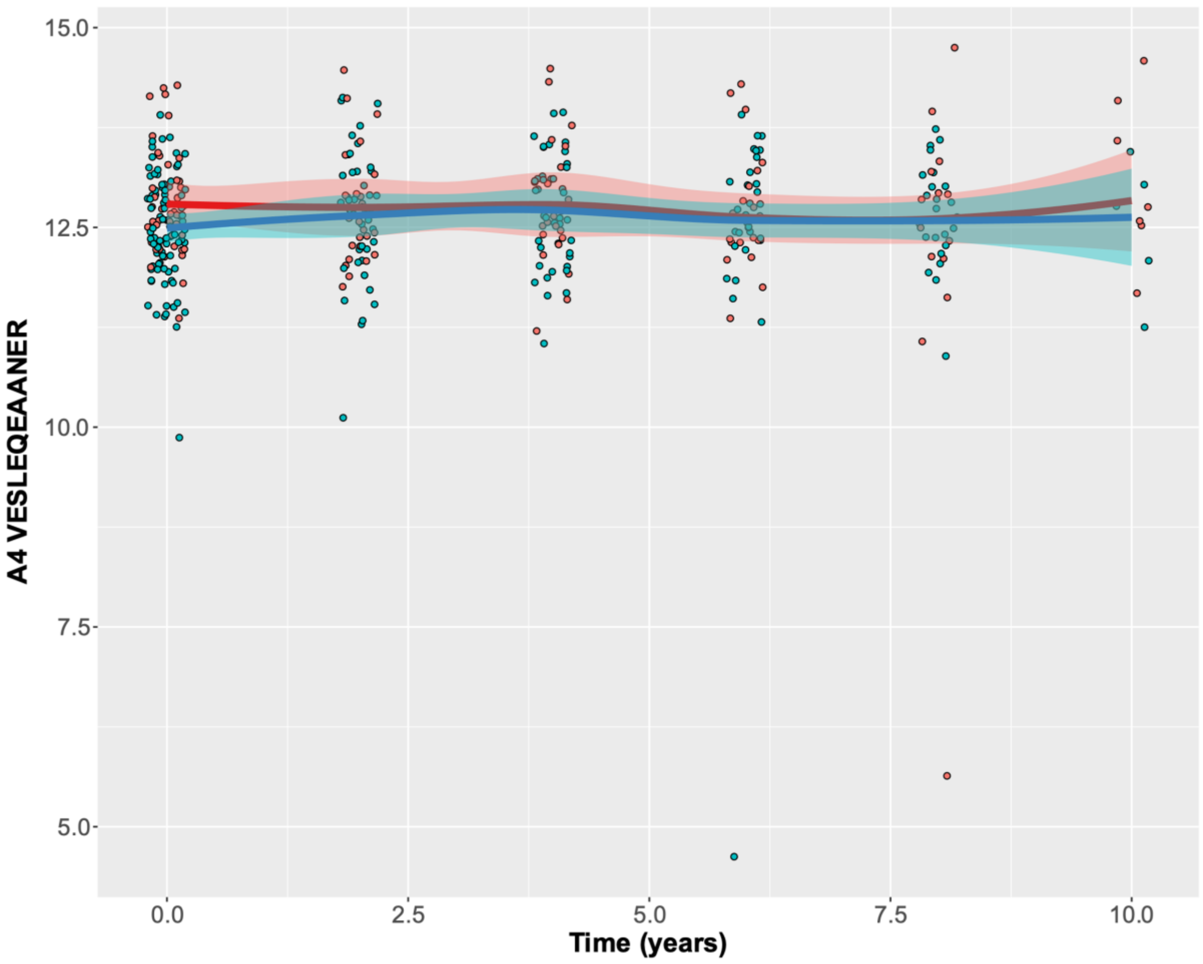


***Supplementary Figure 6b:*** *Longitudinal analysis of the cerebrospinal fluid levels over 10 years. The ribbon gives estimates of the standard error, the solid line gives a loose fit of the measured data points. Parkinson's disease: blue, healthy controls: red*

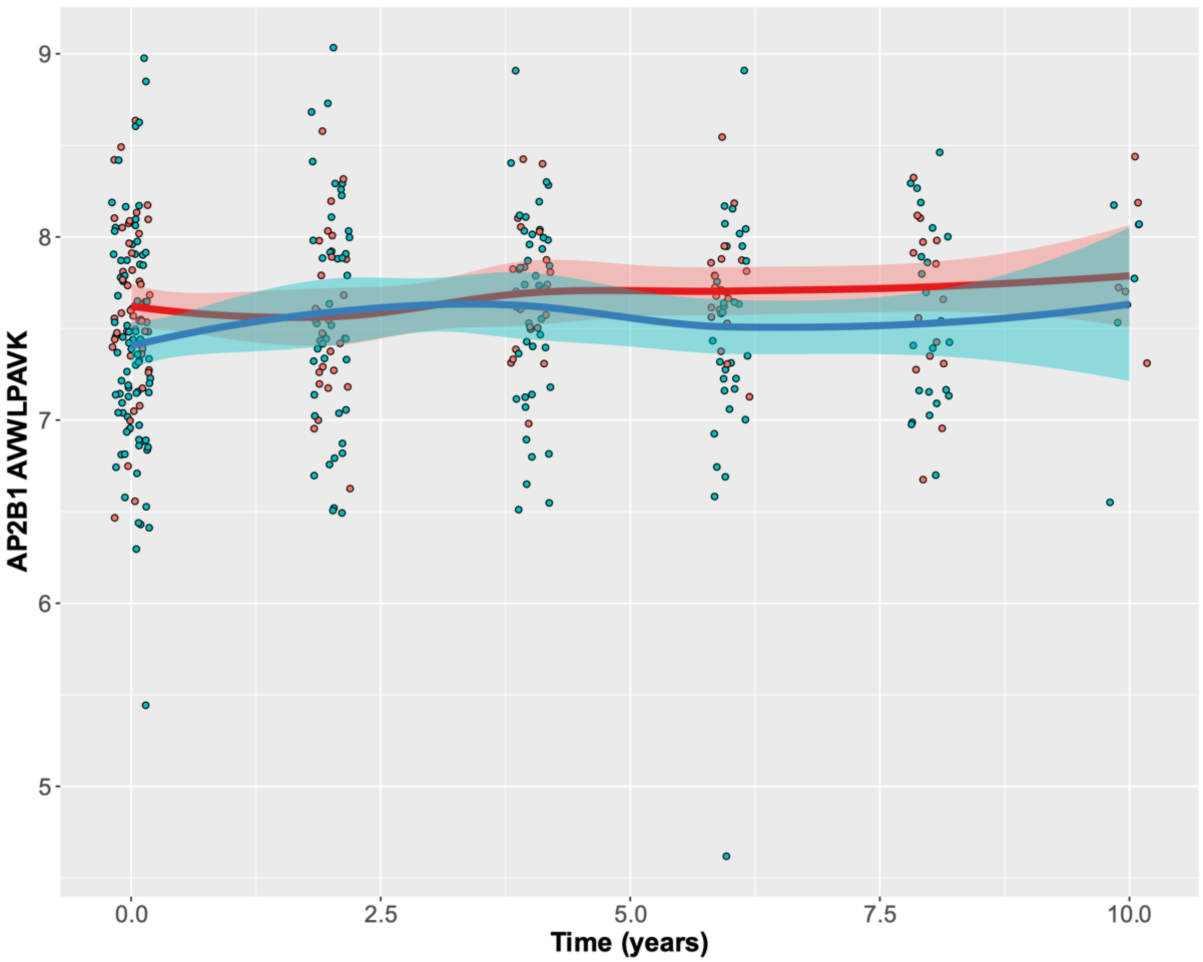


***Supplementary Figure 6c:*** *Longitudinal analysis of the cerebrospinal fluid levels over 10 years. The ribbon gives estimates of the standard error, the solid line gives a loose fit of the measured data points. Parkinson's disease: blue, healthy controls: red*

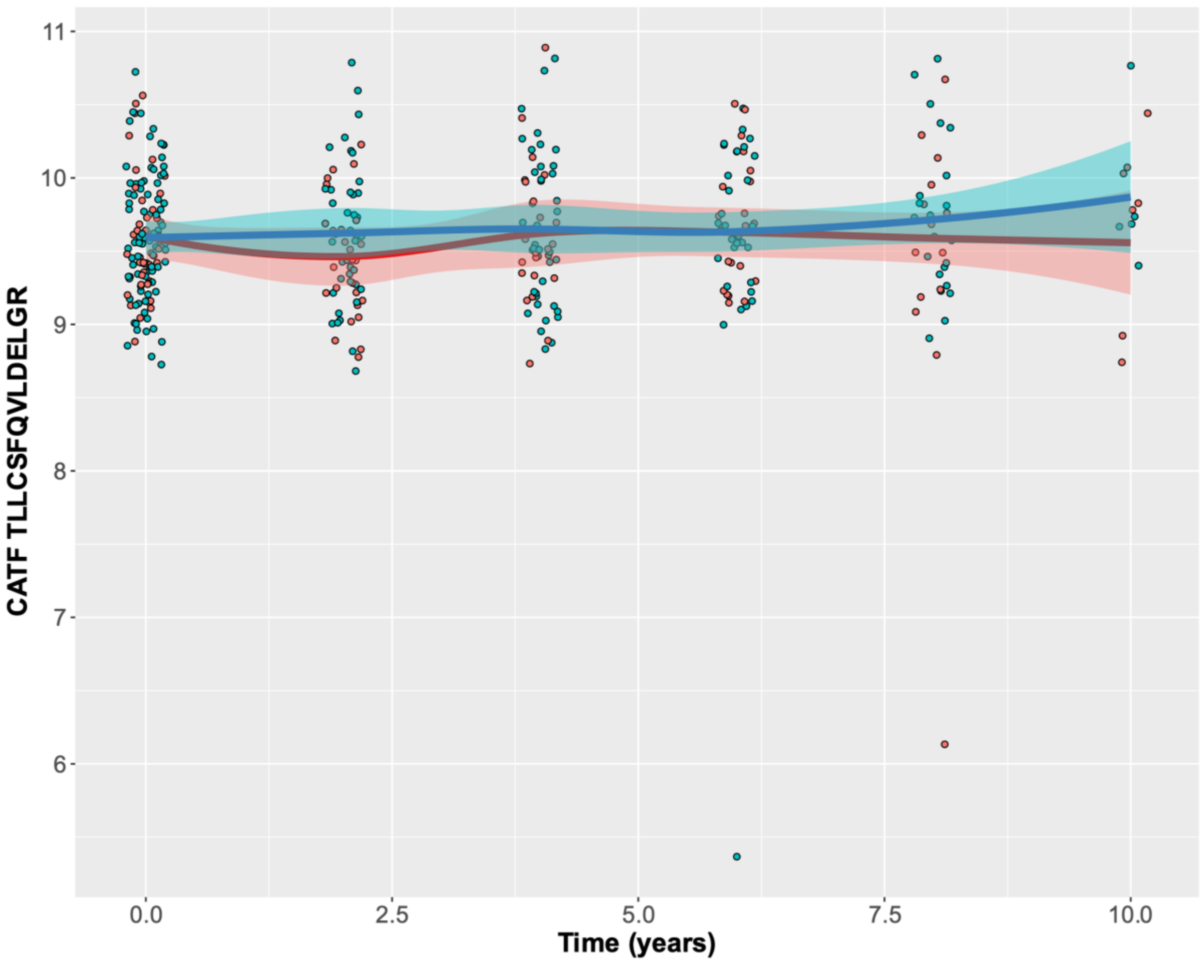


***Supplementary Figure 6d:*** *Longitudinal analysis of the cerebrospinal fluid levels over 10 years. The ribbon gives estimates of the standard error, the solid line gives a loose fit of the measured data points. Parkinson's disease: blue, healthy controls: red*

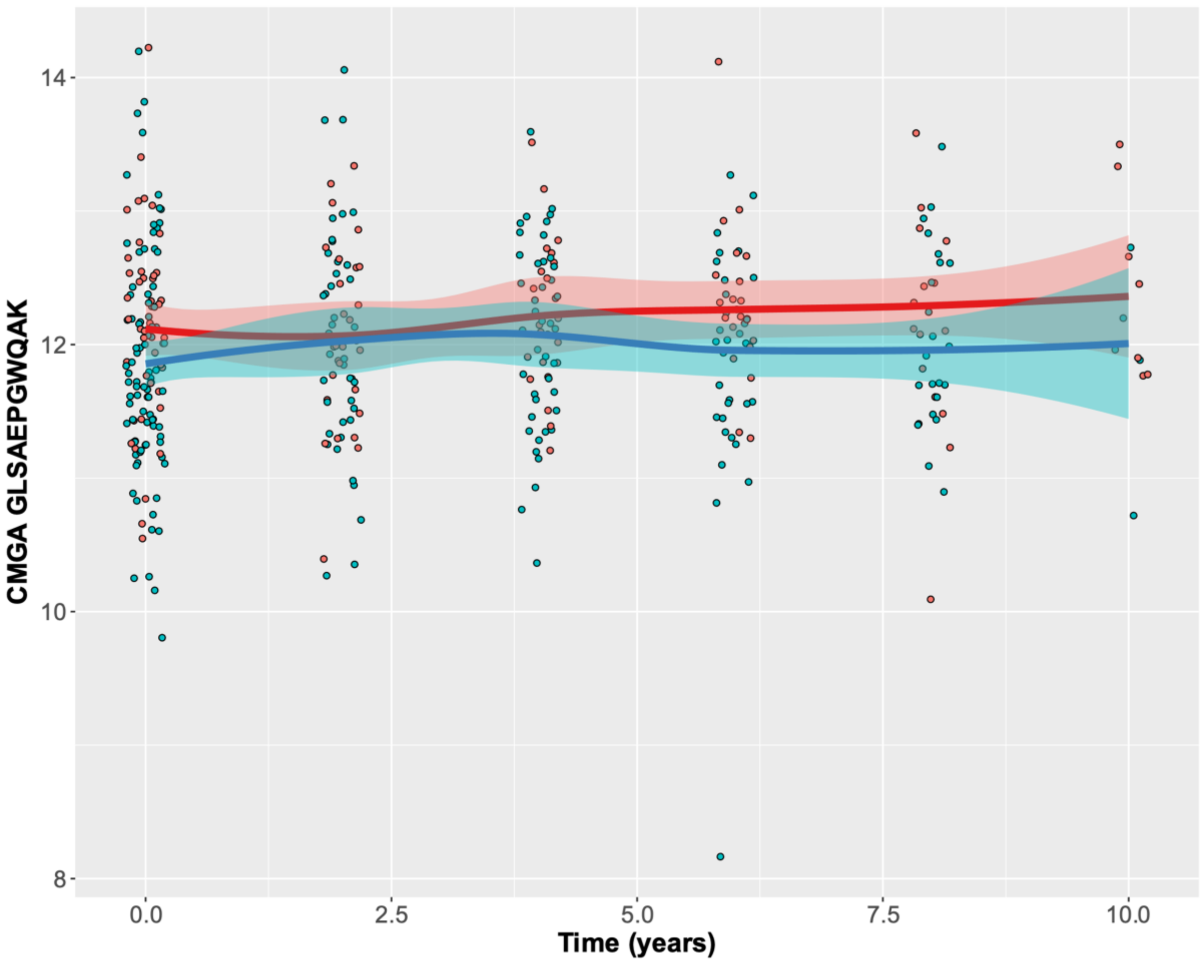


***Supplementary Figure 6e:*** *Longitudial analysis of the cerebrospinal fluid levels over 10 years. The ribbon gives estimates of the standard error, the solid line gives a loose fit of the measured data points. Parkinson's disease: blue, healthy controls: red*

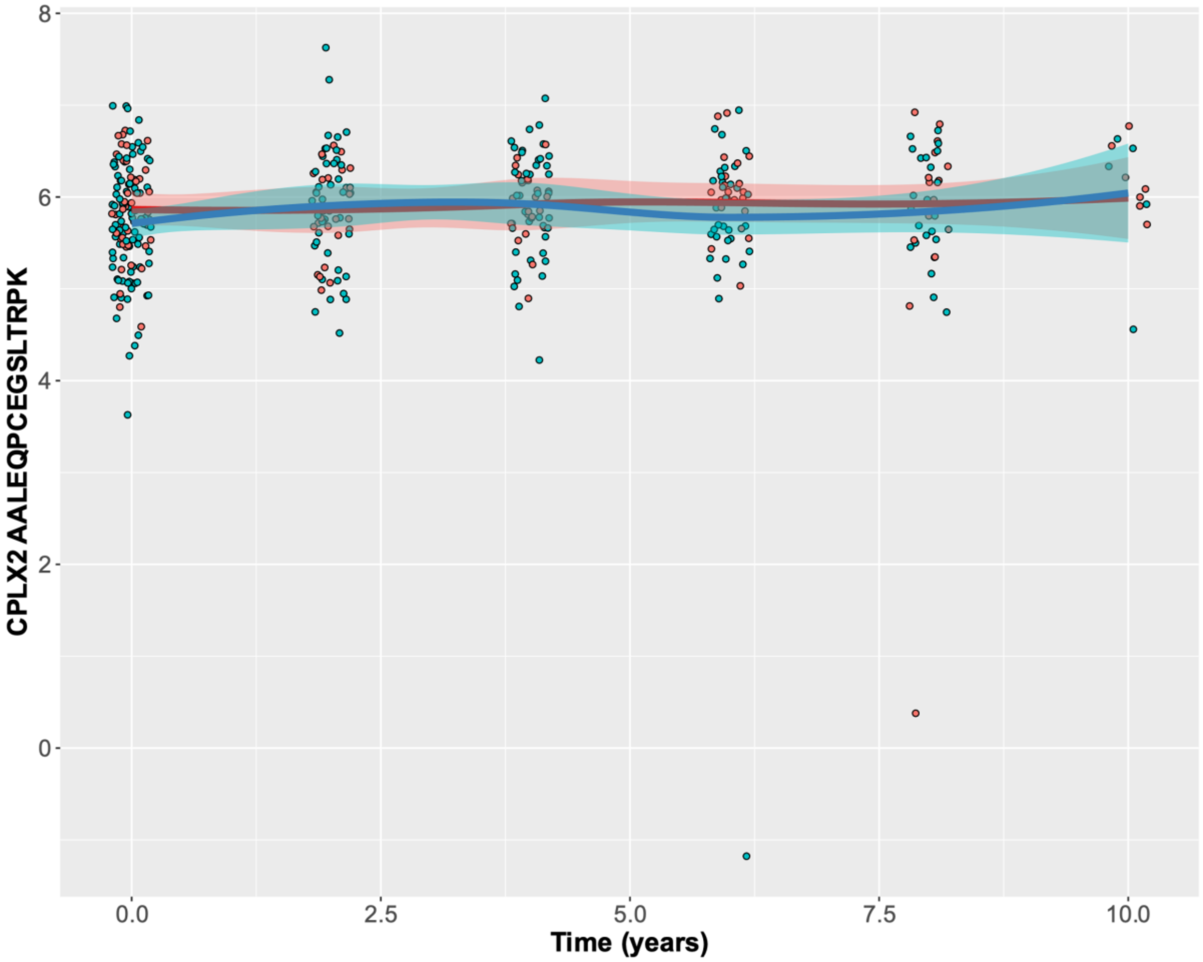


***Supplementary Figure 6f:*** *Longitudial analysis of the cerebrospinal fluid levels over 10 years. The ribbon gives estimates of the standard error, the solid line gives a loose fit of the measured data points. Parkinson's disease: blue, healthy controls: red*

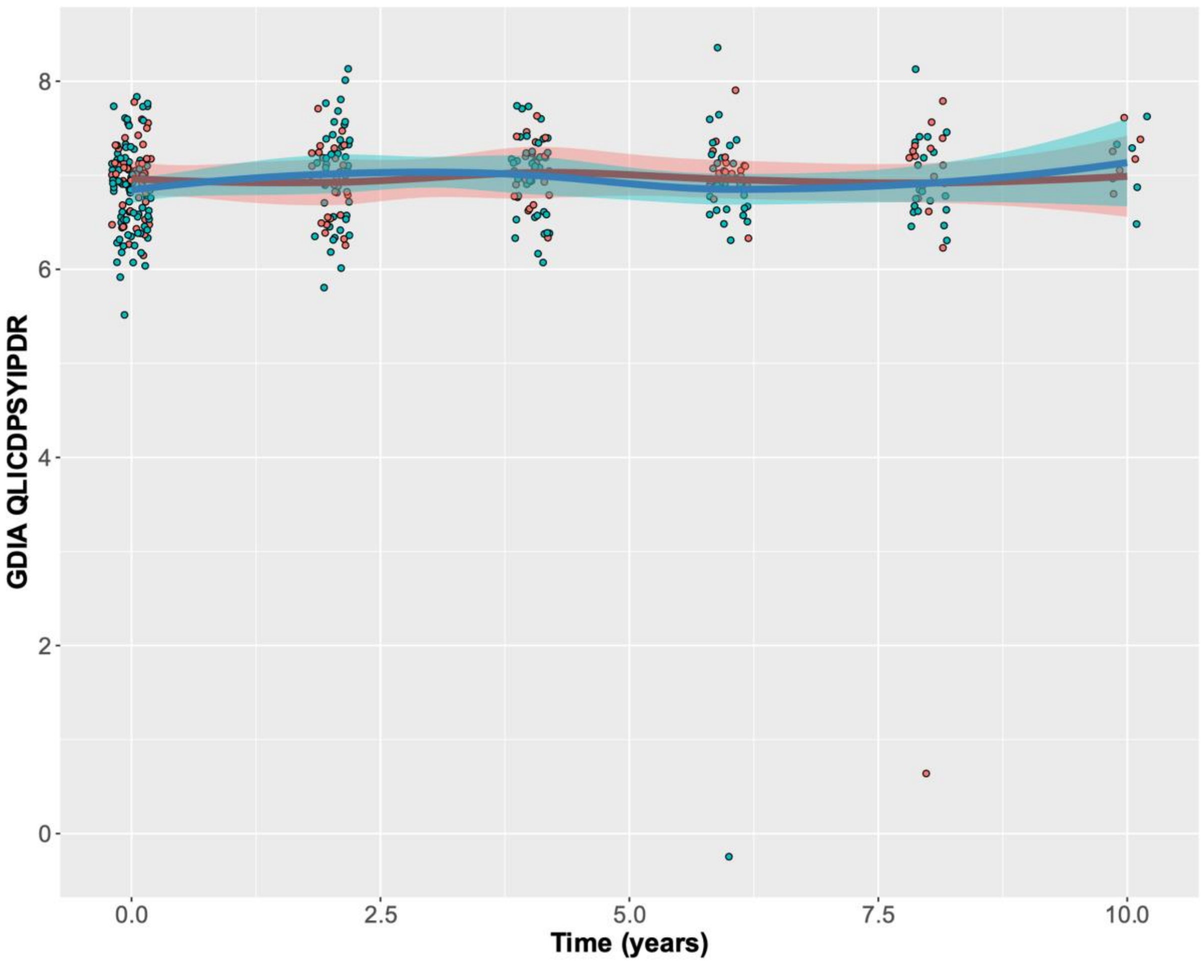


***Supplementary Figure 6g:*** *Longitudinal analysis of the cerebrospinal fluid levels over 10 years. The ribbon gives estimates of the standard error, the solid line gives a loose fit of the measured data points. Parkinson's disease: blue, healthy controls: red*

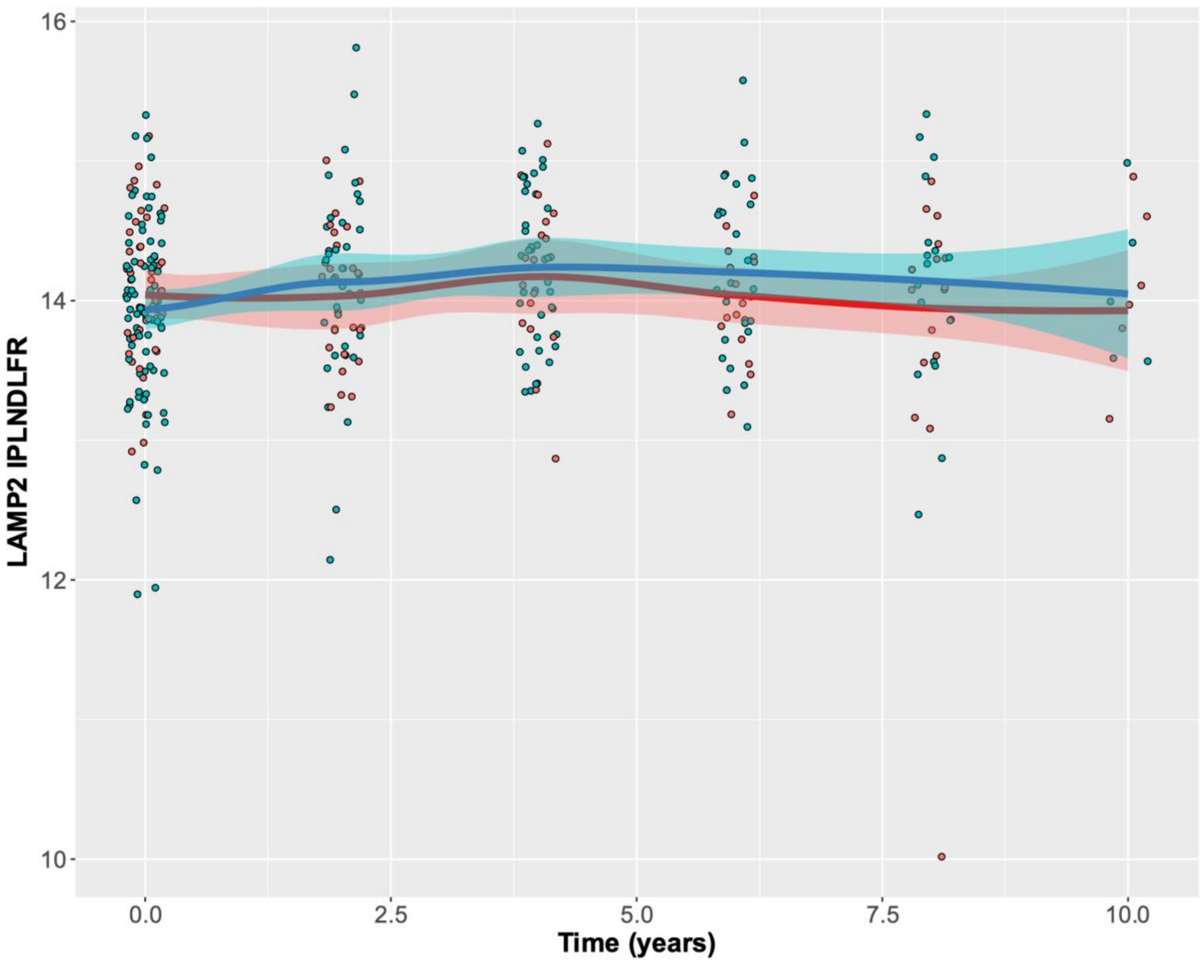


***Supplementary Figure 6h:*** *Longitudinal analysis of the cerebrospinal fluid levels over 10 years. The ribbon gives estimates of the standard error, the solid line gives a loose fit of the measured data points. Parkinson's disease: blue, healthy controls: red*

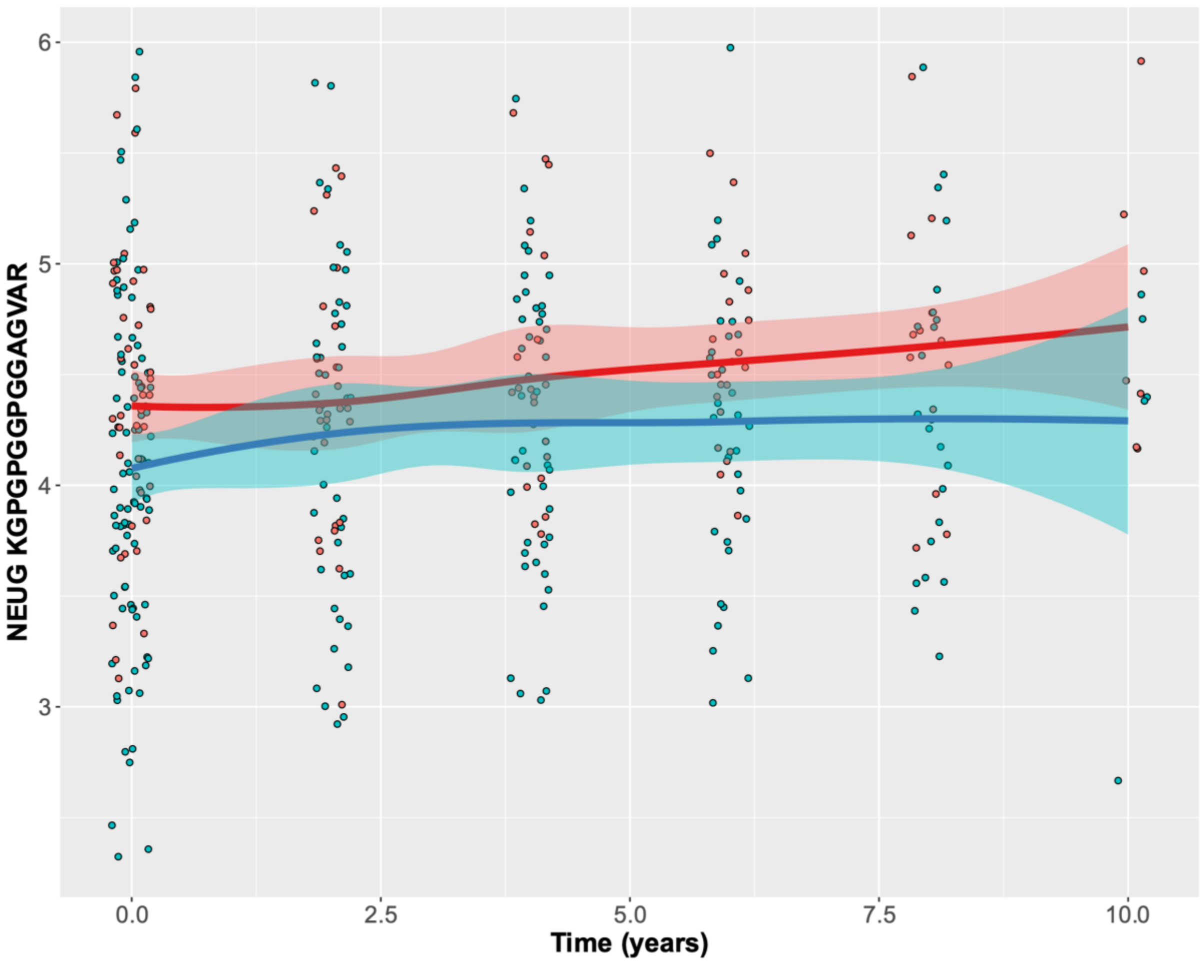


***Supplementary Figure 6i:*** *Longitudinal analysis of the cerebrospinal fluid levels over 10 years. The ribbon gives estimates of the standard error, the solid line gives a loose fit of the measured data points. Parkinson's disease: blue, healthy controls: red*

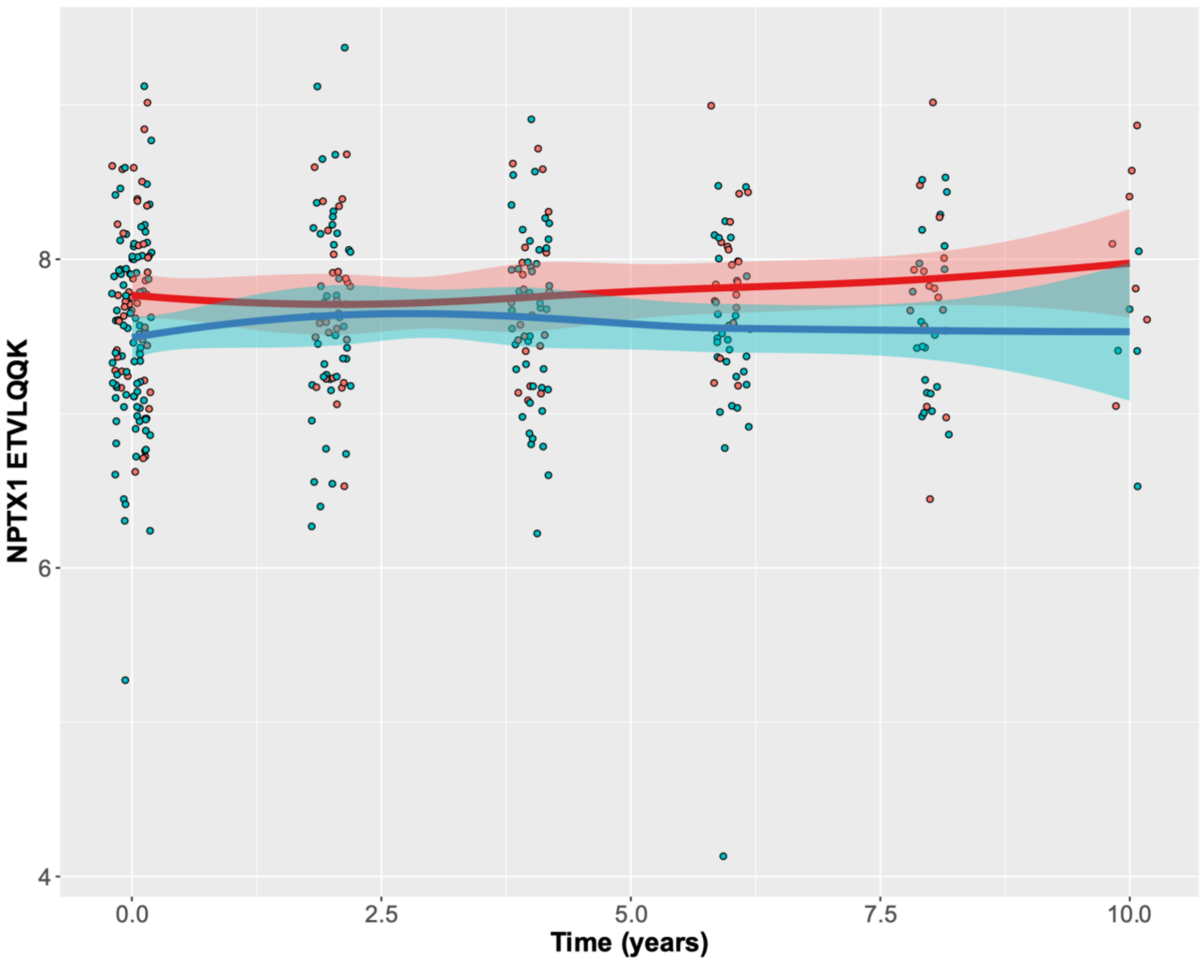


***Supplementary Figure 6j:*** *Longitudinal analysis of the cerebrospinal fluid levels over 10 years. The ribbon gives estimates of the standard error, the solid line gives a loose fit of the measured data points. Parkinson's disease: blue, healthy controls: red*

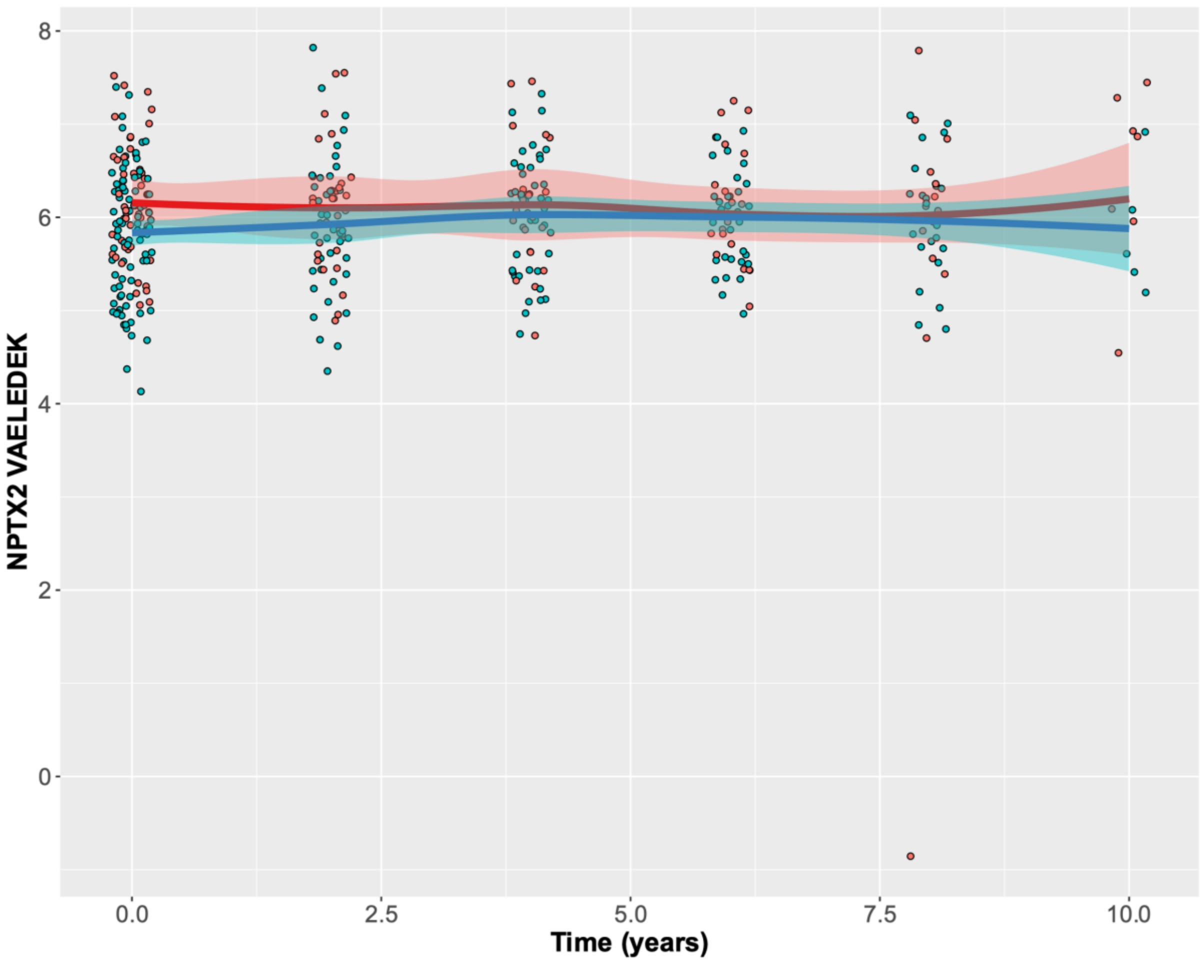


***Supplementary Figure 6k:*** *Longitudinal analysis of the cerebrospinal fluid levels over 10 years. The ribbon gives estimates of the standard error, the solid line gives a loose fit of the measured data points. Parkinson's disease: blue, healthy controls: red*

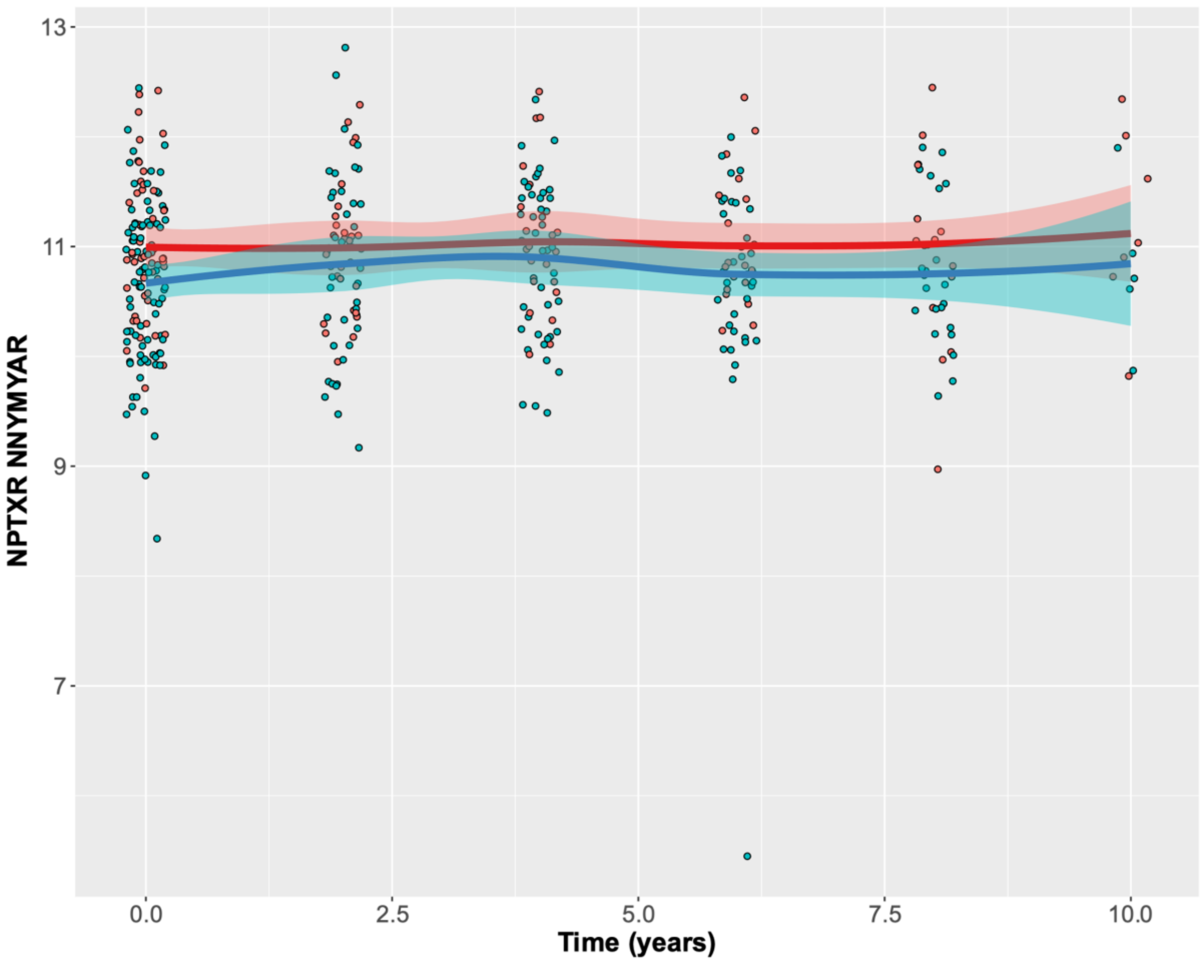


***Supplementary Figure 6l:*** *Longitudinal analysis of the cerebrospinal fluid levels over 10 years. The ribbon gives estimates of the standard error, the solid line gives a loose fit of the measured data points. Parkinson's disease: blue, healthy controls: red*

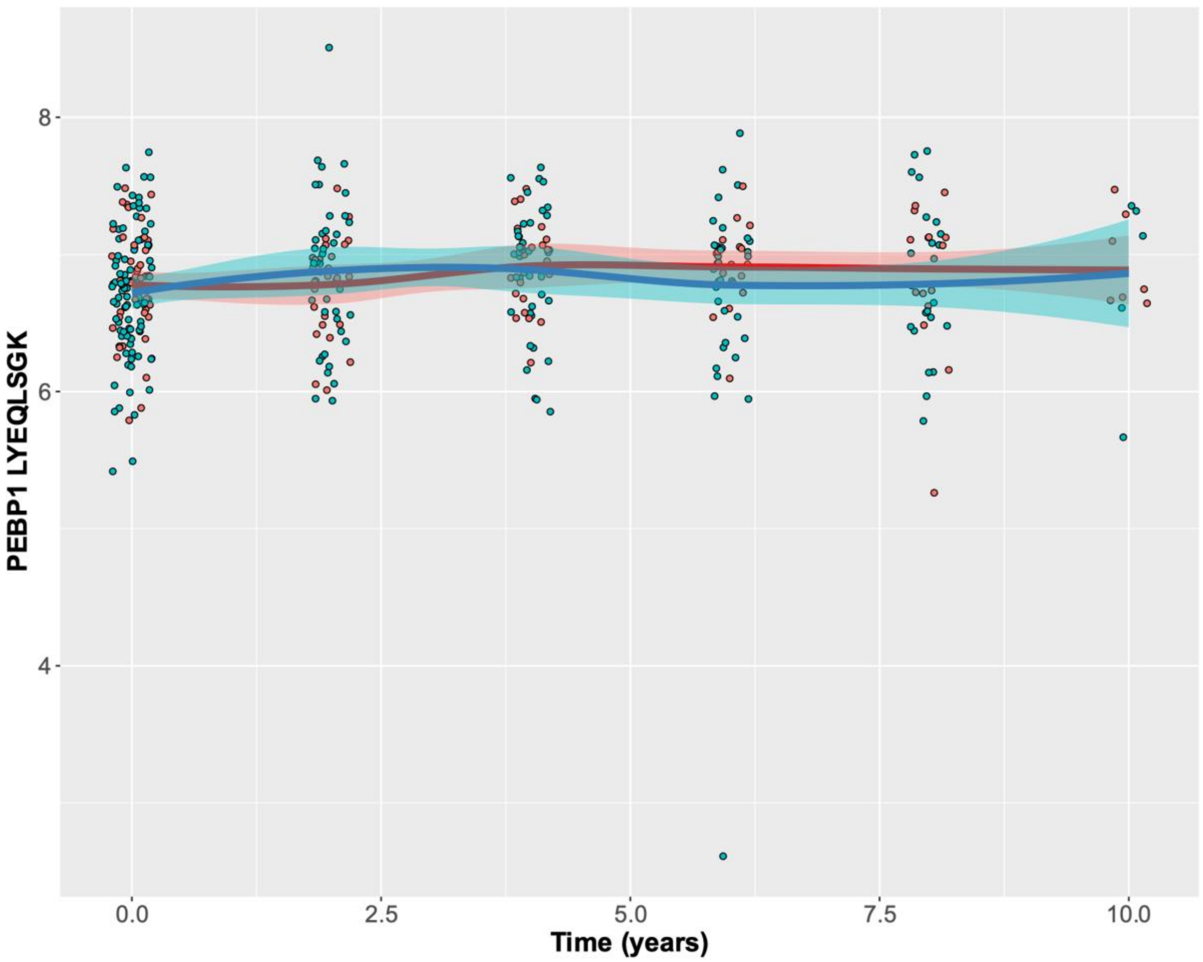


***Supplementary Figure 6m:*** *Longitudinal analysis of the cerebrospinal fluid levels over 10 years. The ribbon gives estimates of the standard error, the solid line gives a loose fit of the measured data points. Parkinson's disease: blue, healthy controls: red*

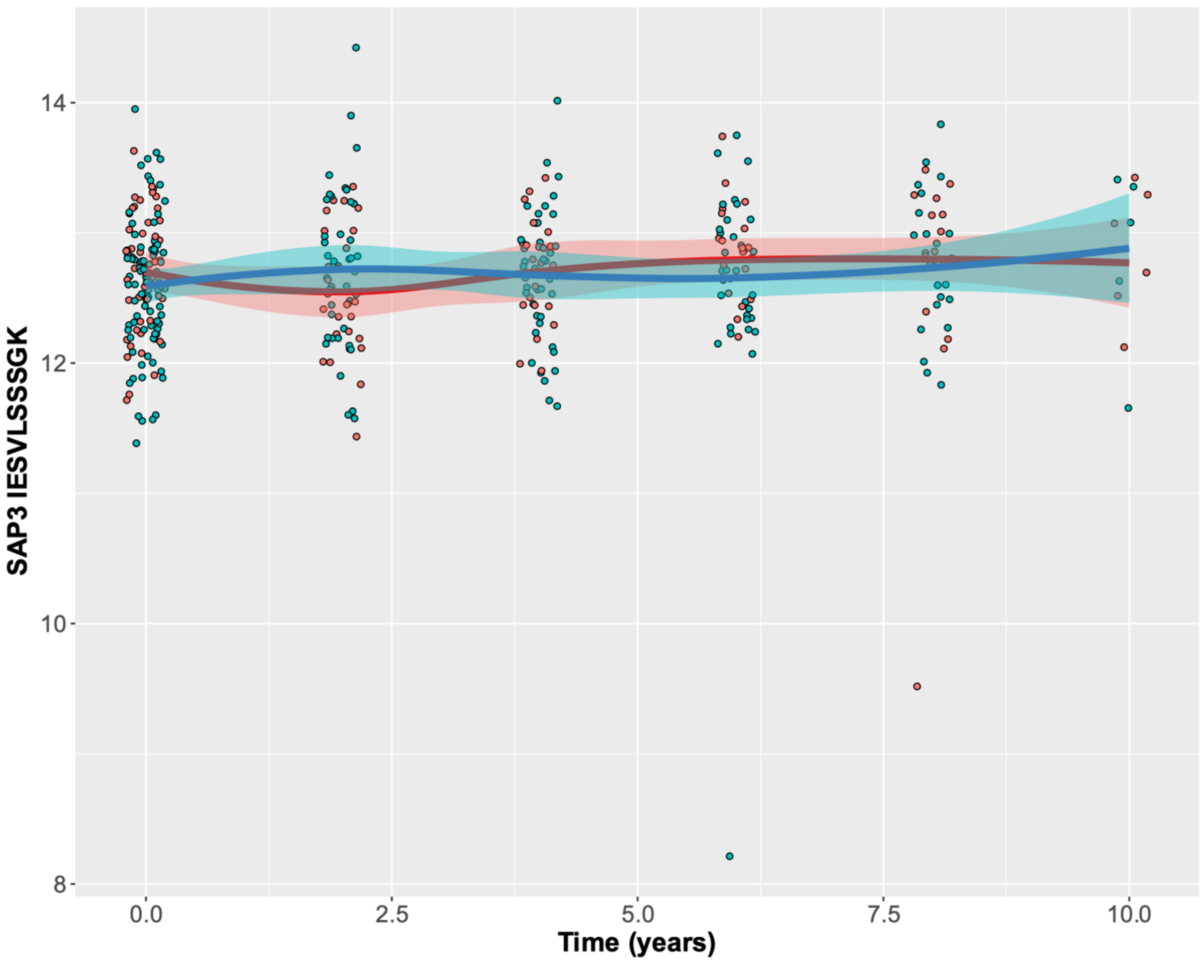


***Supplementary Figure 6n:*** *Longitudinal analysis of the cerebrospinal fluid levels over 10 years. The ribbon gives estimates of the standard error, the solid line gives a loose fit of the measured data points. Parkinson's disease: blue, healthy controls: red*

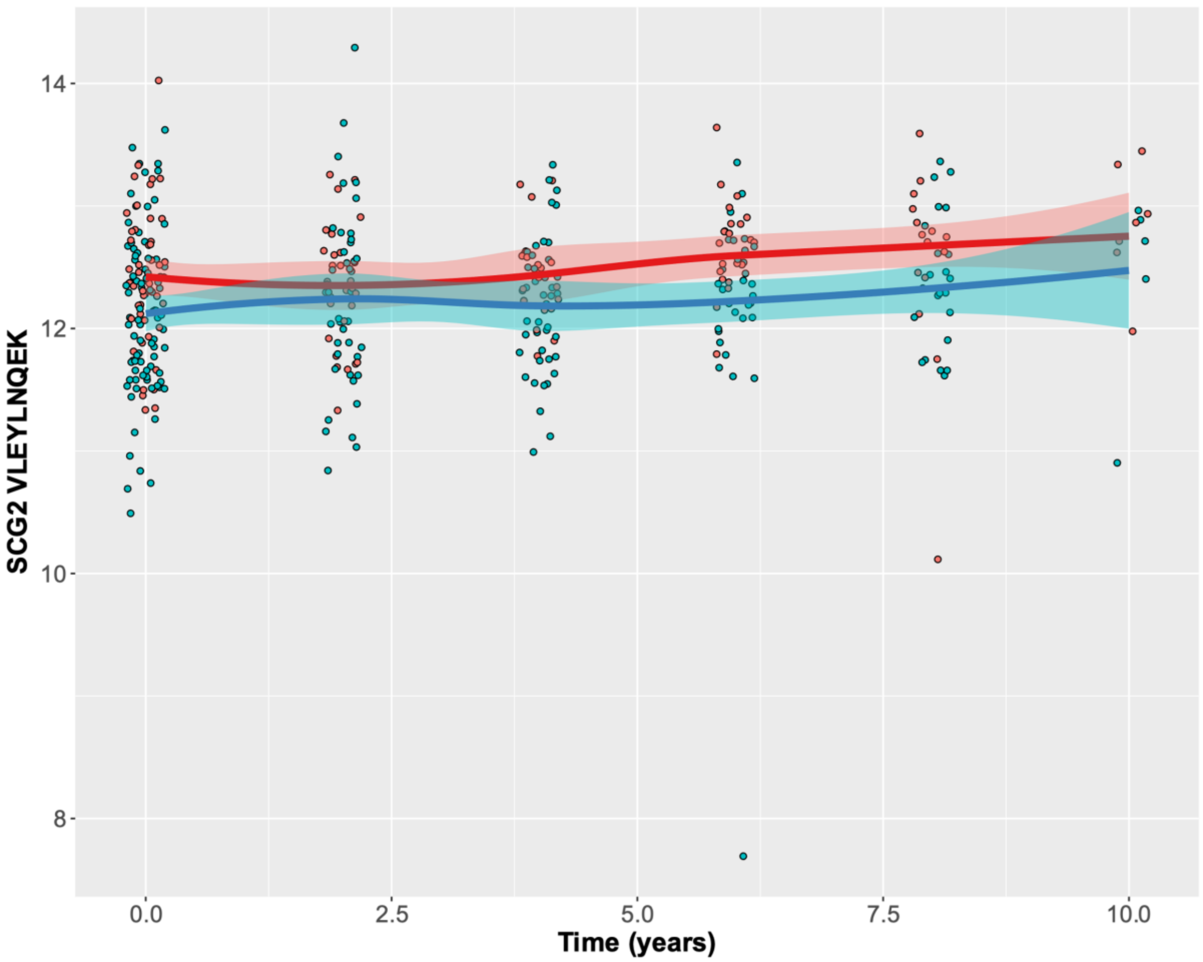


***Supplementary Figure 6o:*** *Longitudinal analysis of the cerebrospinal fluid levels over 10 years. The ribbon gives estimates of the standard error, the solid line gives a loose fit of the measured data points. Parkinson's disease: blue, healthy controls: red*

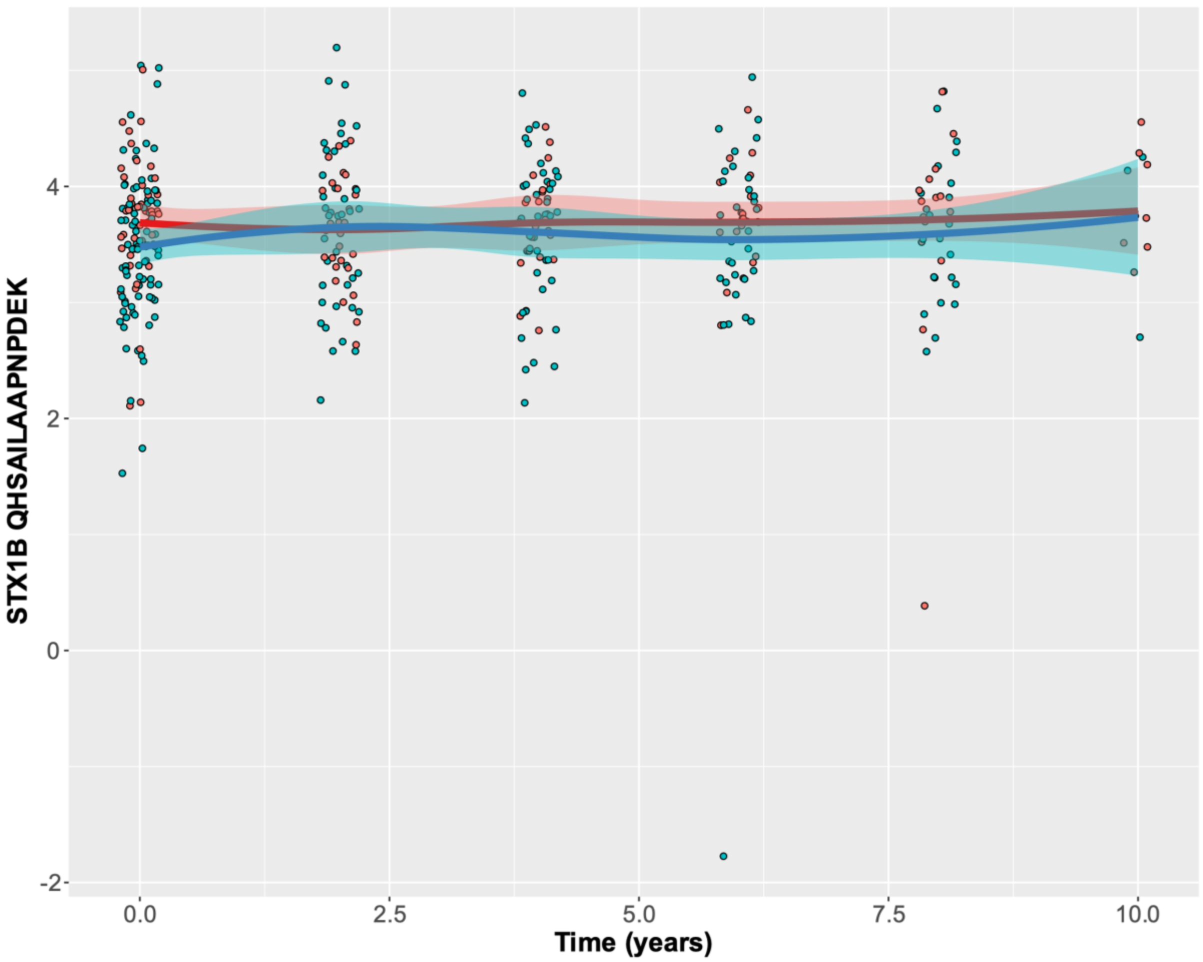


***Supplementary Figure 6p:*** *Longitudinal analysis of the cerebrospinal fluid levels over 10 years. The ribbon gives estimates of the standard error, the solid line gives a loose fit of the measured data points. Parkinson's disease: blue, healthy controls: red*

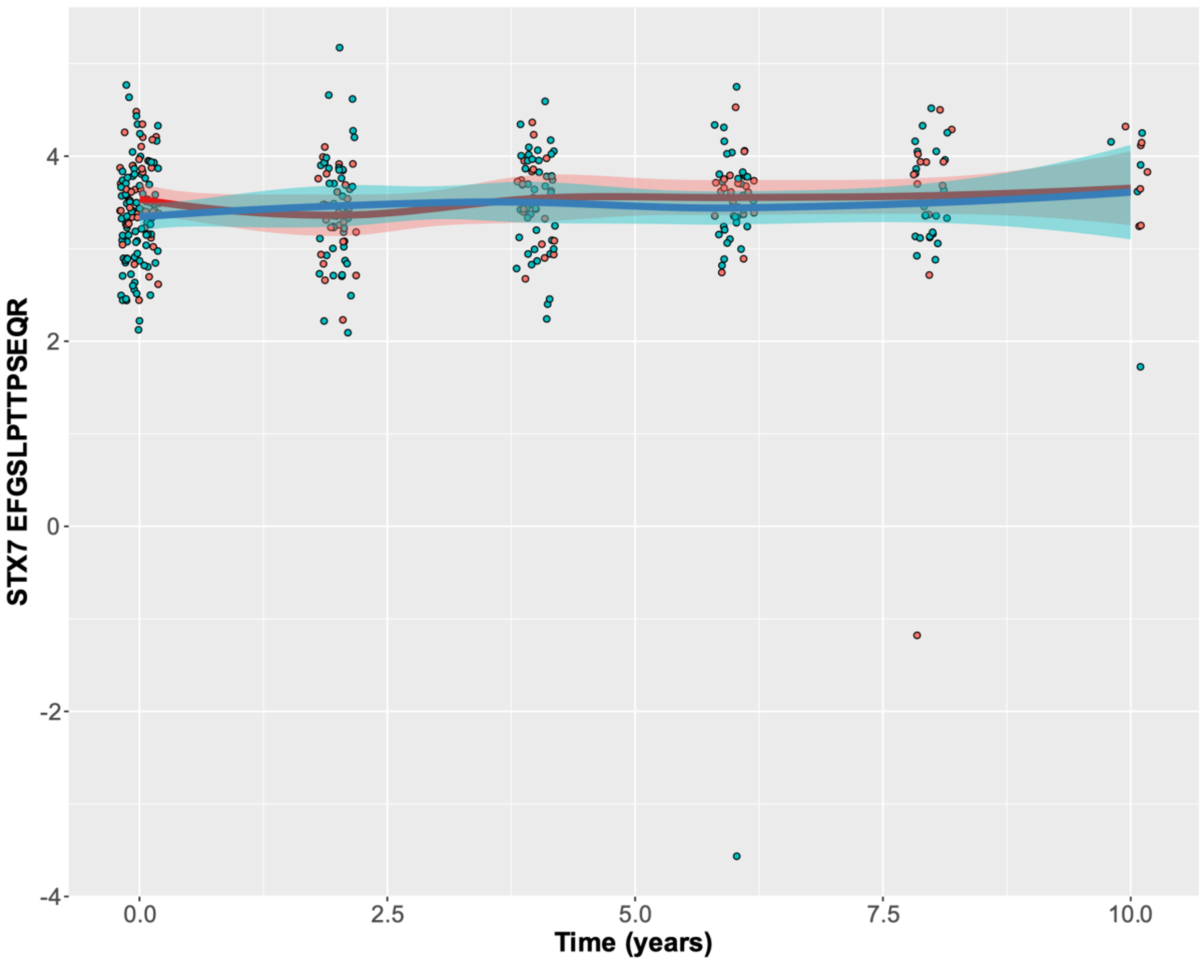


***Supplementary Figure 6q:*** *Longitudinal analysis of the cerebrospinal fluid levels over 10 years. The ribbon gives estimates of the standard error, the solid line gives a loose fit of the measured data points. Parkinson's disease: blue, healthy controls: red*

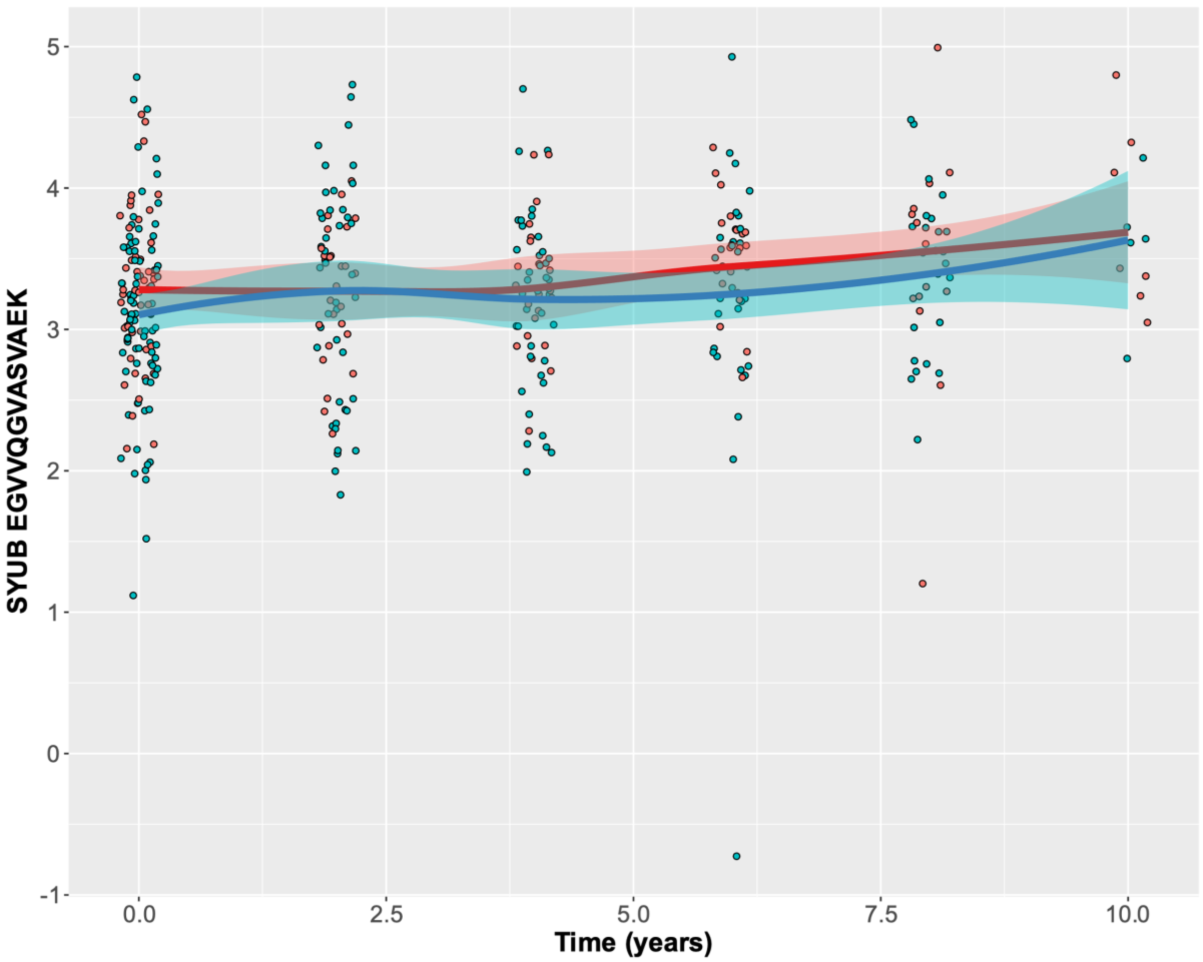


***Supplementary Figure 6r:*** *Longitudinal analysis of the cerebrospinal fluid levels over 10 years. The ribbon gives estimates of the standard error, the solid line gives a loose fit of the measured data points. Parkinson's disease: blue, healthy controls: red*

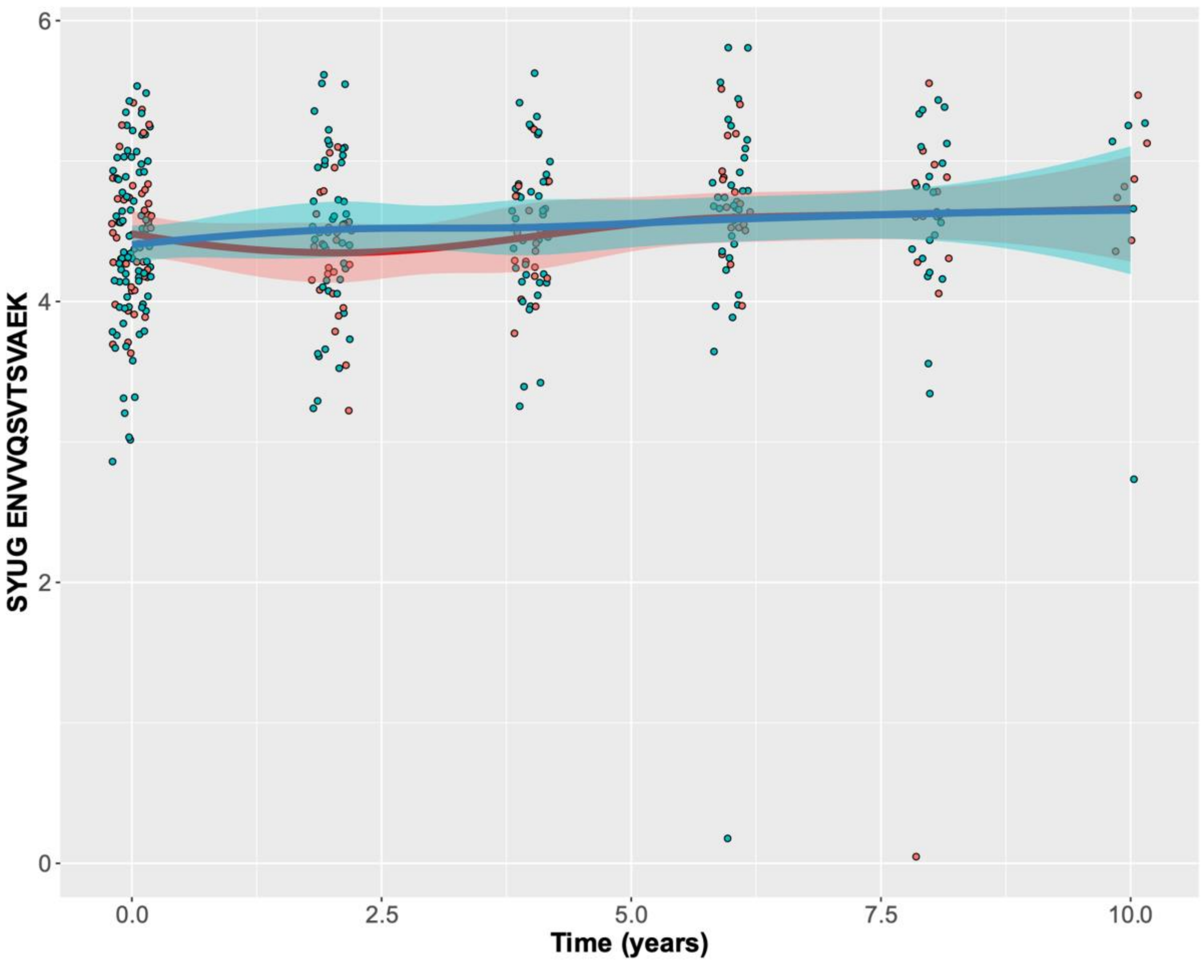


***Supplementary Figure 6s:*** *Longitudinal analysis of the cerebrospinal fluid levels over 10 years. The ribbon gives estimates of the standard error, the solid line gives a loose fit of the measured data points. Parkinson's disease: blue, healthy controls: red*

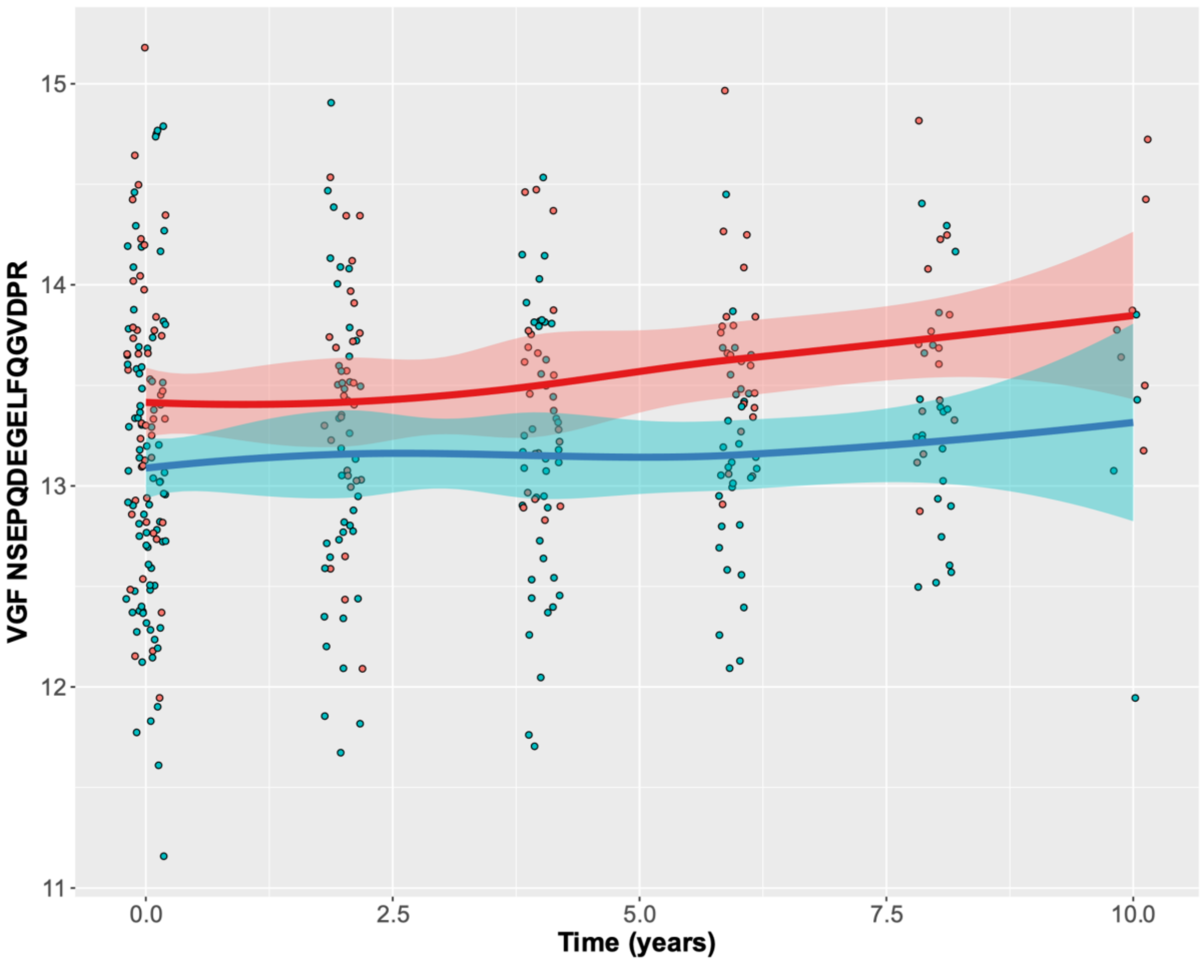


***Supplementary Figure 6t:*** *Longitudinal analysis of the cerebrospinal fluid levels over 10 years. The ribbon gives estimates of the standard error, the solid line gives a loose fit of the measured data points. Parkinson's disease: blue, healthy controls: red*

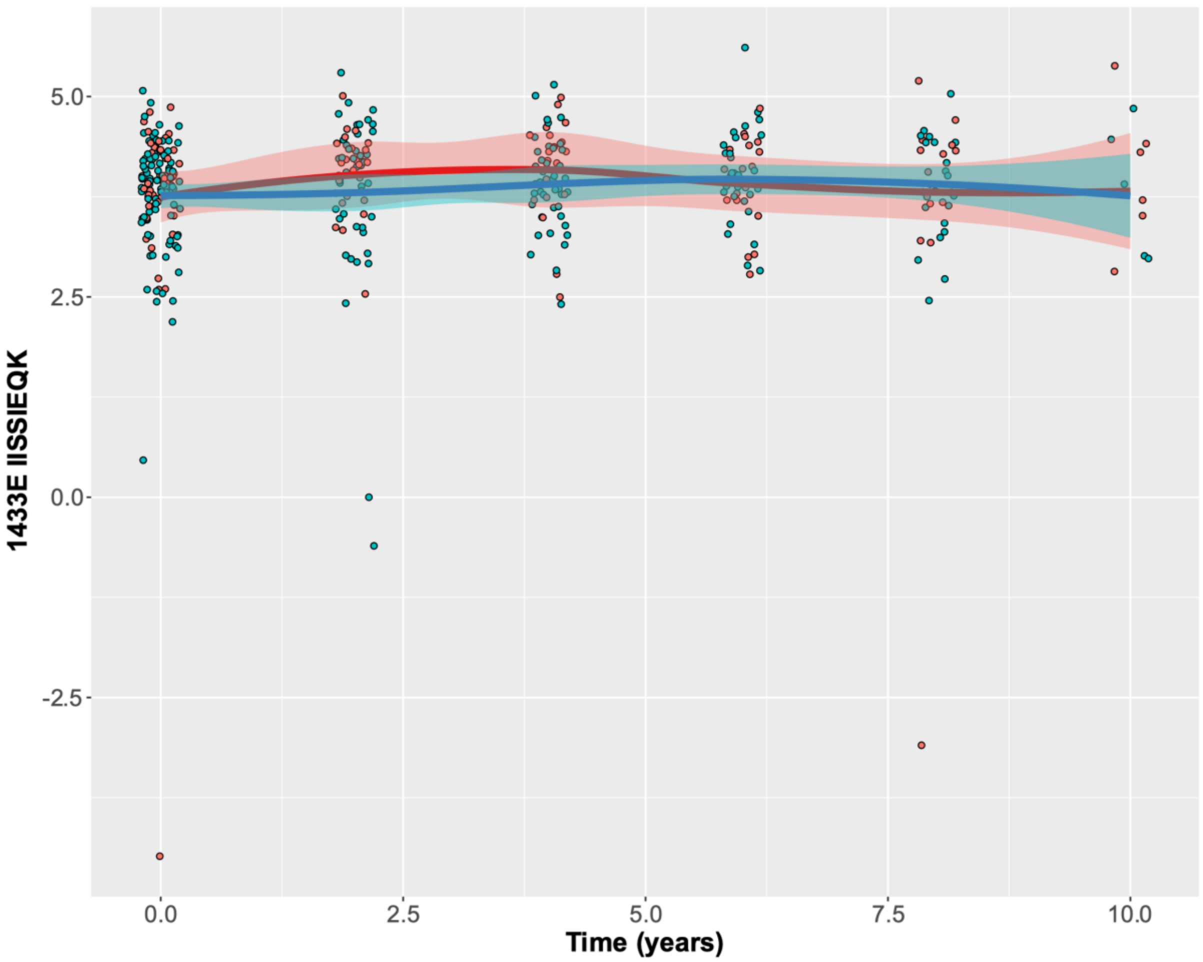


***Supplementary Figure 6u:*** *Longitudinal analysis of the cerebrospinal fluid levels over 10 years. The ribbon gives estimates of the standard error, the solid line gives a loose fit of the measured data points. Parkinson's disease: blue, healthy controls: red*

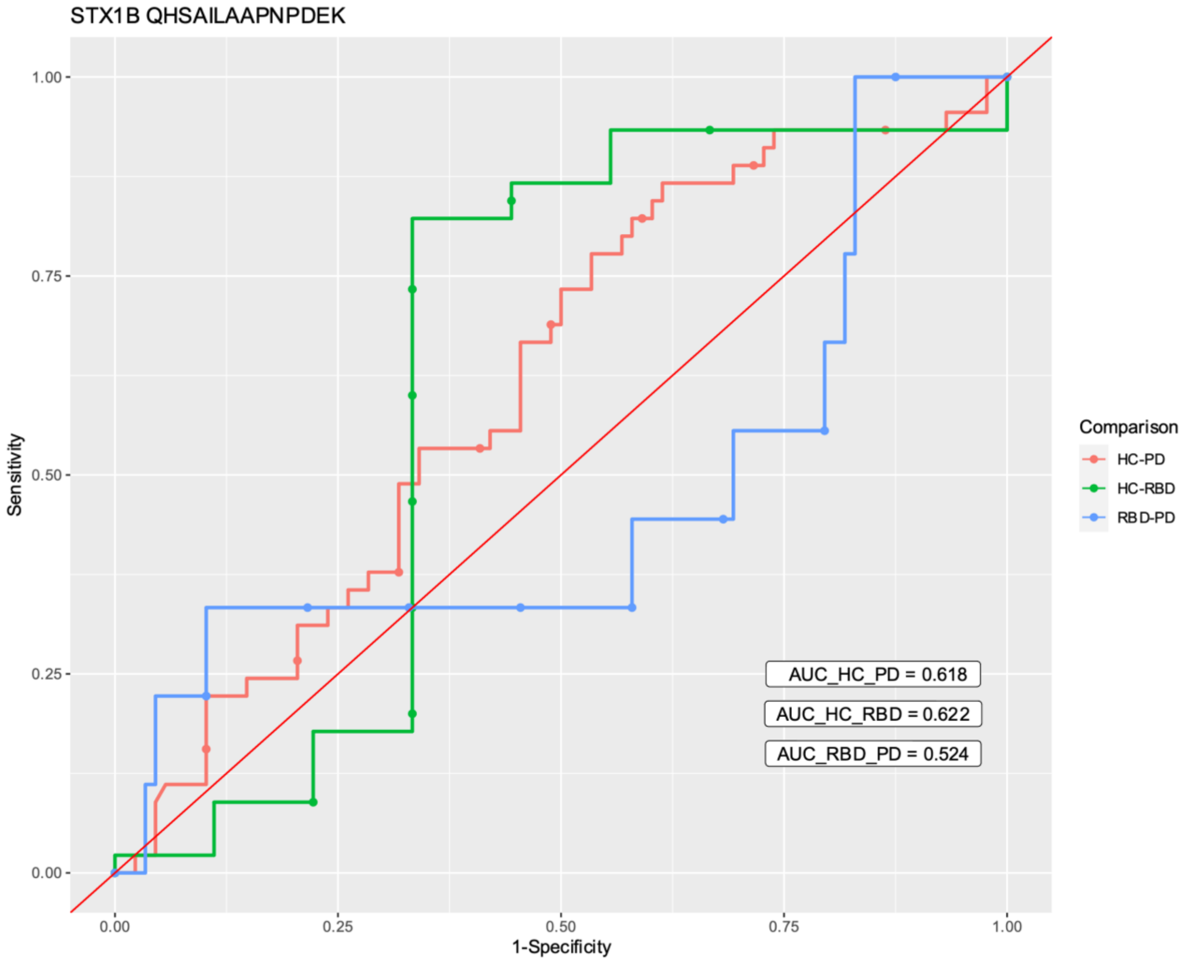


***Supplementary Figure 7a****: Results of the discrimination analysis based on receiver operating characteristics (ROC)*

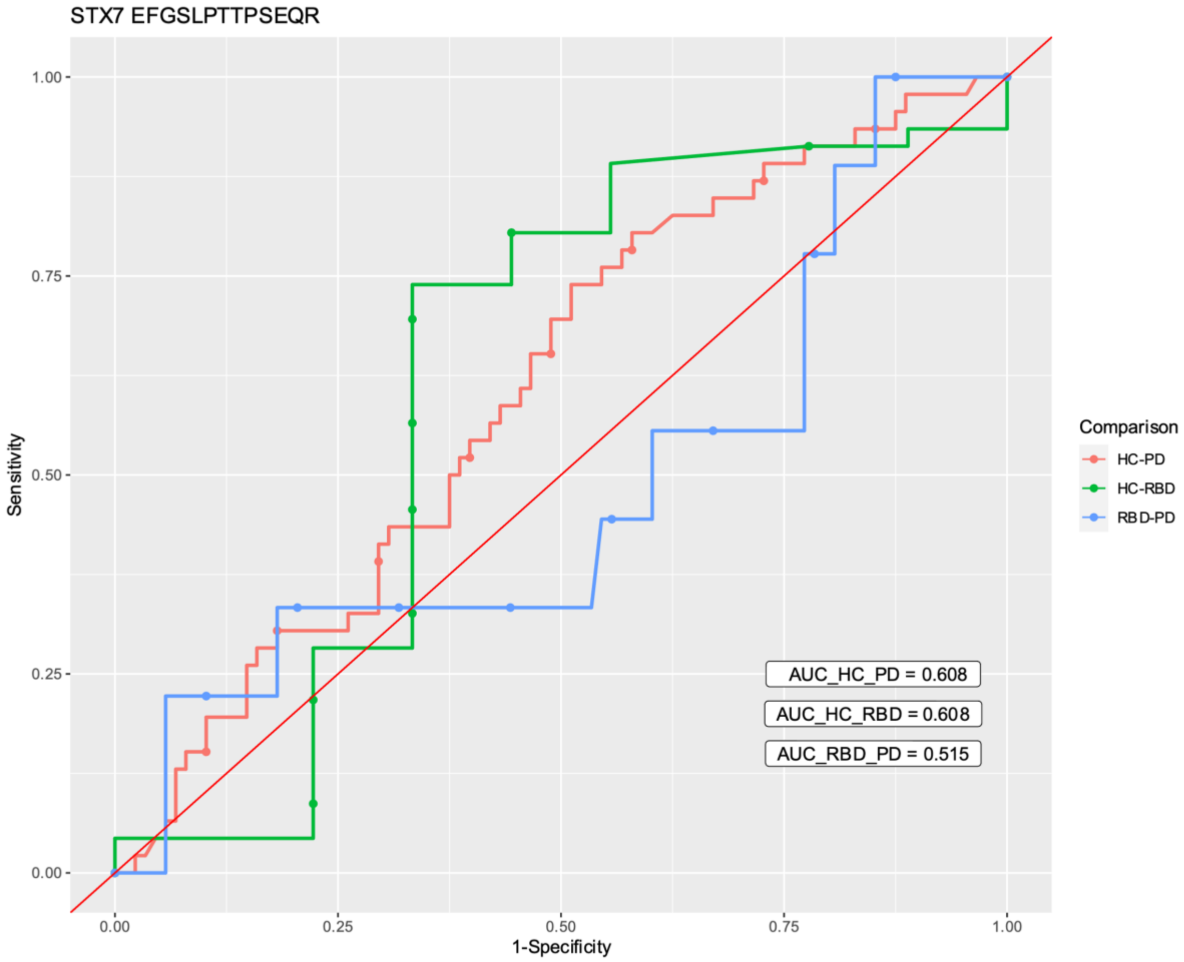


***Supplementary Figure 7b***: *Results of the discrimination analysis based on receiver operating characteristics (ROC)*

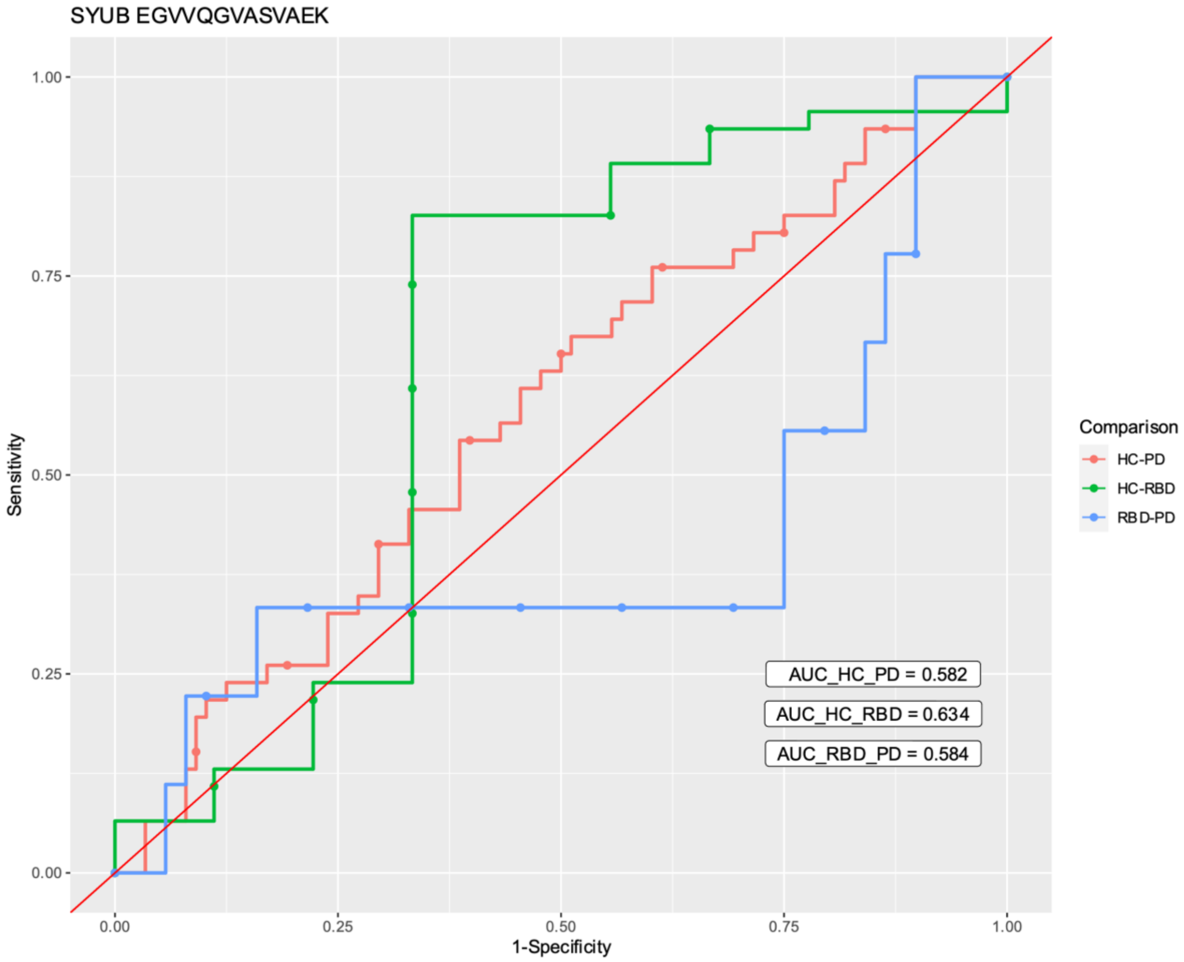


***Supplementary Figure 7c****: Results of the discrimination analysis based on receiver operating characteristics (ROC)*

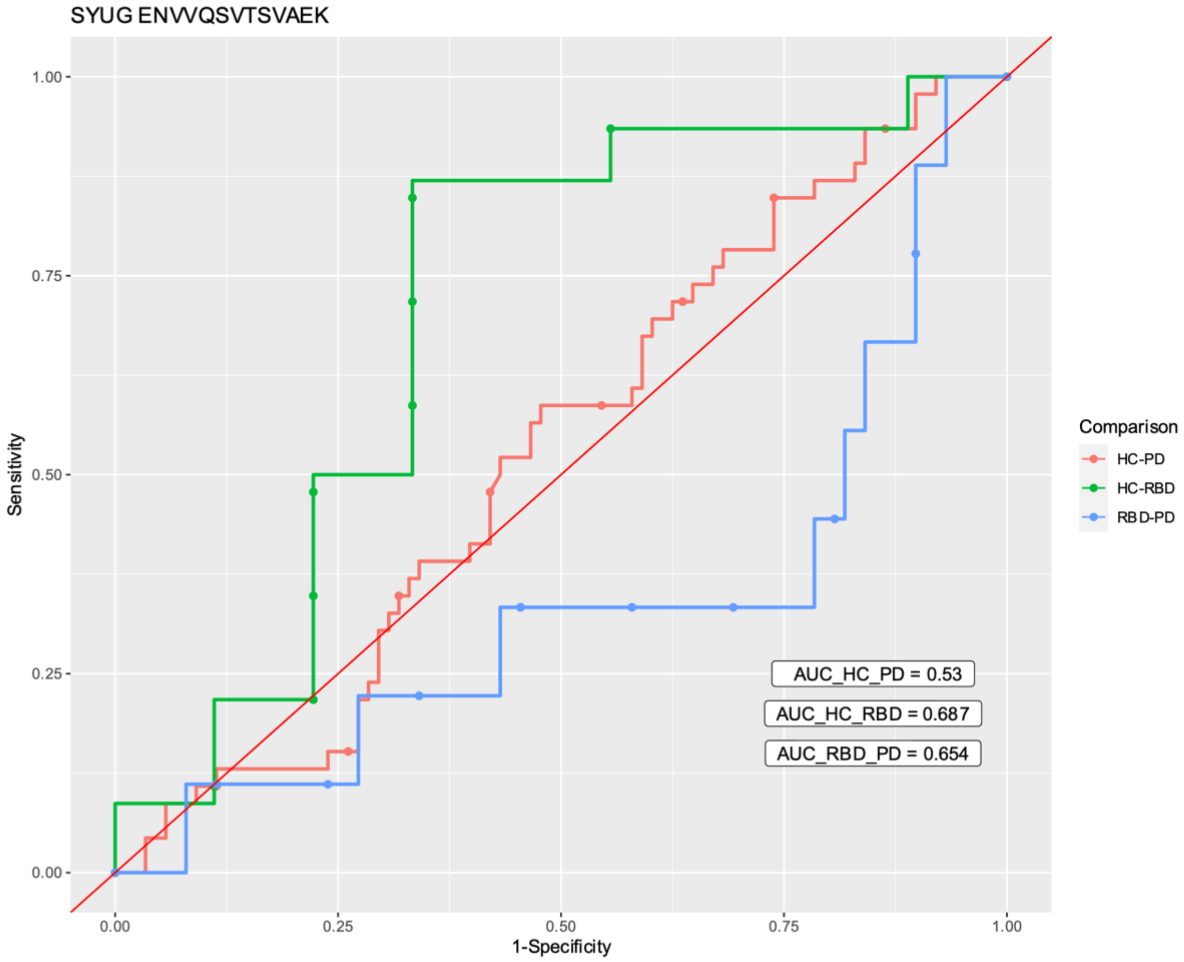


***Supplementary Figure 7d***: *Results of the discrimination analysis based on receiver operating characteristics (ROC)*

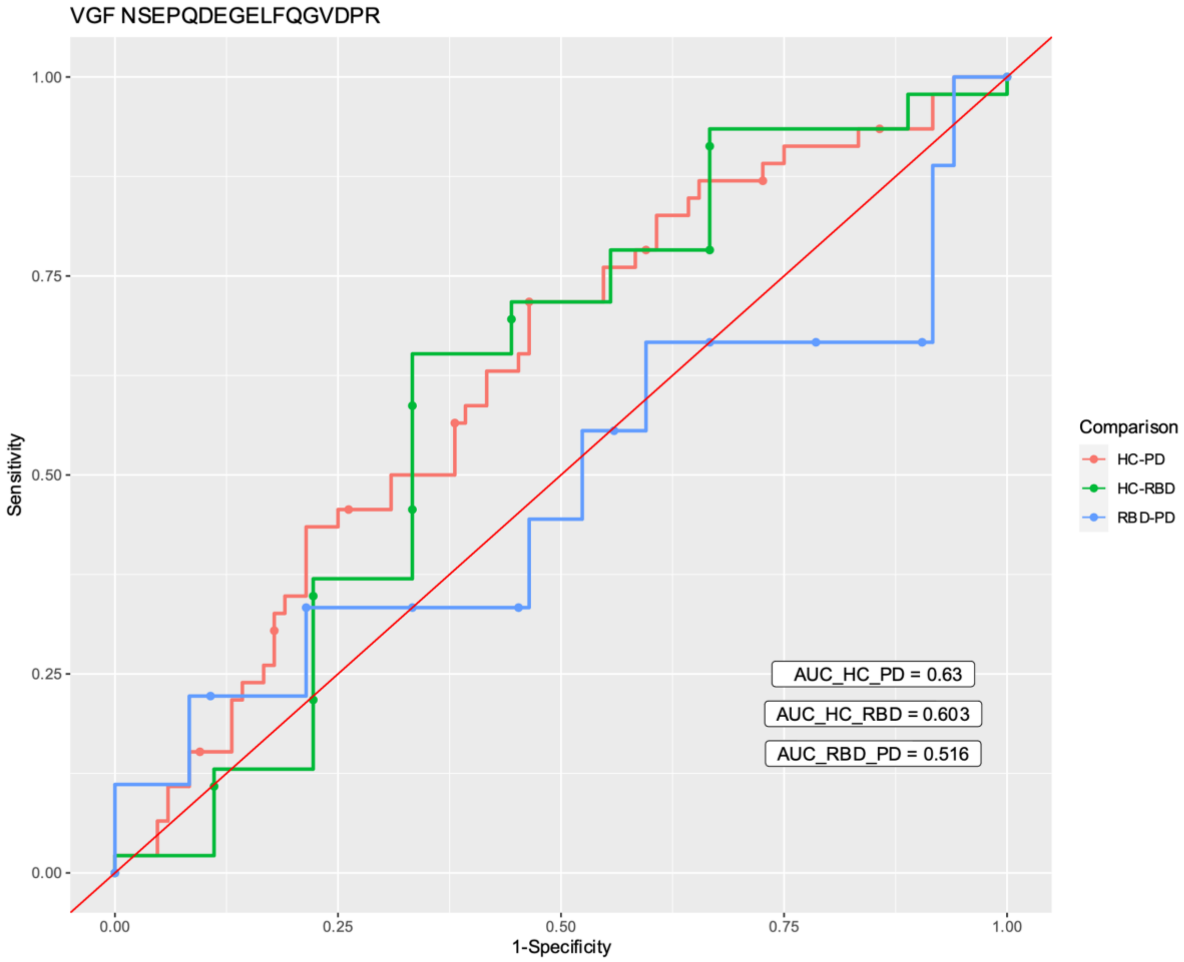


***Supplementary Figure 7e***: *Results of the discrimination analysis based on receiver operating characteristics (ROC)*

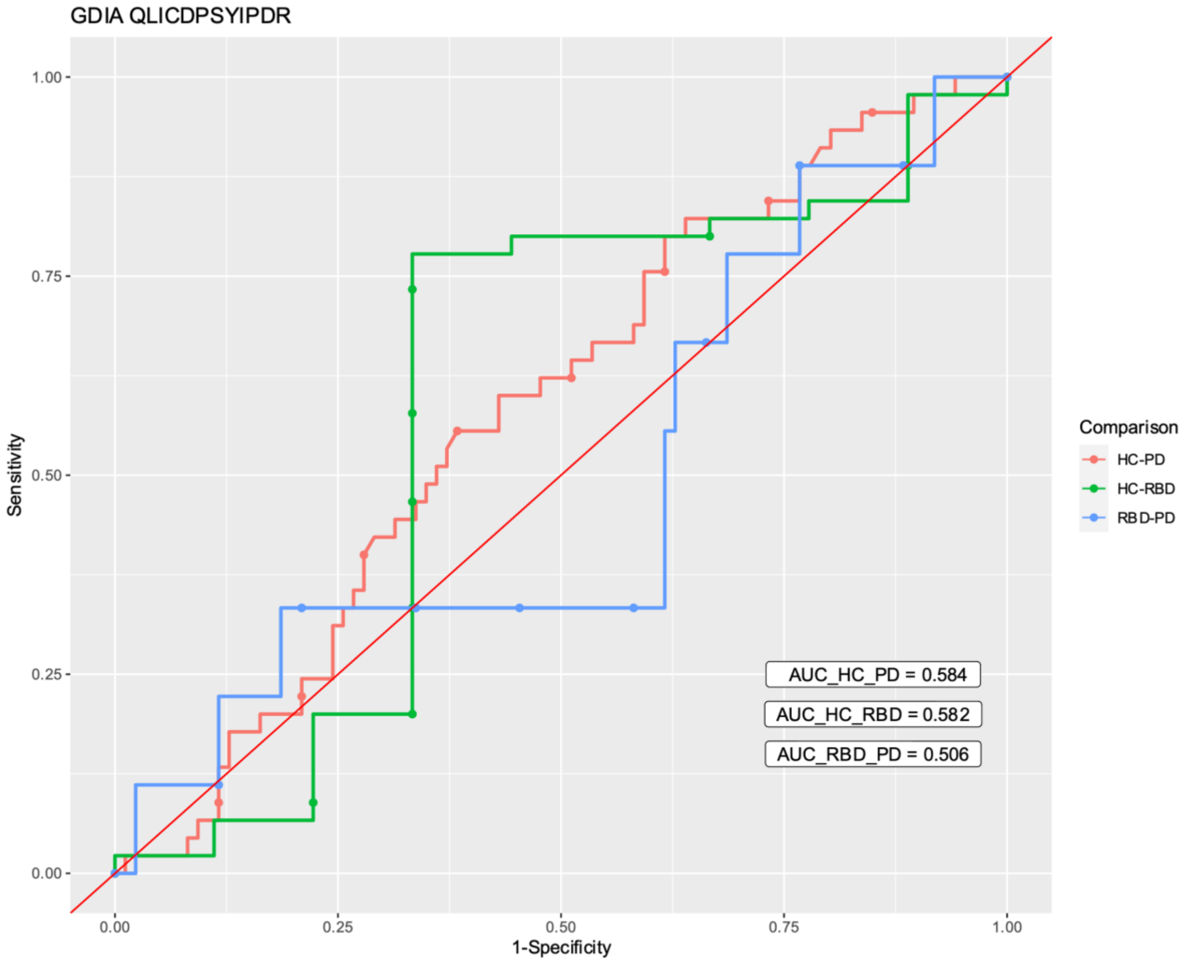


***Supplementary Figure 7f***: *Results of the discrimination analysis based on receiver operating characteristics (ROC)*

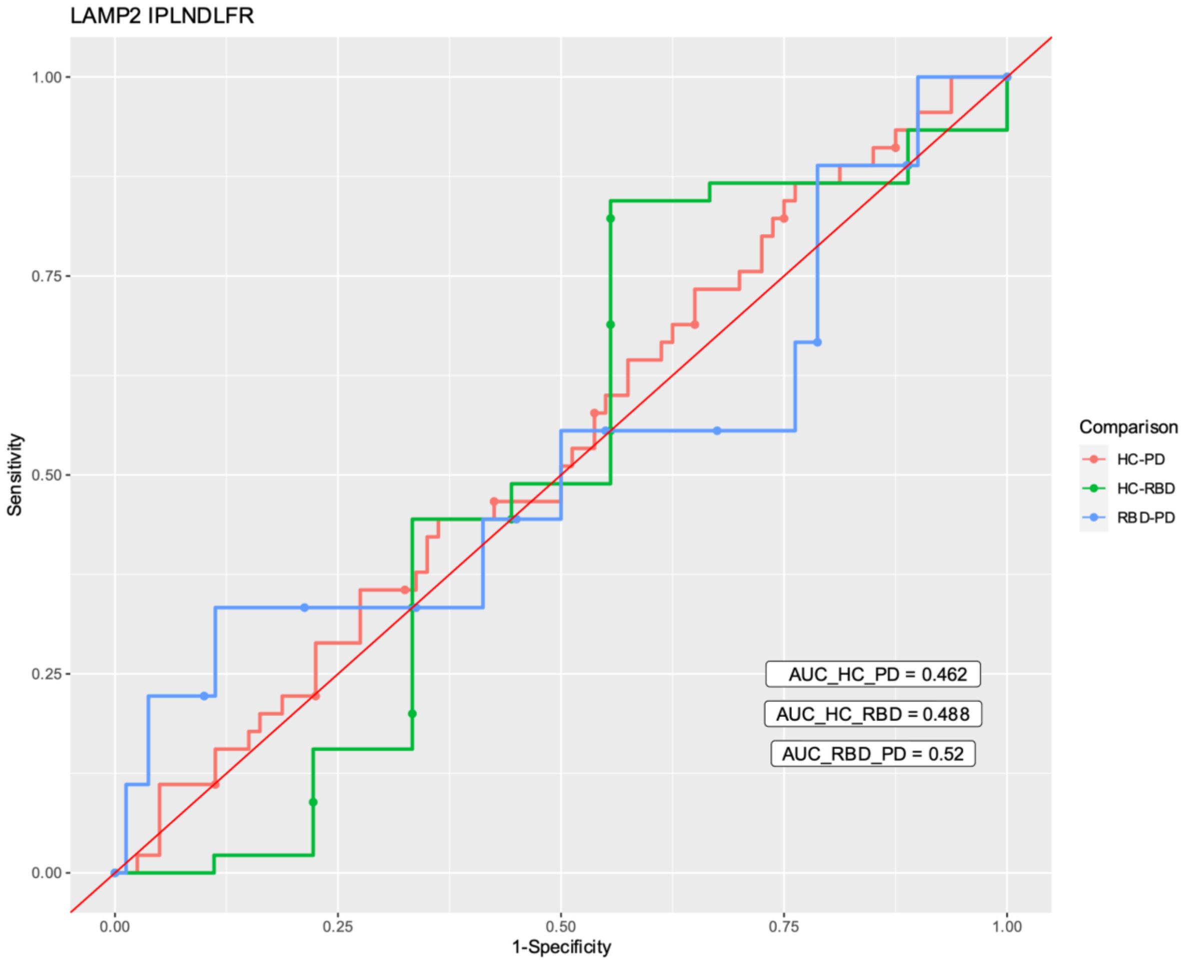


***Supplementary Figure 7g****: Results of the discrimination analysis based on receiver operating characteristics (ROC)*

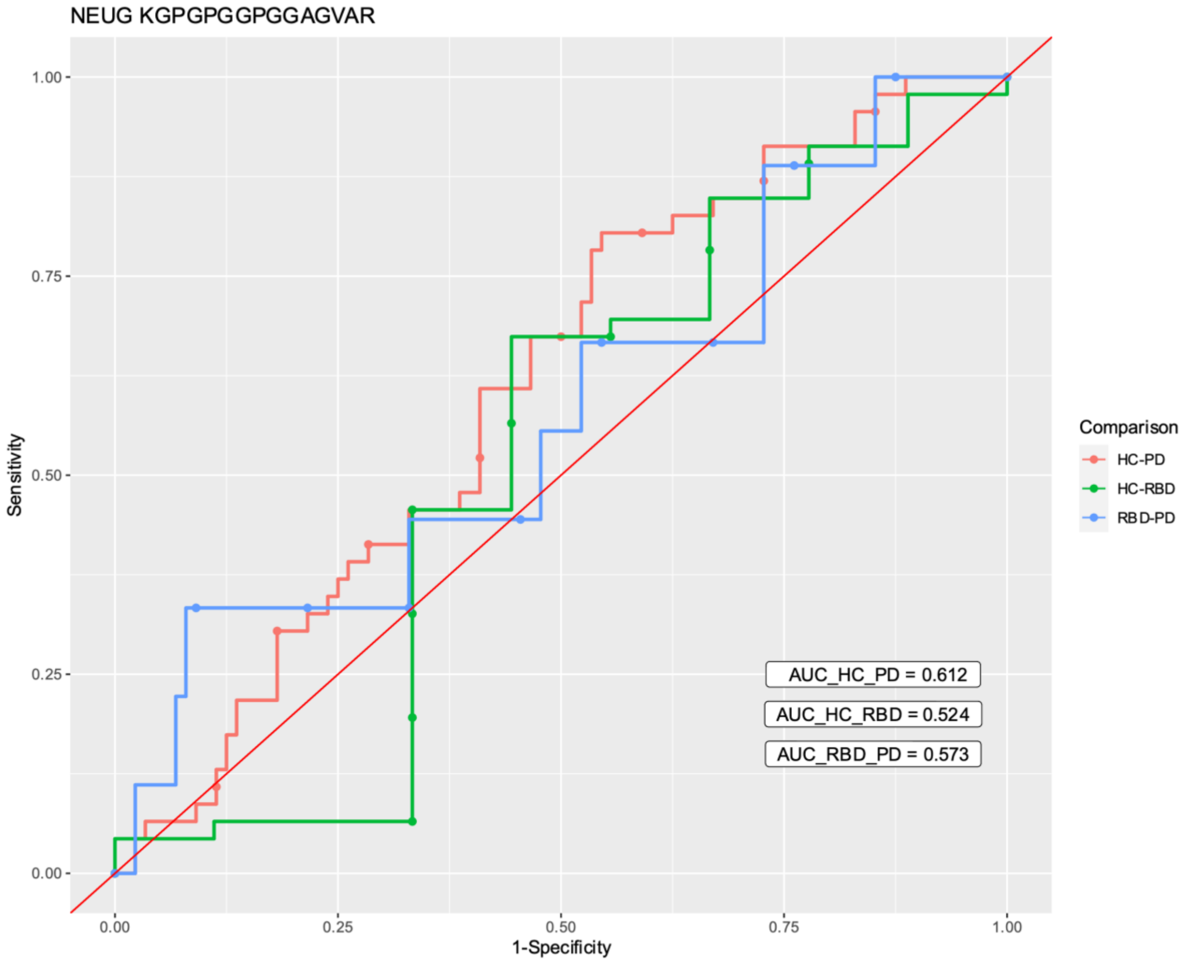


***Supplementary Figure 7h***: *Results of the discrimination analysis based on receiver operating characteristics (ROC)*

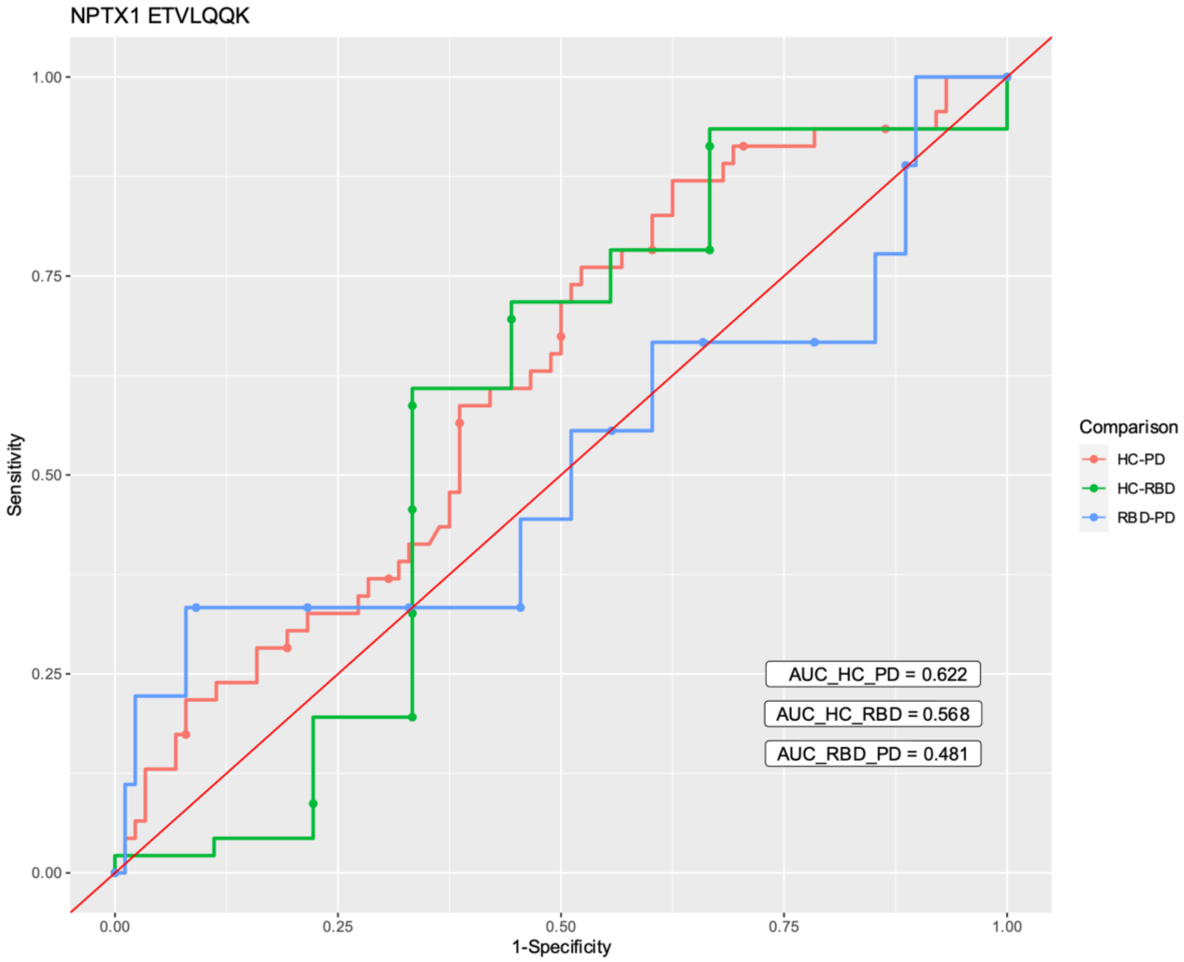


***Supplementary Figure 7i****: Results of the discrimination analysis based on receiver operating characteristics (ROC)*

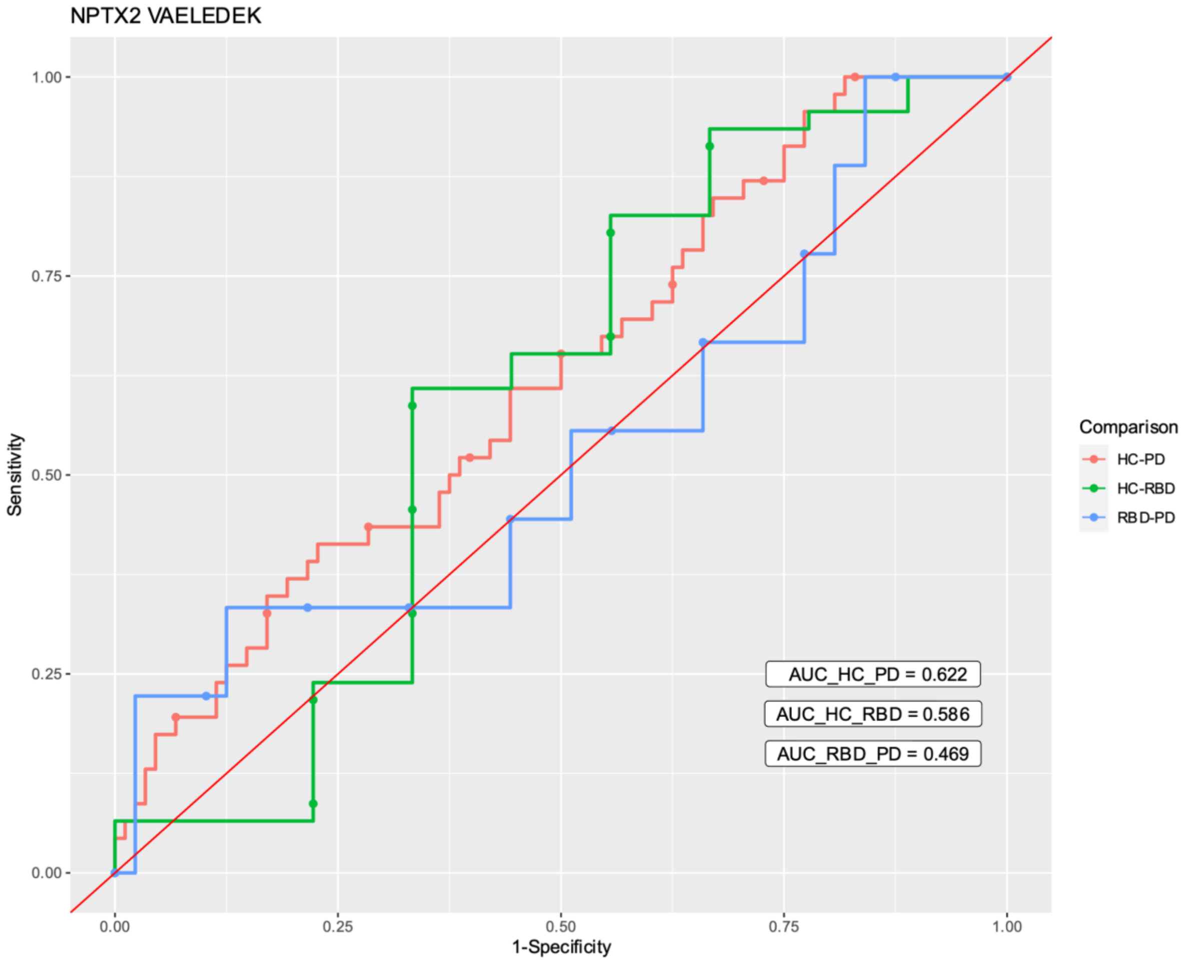


***Supplementary Figure 7j***: *Results of the discrimination analysis based on receiver operating characteristics (ROC)*

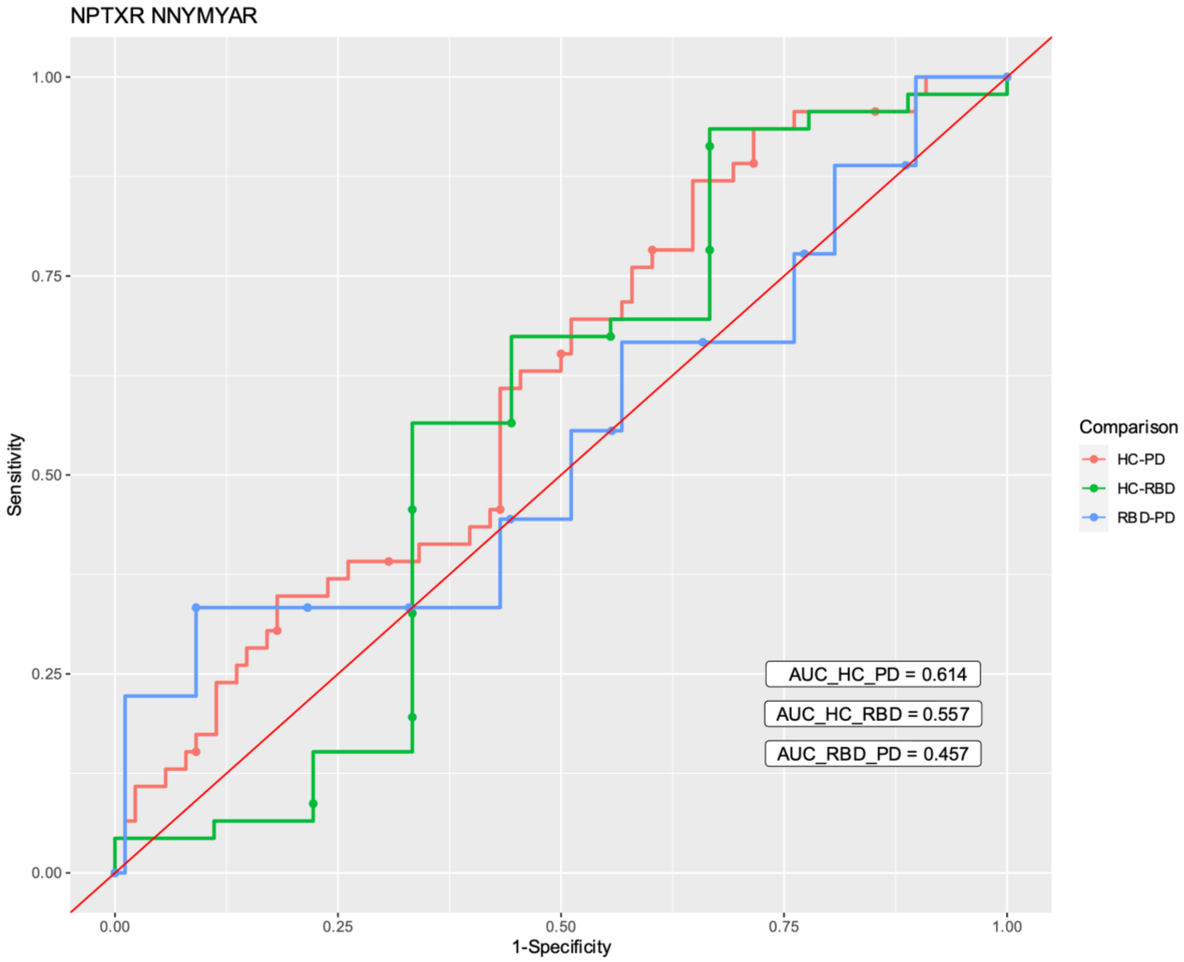


***Supplementary Figure 7k***: *Results of the discrimination analysis based on receiver operating characteristics (ROC)*

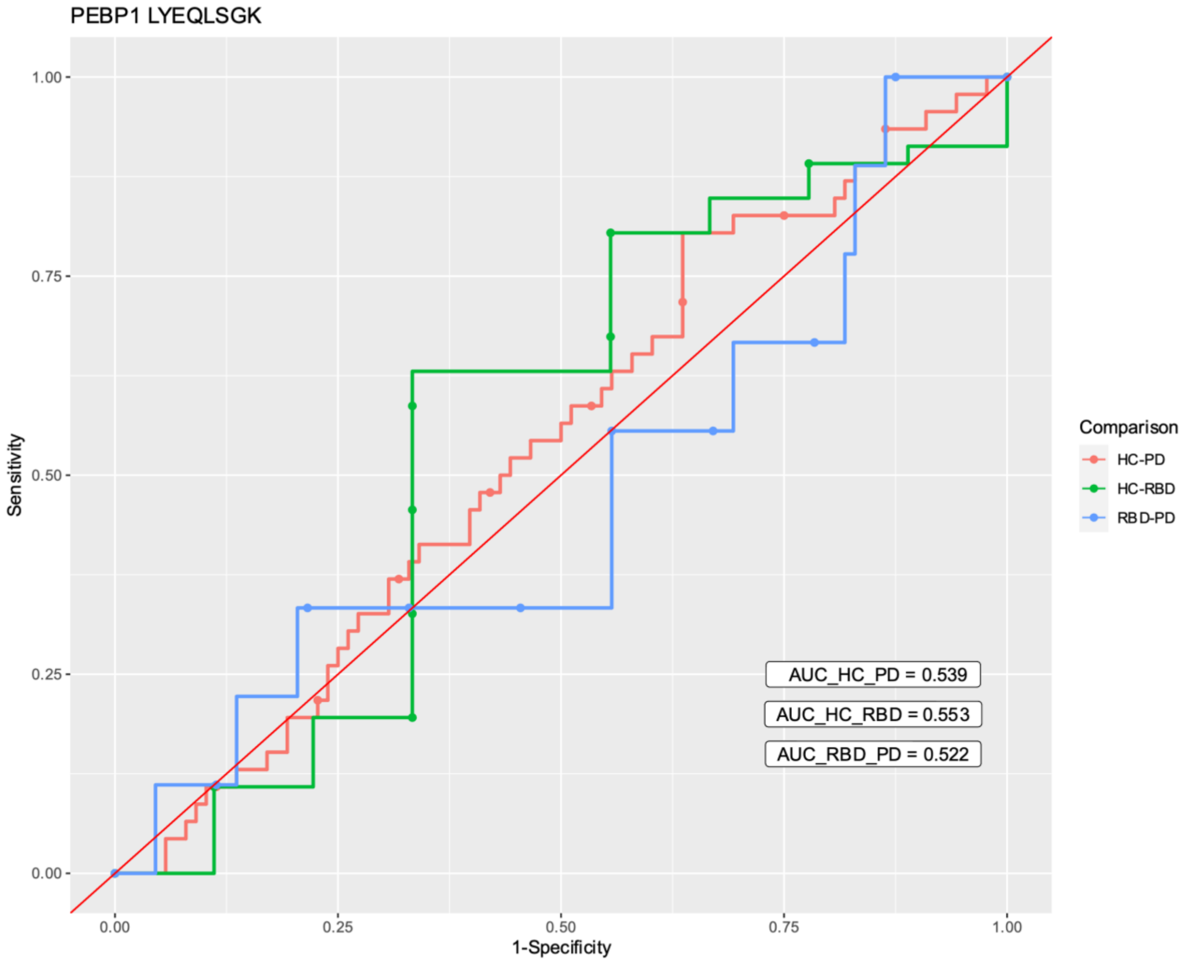


***Supplementary Figure 7l***: *Results of the discrimination analysis based on receiver operating characteristics (ROC)*

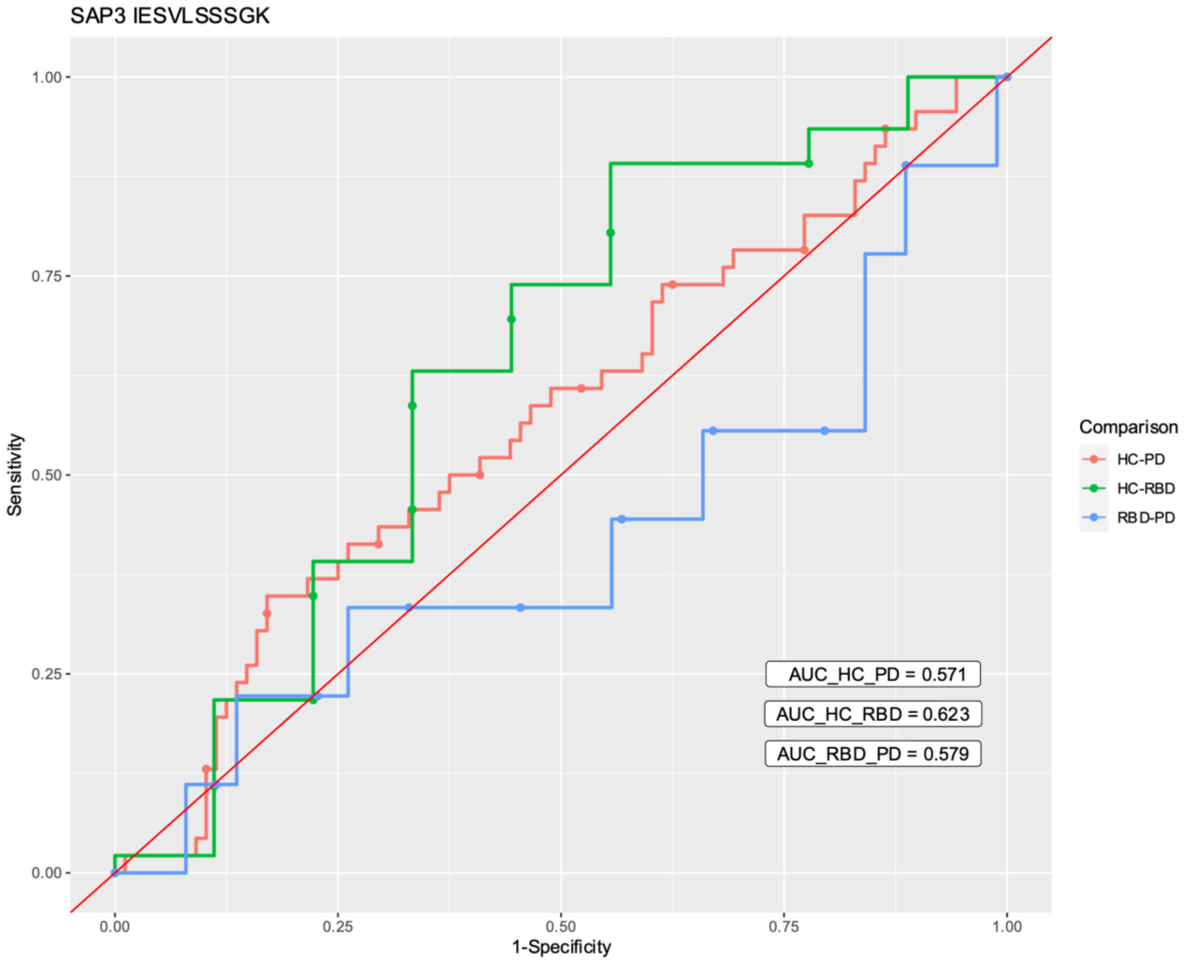


***Supplementary Figure 7m***: *Results of the discrimination analysis based on receiver operating characteristics (ROC)*

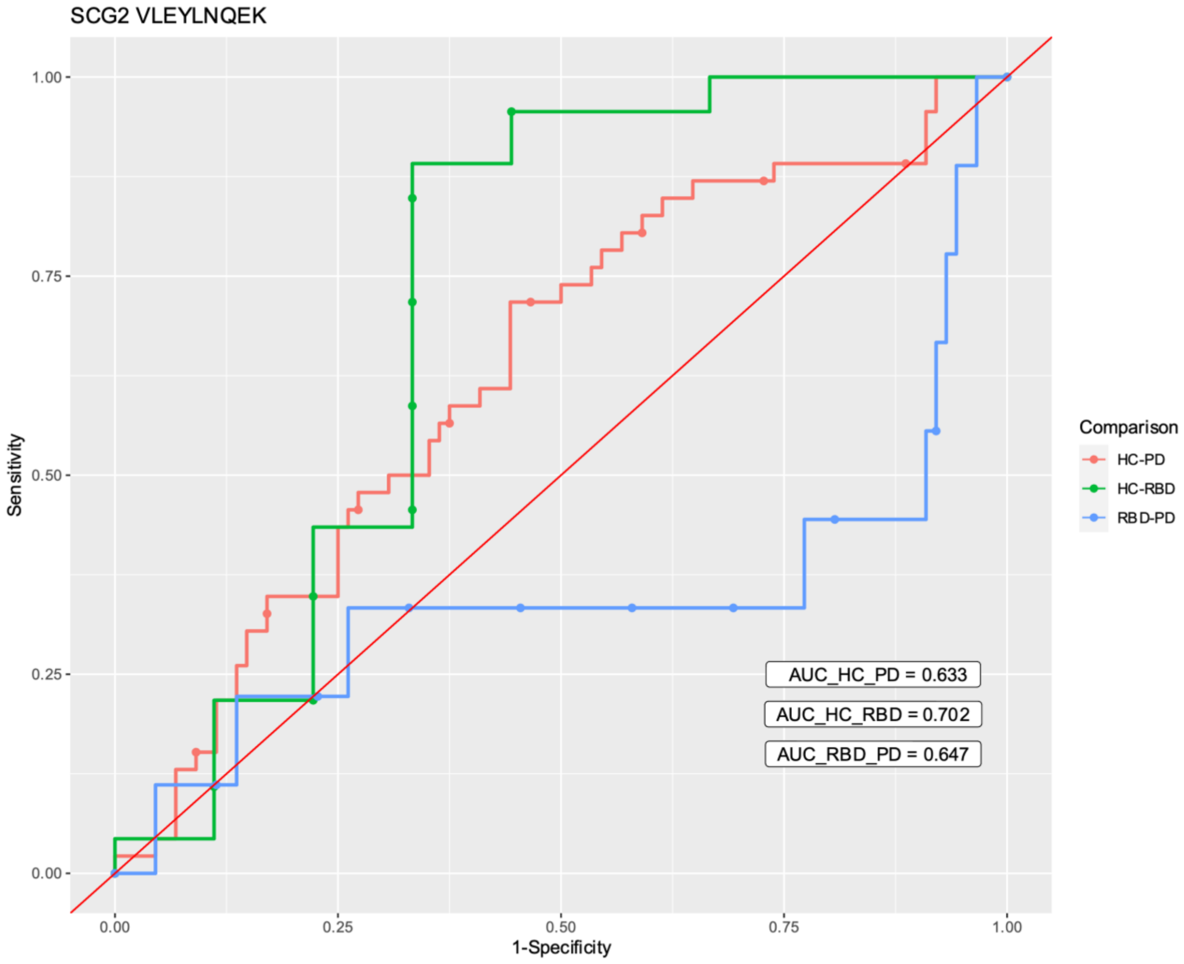


***Supplementary Figure 7n***: *Results of the discrimination analysis based on receiver operating characteristics (ROC)*

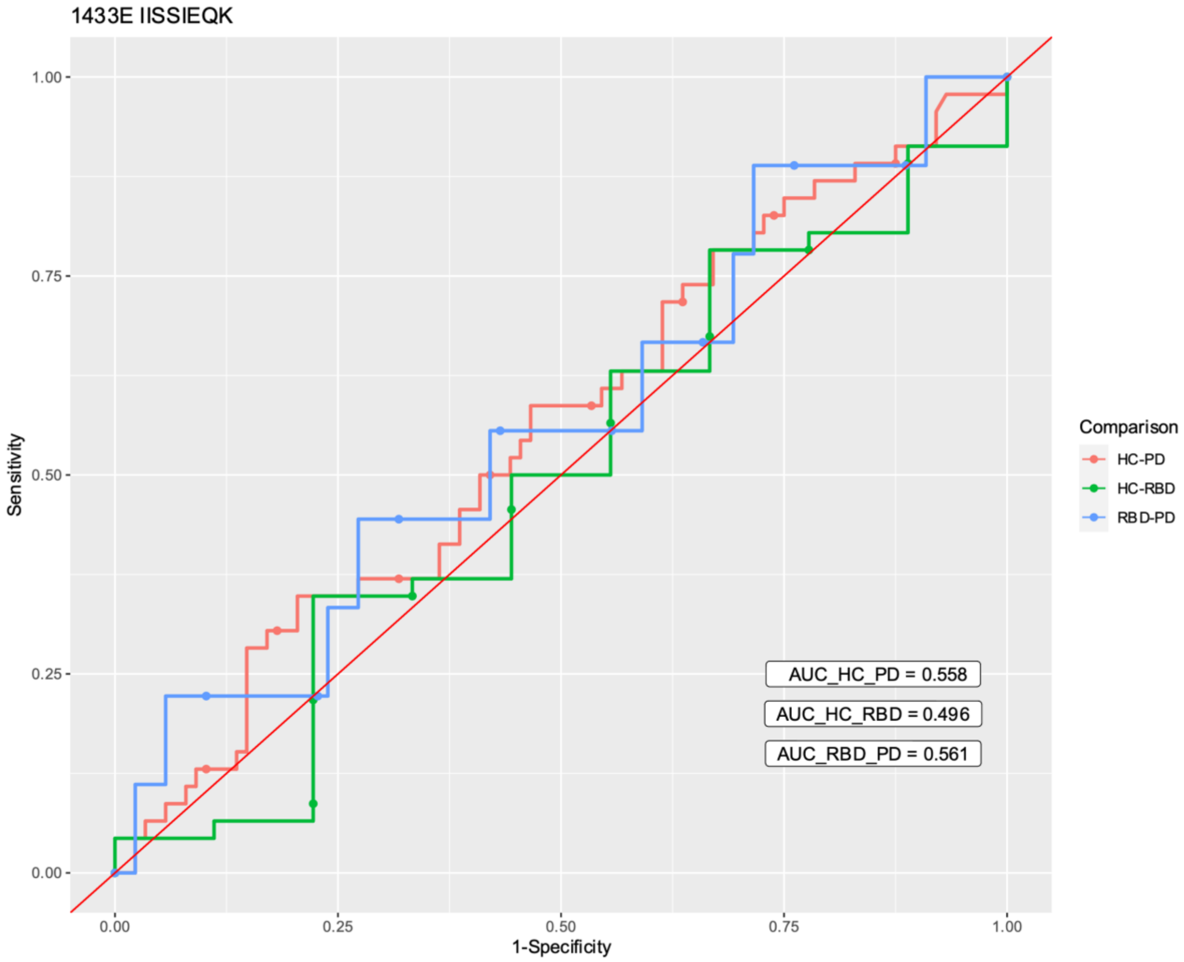


***Supplementary Figure 7o***: *Results of the discrimination analysis based on receiver operating characteristics (ROC)*

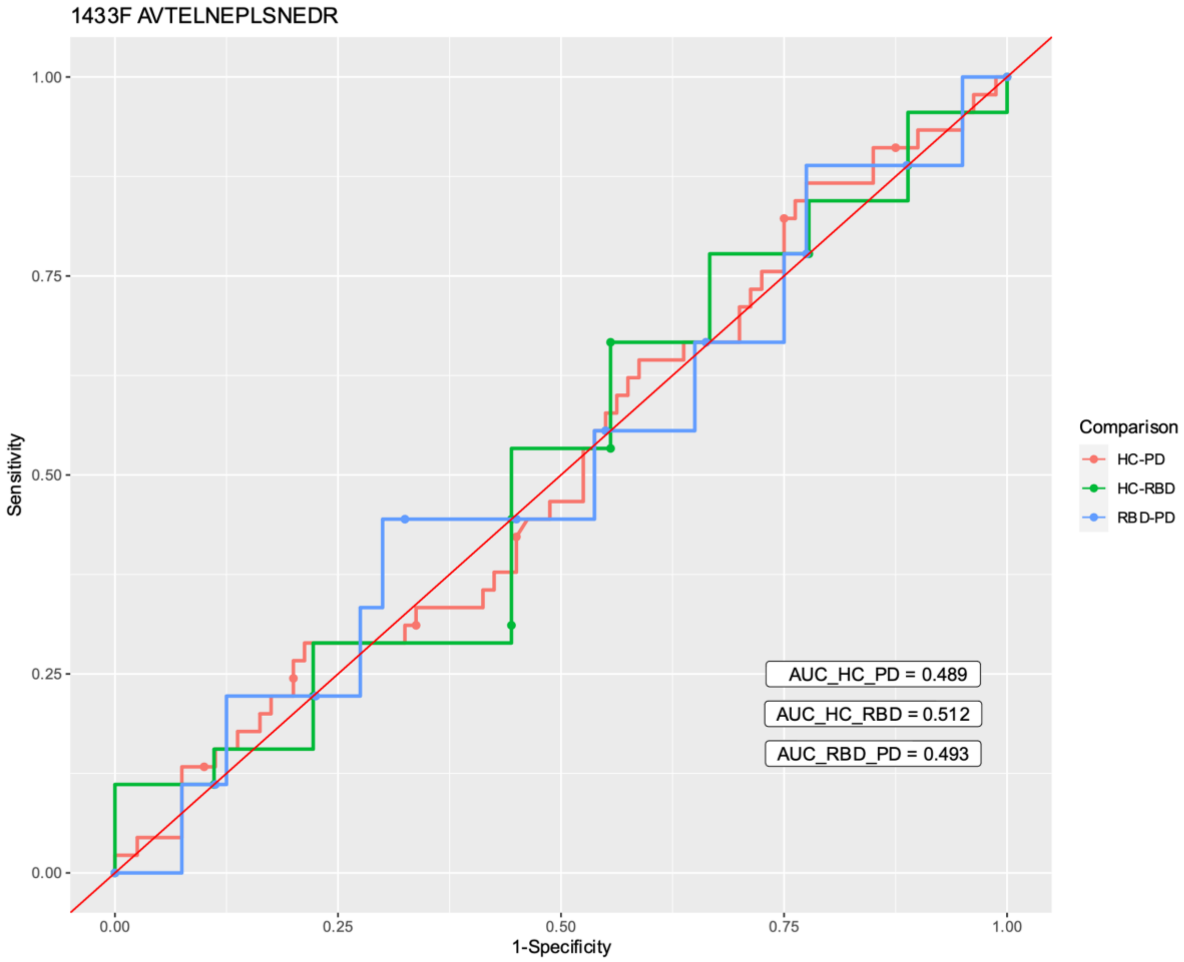


***Supplementary Figure 7p***: *Results of the discrimination analysis based on receiver operating characteristics (ROC)*

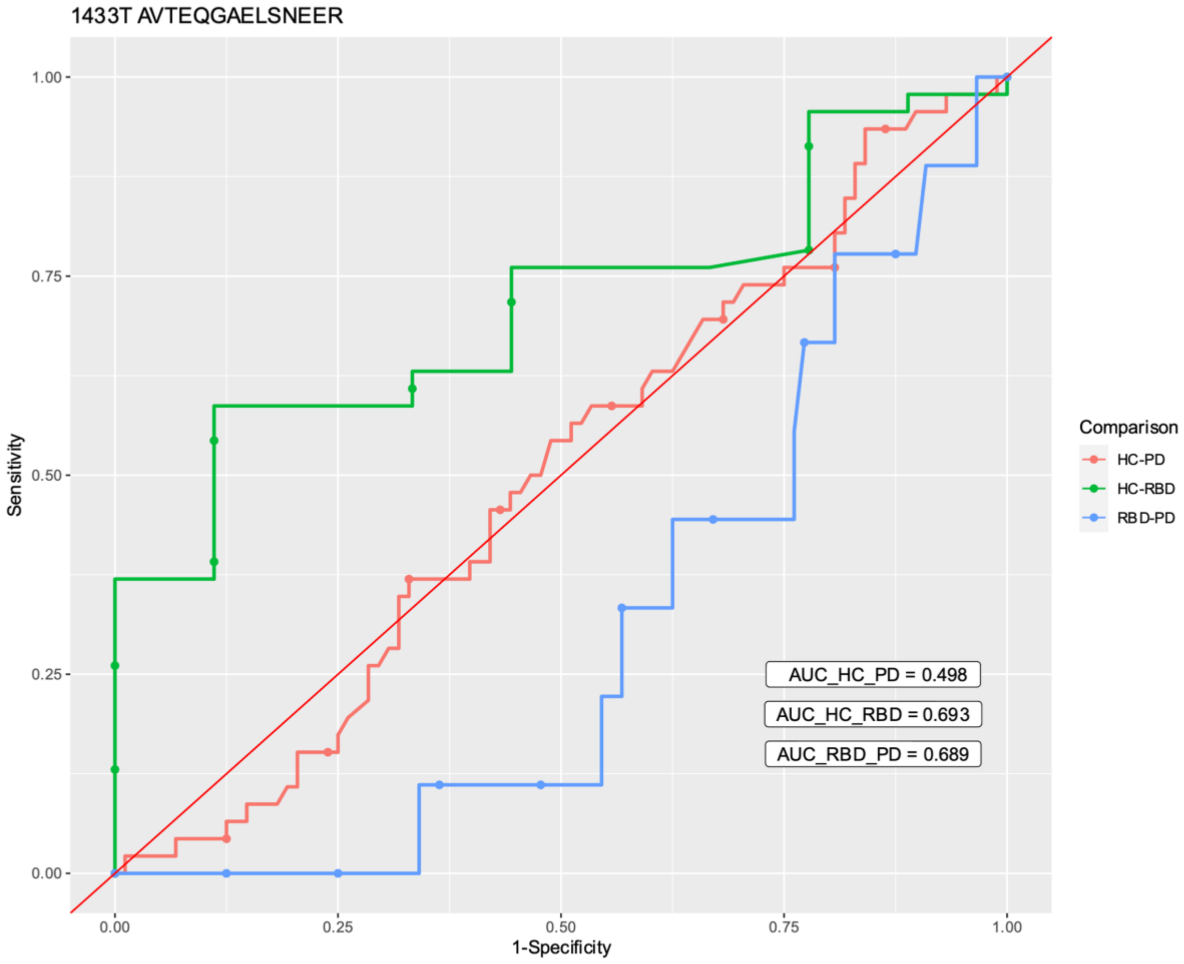


***Supplementary Figure 7q***: *Results of the discrimination analysis based on receiver operating characteristics (ROC)*

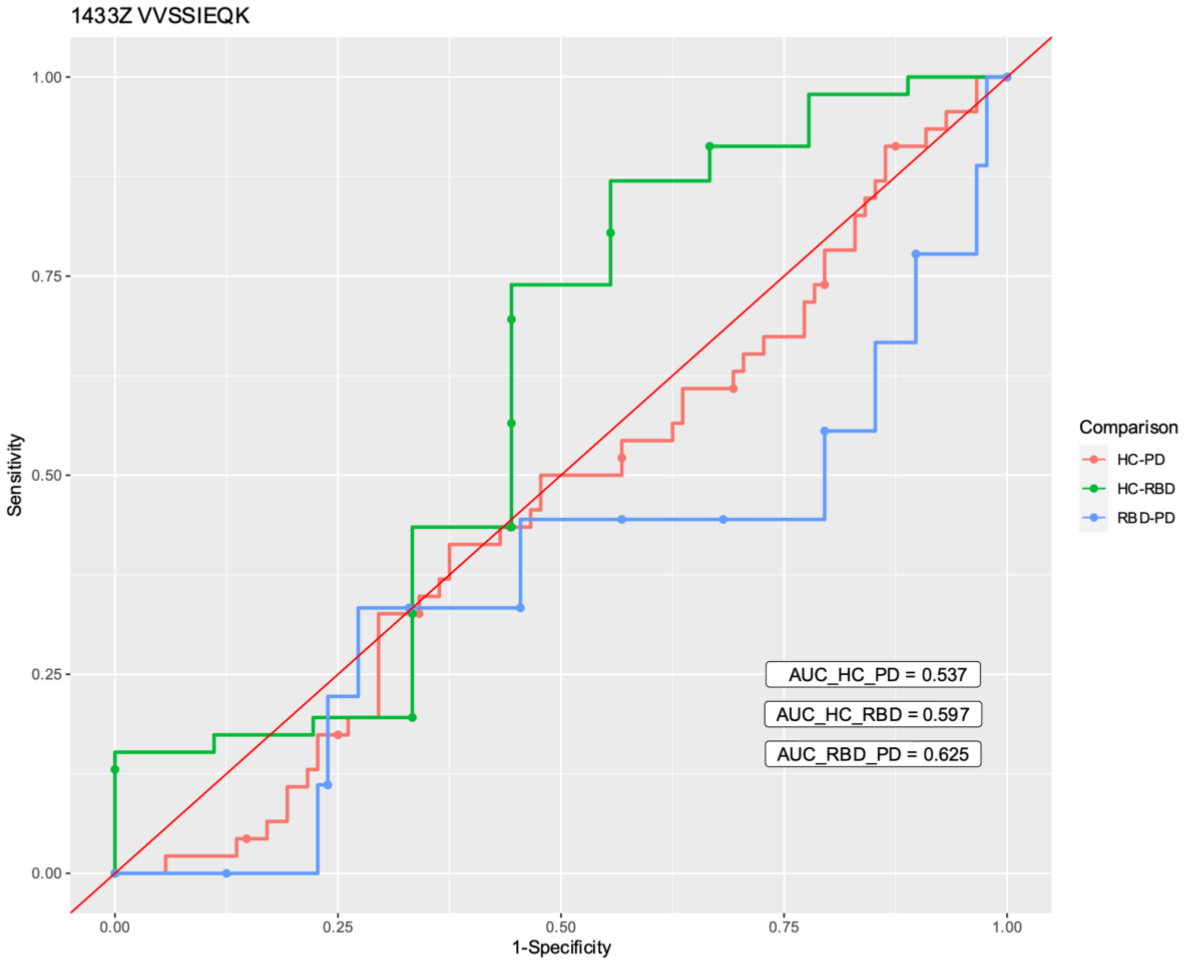


***Supplementary Figure 7r**: Results of the discrimination analysis based on receiver operating characteristics (ROC)*

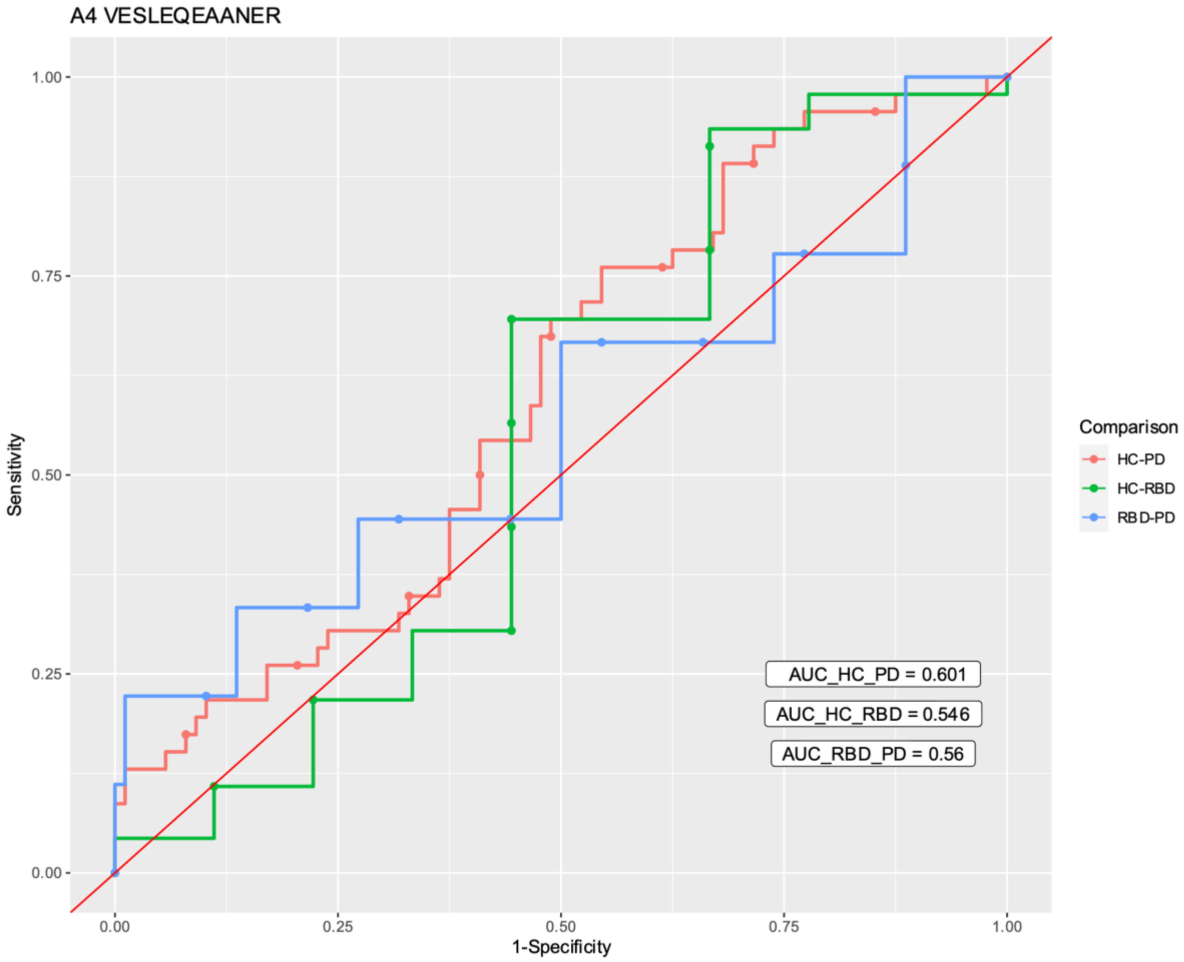


***Supplementary Figure 7s****: Results of the discrimination analysis based on receiver operating characteristics (ROC)*

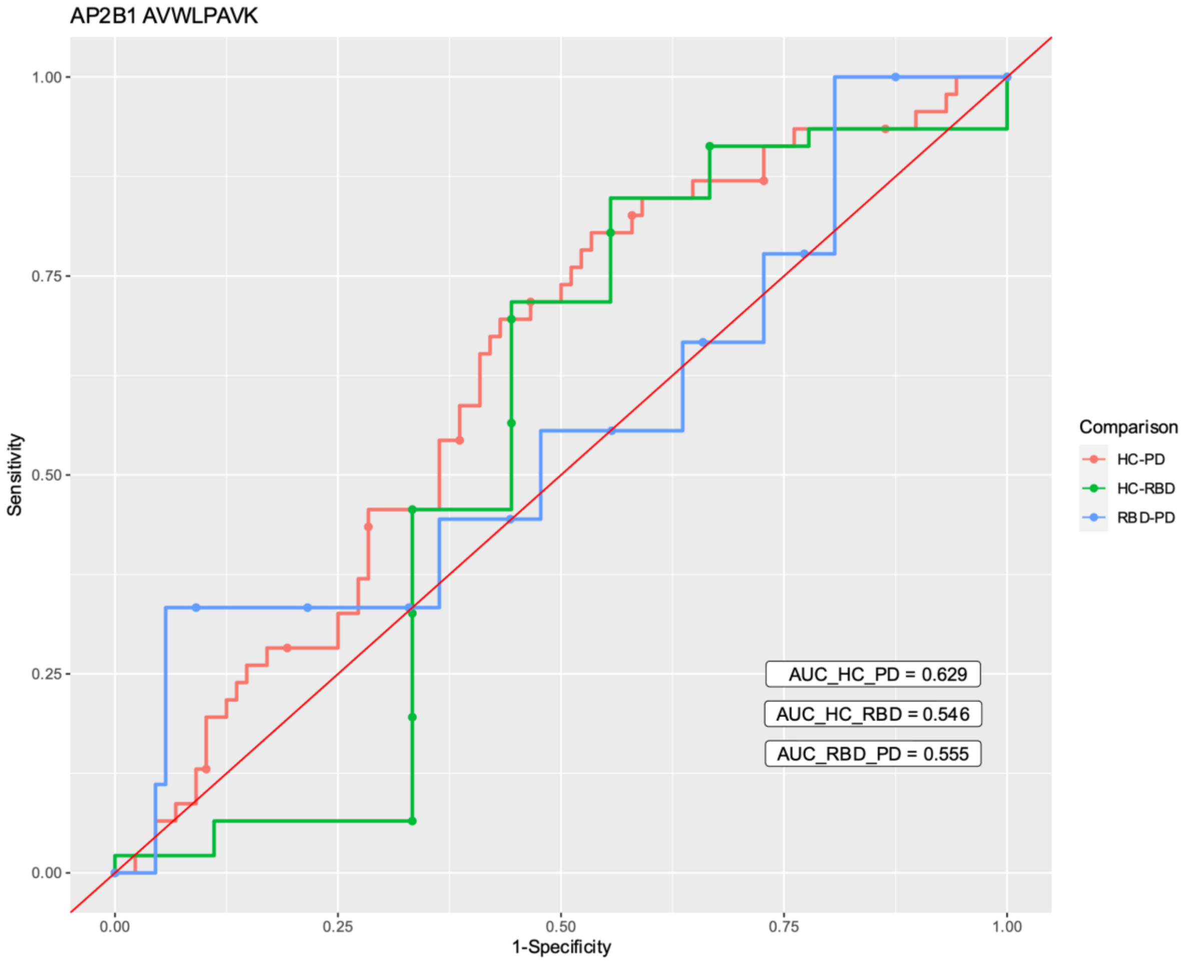


***Supplementary Figure 7t***: *Results of the discrimination analysis based on receiver operating characteristics (ROC)*

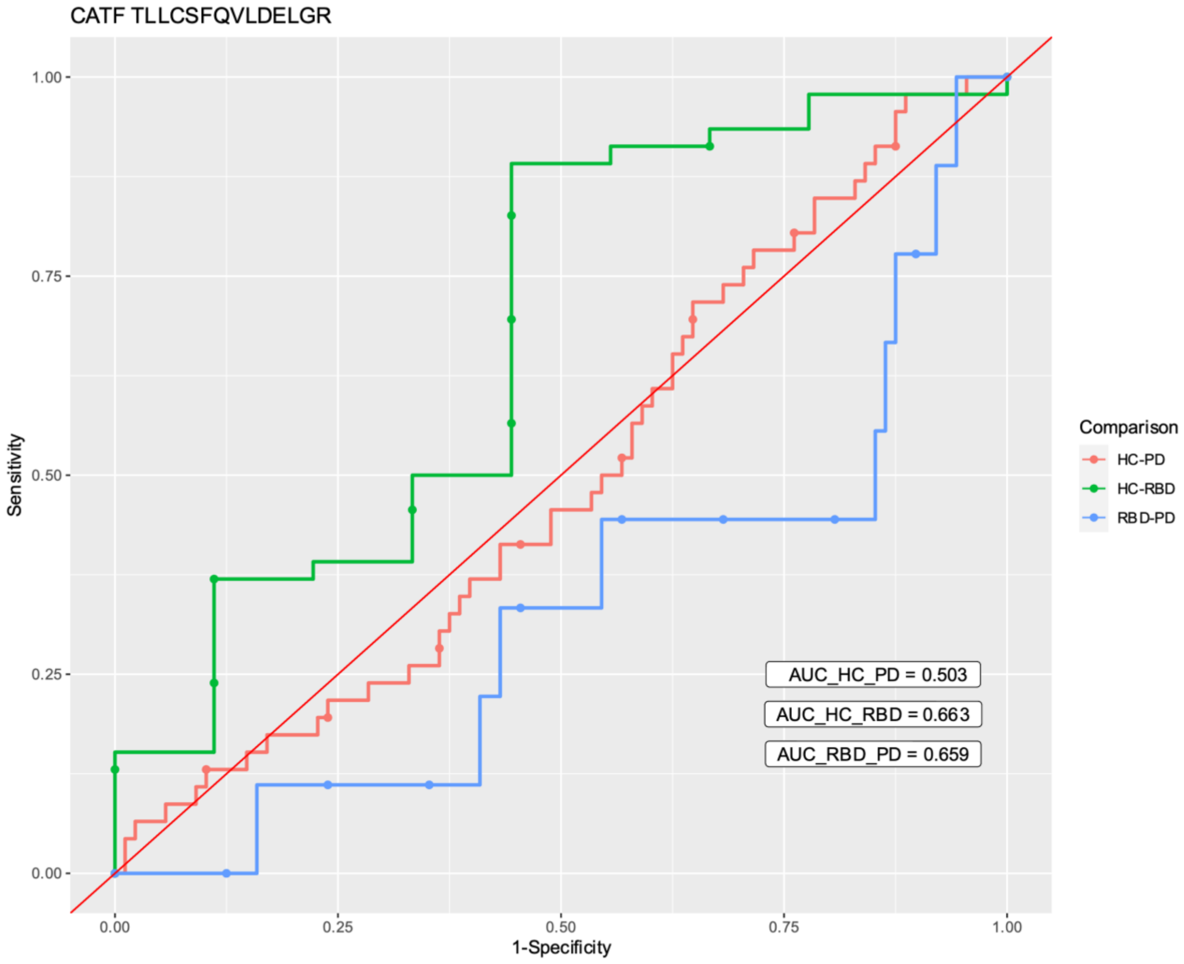


***Supplementary Figure 7u***: *Results of the discrimination analysis based on receiver operating characteristics (ROC)*

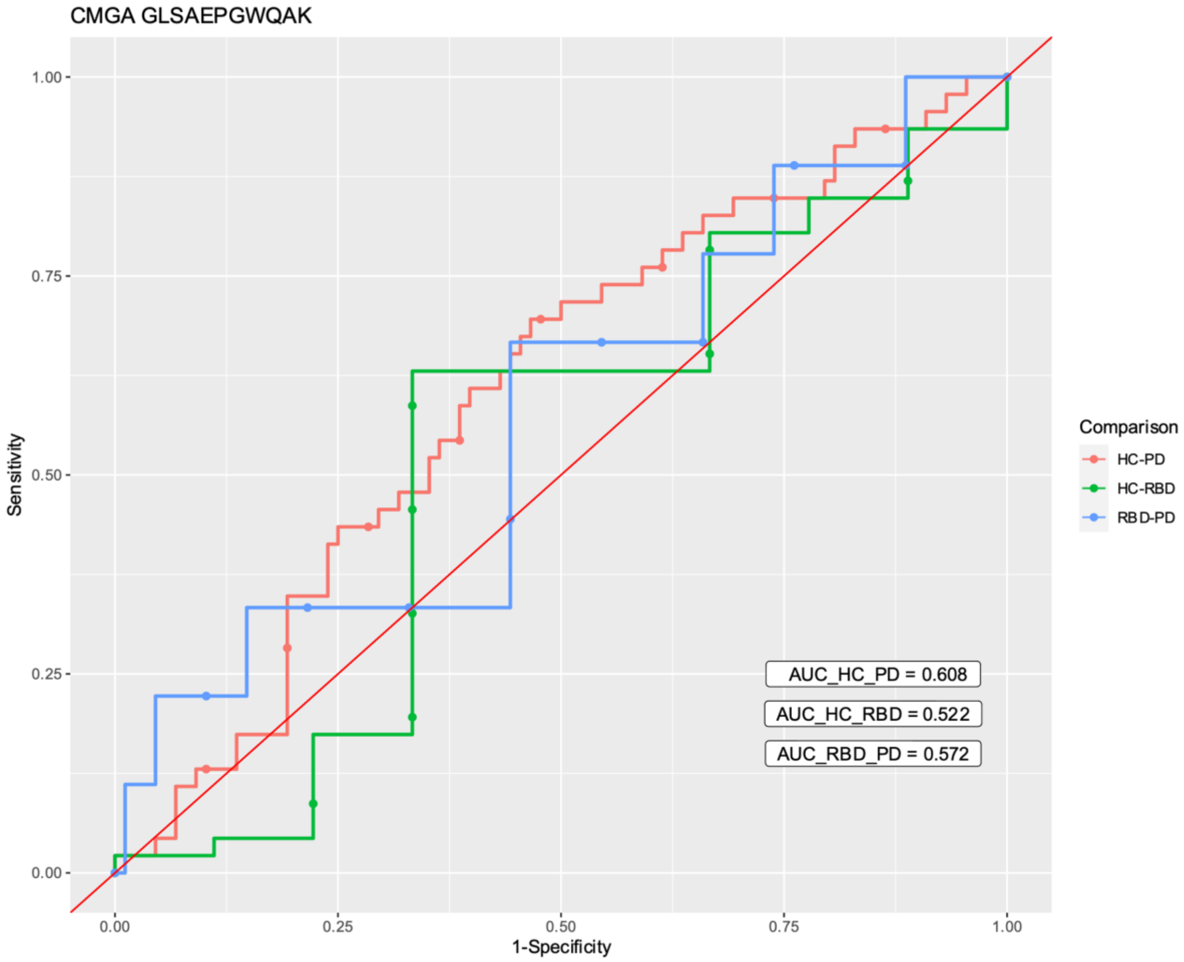


***Supplementary Figure 7v***: *Results of the discrimination analysis based on receiver operating characteristics (ROC)*

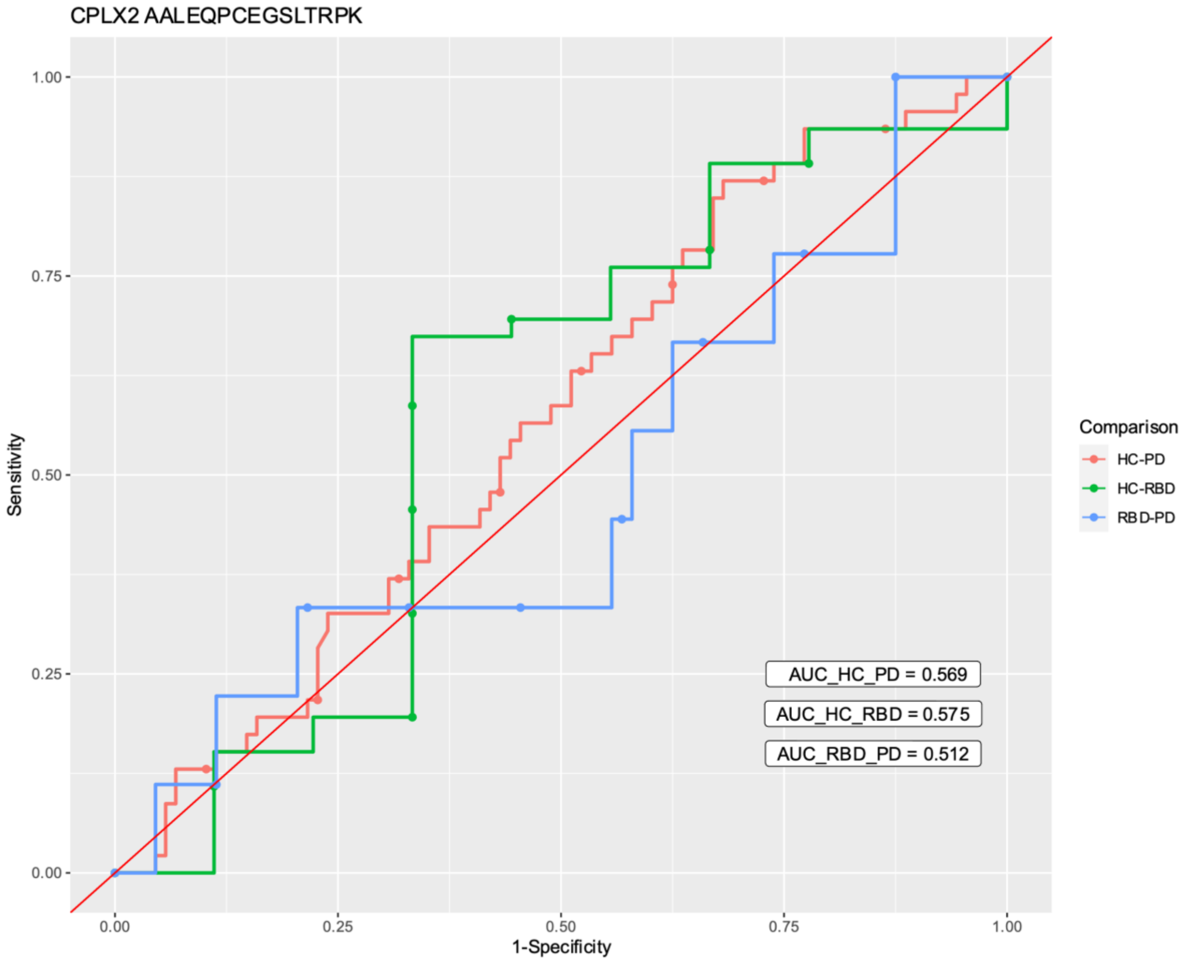


***Supplementary Figure 7w****: Results of the discrimination analysis based on receiver operating characteristics (ROC)*

## Supplementary Tables

***Supplementary Table 1.*** *Concentrations of internal standard for each peptide as well as repeatability and intermediate precision for the study after plate correction. Bold peptide indicate peptide selected per protein for statistical analyses.*

| TARGETS | IS CONC [FMOL/MICROL] | QC[1] Repeatability %CV[3] | QC[1] Intermediate precision %CV |
|---|---|---|---|
| **1433E IISSIEQK** | 0.26 | 21.40 | 19.71 |
| **1433F AVTELNEPLSNEDR** | 0.26 | 16.48 | 9.66 |
| **1433T AVTEQGAELSNEER** | 2.6 | 25.06 | 19.35 |
| **1433Z VVSSIEQK** | 0.26 | 7.36 | 4.71 |
| **A4 VESLEQEAANER** | 8.89 | 18.24 | 16.81 |
| **AP2B1 AVWLPAVK** | 0.26 | 4.36 | 2.36 |
| **AP2B1 IQPGNPNYTLSLK** | 0.82 | 16.43 | 14.36 |
| **AP2B1 QVFLATWK** | 3.53 | 15.61 | 12.81 |
| **CATF TLLCSFQVLDELGR** | 6.36 | 24.97 | 20.54 |
| **CMGA EDSLEAGLPLQVR** | 4.53 | 9.27 | 7.28 |
| **CMGA GLSAEPGWQAK** | 5.9 | 7.09 | 3.41 |
| **CPLX2 AALEQPCEGSLTRPK** | 0.26 | 9.69 | 5.36 |
| **GDIA QLICDPSYIPDR** | 2.6 | 12.38 | 7.42 |
| **LAMP2 IPLNDLFR** | 3.71 | 10.76 | 6.36 |
| **NEUG KGPGPGGPGGAGVAR** | 0.26 | 8.99 | 2.52 |
| **NPTX1 CESQSTLDPGAGEAR** | 2.6 | 9.24 | 5.45 |
| **NPTX1 ETVLQQK** | 2.6 | 8.05 | 5.36 |
| **NPTX2 ETVVQQK** | 2.6 | 15.64 | 6.46 |
| **NPTX2 VAELEDEK** | 0.26 | 6.90 | 4.70 |
| **NPTXR LVEAFGGATK** | 2.6 | 26.47 | 25.08 |
| **NPTXR NNYMYAR** | 2.6 | 11.20 | 9.72 |
| **PEBP1 LYEQLSGK** | 0.26 | 6.32 | 1.95 |
| **PEBP1 NRPTSISWDGLDSGK** | 2.6 | 6.85 | 5.14 |
| **SAP3 EVAGLWIK** | 9.93 | 13.12 | 11.68 |
| **SAP3 IESVLSSSGK** | 6.28 | 7.25 | 5.33 |
| **SCG2 ALEYIENLR** | 3.45 | 8.19 | 5.72 |
| **SCG2 VLEYLNQEK** | 7.35 | 6.31 | 3.33 |
| **STX1B QHSAILAAPNPDEK** | 0.26 | 20.36 | 17.89 |
| **STX7 EFGSLPTTPSEQR** | 0.26 | 13.13 | 9.15 |
| **SYUB EGVVQGVASVAEK** | 0.26 | 16.15 | 11.92 |
| **SYUG ENVVQSVTSVAEK** | 0.26 | 8.98 | 5.40 |
| **VGF AYQGVAAPFPK** | 3.71 | 6.30 | 3.26 |
| **VGF NSEPQDEGELFQGVDPR** | 17.1 | 5.56 | 2.04 |

***Supplementary Table 2**. LC-MS/MS settings for the analysis of the synaptic and lysosomal protein panel.*

| | Parameter | Setting |
|---|---|---|
| **LC** | Sample injection volume | 40 µL |
| | Flow-rate | 0.3 mL/min |
| | Gradient | Broken; 5–20%B (25 min), 20–45%B (6,5 min) |
| | Total cycle time | 35 min |
| | Mobile phase A | 0.1% formic acid in water (v/v) |
| | Mobile phase B | 0.1% formic acid/84% acetonitrile in water (v/v) |
| **Electrospray** | Mode | Positive |
| | Gas temperature | 220 °C |
| | Gas flow | 15 L/min |
| | Nebulizer pressure | 40 psi |
| | Sheath gas temperature | 200 °C |
| | Sheath gas flow | 11 L/min |
| | Capillary voltage | 3500 V |
| | Nozzle voltage | 500 V |
| **iFunnel** | Mode | Positive |
| | High-pressure radio frequency | 200 V |
| | Low-pressure radio frequency | 160 V |
| **MRM method** | Retention time window | 1 min |
| | Collision energies | Individually optimized per transition |
| | Cell accelerator voltage | Individually optimized per transition |

***Supplementary Table 3:*** *Results of the linear mixed models for the applied protein panel, adjusted for age, sex, time and levodopa equivalent dosage (LEDD)*

| marker | term | sumsq | meansq | NumDF | DenDF | statistic | p.value | pval_temp | pval_adj | Thresh |
|---|---|---|---|---|---|---|---|---|---|---|
| 1433E IISSIEQK | AGE | 2.90 | 2.90 | 1.00 | 169.03 | 5.73 | 0.02 | | | sig |
| 1433E IISSIEQK | SEX | 2.11 | 2.11 | 1,00 | 157,78 | 4.16 | 0.04 | | | sig |
| 1433E IISSIEQK | COHORT | 0.28 | 0.28 | 1.00 | 293.16 | 0.55 | 0.46 | 0.46 | 0.78 | no-sig |
| 1433E IISSIEQK | TIME | 0.01 | 0.01 | 1.00 | 345.21 | 0.02 | 0.89 | 0.89 | 0.91 | no-sig |
| 1433E IISSIEQK | log10LEDD | 0.00 | 0.00 | 1.00 | 303.82 | 0.00 | 0.98 | 0,98 | 0.98 | no-sig |
| 1433E IISSIEQK | COHORT:TIME | 0.33 | 0.33 | 1.00 | 314.99 | 0.65 | 0.42 | 0.42 | 0.78 | no-sig |
| 1433F AVTELNEPLSNEDR | AGE | 0.42 | 0.42 | 1.00 | 145.88 | 1.92 | 0.17 | | | |
| 1433F AVTELNEPLSNEDR | SEX | 1.94 | 1.94 | 1.00 | 140.25 | 8.89 | 0.00 | | | |
| 1433F AVTELNEPLSNEDR | COHORT | 0.24 | 0.24 | 1.00 | 200.33 | 1.10 | 0.30 | 0.30 | 0.75 | no-sig |
| 1433F AVTELNEPLSNEDR | TIME | 0.05 | 0.05 | 1.00 | 290.52 | 0.23 | 0.64 | 0.64 | 0.81 | no-sig |
| 1433F AVTELNEPLSNEDR | log10LEDD | 0.03 | 0.03 | 1.00 | 224.25 | 0.12 | 0.73 | 0.73 | 0.86 | no-sig |
| 1433F AVTELNEPLSNEDR | COHORT:TIME | 0.08 | 0.08 | 1.00 | 219.52 | 0.35 | 0.56 | 0.56 | 0.81 | no-sig |
| 1433T AVTEQGAELSNEER | AGE | 4.47 | 4.47 | 1.00 | 172.98 | 22.53 | 0.00 | | | |
| 1433T AVTEQGAELSNEER | SEX | 0.27 | 0.27 | 1.00 | 164.00 | 1.39 | 0.24 | | | |
| 1433T AVTEQGAELSNEER | COHORT | 0.02 | 0.02 | 1.00 | 275.29 | 0.09 | 0.76 | 0.76 | 0.86 | no-sig |
| 1433T AVTEQGAELSNEER | TIME | 0.01 | 0.01 | 1.00 | 339.88 | 0.07 | 0.79 | 0.79 | 0.86 | no-sig |
| 1433T AVTEQGAELSNEER | log10LEDD | 0.30 | 0.30 | 1.00 | 293.49 | 1.51 | 0.22 | 0.22 | 0.75 | no-sig |
| 1433T AVTEQGAELSNEER | COHORT:TIME | 0.19 | 0.19 | 1.00 | 298.83 | 0.96 | 0.33 | 0.33 | 0.75 | no-sig |
| 1433Z VVSSIEQK | AGE | 6.10 | 6.10 | 1.00 | 172.23 | 54.11 | 0.00 | | | |
| 1433Z VVSSIEQK | SEX | 0.07 | 0.07 | 1.00 | 165.02 | 0.59 | 0.44 | | | |
| 1433Z VVSSIEQK | COHORT | 0.09 | 0.09 | 1.00 | 256.43 | 0.82 | 0.37 | 0.37 | 0.76 | no-sig |
| 1433Z VVSSIEQK | TIME | 0.01 | 0.01 | 1.00 | 334.39 | 0.09 | 0.77 | 0.77 | 0.86 | no-sig |
| 1433Z VVSSIEQK | log10LEDD | 0.01 | 0.01 | 1.00 | 279.87 | 0.10 | 0.75 | 0.75 | 0.86 | no-sig |
| 1433Z VVSSIEQK | COHORT:TIME | 0.05 | 0.05 | 1.00 | 281.09 | 0.43 | 0.51 | 0.51 | 0.81 | no-sig |
| A4 VESLEQEAANER | AGE | 0.82 | 0.82 | 1.00 | 189.37 | 1.82 | 0.18 | | | |
| A4 VESLEQEAANER | SEX | 8.81 | 8.81 | 1.00 | 178.53 | 19.61 | 0.00 | | | |
| A4 VESLEQEAANER | COHORT | 2.05 | 2.05 | 1.00 | 301.05 | 4.57 | 0.03 | 0.03 | 0.34 | no-sig |
| A4 VESLEQEAANER | TIME | 0.55 | 0.55 | 1.00 | 349.02 | 1.22 | 0.27 | 0.27 | 0.75 | no-sig |
| A4 VESLEQEAANER | log10LEDD | 0.07 | 0.07 | 1.00 | 313.19 | 0.15 | 0.70 | 0.70 | 0.86 | no-sig |
| A4 VESLEQEAANER | COHORT:TIME | 0.29 | 0.29 | 1.00 | 321.45 | 0.64 | 0.42 | 0.42 | 0.78 | no-sig |
| AP2B1 AVWLPAVK | AGE | 0.79 | 0.79 | 1.00 | 162.33 | 12.18 | 0.00 | | | |
| AP2B1 AVWLPAVK | SEX | 0.89 | 0.89 | 1.00 | 158.06 | 13.71 | 0.00 | | | |
| AP2B1 AVWLPAVK | COHORT | 0.35 | 0.35 | 1.00 | 200.87 | 5.37 | 0.02 | 0.02 | 0.25 | no-sig |
| AP2B1 AVWLPAVK | TIME | 0.02 | 0.02 | 1.00 | 340.05 | 0.28 | 0.59 | 0.59 | 0.81 | no-sig |
| AP2B1 AVWLPAVK | log10LEDD | 0.02 | 0.02 | 1.00 | 235.60 | 0.36 | 0.55 | 0.55 | 0.81 | no-sig |
| AP2B1 AVWLPAVK | COHORT:TIME | 0.04 | 0.04 | 1.00 | 231.52 | 0.57 | 0.45 | 0.45 | 0.78 | no-sig |
| CATF TLLCSFQVLDELGR | AGE | 0.20 | 0.20 | 1.00 | 181.00 | 1.10 | 0.30 | | | |
| CATF TLLCSFQVLDELGR | SEX | 0.57 | 0.57 | 1.00 | 170.47 | 3.12 | 0.08 | | | |
| CATF TLLCSFQVLDELGR | COHORT | 0.02 | 0.02 | 1.00 | 296.21 | 0.13 | 0.72 | 0.72 | 0.86 | no-sig |
| CATF TLLCSFQVLDELGR | TIME | 0.05 | 0.05 | 1.00 | 343.47 | 0.26 | 0.61 | 0.61 | 0.81 | no-sig |
| CATF TLLCSFQVLDELGR | log10LEDD | 0.01 | 0.01 | 1.00 | 309.62 | 0.08 | 0.78 | 0.78 | 0.86 | no-sig |
| CATF TLLCSFQVLDELGR | COHORT:TIME | 0.15 | 0.15 | 1.00 | 318.30 | 0.80 | 0.37 | 0.37 | 0.76 | no-sig |
| CMGA GLSAEPGWQAK | AGE | 0.81 | 0.81 | 1.00 | 162.67 | 8.08 | 0.01 | | | |

| marker | term | sumsq | meansq | NumDF | DenDF | statistic | p.value | pval_temp | pval_adj | Thresh |
|---|---|---|---|---|---|---|---|---|---|---|
| CMGA GLSAEPGWQAK | SEX | 1.82 | 1.82 | 1.00 | 158.44 | 18.19 | 0.00 | | | |
| CMGA GLSAEPGWQAK | COHORT | 0.54 | 0.54 | 1.00 | 189.22 | 5.39 | 0.02 | 0.02 | 0.25 | no-sig |
| CMGA GLSAEPGWQAK | TIME | 0.07 | 0.07 | 1.00 | 352.99 | 0.72 | 0.40 | 0.40 | 0.78 | no-sig |
| CMGA GLSAEPGWQAK | log10LEDD | 0.02 | 0.02 | 1.00 | 226.10 | 0.21 | 0.65 | 0.65 | 0.81 | no-sig |
| CMGA GLSAEPGWQAK | COHORT:TIME | 0.10 | 0.10 | 1.00 | 221.82 | 0.98 | 0.32 | 0.32 | 0.75 | no-sig |
| CPLX2 AALEQPCEGSLTRPK | AGE | 3.75 | 3.75 | 1.00 | 182.22 | 11.70 | 0.00 | | | |
| CPLX2 AALEQPCEGSLTRPK | SEX | 2.10 | 2.10 | 1.00 | 170.88 | 6.53 | 0.01 | | | |
| CPLX2 AALEQPCEGSLTRPK | COHORT | 0.45 | 0.45 | 1.00 | 300.38 | 1.41 | 0.24 | 0.24 | 0.75 | no-sig |
| CPLX2 AALEQPCEGSLTRPK | TIME | 0.18 | 0.18 | 1.00 | 349.26 | 0.55 | 0.46 | 0.46 | 0.78 | no-sig |
| CPLX2 AALEQPCEGSLTRPK | log10LEDD | 0.08 | 0.08 | 1.00 | 312.14 | 0.25 | 0.62 | 0.62 | 0.81 | no-sig |
| CPLX2 AALEQPCEGSLTRPK | COHORT:TIME | 0.07 | 0.07 | 1.00 | 321.80 | 0.21 | 0.64 | 0.64 | 0.81 | no-sig |
| GDIA QLICDPSYIPDR | AGE | 4.31 | 4.31 | 1.00 | 197.78 | 12.98 | 0.00 | | | |
| GDIA QLICDPSYIPDR | SEX | 0.93 | 0.93 | 1.00 | 180.15 | 2.79 | 0.10 | | | |
| GDIA QLICDPSYIPDR | COHORT | 0.36 | 0.36 | 1.00 | 332.98 | 1.07 | 0.30 | 0.30 | 0.75 | no-sig |
| GDIA QLICDPSYIPDR | TIME | 0.51 | 0.51 | 1.00 | 356.73 | 1.53 | 0.22 | 0.22 | 0.75 | no-sig |
| GDIA QLICDPSYIPDR | log10LEDD | 0.19 | 0.19 | 1.00 | 337.22 | 0.58 | 0.45 | 0.45 | 0.78 | no-sig |
| GDIA QLICDPSYIPDR | COHORT:TIME | 0.01 | 0.01 | 1.00 | 348.24 | 0.03 | 0.86 | 0.86 | 0.89 | no-sig |
| LAMP2 IPLNDLFR | AGE | 0.69 | 0.69 | 1.00 | 151.58 | 4.50 | 0.04 | | | |
| LAMP2 IPLNDLFR | SEX | 1.27 | 1.27 | 1.00 | 145.56 | 8.31 | 0.00 | | | |
| LAMP2 IPLNDLFR | COHORT | 0.29 | 0.29 | 1.00 | 214.20 | 1.89 | 0.17 | 0.17 | 0.75 | no-sig |
| LAMP2 IPLNDLFR | TIME | 0.20 | 0.20 | 1.00 | 293.60 | 1.32 | 0.25 | 0.25 | 0.75 | no-sig |
| LAMP2 IPLNDLFR | log10LEDD | 0.10 | 0.10 | 1.00 | 235.19 | 0.64 | 0.43 | 0.43 | 0.78 | no-sig |
| LAMP2 IPLNDLFR | COHORT:TIME | 0.28 | 0.28 | 1.00 | 233.80 | 1.84 | 0.18 | 0.18 | 0.75 | no-sig |
| NEUG KGPGPGGPGGAGVAR | AGE | 0.62 | 0.62 | 1.00 | 164.46 | 11.62 | 0.00 | | | |
| NEUG KGPGPGGPGGAGVAR | SEX | 1.43 | 1.43 | 1.00 | 159.96 | 26.93 | 0.00 | | | |
| NEUG KGPGPGGPGGAGVAR | COHORT | 0.39 | 0.39 | 1.00 | 182.21 | 7.24 | 0.01 | 0.01 | 0.12 | no-sig |
| NEUG KGPGPGGPGGAGVAR | TIME | 0.12 | 0.12 | 1.00 | 363.86 | 2.16 | 0.14 | 0.14 | 0.75 | no-sig |
| NEUG KGPGPGGPGGAGVAR | log10LEDD | 0.01 | 0.01 | 1.00 | 220.70 | 0.09 | 0.76 | 0.76 | 0.86 | no-sig |
| NEUG KGPGPGGPGGAGVAR | COHORT:TIME | 0.13 | 0.13 | 1.00 | 217.57 | 2.37 | 0.12 | 0.12 | 0.75 | no-sig |
| NPTX1 ETVLQQK | AGE | 0.23 | 0.23 | 1.00 | 162.47 | 2.94 | 0.09 | | | |
| NPTX1 ETVLQQK | SEX | 1.26 | 1.26 | 1.00 | 158.28 | 16.00 | 0.00 | | | |
| NPTX1 ETVLQQK | COHORT | 0.59 | 0.59 | 1.00 | 198.99 | 7.48 | 0.01 | 0.01 | 0.12 | no-sig |
| NPTX1 ETVLQQK | TIME | 0.02 | 0.02 | 1.00 | 343.40 | 0.25 | 0.61 | 0.61 | 0.81 | no-sig |
| NPTX1 ETVLQQK | log10LEDD | 0.01 | 0.01 | 1.00 | 234.69 | 0.15 | 0.70 | 0.70 | 0.86 | no-sig |
| NPTX1 ETVLQQK | COHORT:TIME | 0.02 | 0.02 | 1.00 | 230.68 | 0.22 | 0.64 | 0.64 | 0.81 | no-sig |
| NPTX2 VAELEDEK | AGE | 0.03 | 0.03 | 1.00 | 170.72 | 0.14 | 0.71 | | | |
| NPTX2 VAELEDEK | SEX | 3.20 | 3.20 | 1.00 | 164.84 | 15.44 | 0.00 | | | |
| NPTX2 VAELEDEK | COHORT | 1.99 | 1.99 | 1.00 | 239.52 | 9.60 | 0.00 | 0.00 | 0.11 | no-sig |
| NPTX2 VAELEDEK | TIME | 0.20 | 0.20 | 1.00 | 331.00 | 0.98 | 0.32 | 0.32 | 0.75 | no-sig |
| NPTX2 VAELEDEK | log10LEDD | 0.02 | 0.02 | 1.00 | 266.17 | 0.09 | 0.77 | 0.77 | 0.86 | no-sig |
| NPTX2 VAELEDEK | COHORT:TIME | 0.41 | 0.41 | 1.00 | 264.73 | 2.00 | 0.16 | 0.16 | 0.75 | no-sig |
| NPTXR NNYMYAR | AGE | 0.41 | 0.41 | 1.00 | 165.46 | 2.90 | 0.09 | | | |
| NPTXR NNYMYAR | SEX | 2.68 | 2.68 | 1,00 | 160.98 | 19.10 | 0.00 | | | |
| NPTXR NNYMYAR | COHORT | 1.21 | 1.21 | 1.00 | 210.25 | 8.63 | 0.00 | 0.00 | 0.11 | no-sig |
| NPTXR NNYMYAR | TIME | 0.17 | 0.17 | 1.00 | 337.52 | 1.24 | 0.27 | 0.27 | 0.75 | no-sig |

| marker | term | sumsq | meansq | NumDF | DenDF | statistic | p.value | pval_temp | pval_adj | Thresh |
|---|---|---|---|---|---|---|---|---|---|---|
| NPTXR NNYMYAR | log10LEDD | 0.05 | 0.05 | 1.00 | 244.13 | 0.36 | 0.55 | 0.55 | 0.81 | no-sig |
| NPTXR NNYMYAR | COHORT:TIME | 0.04 | 0.04 | 1.00 | 240.30 | 0.25 | 0.62 | 0.62 | 0.81 | no-sig |
| PEBP1 LYEQLSGK | AGE | 3.51 | 3.51 | 1.00 | 170.34 | 34.60 | 0.00 | | | |
| PEBP1 LYEQLSGK | SEX | 0.47 | 0.47 | 1.00 | 163.02 | 4.68 | 0.03 | | | |
| PEBP1 LYEQLSGK | COHORT | 0.03 | 0.03 | 1.00 | 256.19 | 0.33 | 0.57 | 0.57 | 0.81 | no-sig |
| PEBP1 LYEQLSGK | TIME | 0.14 | 0.14 | 1.00 | 334.10 | 1.42 | 0.23 | 0.23 | 0.75 | no-sig |
| PEBP1 LYEQLSGK | log10LEDD | 0.04 | 0.04 | 1.00 | 279.50 | 0.37 | 0.55 | 0.55 | 0.81 | no-sig |
| PEBP1 LYEQLSGK | COHORT:TIME | 0.01 | 0.01 | 1.00 | 281.01 | 0.06 | 0.81 | 0.81 | 0.88 | no-sig |
| SAP3 IESVLSSSGK | AGE | 1.54 | 1.54 | 1.00 | 175.21 | 11.19 | 0.00 | | | |
| SAP3 IESVLSSSGK | SEX | 0.40 | 0.40 | 1.00 | 167.92 | 2.94 | 0.09 | | | |
| SAP3 IESVLSSSGK | COHORT | 0.04 | 0.04 | 1.00 | 255.19 | 0.31 | 0.58 | 0.58 | 0.81 | no-sig |
| SAP3 IESVLSSSGK | TIME | 0.14 | 0.14 | 1.00 | 333.36 | 1.03 | 0.31 | 0.31 | 0.75 | no-sig |
| SAP3 IESVLSSSGK | log10LEDD | 0.07 | 0.07 | 1.00 | 279.47 | 0.48 | 0.49 | 0.49 | 0,81 | no-sig |
| SAP3 IESVLSSSGK | COHORT:TIME | 0.17 | 0.17 | 1.00 | 278.97 | 1.21 | 0.27 | 0.27 | 0.75 | no-sig |
| SCG2 VLEYLNQEK | AGE | 0.51 | 0.51 | 1.00 | 160.45 | 3.58 | 0.06 | | | |
| SCG2 VLEYLNQEK | SEX | 1.81 | 1.81 | 1.00 | 154.98 | 12.81 | 0.00 | | | |
| SCG2 VLEYLNQEK | COHORT | 1.04 | 1.04 | 1.00 | 224.03 | 7.34 | 0.01 | 0.01 | 0.12 | no-sig |
| SCG2 VLEYLNQEK | TIME | 0.42 | 0.42 | 1.00 | 329.06 | 3.00 | 0.08 | 0.08 | 0.70 | no-sig |
| SCG2 VLEYLNQEK | log10LEDD | 0.13 | 0.13 | 1.00 | 254.37 | 0.94 | 0.33 | 0.33 | 0.75 | no-sig |
| SCG2 VLEYLNQEK | COHORT:TIME | 0.19 | 0.19 | 1.00 | 251.98 | 1.34 | 0.25 | 0.25 | 0.75 | no-sig |
| STX1B QHSAILAAPNPDEK | AGE | 2.55 | 2.55 | 1.00 | 172.38 | 13.75 | 0.00 | | | |
| STX1B QHSAILAAPNPDEK | SEX | 1.60 | 1.60 | 1.00 | 164.67 | 8.63 | 0.00 | | | |
| STX1B QHSAILAAPNPDEK | COHORT | 0.52 | 0.52 | 1.00 | 257.63 | 2.79 | 0.10 | 0.10 | 0.73 | no-sig |
| STX1B QHSAILAAPNPDEK | TIME | 0.07 | 0.07 | 1.00 | 332.54 | 0.40 | 0.53 | 0.53 | 0.81 | no-sig |
| STX1B QHSAILAAPNPDEK | log10LEDD | 0.01 | 0.01 | 1.00 | 278.74 | 0.04 | 0.85 | 0.85 | 0.89 | no-sig |
| STX1B QHSAILAAPNPDEK | COHORT:TIME | 0.13 | 0.13 | 1.00 | 280.27 | 0.70 | 0.40 | 0.40 | 0.78 | no-sig |
| STX7 EFGSLPTTPSEQR | AGE | 5.11 | 5.11 | 1.00 | 189.25 | 18.29 | 0.00 | | | |
| STX7 EFGSLPTTPSEQR | SEX | 1.21 | 1.21 | 1.00 | 177.34 | 4.32 | 0.04 | | | |
| STX7 EFGSLPTTPSEQR | COHORT | 0.43 | 0.43 | 1.00 | 306.28 | 1.53 | 0.22 | 0.22 | 0.75 | no-sig |
| STX7 EFGSLPTTPSEQR | TIME | 0.01 | 0.01 | 1.00 | 350.63 | 0.03 | 0.86 | 0.86 | 0.89 | no-sig |
| STX7 EFGSLPTTPSEQR | log10LEDD | 0.00 | 0.00 | 1.00 | 317.16 | 0.01 | 0.92 | 0.92 | 0.93 | no-sig |
| STX7 EFGSLPTTPSEQR | COHORT:TIME | 0.23 | 0.23 | 1.00 | 326.60 | 0.84 | 0.36 | 0.36 | 0.76 | no-sig |
| SYUB EGVVQGVASVAEK | AGE | 2.46 | 2.46 | 1.00 | 167.67 | 16.54 | 0.00 | | | |
| SYUB EGVVQGVASVAEK | SEX | 1.65 | 1.65 | 1.00 | 161.48 | 11.10 | 0.00 | | | |
| SYUB EGVVQGVASVAEK | COHORT | 0.21 | 0.21 | 1.00 | 239.30 | 1.43 | 0.23 | 0.23 | 0.75 | no-sig |
| SYUB EGVVQGVASVAEK | TIME | 0.60 | 0.60 | 1.00 | 328.93 | 4.01 | 0.05 | 0.05 | 0.42 | no-sig |
| SYUB EGVVQGVASVAEK | log10LEDD | 0.15 | 0.15 | 1.00 | 265.44 | 0.99 | 0.32 | 0.32 | 0.75 | no-sig |
| SYUB EGVVQGVASVAEK | COHORT:TIME | 0.22 | 0.22 | 1.00 | 264.22 | 1.51 | 0.22 | 0.22 | 0.75 | no-sig |
| SYUG ENVVQSVTSVAEK | AGE | 4.42 | 4.42 | 1.00 | 172.02 | 23.01 | 0.00 | | | |
| SYUG ENVVQSVTSVAEK | SEX | 0.59 | 0.59 | 1.00 | 163.06 | 3.09 | 0.08 | | | |
| SYUG ENVVQSVTSVAEK | COHORT | 0.01 | 0.01 | 1.00 | 272.88 | 0.04 | 0.85 | 0.85 | 0.89 | no-sig |
| SYUG ENVVQSVTSVAEK | TIME | 0.17 | 0.17 | 1.00 | 338.37 | 0.91 | 0.34 | 0.34 | 0.75 | no-sig |
| SYUG ENVVQSVTSVAEK | log10LEDD | 0.09 | 0.09 | 1.00 | 291.79 | 0.46 | 0.50 | 0.50 | 0.81 | no-sig |
| SYUG ENVVQSVTSVAEK | COHORT:TIME | 0.41 | 0.41 | 1.00 | 296.59 | 2.15 | 0.14 | 0.14 | 0.75 | no-sig |
| VGF NSEPQDEGELFQGVDPR | AGE | 0.08 | 0.08 | 1.00 | 159.26 | 1.56 | 0.21 | | | |

| marker | term | sumsq | meansq | NumDF | DenDF | statistic | p.value | pval_temp | pval_adj | Thresh |
|---|---|---|---|---|---|---|---|---|---|---|
| VGF NSEPQDEGELFQGVDPR | SEX | 0.87 | 0.87 | 1.00 | 154.73 | 16.28 | 0.00 | | | |
| VGF NSEPQDEGELFQGVDPR | COHORT | 0.50 | 0.50 | 1.00 | 174.43 | 9.33 | 0.00 | 0.00 | 0.11 | no-sig |
| VGF NSEPQDEGELFQGVDPR | TIME | 0.13 | 0.13 | 1.00 | 353.74 | 2.37 | 0.12 | 0.12 | 0.75 | no-sig |
| VGF NSEPQDEGELFQGVDPR | log10LEDD | 0.07 | 0.07 | 1.00 | 210.19 | 1.36 | 0.25 | 0.25 | 0.75 | no-sig |
| VGF NSEPQDEGELFQGVDPR | COHORT:TIME | 0.07 | 0.07 | 1.00 | 207.59 | 1.34 | 0.25 | 0.25 | 0.75 | no-sig |

***Supplementary Table 4:*** *Data on the subjects in the longitudinal model. For some subjects, no baseline samples was available.  SAA data displays positive and negative results of the available samples Abbreviations: PD = Parkinson's Disease, HC = healthy control, iRBD = isolated REM sleep behavior disorder, n= number, sd = standard deviation, CSF= cerebrospinal fluid, MDS-UPDRS = Movement Disorders Society – United Parkinson's Disease Rating Scale, MMSE= Mini-Mental-State Examination, MoCa= Montreal Cognitive Assessment Score*

| | HC (N=58) | PD (N=104) | RBD (N=12) | Adj. p-values |
|---|---|---|---|---|
| **Sex** | | | | 0.176 |
| **- female** | 18 (31.0%) | 34 (32.7%) | 7 (58.3%) | |
| **- male** | 40 (69.0%) | 70 (67.3%) | 5 (41.7%) | |
| **Age** | | | | 0.692 |
| **- Mean (SD)** | 67.5 (7.23) | 67.12 (9.74) | 65.3 (8.52) | |
| **BMI** | | | | 0.072 |
| **- Mean (SD)** | 26.97 (4.45) | 28.0 (4.61) | 25.6 (3.68) | |
| **MDS-UPDRS-III** | | | | < 0.001** |
| **- Mean (SD)** | 1.07 (1.89) | 25.99 (12.79) | 2.000 (2.56) | |
| **MDS-UPDRS total score** | | | | < 0.001** |
| **- Mean (SD)** | 6.07 (6.69) | 45.98 (21.05) | 18.75 (7.74) | |
| **Hoehn & Yahr** | | | | < 0.001** |
| **- Mean (SD)** | 0.02 (0.13) | 2.098 (0.74) | 0.08 (0.29) | |
| **MMSE total score** | | | | 0.007 |
| **- Mean (SD)** | 28.67 (1.37) | 28.00 (1.65) | 28.833 (1.27) | |
| **MoCA total score** | | | | 0.02* |
| **- Mean (SD)** | 25.76 (2.54) | 24.53 (3.32) | 27.42 (2.07) | |
| **CSF albumin quotient** | | | | 0.080 |
| **- Mean (SD)** | 7.39 (3.18) | 8.42 (3.84) | 6.58 (1.64) | |
| **CSF β-amyloid (Aβ)** | | | | 0.154 |
| **- Mean (SD)** | 851.626 (197.58) | 883.72 (233.42) | 673.667 (65.68) | |
| **CSF phospho-tau protein (pTau)** | | | | 0.066 |
| **- Mean (SD)** | 44.966 (16.72) | 42.192 (16.33) | 27.767 (3.36) | |
| **CSF Total tau protein (tTau)** | | | | 0.099 |
| **- Mean (SD)** | 256.842 (122.39) | 235.63 (115.46) | 159.000 (16.82) | |
| **CSF neurofilament light chains (NfL)** | | | | < 0.001** |
| **- Mean (SD)** | 582.15 (252.59) | 799.90 (626.59) | 368.70 (88.29) | < 0.001** |
| **α-synuclein seeding aggregation assay (SAA)** | 0/50 (+/-) out of 50 CSF samples | 87/13 (+/-) out of 100 CSF samples | 11/1 (+/-) out of 12 CSF samples | N/A |

***Supplementary Table 5:*** *Neuronal α-synuclein-disease integrated staging system (NSD-ISS) based on the available clinical, α-synuclein seeding aggregation assay (SAA) and DAT-SPECT data. Applying the NSD-ISS categorized four subjects that in stage NSD2A (iRBD, S+ D-), six subjects in stage NSD2B (iRBD, S+ D+) and 78 subjects fulfilling the criteria for stage 4 (PD, S+ D+, moderate clinical impairment).*

| Pat. ID | Stage | aSyn Biomarker (S) | Dopamine Dysfunction Biomarker (D) | Clinical Signs and Symptoms | Functional Impariment |
|---|---|---|---|---|---|
| **ISS-PD1** | 4 | S+ | D+ | Relevant motor/non-motor signs | Mild functional impairment |
| **ISS-PD2** | 4 | S- | D+ | Relevant motor/non-motor signs | Mild functional impairment |
| **ISS-PD3** | 4 | S+ | D+ | Relevant motor/non-motor signs | Mild functional impairment |
| **ISS-PD4** | 4 | S+ | D+ | Relevant motor/non-motor signs | Mild functional impairment |
| **ISS-PD5** | 4 | S+ | D+ | Relevant motor/non-motor signs | Mild functional impairment |
| **ISS-PD6** | 4 | S+ | D+ | Relevant motor/non-motor signs | Mild functional impairment |
| **ISS-PD7** | 4 | S- | D+ | Relevant motor/non-motor signs | Mild functional impairment |
| **ISS-PD8** | 4 | S+ | D+ | Relevant motor/non-motor signs | Mild functional impairment |
| **ISS-PD9** | 4 | S+ | D+ | Relevant motor/non-motor signs | Mild functional impairment |
| **ISS-PD10** | 4 | S+ | D+ | Relevant motor/non-motor signs | Mild functional impairment |
| **ISS-PD11** | 4 | S+ | D+ | Relevant motor/non-motor signs | Mild functional impairment |
| **ISS-PD12** | 4 | S- | D+ | Relevant motor/non-motor signs | Mild functional impairment |
| **ISS-PD13** | 4 | S+ | D+ | Relevant motor/non-motor signs | Mild functional impairment |
| **ISS-PD14** | 4 | S+ | D+ | Relevant motor/non-motor signs | Mild functional impairment |
| **ISS-PD15** | 4 | S+ | D+ | Relevant motor/non-motor signs | Mild functional impairment |
| **ISS-PD16** | 4 | S+ | D+ | Relevant motor/non-motor signs | Mild functional impairment |
| **ISS-PD17** | 4 | S- | D+ | Relevant motor/non-motor signs | Mild functional impairment |
| **ISS-PD18** | 4 | S+ | D+ | Relevant motor/non-motor signs | Mild functional impairment |
| **ISS-PD19** | 4 | S+ | D+ | Relevant motor/non-motor signs | Mild functional impairment |
| **ISS-PD20** | 4 | S- | D+ | Relevant motor/non-motor signs | Mild functional impairment |
| **ISS-PD21** | 4 | S+ | D+ | Relevant motor/non-motor signs | Mild functional impairment |
| **ISS-PD22** | 4 | S- | D+ | Relevant motor/non-motor signs | Mild functional impairment |
| **ISS-PD23** | 4 | S+ | D+ | Relevant motor/non-motor signs | Mild functional impairment |
| **ISS-PD24** | 4 | S+ | D+ | Relevant motor/non-motor signs | Mild functional impairment |
| **ISS-PD25** | 4 | S+ | D+ | Relevant motor/non-motor signs | Mild functional impairment |
| **ISS-PD26** | 4 | S+ | D+ | Relevant motor/non-motor signs | Mild functional impairment |
| **ISS-PD27** | 4 | S+ | D+ | Relevant motor/non-motor signs | Mild functional impairment |
| **ISS-PD28** | 4 | S+ | D+ | Relevant motor/non-motor signs | Mild functional impairment |
| **ISS-PD29** | 4 | S+ | D+ | Relevant motor/non-motor signs | Mild functional impairment |
| **ISS-PD30** | 4 | S+ | D+ | Relevant motor/non-motor signs | Mild functional impairment |
| **ISS-PD31** | 4 | S+ | D+ | Relevant motor/non-motor signs | Mild functional impairment |
| **ISS-PD32** | 4 | S+ | D+ | Relevant motor/non-motor signs | Mild functional impairment |
| **ISS-PD33** | 4 | S+ | D+ | Relevant motor/non-motor signs | Mild functional impairment |
| **ISS-PD34** | 4 | S+ | D+ | Relevant motor/non-motor signs | Mild functional impairment |
| **ISS-PD35** | 4 | S+ | D+ | Relevant motor/non-motor signs | Mild functional impairment |
| **ISS-PD36** | 4 | S+ | D+ | Relevant motor/non-motor signs | Mild functional impairment |
| **ISS-PD37** | 4 | S+ | D+ | Relevant motor/non-motor signs | Mild functional impairment |
| **ISS-PD38** | 4 | S+ | D+ | Relevant motor/non-motor signs | Mild functional impairment |
| **ISS-PD39** | 4 | S+ | D+ | Relevant motor/non-motor signs | Mild functional impairment |
| **ISS-PD40** | 4 | S+ | D+ | Relevant motor/non-motor signs | Mild functional impairment |
| **ISS-PD41** | 4 | S+ | D+ | Relevant motor/non-motor signs | Mild functional impairment |

| Pat. ID | Stage | aSyn Biomarker (S) | Dopamine Dysfunction Biomarker (D) | Clinical Signs and Symptoms | Functional Impariment |
|---|---|---|---|---|---|
| **ISS-PD42** | 4 | S+ | D+ | Relevant motor/non-motor signs | Mild functional impairment |
| **ISS-PD43** | 4 | S+ | D+ | Relevant motor/non-motor signs | Mild functional impairment |
| **ISS-PD44** | 4 | S- | D+ | Relevant motor/non-motor signs | Mild functional impairment |
| **ISS-PD45** | 4 | S+ | D+ | Relevant motor/non-motor signs | Mild functional impairment |
| **ISS-PD46** | 4 | S+ | D+ | Relevant motor/non-motor signs | Mild functional impairment |
| **ISS-PD47** | 4 | S+ | D+ | Relevant motor/non-motor signs | Mild functional impairment |
| **ISS-PD48** | 4 | S+ | D+ | Relevant motor/non-motor signs | Mild functional impairment |
| **ISS-PD49** | 4 | S- | D+ | Relevant motor/non-motor signs | Mild functional impairment |
| **ISS-PD50** | 4 | S+ | D+ | Relevant motor/non-motor signs | Mild functional impairment |
| **ISS-PD51** | 4 | S+ | D+ | Relevant motor/non-motor signs | Mild functional impairment |
| **ISS-PD52** | 4 | S+ | D+ | Relevant motor/non-motor signs | Mild functional impairment |
| **ISS-PD53** | 4 | S+ | D+ | Relevant motor/non-motor signs | Mild functional impairment |
| **ISS-PD54** | 4 | S+ | D+ | Relevant motor/non-motor signs | Mild functional impairment |
| **ISS-PD55** | 4 | S+ | D+ | Relevant motor/non-motor signs | Mild functional impairment |
| **ISS-PD56** | 4 | S+ | D+ | Relevant motor/non-motor signs | Mild functional impairment |
| **ISS-PD57** | 4 | S- | D+ | Relevant motor/non-motor signs | Mild functional impairment |
| **ISS-PD58** | 4 | S+ | D+ | Relevant motor/non-motor signs | Mild functional impairment |
| **ISS-PD59** | 4 | S+ | D+ | Relevant motor/non-motor signs | Mild functional impairment |
| **ISS-PD60** | 4 | S+ | D+ | Relevant motor/non-motor signs | Mild functional impairment |
| **ISS-PD61** | 4 | S+ | D+ | Relevant motor/non-motor signs | Mild functional impairment |
| **ISS-PD62** | 4 | S- | D+ | Relevant motor/non-motor signs | Mild functional impairment |
| **ISS-PD63** | 4 | S+ | D+ | Relevant motor/non-motor signs | Mild functional impairment |
| **ISS-PD64** | 4 | S- | D+ | Relevant motor/non-motor signs | Mild functional impairment |
| **ISS-PD65** | 4 | S- | D+ | Relevant motor/non-motor signs | Mild functional impairment |
| **ISS-PD66** | 4 | S+ | D+ | Relevant motor/non-motor signs | Mild functional impairment |
| **ISS-PD67** | 4 | S+ | D+ | Relevant motor/non-motor signs | Mild functional impairment |
| **ISS-PD68** | 4 | S+ | D+ | Relevant motor/non-motor signs | Mild functional impairment |
| **ISS-PD69** | 4 | S+ | D+ | Relevant motor/non-motor signs | Mild functional impairment |
| **ISS-PD70** | 4 | S+ | D+ | Relevant motor/non-motor signs | Mild functional impairment |
| **ISS-PD71** | 4 | S+ | D+ | Relevant motor/non-motor signs | Mild functional impairment |
| **ISS-PD72** | 4 | S+ | D+ | Relevant motor/non-motor signs | Mild functional impairment |
| **ISS-PD73** | 4 | S+ | D+ | Relevant motor/non-motor signs | Mild functional impairment |
| **ISS-PD74** | 4 | S+ | D+ | Relevant motor/non-motor signs | Mild functional impairment |
| **ISS-PD75** | 4 | S+ | D+ | Relevant motor/non-motor signs | Mild functional impairment |
| **ISS-PD76** | 4 | S+ | D+ | Relevant motor/non-motor signs | Mild functional impairment |
| **ISS-PD77** | 4 | S+ | D+ | Relevant motor/non-motor signs | Mild functional impairment |
| **ISS-PD78** | 4 | S+ | D+ | Relevant motor/non-motor signs | Mild functional impairment |
| **ISS-PD79** | 4 | S+ | D+ | Relevant motor/non-motor signs | Mild functional impairment |
| **ISS-PD80** | 4 | S- | D+ | Relevant motor/non-motor signs | Mild functional impairment |
| **ISS-PD81** | 4 | S+ | D+ | Relevant motor/non-motor signs | Mild functional impairment |
| **ISS-PD82** | 4 | S+ | D+ | Relevant motor/non-motor signs | Mild functional impairment |
| **ISS-PD83** | 4 | S+ | D+ | Relevant motor/non-motor signs | Mild functional impairment |
| **ISS-PD84** | 4 | S+ | D+ | Relevant motor/non-motor signs | Mild functional impairment |

| Pat. ID | Stage | aSyn Biomarker (S) | Dopamine Dysfunction Biomarker (D) | Clinical Signs and Symptoms | Functional Impariment |
|---|---|---|---|---|---|
| **ISS-PD85** | 4 | S+ | D+ | Relevant motor/non-motor signs | Mild functional impairment |
| **ISS-PD86** | 4 | S+ | D+ | Relevant motor/non-motor signs | Mild functional impairment |
| **ISS-PD87** | 4 | S+ | D+ | Relevant motor/non-motor signs | Mild functional impairment |
| **ISS-PD88** | 4 | S+ | D+ | Relevant motor/non-motor signs | Mild functional impairment |
| **ISS-PD89** | 4 | S+ | D+ | Relevant motor/non-motor signs | Mild functional impairment |
| **ISS-PD90** | 4 | S+ | D+ | Relevant motor/non-motor signs | Mild functional impairment |
| **ISS-PD91** | 4 | S+ | D+ | Relevant motor/non-motor signs | Mild functional impairment |
| **ISS-PD92** | 4 | S+ | D+ | Relevant motor/non-motor signs | Mild functional impairment |
| **ISS-iRBD1** | 2B | S+ | D+ | Subtle clinical signs: iRBD | No functional impairment |
| **ISS-iRBD2** | 2B | S+ | D+ | Subtle clinical signs: iRBD | No functional impairment |
| **ISS-iRBD3** | 2B | S+ | D+ | Subtle clinical signs: iRBD | No functional impairment |
| **ISS-iRBD4** | 2B | S+ | D+ | Subtle clinical signs: iRBD | No functional impairment |
| **ISS-iRBD5** | 2B | S+ | D+ | Subtle clinical signs: iRBD | No functional impairment |
| **ISS-iRBD6** | 2A | S+ | D- | Subtle clinical signs: iRBD | No functional impairment |
| **ISS-iRBD7** | 2A | S+ | D- | Subtle clinical signs: iRBD | No functional impairment |
| **ISS-iRBD8** | 2B | S+ | D+ | Subtle clinical signs: iRBD | No functional impairment |
| **ISS-iRBD9** | 2A | S+ | D- | Subtle clinical signs: iRBD | No functional impairment |
| **ISS-iRBD10** | 2A | S+ | D- | Subtle clinical signs: iRBD | No functional impairment |

***Supplementary Table 6****: Post hoc analysis using pairwise Fisher test for the chi-square test and the pairwise Dunn test for the Kruskall Wallis test; PD = Parkinson's Disease, HC = healthy control, iRBD = isolated REM sleep behavior disorder, n= number, sd = standard deviation, CSF: cerebrospinal fluid, MDS-UPDRS = Movement Disorders Society – United Parkinson's Disease Rating Scale, MMSE: Mini-Mental-State Examination, MoCa: Montreal Cognitive Assessment Score, BMI: Body-Mass-Index ns: non-significant*

| ***Chisq overall Test*** | | | | | | | | |
|---|---|---|---|---|---|---|---|---|
| **term** | **chisq.statistic** | **chisq.chisq-df** | **chisq.p-value** | | | | | **p-value-signif** |
| **Sex** | 8,65E+01 | 6,00E+00 | <0,0001 | | | | | **** |
| **α-synuclein seeding aggregation assay (SAA)** | 8,65E+01 | 6,00E+00 | <0,0001 | | | | | **** |
| ***Fisher pairwise Sex post hoc Test*** | | | | | | | | |
| **group1** | **group2** | **n** | **p** | **p.adj** | | | | **p-adj.signif** |
| **HC** | PD | 134 | 0,84 | 0,84 | | | | ns |
| **HC** | RBD | 55 | 0,01 | 0,02 | | | | * |
| **PD** | RBD | 97 | 0,01 | 0,02 | | | | * |
| ***Fisher pairwise SAA post hoc Test*** | | | | | | | | |
| **group1** | **group2** | **n** | **p** | **p.adj** | | | | **p-adj.signif** |
| **HC** | PD | 121 | 0,00E+00 | <0,0001 | | | | **** |
| **HC** | RBD | 50 | 1,00E-07 | <0,0001 | | | | **** |
| **PD** | RBD | 89 | 1,00E+00 | 1,00E+00 | | | | ns |

| *Kruskall Wallis overall Test* | | | | | | | | |
|---|---|---|---|---|---|---|---|---|
| **term** | **statistic** | **p.value** | **parameter** | **method** | | | | **p-adj.signif** |
| **CSF β-amyloid (Aβ)** | 3,74 | 0,15 | 2 | Kruskal-Wallis rank sum | | | | ns |
| **AGE** | 0,02 | 0,98 | 2 | Kruskal-Wallis rank sum | | | | ns |
| **BMI** | 8,91 | 0,01 | 2 | Kruskal-Wallis rank sum | | | | * |
| **CSF albumin quotient** | 7,94 | 0,02 | 2 | Kruskal-Wallis rank sum | | | | * |
| **MDS-UPDRS-III** | 102,70 | <0,0001 | 2 | Kruskal-Wallis rank sum | | | | **** |
| **MDS-UPDRS total score** | 95,09 | <0,0001 | 2 | Kruskal-Wallis rank sum | | | | **** |
| **MMSE total score** | 2,55 | 0,28 | 2 | Kruskal-Wallis rank sum | | | | ns |
| **MoCa total score** | 8,62 | 0,01 | 2 | Kruskal-Wallis rank sum | | | | * |
| **CSF neurofilament light chains (NfL)** | 16,52 | <0,001 | 2 | Kruskal-Wallis rank sum | | | | *** |
| **Hoehn & Yahr** | 109,39 | <0,0001 | 2 | Kruskal-Wallis rank sum | | | | **** |
| **CSF phospho-tau protein (pTau)** | 5,45 | 0,06 | 2 | Kruskal-Wallis rank sum | | | | ns |
| **CSF Total tau protein (tTau)** | 4,63 | 0,09 | 2 | Kruskal-Wallis rank sum | | | | ns |
| *Dunn Test pairwise post hoc* | | | | | | | | |
| **term** | **.y.** | **group1** | **group2** | **n1** | **n2** | **statistic** | **p.adj** | **p-adj.signif** |
| **CSF β-amyloid (Aβ)** | value | HC | PD | 45 | 88 | 0,06 | 1 | ns |
| **CSF β-amyloid (Aβ)** | value | HC | RBD | 45 | 3 | -1,88 | 0 | ns |
| **CSF β-amyloid (Aβ)** | value | PD | RBD | 88 | 3 | -1,93 | 0 | ns |
| **AGE** | value | HC | PD | 46 | 88 | 0,11 | 1 | ns |
| **AGE** | value | HC | RBD | 46 | 9 | -0,06 | 1 | ns |
| **AGE** | value | PD | RBD | 88 | 9 | -0,12 | 1 | ns |
| **BMI** | value | HC | PD | 46 | 88 | 1,94 | 0 | ns |
| **BMI** | value | HC | RBD | 46 | 9 | -1,50 | 0 | ns |
| **BMI** | value | PD | RBD | 88 | 9 | -2,58 | 0 | * |
| **CSF albumin quotient** | value | HC | PD | 46 | 88 | 1,79 | 0 | ns |
| **CSF albumin quotient** | value | HC | RBD | 46 | 9 | -1,47 | 0 | ns |
| **CSF albumin quotient** | value | PD | RBD | 88 | 9 | -2,46 | 0 | * |

| term | .y. | group1 | group2 | n1 | n2 | statistic | p.adj | p-adj.signif |
|---|---|---|---|---|---|---|---|---|
| **MDS-UPDRS-III** | value | HC | PD | 46 | 88 | 9,90 | 0 | **** |
| **MDS-UPDRS-III** | value | HC | RBD | 46 | 9 | 1,18 | 1 | ns |
| **MDS-UPDRS-III** | value | PD | RBD | 88 | 9 | -3,92 | 0 | *** |
| **MDS-UPDRS total score** | value | HC | PD | 46 | 88 | 9,73 | 0 | **** |
| **MDS-UPDRS total score** | value | HC | RBD | 46 | 9 | 2,52 | 0 | * |
| **MDS-UPDRS total score** | value | PD | RBD | 88 | 9 | -2,43 | 0 | * |
| **MMSE total score** | value | HC | PD | 44 | 87 | -1,60 | 0 | ns |
| **MMSE total score** | value | HC | RBD | 44 | 9 | -0,44 | 1 | ns |
| **MMSE total score** | value | PD | RBD | 87 | 9 | 0,38 | 1 | ns |
| **MoCA total score** | value | HC | PD | 43 | 74 | -2,90 | 0 | * |
| **MoCA total score** | value | HC | RBD | 43 | 9 | -0,51 | 1 | ns |
| **MoCA total score** | value | PD | RBD | 74 | 9 | 1,05 | 1 | ns |
| **CSF neurofilament light chains (NfL)** | value | HC | PD | 46 | 87 | 2,34 | 0 | ns |
| **CSF neurofilament light chains (NfL)** | value | HC | RBD | 46 | 9 | -2,37 | 0 | ns |
| **CSF neurofilament light chains (NfL)** | value | PD | RBD | 87 | 9 | -3,69 | 0 | *** |
| **Hoehn & Yahr** | value | HC | PD | 46 | 88 | 9,97 | <0,001 | **** |
| **Hoehn & Yahr** | value | HC | RBD | 46 | 9 | 0,30 | 1 | ns |
| **Hoehn & Yahr** | value | PD | RBD | 88 | 9 | -4,88 | <0,001 | **** |
| **CSF phospho-tau protein (pTau)** | value | HC | PD | 44 | 85 | -1,08 | 1 | ns |
| **CSF phospho-tau protein (pTau)** | value | HC | RBD | 44 | 3 | -2,25 | 0 | ns |
| **CSF phospho-tau protein (pTau)** | value | PD | RBD | 85 | 3 | -1,94 | 0 | ns |
| **CSF Total tau protein (tTau)** | value | HC | PD | 45 | 88 | -1,32 | 1 | ns |
| **CSF Total tau protein (tTau)** | value | HC | RBD | 45 | 3 | -1,93 | 0 | ns |
| **CSF Total tau protein (tTau)** | value | PD | RBD | 88 | 3 | -1,55 | 0 | ns |

***Supplementary Table 7.*** *Characteristics of the iRBD subjects, iRBD01, iRBD02 and iRBD03 showed phenoconvertion to Parkinson's disease (08/2020; 03/2019; 04/2021) during Follow-up; all subjects showed hyposmia/anosmia indicated by TDI score <30, BDI >8 can indicate a depressive episode, mean NMS of 10 describes mild non-motor symptoms, abbreviations: MMSE: Mini-mental-state examination, sum score; PD-NMS: Parkinson's disease non-motor symptom scale, sum score; Becks depression index, sum score;*

| Subject | Age, date of diagnosis | Sex | Baseline samples? | MMSE | PD-NMS | BDI | Sniffin sticks Threshold | Sniffin sticks discrimination | Sniffin sticks Identification | Sniffin sticks TDI score |
|---|---|---|---|---|---|---|---|---|---|---|
| **iRBD01** | 65; Jun 12 | M | Y | 29 | 6 | 3 | 1,5 | 11 | 3 | 15,5 |
| **iRBD02** | 52; Apr 21 | F | Y | 29 | 13 | 20 | 0 | 5 | 4 | 9 |
| **iRBD03** | 77; Apr 21 | F | Y | 28 | 8 | 6 | 6 | 5 | 1 | 12 |
| **iRBD04** | 71, Jun 13 | M | N | 30 | 15 | 6 | 4 | 12 | 8 | 24 |
| **iRBD05** | 73; Dez 13 | F | Y | 29 | 11 | 11 | 5 | 7 | 8 | 20 |
| **iRBD06** | 68; Mrch 14 | F | Y | 27 | 9 | 13 | 3 | 8 | 10 | 21 |
| **iRBD07** | 62; Mrch 14 | F | Y | 30 | 11 | 7 | 1,5 | 6 | 5 | 12,5 |
| **iRBD08** | 51; Oct 14 | F | Y | 29 | 11 | 7 | 5 | 12 | 13 | 30 |
| **iRBD09** | 63; Apr 15 | M | Y | 28 | 10 | 8 | 0 | 0 | 1 | 1 |
| **iRBD10** | 55; Jun 16 | M | N | 29 | 8 | 9 | - | - | 11 | 11 |
| **iRBD11** | 53; May 16 | M | N | 29 | 8 | 10 | 0,5 | 1 | 2 | 3,5 |
| **iRBD12** | 72; Jun 13 | F | Y | 28 | 14 | 18 | 1,25 | 7 | 5 | 13,25 |
| **Mean** | | | | 28,8 | 10,3 | 9,8 | 2,5 | 6,7 | 5,9 | 14,4 |
| **SD** | | | | 0,9 | 2,7 | 5,0 | 2,2 | 4,0 | 4,0 | 8,3 |
