# Supplementary material for: Lysosomal and synaptic dysfunction markers in longitudinal cerebrospinal fluid of de novo Parkinson’s disease
Source: NPJ Parkinsons Dis. 2024 May 17;10:102. doi: 10.1038/s41531-024-00714-1 (PMC11101466; doi:10.1038/s41531-024-00714-1)
Supplement: Supplementary file 2 — reporting summary [file 41531_2024_714_MOESM2_ESM.pdf]

nature portfolio

Corresponding author(s): Michael Bartl

Last updated by author(s): Apr 9, 2024

Reporting Summary

Nature Portfolio wishes to improve the reproducibility of the work that we publish. This form provides structure for consistency and transparency in reporting. For further information on Nature Portfolio policies, see our Editorial Policies and the Editorial Policy Checklist.

Statistics

For all statistical analyses, confirm that the following items are present in the figure legend, table legend, main text, or Methods section.

n/a	Confirmed	
☐	☒	The exact sample size (*n*) for each experimental group/condition, given as a discrete number and unit of measurement
☐	☒	A statement on whether measurements were taken from distinct samples or whether the same sample was measured repeatedly
☐	☒	The statistical test(s) used AND whether they are one- or two-sided<br>*Only common tests should be described solely by name; describe more complex techniques in the Methods section.*
☐	☒	A description of all covariates tested
☐	☒	A description of any assumptions or corrections, such as tests of normality and adjustment for multiple comparisons
☐	☒	A full description of the statistical parameters including central tendency (e.g. means) or other basic estimates (e.g. regression coefficient) AND variation (e.g. standard deviation) or associated estimates of uncertainty (e.g. confidence intervals)
☐	☒	For null hypothesis testing, the test statistic (e.g. *F*, *t*, *r*) with confidence intervals, effect sizes, degrees of freedom and *P* value noted<br>*Give P values as exact values whenever suitable.*
☒	☐	For Bayesian analysis, information on the choice of priors and Markov chain Monte Carlo settings
☐	☒	For hierarchical and complex designs, identification of the appropriate level for tests and full reporting of outcomes
☐	☒	Estimates of effect sizes (e.g. Cohen's *d*, Pearson's *r*), indicating how they were calculated

Our web collection on statistics for biologists contains articles on many of the points above.

Software and code

Policy information about availability of computer code

Data collection

tudinal data were collected at baseline (BL) and every 24-months over 10 years. This includes extensive clinical investigations andre-evaluation of the diagnosis as well as sample collections including CSF, serum and plasma.

Data analysis

All analyses were performed with the statistical software R (version 4.0.5). Baseline continuous variables were expressed as mean (standard deviation), median, and the range as given by the minimum and maximum values. Group comparisons were performed using the nonparametric Mann–Whitney Kruskall-Wallis test because some of the parameters had nonnormal distribution. For the binary variable “sex”, the count in each category is provided, and the Fisher exact Chi-square tests were used for comparison. Differential expression was assessed using the empirical Bayes approach as implemented in the Bioconductor limma package. Multiple hypothesis testing corrections were performed by using Benjamini and Hochberg’s (BH) false discovery rate at a = 5%. Linear mixed models were used for longitudinal data analysis that allowed fitting only for random intercept models. They were implemented using the function lmer from the cran package lmerTest. The correlation between the assessed proteins and the clinical parameters was assessed via a nonparametric Spearman coefficient using the base R function cor.test from the cran psych package. Here again, the BH procedure was used to correct for multiplicity. To ensure a capture of the performance of every single marker in the longitudinal cohort in this first assessment of this panel in longitudinal de novo PD, we decided to not perform a dimension reduction.

For machine learning, the Boruta algorithm from the CRAN package Boruta was used, and algorithms were built around the random forest classification algorithm. It aims to capture all the relevant features in the dataset concerning the outcome variables of PD versus healthy controls. The algorithm adds randomness to the dataset by creating shuffled copies of all features (Shadow Features) and trains a random forest classifier on the extended dataset (original attributes plus shadow attributes), applying a feature importance measure (The Mean Decrease Accuracy), evaluating the importance of each feature. At every iteration, the Boruta algorithm checks whether a real feature is more important than others and removes features that are marked as highly unimportant. As a stopping rule, we used 100000 iterations with a

maximum of 500 random forests as indicated by the parameter maxRuns in the Boruta function. With actual CRAN implementation of Boruta, warm-up rounds are removed, and the multiple testing corrections are introduced, marking all features that are either strongly or weakly relevant to PD diagnosis.

For manuscripts utilizing custom algorithms or software that are central to the research but not yet described in published literature, software must be made available to editors and reviewers. We strongly encourage code deposition in a community repository (e.g. GitHub). See the Nature Portfolio guidelines for submitting code & software for further information.

Data

Policy information about availability of data

All manuscripts must include a data availability statement. This statement should provide the following information, where applicable:

- Accession codes, unique identifiers, or web links for publicly available datasets
- A description of any restrictions on data availability
- For clinical datasets or third party data, please ensure that the statement adheres to our policy

The authors declare that all data supporting the findings of this study are available within the paper and its Supplementary information files or available from the corresponding author upon request.

Human research participants

Policy information about studies involving human research participants and Sex and Gender in Research.

Reporting on sex and gender

We evaluated the influence of sex in our analyses by comparing sex-adjusted data to non-adjusted data. We adjusted the data for age and sex and calculated the significance in the difference between the disease groups and controls, and compared these results to the significances achieved from the non-adjusted data. Age and sex contributed significantly to the linea mixed model, the results are reported in the manuscript and the supplementary material.

Population characteristics

Recently diagnosed patients with PD and matched healthy controls were enrolled at the Paracelsus-Elena-Klinik, Kassel, Germany between 2009 and 2012. Participants had to be aged between 40 and 85 years old with newly diagnosed PD according to UK Brain Bank Criteria; UKBBC). To be eligible for inclusion, participants had to meet the criteria for de novo PD: any exposure to L-dopa had to have been less than 2 weeks and not within the 4 weeks prior to study entry. Reasons for exclusion were severe vascular encephalopathy, normal-pressure hydrocephalus (NPH) shown on magnetic resonance imaging (MRI) (when available at screening or when detected during imaging studies), evidence for multiple system atrophy (MSA) or progressive supranuclear palsy (PSP) as well as medication-induced PD.

Healthy individuals between 40 and 85 years, matched to the PD group by age, sex, and education level showing no pathological condition of the central nervous system and a negative family history of PD were included as controls. Biannual longitudinal clinical data were collected at baseline and at 2, 4, 6, 8, and 10 years of follow-up in 104 PD and 94 healthy controls. Clinical diagnosis was reassessed in the On-state for all patients at each follow-up by consensus of two teams of independent neurologists.

iRBD was diagnosed through video polysomnography by experienced raters (CT, FS-D, MLM) on two consecutive nights according to established criteria. The LEDD (levodopa equivalent daily dosage) was calculated as previously described.
The iRBD cohort is a part of the DeNoPa cohort, designed as a single center, longitudinal, observational study, still ongoing and recruiting subjects. Follow-up visits take plays every two years for each single subject. Final diagnosis refers to the consensus diagnosis that was made up to 10years of follow-up, including initial dopamine-transporter–single-photon emission computed tomography (DAT-SPECT), biannual clinical evaluations, levodopa challenge, lasting response to levodopa, and the emergence of advanced PD features such as motor fluctuations or levodopa–induced dyskinesias.
Recently diagnosed patients with PD and matched healthy controls were enrolled at the Paracelsus-Elena-Klinik, Kassel, Germany between 2009 and 2012. Participants had to be aged between 40 and 85 years old with newly diagnosed PD according to UK Brain Bank Criteria; UKBBC). To be eligible for inclusion, participants had to meet the criteria for de novo PD: any exposure to L-dopa had to have been less than 2 weeks and not within the 4 weeks prior to study entry. Reasons for exclusion were severe vascular encephalopathy, normal-pressure hydrocephalus (NPH) shown on magnetic resonance imaging (MRI) (when available at screening or when detected during imaging studies), evidence for multiple system atrophy (MSA) or progressive supranuclear palsy (PSP) as well as medication-induced PD.

Healthy individuals between 40 and 85 years, matched to the PD group by age, sex, and education level showing no pathological condition of the central nervous system and a negative family history of PD were included as controls. Biannual longitudinal clinical data were collected at baseline and at 2, 4, 6, 8, and 10 years of follow-up in 104 PD and 94 healthy controls. Clinical diagnosis was reassessed in the On-state for all patients at each follow-up by consensus of two teams of independent neurologists.

iRBD was diagnosed through video polysomnography by experienced raters (CT, FS-D, MLM) on two consecutive nights according to established criteria. The LEDD (levodopa equivalent daily dosage) was calculated as previously described.
The iRBD cohort is a part of the DeNoPa cohort, designed as a single center, longitudinal, observational study, still ongoing and recruiting subjects. Follow-up visits take plays every two years for each single subject. Final diagnosis refers to the consensus diagnosis that was made up to 10years of follow-up, including initial dopamine-transporter–single-photon emission computed tomography (DAT-SPECT), biannual clinical evaluations, levodopa challenge, lasting response to levodopa, and the emergence of advanced PD features such as motor fluctuations or levodopa–induced dyskinesias.

Recruitment

During the recruitment period, we specifically asked the referring neurologists to send de novo PD subjects for a thorough

Recruitment	clinical evaluation in our inpatient hospital. Healthy controls were recruited through relatives and friends of the enrolled subjects and other patients of our clinic as well as through a newspaper advertisement in early 2009. We offered a free health check for all controls. Screening was first performed by a neurologist specializing in movement disorders. Subjects meeting the inclusion criteria were evaluated by an independent second movement disorder specialist and received a standardized program of investigations.
Ethics oversight	Approval was received from the local ethical standards committee on human experimentation for all human participants in all cohorts (FF 89/2008, FF 130/2012, MC 310/2010). Written informed consent for research was obtained from all study participants. DeNoPa is registered in the German Register for Clinical trials (DRKS00000540),

Note that full information on the approval of the study protocol must also be provided in the manuscript.

Field-specific reporting

Please select the one below that is the best fit for your research. If you are not sure, read the appropriate sections before making your selection.

☒ Life sciences ☐ Behavioural & social sciences ☐ Ecological, evolutionary & environmental sciences

For a reference copy of the document with all sections, see nature.com/documents/nr-reporting-summary-flat.pdf

Life sciences study design

All studies must disclose on these points even when the disclosure is negative.

Sample size	We analyzed CSF samples from subjects of our longitudinal de novo PD (DeNoPa) cohort and included, from baseline , 88 PD patients (62 men, 70.5 %) and 46 healthy controls (HC) (34 men, 73.9 %) for whom CSF samples were available at all time points. Further, we included an exploratory test set of nine subjects at BL with iRBD (2 men, 2.2 %) mean age 65 (± 9).<br>To increase the power of our longitudinal model, we also included additional samples from subjects with no available CSF samples at baseline, for which there were no CSF samples available at baseline. This resulted in a total of 12 iRBD (11 of 12 αS-SAA positive), 104 PD (876 of 100 αS-SAA positive), and 58 HC (all negative) subjects in our longitudinal model
Data exclusions	Extreme outliers, data points deviating more than ten median absolute deviations, were excluded.
Replication	The exploratory iRBD cohort was used as replication cohort for the baseline and longitudinal measurements of the PD/HC cohort
Randomization	For instrumental analysis, the samples were randomised by the "constrained randomisation" strategy, thus randomising within and between blocks of samples consisting of all conditions.
Blinding	The investigators preparing the samples for analysis were blinded. The investigators performing the instrumental analysis were unblinded to be able to construct the constrained randomisation described above.

Behavioural & social sciences study design

All studies must disclose on these points even when the disclosure is negative.

Study description	*Briefly describe the study type including whether data are quantitative, qualitative, or mixed-methods (e.g. qualitative cross-sectional, quantitative experimental, mixed-methods case study).*
Research sample	*State the research sample (e.g. Harvard university undergraduates, villagers in rural India) and provide relevant demographic information (e.g. age, sex) and indicate whether the sample is representative. Provide a rationale for the study sample chosen. For studies involving existing datasets, please describe the dataset and source.*
Sampling strategy	*Describe the sampling procedure (e.g. random, snowball, stratified, convenience). Describe the statistical methods that were used to predetermine sample size OR if no sample-size calculation was performed, describe how sample sizes were chosen and provide a rationale for why these sample sizes are sufficient. For qualitative data, please indicate whether data saturation was considered, and what criteria were used to decide that no further sampling was needed.*
Data collection	*Provide details about the data collection procedure, including the instruments or devices used to record the data (e.g. pen and paper, computer, eye tracker, video or audio equipment) whether anyone was present besides the participant(s) and the researcher, and whether the researcher was blind to experimental condition and/or the study hypothesis during data collection.*
Timing	*Indicate the start and stop dates of data collection. If there is a gap between collection periods, state the dates for each sample cohort.*
Data exclusions	*If no data were excluded from the analyses, state so OR if data were excluded, provide the exact number of exclusions and the rationale behind them, indicating whether exclusion criteria were pre-established.*

Non-participation	*State how many participants dropped out/declined participation and the reason(s) given OR provide response rate OR state that no participants dropped out/declined participation.*
Randomization	*If participants were not allocated into experimental groups, state so OR describe how participants were allocated to groups, and if allocation was not random, describe how covariates were controlled.*

Ecological, evolutionary & environmental sciences study design

All studies must disclose on these points even when the disclosure is negative.

Study description	*Briefly describe the study. For quantitative data include treatment factors and interactions, design structure (e.g. factorial, nested, hierarchical), nature and number of experimental units and replicates.*
Research sample	*Describe the research sample (e.g. a group of tagged Passer domesticus, all Stenocereus thurberi within Organ Pipe Cactus National Monument), and provide a rationale for the sample choice. When relevant, describe the organism taxa, source, sex, age range and any manipulations. State what population the sample is meant to represent when applicable. For studies involving existing datasets, describe the data and its source.*
Sampling strategy	*Note the sampling procedure. Describe the statistical methods that were used to predetermine sample size OR if no sample-size calculation was performed, describe how sample sizes were chosen and provide a rationale for why these sample sizes are sufficient.*
Data collection	*Describe the data collection procedure, including who recorded the data and how.*
Timing and spatial scale	*Indicate the start and stop dates of data collection, noting the frequency and periodicity of sampling and providing a rationale for these choices. If there is a gap between collection periods, state the dates for each sample cohort. Specify the spatial scale from which the data are taken*
Data exclusions	*If no data were excluded from the analyses, state so OR if data were excluded, describe the exclusions and the rationale behind them, indicating whether exclusion criteria were pre-established.*
Reproducibility	*Describe the measures taken to verify the reproducibility of experimental findings. For each experiment, note whether any attempts to repeat the experiment failed OR state that all attempts to repeat the experiment were successful.*
Randomization	*Describe how samples/organisms/participants were allocated into groups. If allocation was not random, describe how covariates were controlled. If this is not relevant to your study, explain why.*
Blinding	*Describe the extent of blinding used during data acquisition and analysis. If blinding was not possible, describe why OR explain why blinding was not relevant to your study.*

Did the study involve field work? ☐ Yes ☐ No

Field work, collection and transport

Field conditions	*Describe the study conditions for field work, providing relevant parameters (e.g. temperature, rainfall).*
Location	*State the location of the sampling or experiment, providing relevant parameters (e.g. latitude and longitude, elevation, water depth).*
Access & import/export	*Describe the efforts you have made to access habitats and to collect and import/export your samples in a responsible manner and in compliance with local, national and international laws, noting any permits that were obtained (give the name of the issuing authority, the date of issue, and any identifying information).*
Disturbance	*Describe any disturbance caused by the study and how it was minimized.*

Reporting for specific materials, systems and methods

We require information from authors about some types of materials, experimental systems and methods used in many studies. Here, indicate whether each material, system or method listed is relevant to your study. If you are not sure if a list item applies to your research, read the appropriate section before selecting a response.

Materials & experimental systems

n/a	Involved in the study
☒	☐ Antibodies
☒	☐ Eukaryotic cell lines
☒	☐ Palaeontology and archaeology
☒	☐ Animals and other organisms
☐	☒ Clinical data
☒	☐ Dual use research of concern

Methods

n/a	Involved in the study
☒	☐ ChIP-seq
☒	☐ Flow cytometry
☒	☐ MRI-based neuroimaging

Antibodies

Antibodies used

Describe all antibodies used in the study; as applicable, provide supplier name, catalog number, clone name, and lot number.

Validation

Describe the validation of each primary antibody for the species and application, noting any validation statements on the manufacturer's website, relevant citations, antibody profiles in online databases, or data provided in the manuscript.

Eukaryotic cell lines

Policy information about cell lines and Sex and Gender in Research

Cell line source(s)

State the source of each cell line used and the sex of all primary cell lines and cells derived from human participants or vertebrate models.

Authentication

Describe the authentication procedures for each cell line used OR declare that none of the cell lines used were authenticated.

Mycoplasma contamination

Confirm that all cell lines tested negative for mycoplasma contamination OR describe the results of the testing for mycoplasma contamination OR declare that the cell lines were not tested for mycoplasma contamination.

Commonly misidentified lines
(See ICLAC register)

Name any commonly misidentified cell lines used in the study and provide a rationale for their use.

Palaeontology and Archaeology

Specimen provenance

Provide provenance information for specimens and describe permits that were obtained for the work (including the name of the issuing authority, the date of issue, and any identifying information). Permits should encompass collection and, where applicable, export.

Specimen deposition

Indicate where the specimens have been deposited to permit free access by other researchers.

Dating methods

If new dates are provided, describe how they were obtained (e.g. collection, storage, sample pretreatment and measurement), where they were obtained (i.e. lab name), the calibration program and the protocol for quality assurance OR state that no new dates are provided.

☐ Tick this box to confirm that the raw and calibrated dates are available in the paper or in Supplementary Information.

Ethics oversight

Identify the organization(s) that approved or provided guidance on the study protocol, OR state that no ethical approval or guidance was required and explain why not.

Note that full information on the approval of the study protocol must also be provided in the manuscript.

Animals and other research organisms

Policy information about studies involving animals; ARRIVE guidelines recommended for reporting animal research, and Sex and Gender in Research

Laboratory animals

For laboratory animals, report species, strain and age OR state that the study did not involve laboratory animals.

Wild animals

Provide details on animals observed in or captured in the field; report species and age where possible. Describe how animals were caught and transported and what happened to captive animals after the study (if killed, explain why and describe method; if released, say where and when) OR state that the study did not involve wild animals.

Reporting on sex

Indicate if findings apply to only one sex; describe whether sex was considered in study design, methods used for assigning sex. Provide data disaggregated for sex where this information has been collected in the source data as appropriate; provide overall numbers in this Reporting Summary. Please state if this information has not been collected. Report sex-based analyses where performed, justify reasons for lack of sex-based analysis.

Field-collected samples | *For laboratory work with field-collected samples, describe all relevant parameters such as housing, maintenance, temperature, photoperiod and end-of-experiment protocol OR state that the study did not involve samples collected from the field.*

Ethics oversight | *Identify the organization(s) that approved or provided guidance on the study protocol, OR state that no ethical approval or guidance was required and explain why not.*

Note that full information on the approval of the study protocol must also be provided in the manuscript.

Clinical data

Policy information about clinical studies

All manuscripts should comply with the ICMJE guidelines for publication of clinical research and a completed CONSORT checklist must be included with all submissions.

Clinical trial registration | Institutional review board statements were obtained from the University Medical Centre in Goettingen, Germany Approval No. 9/7/04 and 36/7/02. The study was conducted according to the Declaration of Helsinki and all participants gave written informed consent.

Study protocol | Additional information can be found here: https://drks.de/search/de/trial/DRKS00000540

Data collection | Biannual longitudinal data were collected at baseline (BL) and every 24-months over 10 years. This includes extensive clinical investigations andre-evaluation of the diagnosis as well as sample collections including CSF, serum and plasma.

Outcomes | DeNoPa is a non-interventional diagnostic study. One outcome measure is the convertion frm iRBD to neuronal synuclein disease.

Dual use research of concern

Policy information about dual use research of concern

Hazards

Could the accidental, deliberate or reckless misuse of agents or technologies generated in the work, or the application of information presented in the manuscript, pose a threat to:

No	Yes	
☐	☐	Public health
☐	☐	National security
☐	☐	Crops and/or livestock
☐	☐	Ecosystems
☐	☐	Any other significant area

Experiments of concern

Does the work involve any of these experiments of concern:

No	Yes	
☐	☐	Demonstrate how to render a vaccine ineffective
☐	☐	Confer resistance to therapeutically useful antibiotics or antiviral agents
☐	☐	Enhance the virulence of a pathogen or render a nonpathogen virulent
☐	☐	Increase transmissibility of a pathogen
☐	☐	Alter the host range of a pathogen
☐	☐	Enable evasion of diagnostic/detection modalities
☐	☐	Enable the weaponization of a biological agent or toxin
☐	☐	Any other potentially harmful combination of experiments and agents

ChIP-seq

Data deposition

☐ Confirm that both raw and final processed data have been deposited in a public database such as GEO.

☐ Confirm that you have deposited or provided access to graph files (e.g. BED files) for the called peaks.

Data access links
May remain private before publication. | *For "Initial submission" or "Revised version" documents, provide reviewer access links. For your "Final submission" document, provide a link to the deposited data.*

Files in database submission | *Provide a list of all files available in the database submission.*

Genome browser session
(e.g. UCSC)

Provide a link to an anonymized genome browser session for "Initial submission" and "Revised version" documents only, to enable peer review. Write "no longer applicable" for "Final submission" documents.

Methodology

Replicates | *Describe the experimental replicates, specifying number, type and replicate agreement.*

Sequencing depth | *Describe the sequencing depth for each experiment, providing the total number of reads, uniquely mapped reads, length of reads and whether they were paired- or single-end.*

Antibodies | *Describe the antibodies used for the ChIP-seq experiments; as applicable, provide supplier name, catalog number, clone name, and lot number.*

Peak calling parameters | *Specify the command line program and parameters used for read mapping and peak calling, including the ChIP, control and index files used.*

Data quality | *Describe the methods used to ensure data quality in full detail, including how many peaks are at FDR 5% and above 5-fold enrichment.*

Software | *Describe the software used to collect and analyze the ChIP-seq data. For custom code that has been deposited into a community repository, provide accession details.*

Flow Cytometry

Plots

Confirm that:

☐ The axis labels state the marker and fluorochrome used (e.g. CD4-FITC).

☐ The axis scales are clearly visible. Include numbers along axes only for bottom left plot of group (a 'group' is an analysis of identical markers).

☐ All plots are contour plots with outliers or pseudocolor plots.

☐ A numerical value for number of cells or percentage (with statistics) is provided.

Methodology

Sample preparation | *Describe the sample preparation, detailing the biological source of the cells and any tissue processing steps used.*

Instrument | *Identify the instrument used for data collection, specifying make and model number.*

Software | *Describe the software used to collect and analyze the flow cytometry data. For custom code that has been deposited into a community repository, provide accession details.*

Cell population abundance | *Describe the abundance of the relevant cell populations within post-sort fractions, providing details on the purity of the samples and how it was determined.*

Gating strategy | *Describe the gating strategy used for all relevant experiments, specifying the preliminary FSC/SSC gates of the starting cell population, indicating where boundaries between "positive" and "negative" staining cell populations are defined.*

☐ Tick this box to confirm that a figure exemplifying the gating strategy is provided in the Supplementary Information.

Magnetic resonance imaging

Experimental design

Design type | *Indicate task or resting state; event-related or block design.*

Design specifications | *Specify the number of blocks, trials or experimental units per session and/or subject, and specify the length of each trial or block (if trials are blocked) and interval between trials.*

Behavioral performance measures | *State number and/or type of variables recorded (e.g. correct button press, response time) and what statistics were used to establish that the subjects were performing the task as expected (e.g. mean, range, and/or standard deviation across subjects).*

Acquisition

Imaging type(s) | *Specify: functional, structural, diffusion, perfusion.*

Field strength | *Specify in Tesla*

Sequence & imaging parameters | *Specify the pulse sequence type (gradient echo, spin echo, etc.), imaging type (EPI, spiral, etc.), field of view, matrix size, slice thickness, orientation and TE/TR/flip angle.*

Area of acquisition | *State whether a whole brain scan was used OR define the area of acquisition, describing how the region was determined.*

Diffusion MRI ☐ Used ☐ Not used

Preprocessing

Preprocessing software | *Provide detail on software version and revision number and on specific parameters (model/functions, brain extraction, segmentation, smoothing kernel size, etc.).*

Normalization | *If data were normalized/standardized, describe the approach(es): specify linear or non-linear and define image types used for transformation OR indicate that data were not normalized and explain rationale for lack of normalization.*

Normalization template | *Describe the template used for normalization/transformation, specifying subject space or group standardized space (e.g. original Talairach, MNI305, ICBM152) OR indicate that the data were not normalized.*

Noise and artifact removal | *Describe your procedure(s) for artifact and structured noise removal, specifying motion parameters, tissue signals and physiological signals (heart rate, respiration).*

Volume censoring | *Define your software and/or method and criteria for volume censoring, and state the extent of such censoring.*

Statistical modeling & inference

Model type and settings | *Specify type (mass univariate, multivariate, RSA, predictive, etc.) and describe essential details of the model at the first and second levels (e.g. fixed, random or mixed effects; drift or auto-correlation).*

Effect(s) tested | *Define precise effect in terms of the task or stimulus conditions instead of psychological concepts and indicate whether ANOVA or factorial designs were used.*

Specify type of analysis: ☐ Whole brain ☐ ROI-based ☐ Both

Statistic type for inference (See Eklund et al. 2016) | *Specify voxel-wise or cluster-wise and report all relevant parameters for cluster-wise methods.*

Correction | *Describe the type of correction and how it is obtained for multiple comparisons (e.g. FWE, FDR, permutation or Monte Carlo).*

Models & analysis

n/a	Involved in the study
☐	☐ Functional and/or effective connectivity
☐	☐ Graph analysis
☐	☐ Multivariate modeling or predictive analysis

Functional and/or effective connectivity | *Report the measures of dependence used and the model details (e.g. Pearson correlation, partial correlation, mutual information).*

Graph analysis | *Report the dependent variable and connectivity measure, specifying weighted graph or binarized graph, subject- or group-level, and the global and/or node summaries used (e.g. clustering coefficient, efficiency, etc.).*

Multivariate modeling and predictive analysis | *Specify independent variables, features extraction and dimension reduction, model, training and evaluation metrics.*
